# Supplementary material for: Detection of offensive terms in resource-poor language using machine learning algorithms
Source: PeerJ Comput Sci. 2023 Aug 29;9:e1524. doi: 10.7717/peerj-cs.1524 (PMC10496005; doi:10.7717/peerj-cs.1524)
Supplement: Supplemental Information 1 — This dataset is gathered from Twitter. It contains 3 columns 1 ID that distinguishes each tweet from one another, a tweet column contains the text of the tweet and target contains 0 and 1 to represent if the tweet is abusive or not. Dataset 1 is used to perform experiments in this study. In scenario 1 in the result section it serves as training datasets. [file peerj-cs-09-1524-s001.htm]

Resource-Poor-Language-Urdu-Dataset/Dataset 1.csv at main · owais4321/Resource-Poor-Language-Urdu-Dataset · GitHub


Skip to content


Toggle navigation

Sign up

- Product

  - Actions

    Automate any workflow
  - Packages

    Host and manage packages
  - Security

    Find and fix vulnerabilities
  - Codespaces

    Instant dev environments
  - Copilot

    Write better code with AI
  - Code review

    Manage code changes
  - Issues

    Plan and track work
  - Discussions

    Collaborate outside of code
  - Explore
  - All features
  - Documentation
  - GitHub Skills
  - Blog
- Solutions

  - For
  - Enterprise
  - Teams
  - Startups
  - Education
  - By Solution
  - CI/CD & Automation
  - DevOps
  - DevSecOps
  - Case Studies
  - Customer Stories
  - Resources
- Open Source

  - GitHub Sponsors

    Fund open source developers
  - The ReadME Project

    GitHub community articles
  - Repositories
  - Topics
  - Trending
  - Collections
- Pricing

- In this repository

  All GitHub
  ↵

  Jump to
  ↵

- No suggested jump to results

- In this repository

  All GitHub
  ↵

  Jump to
  ↵
- In this user

  All GitHub
  ↵

  Jump to
  ↵
- In this repository

  All GitHub
  ↵

  Jump to
  ↵

Sign in

Sign up

{{ message }}

owais4321
/
**Resource-Poor-Language-Urdu-Dataset**
Public

- Notifications
- Fork
  0
- Star
   0

- Code
- Issues
  0
- Pull requests
  0
- Actions
- Projects
  0
- Security
- Insights

More


- Code
- Issues
- Pull requests
- Actions
- Projects
- Security
- Insights

Permalink

main

Switch branches/tags


Branches
Tags

Could not load branches


Nothing to show

{{ refName }}
default
View all branches

Could not load tags


Nothing to show


{{ refName }}
default
View all tags

# Name already in use

A tag already exists with the provided branch name. Many Git commands accept both tag and branch names, so creating this branch may cause unexpected behavior. Are you sure you want to create this branch?

 Cancel
 Create

## Resource-Poor-Language-Urdu-Dataset/**Dataset 1.csv**

 Go to file

 

- Go to file
  T
- Go to line
  L
- Copy path
- Copy permalink

This commit does not belong to any branch on this repository, and may belong to a fork outside of the repository.

Cannot retrieve contributors at this time

2401 lines (2401 sloc)
323 KB

Raw
  Blame

Edit this file

E


Open in GitHub Desktop

- Open with Desktop
- View raw
- Copy raw contents
   Copy raw contents

   Copy raw contents

   Copy raw contents
- View blame

This file contains bidirectional Unicode text that may be interpreted or compiled differently than what appears below. To review, open the file in an editor that reveals hidden Unicode characters.
Learn more about bidirectional Unicode characters

Show hidden characters


|  | id | tweet | target |
| --- | --- | --- | --- |
|  | 1024 | قوم کے لیے پرانے خاندانی نمک حرام ہیں | 1 |
|  | 1919 | اور پھر بھی ان خنزیروں کو بغض نواز میں سپورٹ کرتا رہا اس کو ان سے… | 0 |
|  | 102 | پاکستان کے قبضے میں اسرائیلی پائلٹ ہے۔ | 0 |
|  | 679 | اور نواز شریف کا نواسہ مستقبل کا وزیر اعظم | 0 |
|  | 2093 | صرف ٹویٹر پہ بکواس کرنا ہی آسان ہے | 0 |
|  | 1028 | پاکستان کشمیوں کو مشکل وقت میں تنہا نہیں چھوڑے گا، جبکہ سندھیوں، بلوچوں، پشتونوں کو زندہ نہیں چھوڑے گا | 0 |
|  | 2097 | ایسے مادرچود یوتھئے ہیں کہ کشمیر ہاتھہ سے نکل رہا ہے لیکن یوتھئے ابھی بھی کشمیر کی ثالثی پر نیازی کی واہ واہ کررہے | 1 |
|  | 2297 | نکل بھوسڑی کے مادرچود گانڈو | 1 |
|  | 2572 | کشمیر بیچنے والے کتو | 1 |
|  | 3313 | یہ ہے عالمی سازش اور ہمارےسلیکٹڈ اور سلیکٹرز کےمنہ پر سعودی عرب کا شرمناک طمانچہ | 0 |
|  | 2597 | کسی کتی کی بچے، مادرچود بہت ہی گھٹیا ہو تم، حرام کی پیداوار ہو تم | 1 |
|  | 1893 | یہ کیا بکواس ہے اس سے بہتر ہے انکو گھر بھیج دیں ان کنجروں کی تو موجیں لگی ہوئی ہیں نیب کیا ڈرامہ کر رہا ہے | 1 |
|  | 405 | آج میرا دل خدا کی قسم خون کے آنسو رویا ہے جب میں نے قبرستان میں قبروں کی یہ حالت دیکھی ہے | 0 |
|  | 1232 | لعنت تمھارے ٹوٹ کرنے پر بے شرمی کی انتہا ہے تمھارے اندر۔ | 1 |
|  | 2092 | یہ رنڈی پاکستان وآپس آئے تو عوام کو چاہیئے جہاں بھی نظر آئے اس کو پکڑ کہ اس رنڈی کی گانڈ کا کیڑا مارے | 1 |
|  | 896 | جاو اور جاکر کشمیر میں کشمیریوں کے ساتھ مل کر جنگ کرو۔… | 0 |
|  | 807 | جنرل باجوہ کا نام سن کر مودی کی ٹانگیں کانپتی ہیں آرمی چیف نے پُلوامہ میں بھارتی فوج کو ڈنڈا دیا | 0 |
|  | 1315 | کیسی خبیث انسان تھی جسکے مرنے کی ہمیں خوشی ہوئی ہے یہ لعنتی مودی پتا نہیں کب مرے گا کتے کی موت ۔ | 1 |
|  | 916 | مجھے یہ مادرچود شروع سے ہی برا لگتا تھا ، زندگی کا پہلا ووٹ دیا اس اس بار سلیکشن میں | 1 |
|  | 2730 | گورایہ گٹر کی پیداوار | 1 |
|  | 944 | فوجیستان بس ایک ہی کام آتا ھےالطاف بھائی کےاوپر پابندی لگا دو گھر پر تالا ڈال دو | 0 |
|  | 1305 | مریم نواز کو احتساب عدالت بلا کر صرف سیکنڈز میں دن کا ریمانڈ دلوانا ہی تو تیز ترین کاروائی ہے | 0 |
|  | 1249 | یہ وہ مادرچود ہیں جن کی پارلمنٹ کی بلڈنگ بھی پاکستان نے بھیک میں بنا کے دی تھی | 1 |
|  | 331 | اپنی بکواس بند کرو | 1 |
|  | 1746 | گھسرا گھسرے کی گانڈ مارے تو مزہ کس کو آۓ گا | 1 |
|  | 560 | بھول کر بھی تم لوگوں کی زبان پر نہیں آئے گا | 0 |
|  | 159 | بشری پیرنی کہاں گئی جس کو بنی گالہ کے کتوں کے آنسو تو نظر آتے ہیں ہیں مگر لاکھوں مظلوم کشمیریوں کے آنسو اور خون نہیں | 0 |
|  | 925 | ممتاز اے اپنی ماں دی پھودی چ دے جا کے | 1 |
|  | 3037 | اس کو کس مادرچود حرامی نے پاکستان بلوایا اس کی گ میں ڈنڈا ڈال کر اس بھی میکاسنگھ کے ساتھ انڈیا بھیج دو | 1 |
|  | 324 | کرنٹ اکاؤنٹ خسارہ میں کمی بڑی کامیابی ہے، وزیراعظم | 0 |
|  | 3025 | تیرے جیسے کنجروں کا خاتمہ ہونے والا ہے پاکستان سے انشاءلله | 0 |
|  | 213 | یہ گانڈو خود کو سندھی بولتا ھے مگر فخر بلوچ ھونے پر کرتا ھے اور خود کا بلوچست… | 1 |
|  | 1221 | کاش اج وہ زندہ ہوتا | 0 |
|  | 301 | اب موچھیں کاٹ کے پچھواڑا پھاڑ کے بھیجیں گے | 1 |
|  | 801 | جانی میں طوفانی بارش نے تباہی مچا دی ، سینکڑوں مکانات کو نقصان ، سیلابی ریلے سے عوام محصور | 0 |
|  | 1716 | محترم جناب اگر آپ انکل باجرہ کی ایکسٹینشن پر ٹیوٹ نہ کرتے تو کوئی قیامت نہیں آگئی تھی،آپ اپنے نظریات پر پہرہ دیں | 0 |
|  | 270 | کمینی منہ بند رکھ اپنا | 1 |
|  | 1595 | سچ اور حق بولنے اور لکھنے والا نڈر انسان بیوروچیف ٹرائیبل ٹائمز اور ڈسٹرک ویسٹ بلدیہ ٹاون کراچی میںشہید | 0 |
|  | 1263 | تمہاری تو اوقات ہی نہیں مادر چود فوج کو سامنے لا کر like لیتا ھے خنزیر کی اولاد جتنا یہ ملک تمہارا ھے | 1 |
|  | 980 | نہیں میری جان ایسی کوئی بات نہیں ہے۔ بس مزاق کرتے ہیں۔ تیری ماں میری ماں ہے۔ | 0 |
|  | 0 | بھگوڑی لیگ پر پورے کشمیروں کی لعنت | 1 |
|  | 1603 | بکواس کے علاوہ بھی کچھ بھیج سکتا ہے؟ | 0 |
|  | 2221 | چھوڑ کر غلط کر رہے ہیں برگہ کشتہ روز ِاول پہلے ہی دن طبیعت صاف کر دینی چاہئیے | 0 |
|  | 2589 | ایسے لعنتییوں کو سیاست میں نہ لیکر آتے اسٹیبلشمنٹ اور بار بار ہر کیسیس پر اور ہر کالے کرتوتوں پر نہ بچاتے | 1 |
|  | 3345 | بہن چود قومی اثاثے برباد کر رھا ھے | 1 |
|  | 2688 | خبیث انسان اس موقع پر تو اپنی سیاست چمکانے میں لگا ہوا ہے حرامی کی نسل کچھ شرم نہیں تم لوگوں میں | 1 |
|  | 2178 | تیری بہن کو چود رہا ہوں ابھی پھر تیرے ابے کی گانڈ مار کے تیری ماں کو چودوں گا | 1 |
|  | 965 | یار افغانیوں کی ماں بہن کی چدائی کرنے کا بہت مزہ آتا ہے بہت سفید ہوتی ہیں | 0 |
|  | 3300 | پتہ نہیں کسے ڈرا رہے ہیں انڈیا کو یا اپنے ملک کو ایسے غلیظ بیان انڈیا کو دینے والے کتے کو حکومت کیوں نہیں پکڑتے | 1 |
|  | 1033 | پاک فوج کا لائن آف کنٹرول پر بھارتی اشتعال انگیزی کا منہ توڑ جواب پاک فوج کی کارروائی میں افسر سمیت بھارتی فوجی ہلاک، آئی ایس پی آر | 0 |
|  | 2553 | خدا وہ وقت نہ لائے کہ سوگوار ہو تو | 0 |
|  | 3087 | تم اتنے کسی کنجری ماں کے بچے ہو۔ گشتی ماں بچے۔ بہن چود دلے۔ جا کے انڈیا کی جھولی میں بیٹھ جا۔ تیری ماں ل۔۔۔ کتے کے بچے | 1 |
|  | 3497 | ایسے نہیں ایسے سن زراہ غور سے حرامی | 1 |
|  | 1478 | او دلے کسی گشتی کی حرامی نسل تیرا بھونکنا بند نہیں ہوگا لیکن کسی دن اس کی طرح عوام کے ہتھے آیا تو مر جاۓ گا | 0 |
|  | 4 | عبدالقدیر خان نے کہا کارگل جنگ میں ہم دہلی صرف منٹ میں ایک بٹن کی کلک سے تباہ کرسکتے تھے۔۔یہ تو ہے… | 0 |
|  | 1268 | لکھتے ریے مجھے تو تمہارے حلال زادے ہونے پر شک ہے | 1 |
|  | 2413 | کارتک کی اچھی خاصی طبیعت صاف ہو چکی ہے کمنٹس میں لہذا میں کچھ نہیں کہوںگا | 0 |
|  | 630 | بریکنگ نیوز نیب کو اچانک شاندار کامیابی مل گئی ، زرداری اینڈ کمپنی کی کمر توڑ کر رکھ دینے والی خبر | 0 |
|  | 644 | پاگل تم ہو گی زرا منہ سنبھال کر بات کرو | 0 |
|  | 893 | کسی بدکردار ماں باپ کے بچے نیازی کی واشل بکواس بند کرو | 1 |
|  | 2003 | کیا گدھا راگنی شروع کی ہوئی ہے آپ سب گدھا خوروں نے، گدھی کے بچو | 1 |
|  | 2076 | یہ بہت بکواس کرنے لگ گئے ہیں اپنی باری ان کو لال مرچیں لڑتی ہیں گائے کا پیشاپ پینے والی گندی قوم | 1 |
|  | 2616 | ہم کشمیری اللہؐ کا لاکھ لاکھ شکر اداکرتے ہیں ان ضمیر فروشوں سے ہماری اور پاکستانیوں کی جان چھوٹی | 0 |
|  | 1222 | علماء کرام آقا علیہ السلام کے وارث ہیں ان کتوں کی کیا حثیت ہے جو علماء کرام کے بارے میں بکواس کر رہے ہ… | 0 |
|  | 867 | کسے ٹیکسی دے | 1 |
|  | 3434 | لاوارث گٹر کی پیداوار | 1 |
|  | 812 | دنیا کا بہترین ہتھیار ایمانداری دنیا کی سب سے تیز تلوار بھی دیانتداری | 0 |
|  | 1194 | او کتے کی بچی حرام خور پاکستان کے جھنڈے کو ناپاک مت کر کتی | 1 |
|  | 3016 | کتے کو وہ کبھی شیر نہیں کہتے کتا ہی کہتے ہیں | 1 |
|  | 1271 | اب وہ حضرات جو مسلم اُمّہ کے سُحانے خواب دیکھتے رہتے ہیں، جاگ جائیں۔ یہ ایک طمانچہ ہے پوری اُمت کے منہ پر۔ | 0 |
|  | 2459 | میٹرو پشاور پر کام اور ن لیگی میڈیا ٹیم کے پھڈے تا قیامت جاری رہیں گے۔ | 0 |
|  | 378 | بہن چود کنجر بیغیرت بیغیرت بہن چود کنجر بیغیرت بیغیرت بہن چود کنجر بیغیرت بیغیرت بہن چود کنجر بیغیرت | 1 |
|  | 2289 | اور جس طرح سے تم بیغیرتوں نے عوام کو بے دردی سے لُوٹا ہے اس سے تم لوگوں کا حرامی پن نظر آتا ہے | 1 |
|  | 1817 | مادرچود نیب کی گاڑی کے آگے لیٹا کیوں نہیں تو | 1 |
|  | 1386 | ایک منٹ میں سوات سے ہوں اور دوسرے منٹ میں وزیرستانی بن گئے۔ کچھ شرم کرو | 0 |
|  | 2950 | حرام خوروں سے ایسے دلال بہتر ہیں | 1 |
|  | 3421 | کشمیر بنےگا پاکستان ۔۔۔۔۔ یارب العزت پاکستان کی شہ رگ کشمیر کو جلدازجلد بھارت کی قابض افواج سے آزادی نصیب فرماء آمی… | 0 |
|  | 2295 | نکل بھوسڑی کے مادرچود گانڈو | 0 |
|  | 314 | اور تمہارے جیسے کتوں کی بوری یہی ادارے ہیں جن کی وجہ سے انہیں پر بھونک کر بھی عوام سے بچے رہتے ہو۔ | 1 |
|  | 446 | تو اپنی بکواس بند رکھ بےغیرت انسان | 1 |
|  | 2821 | حاجی جامل حالتی انتقال کرگئے نماز جنازہ گیارہ بجے نڑیل پیر قبرستان میں ادا کی جائےگی | 0 |
|  | 3371 | ایک تو یہ بھی تیرا ٹھوکو تھا ویسے گانڈ مرواتے مرواتے تع دلال بھی بن گیا اور پھر مشہور زمانہ گانڈو دل | 1 |
|  | 518 | یہی ان بےغیرتوں کی پہچان ہے | 1 |
|  | 3267 | دوستو یہ رنڈی اس قابل نہیں کہ اسکے بکواس سنو | 1 |
|  | 3009 | اب اس قوم کا اللہ ہی حافظ ہے بلاتحقیق اپنے وزیر خارجہ پر ایک خبیث انسان کے کہنے پر دشنام طرازی کر رہے ہیں | 1 |
|  | 2893 | سر اب جوابي كارواي نهى انكو سبق سكها دينا | 1 |
|  | 2036 | ان بیغیرتوں کی حکومت بہنوں اور بیٹیوں کو جیل نہیں بھیجتی، یہ سیدھا منہ پر گولی مارتے ہیں | 0 |
|  | 1907 | یہ تمھارے ماں کی کوس ہورہاہے بہن چود یہ چور ہے چور کو چایئے دن میں سزا ہو یا رات میں سر کٹ جائے یا ہات | 1 |
|  | 1999 | ہولناک خبریں پڑھ کر گھنٹوں اداس رہتی ہوں پاکستانیوں حیا کرو یار ایک معصوم سے بچے کی جان لے لی مرے نہیں تم؟ | 0 |
|  | 3404 | مادرچود کیا الگ گیراج میں لے جا کے گرفتار کرتے۔ کھوتی کے بچو اب پرانی عادتیں بھول جاؤ | 1 |
|  | 149 | خاموشی اختیار کرلیتا تھا وہ مسلمان ضیاء کی موت پر جشن منارہے | 0 |
|  | 2312 | پاکستان کو اپنی نظریاتی سرحدوں پہ کام کرنے کی اشد ضرورت ہے | 0 |
|  | 2304 | یا اللہ پاک بیشک ھم بہت گناہگار سیاہکار ھے | 0 |
|  | 1320 | دیکھیں حامد ماضی میں ایکسٹنشنز کے بارے میں جو کچھ میں نے کہا وہ نری بکواس تھی۔۔۔ | 0 |
|  | 2601 | اوئے مریم کے گمشدہ کتورے ،اوہ بیغیرتوں کا اک پورا میگزین، شکل دیکھ اپنی آئینے | 0 |
|  | 58 | یہ سب نہیں کر سکتے توآپکا اخلاقی فرض ہے اپنی بکواس بند رکھے اور فوج کے خلاف بات مت کریں | 0 |
|  | 258 | گھٹیا الزام لگائے تمہاری ماں | 1 |
|  | 3265 | گا ئے کا بیشاب پینے والے کتے | 1 |
|  | 3496 | آپکے لاکھوں فوجی کشمیر میں لوگوں کو مارے وہ صحیح اور ھمارے کچھ جانبازوں سے آپکی چیخیں نکل گئ۔ | 0 |
|  | 2804 | ان قاتل کنجروں کا ایک ہی حل سب کو اکٹھے باندھ کر کراچی سے بیس میل دور سمندر میں پھینک دو | 0 |
|  | 1470 | تم اس کتیا کے بچے ہو جو دن رات ہیرا منڈی میں گزارتی ہے کیسی گشتی کے بچے | 1 |
|  | 97 | ضلع باجوڑ سے تعلق رکھنے والے نوجوان ناران کاغان سیر کیلئے جارہے تھے راستے میں کار حادثے کا شکار… | 0 |
|  | 3111 | حضرت داتا گنج بخش رحمتہ اللہ علیہ کا فرمان ہے عاجزی کے تکبر سے بچو، کیونکہ یہ نفس کا فریب ہے] | 0 |
|  | 700 | سپریم کورٹ کے منہ پر طمانچہ اسے کہتے ہیں کہ ایک نااہل شخص ڈی فیکٹو وزیراعظم کے فرائض سر انجام دے رہا ہے | 0 |
|  | 1880 | سال پہلے جب یہ اٹالین گورنمنٹ بنی تھی میں نے کوئ باجوہ غفور نہیں دیکھا تھا جو لکھتا | 0 |
|  | 3190 | سیالکوٹ جوتوں کا سیزن چل پڑا ہے اس سلسلے میں | 0 |
|  | 406 | اوئے کھوتی کے بچے تجھے کشمیر میں بسنے والی ہماری مائیں بہنیں نظر نہیں آتیں | 1 |
|  | 1381 | ہمارا شہر لوٹ رہا ہے کراچی بلاول زرداری بغیرت انسان اب تیری غیرت کہا ہیں کنجر انسان حرام خور کی گندی اولاد | 1 |
|  | 156 | لعنت اس کتے کے شکل پر | 1 |
|  | 2645 | مودی کشمیر میں مسلمانوں کا قتل عام کر رہا ہے جیسا کہ دوسری جنگ عظیم میں ہٹلر اور مسولینی نے یہودیوں کا قتل عام کیا | 0 |
|  | 718 | ان بےغیرتوں کو کسی بل میں گھس جانا چاہیے شرم سے ڈوب مرنے کا مقام ہے بیغیرتوں کے لئے | 1 |
|  | 1898 | پائن تسی منہ ذرا بند کرو میں اس کی طبیعت صاف کرتا ہوں | 0 |
|  | 2325 | ان کی طبیعت صاف کر دونگا حرامیوں کی | 1 |
|  | 2447 | مودی بھڑوا کتے اور حرامی جب بھی کشمیر میں ظلم کرے گا تیری بھارت ماتا کو تباہ کریں گے | 1 |
|  | 2207 | یہ بیٹی بیچ ہے مادرچود | 1 |
|  | 1101 | نہ صرف تربیت کا پتہ چلتا ہے بلکہ تخم کا بھی پتہ چلتا ہے | 0 |
|  | 443 | یار یہ انڈین کیا چاہتے ہیں۔۔؟ اپنی کی بہن کو عزت سمجهتے هيں اور کشمیر میں دوسروں کی بہنوں کی عزت لوٹ رہے ہیں۔ | 0 |
|  | 2982 | حالیہ بارشوں میں کراچی کے حالات دیکھ کر لگتا ہے کہ سارے حرام خور ڈھٹائی کی چادر اوڑھ کر بے غیرتی کی نیند سو رہے ہیں | 0 |
|  | 3448 | اوئے گانڈو کے جھریوں والے لوڑے، تو پہلے لکھنا سیکھ لے مادرچود، جُگت بعد میں | 1 |
|  | 900 | سور کی اولاد حرامی کتے دلے کمینے ذلیل لاشوں پر بھی سیاست کرتے ہوئے شرم نہیں آتی | 1 |
|  | 621 | عالمی عدالت انصاف نے بلوچستان میں کلبھوشن کی گرفتاری پر بھارت کو مجرم قرار دیا | 0 |
|  | 1714 | اسکی شیخ رشید نے گانڈ ماری ہوگی | 1 |
|  | 706 | تم نے صحیح کہا مہاجروں کے کراچی میں نمک حرام غیر مہاجر بھی شامل ہوگئے ہیں | 0 |
|  | 438 | تم کنجر لوگوں کا کام ہے بھونکنا کاٹنے کی ہمت نہیں ہے تم کنجروں میں | 0 |
|  | 1419 | کنجروں اور رنڈیوں | 1 |
|  | 282 | بلاول کے خاندان کے کسی بھی فرد نے تیری بہن نہیں چودی بس یہ غلطی ہو گئی | 1 |
|  | 703 | حامد میر خبیث کو الٹا لٹکانا چاہیے | 1 |
|  | 3168 | اس کنجھر خسرے کی سر عام روڈ پر گانڈ ماری چاہیے۔ بیغیرت باپ کا بیغیرت بیٹا بے غیرت ہوگا تیرا باب | 0 |
|  | 347 | شرم کرو اتنی ہمدردی ہے انڈیا کی | 0 |
|  | 3282 | گھٹیا لعنتی بندہ | 1 |
|  | 2276 | جو ڈنڈا تیری امی کو قطری نے دیا تھا اسکے بارے میں کیا خیال ہے مادرچود کتی نسل کے | 1 |
|  | 91 | اور خان کی حکومت میں پاکستان کان شا چاند پر پہنچ گیا ہے۔ لوگ مہنگائی ست زندہ درگور ہو رہے ہیں۔ | 0 |
|  | 3239 | تمہاری طبیعت صاف کرتی ہوں تیرے بچوں کا خیال نہ ہوتا تو کب کی تمہیں دفع دور کر چکی ہوتی | 0 |
|  | 591 | ہاہا فکر نا کر جانی بولے تو کتی چیز ہوں | 1 |
|  | 3067 | یہاں تو سب یکجہتی کے لیے اکٹھے ہوۓ | 1 |
|  | 787 | گندگی کی پیداوار سے بدبو کی ہی امید رکھی جا سکتی ہے۔ | 1 |
|  | 592 | گرین پاسپورٹ عبرت کا نشان بن گیا میکسیکو اور برازیل کی سیر کے لئے جانے والی پاکستانی خاتون | 0 |
|  | 2974 | انڈیا ھر بار لاھور مانگتا ھے حالانکہ بہن انہوں نے سیالکوٹ میں دی ھوئی ھے | 1 |
|  | 356 | دنیا کے زلیل ترین خبیث انسان تب انیل مسرت کے انڈیا کی ایسی حرکت کرنے کی جرت نہیں تھی | 1 |
|  | 3201 | ھمارے تمام پاپوش اینکرز مبارکباد کے مستحق ہیں ان کےپیروڈی ٹائپ تبصروں نے وہ کام کر دکھایا ہے جو اجتک نہ فوج کرسکی… | 0 |
|  | 773 | چوتیے تو تم ہو کسی کنجر کی نسل ، مادرچود بیچا تو تمہارے چور گنجے نے کشمیر کو | 1 |
|  | 1680 | ناندیڑ ٹریکٹرحادثہ کاشکار‘ڈارئیورسید قاسم سمیت دوافراد ہلاک | 0 |
|  | 3089 | رنڈی کے بچے یہ تیرا اور تیرے لندن والوں کا کام ہے تیری گانڈ ماری ہے | 0 |
|  | 291 | اس کھسرا اینڈ کمپنی کو بندہ بیچ چوراہے پہ کھڑا کر کہ گولی مار دے بیڑا غرق کر کہ رکھ دیا ہے | 1 |
|  | 2967 | بھارت کی سفاکیت پر عرب ممالک کی خاموشی مسلمانوں کے منہ پر طمانچہ ہے | 0 |
|  | 927 | کونسے ووٹ کی عزت؟ محترمہ سارے پاکستان کو کھوتا کھانے والے پٹواری سمجھ رکھا ھے؟ | 0 |
|  | 2808 | آپ کی بکواس کا ہمیں یقین نہیں ذرا دیکھیں آپ کے ابا جی کیا فرما رہے ہیں | 0 |
|  | 683 | پشتون قوم غیرت مند ہوتے ہیں انکے خون میں وفاداری ہوتی ہے یہ پشتون نہیں ہے اور اسکو انکے ساتھ ملانا منا… | 0 |
|  | 3283 | پاک فوج نے بھارتی چوکیوں کو نشانہ بنایا۔ ایک افسر سمیت ہندوستانی فوجی ہلاک | 0 |
|  | 1251 | حرامی پن کی کوئی حد اس پر تبصرہ کیا کریں، خود ہی دیکھ لیں انڈیا کا حرامی پن اور پاکستانی دنیا میں اوقات | 1 |
|  | 154 | لعنت ہے تم لوگوں کی خاموش بندوقوں اور توپوں پر۔ | 1 |
|  | 2210 | اوئے لونڈے کتے اپنی بکواس بند کرو حرامی | 1 |
|  | 234 | حرام کی کمائی سے حرامی پیدا ہوتے ہے اس کی مثال بلاول نے اچھے سے قائم کی باپ کی گرفتاری پر | 1 |
|  | 2384 | کہاں ہے وہ بہن چود بےغیرت ہیجڑا | 1 |
|  | 2231 | دو ٹھگوں کی جوڑی ایک ہجڑہ ایک بھگوڑی | 1 |
|  | 3065 | اقوام متحدہ میں بھارتی مستقل مندوب سید اکبرالدین تو جھوٹوں کا سردار نکلا | 0 |
|  | 1667 | کیسے کر لیتا ہے بہن چود کیسے | 1 |
|  | 1990 | انڈین کتوں کا بکواس کرنا سمجھ میں آتا ہے لیکن یہ پٹواری کیوں بھونک رہے ہیں؟؟؟؟ پٹواریوں اور لفافہ صح… | 0 |
|  | 789 | کشیدگی میں کمی؟؟ کیا بکواس کر رہے ہو بھائی | 0 |
|  | 2775 | ایک مظلوم ہی دوسرے مظلوم کا درد سمجھ سکتا ہے فلسطینی مسلمانوں کی کشمیری مسلمانوں کے حق میں ریلی | 0 |
|  | 2712 | یہ بھی ہو جائے گا۔ تُم اپنی چونچ بند رکھو | 1 |
|  | 1247 | لگتا ہے حرامی ہندووں تم اپنے باپ ٹیپو سلطان کو بھول چکے ہو جس کے آنے سے تمہاری ماوں کے بچے گر جایا کرتے تھے | 1 |
|  | 3412 | حضورؐ نے فرمایا جب بڑی جنگوں کا آغاز ہو گا تو اللہ تعالی عجمیوں سے ایک لشکر اٹھائے گا جو بہترہن سوار ہوں گے | 0 |
|  | 2025 | اپنی گند اٹھاؤ رنڈی کے بچے بہن چود بھڑوے | 1 |
|  | 3082 | اے دماغ سے پیدل ارسطو | 0 |
|  | 197 | عمران سرکار کا ایک سالمعیشت تباہ قرضوں کے انبار دوستوں نے منہ موڑ لئے | 0 |
|  | 2086 | قادیانی کافر مرتد لعنتی | 1 |
|  | 1827 | کوئی بتائے گا کہ ہمارے قمر درد والے شیر نے بکنے والے سینیٹرز کو نشان عبرت بنانے کا جو اعلان کیا تھا اسکا کیا بنا | 0 |
|  | 1886 | ظالم ہمیشہ صفحہ ہستی سے عبرت کا نشان بن کر مٹے ہیں، اور تاریخ ان پر تھو تھو کررہی ہے۔ انڈیا اور مودی کا اس ضمن بلند… | 0 |
|  | 164 | ان کی طبیعت صاف کر دونگا حرامیوں کی | 0 |
|  | 2704 | بڑے ہی کسی کنجری کی اولاد ہو مادر چود اربوں کھا گئ ہے پھر بھی ترس ارہا ہے یورپ میں ہوتی تو اب تک پھانسی ہو جاتی | 1 |
|  | 1266 | جج تو پھر بلیک میل ہو گیا | 0 |
|  | 2362 | یہ وہی حضرت نہیں جو پہلے لڑکیوں کا نام لے کر بکواس کرتے ہیں پھر رو رو کے پوچھتے ہیں۔۔ | 0 |
|  | 1481 | بے غیرت سے بھی گندا لفظ ہے تو وہ ان کے لئے استعمال کریں ۔۔لعنت بے شمار ان کنجروں پر | 1 |
|  | 2098 | یہ بیغیرت ہیں اور ہمیشہ رہے گے لیکن انکو چُننے والے بھی برے بیغیرت ہیں | 1 |
|  | 2825 | مادرچود دے بچے | 1 |
|  | 3059 | جرنیل تمہاری طرح کے جاہل اور کم ظرف نہیں ہوتے۔ انہیں صورتحال کی سنگینی اور حالات کی نزاکت کا اندازہ ہوتا ہے۔ | 0 |
|  | 1468 | لعنت اے ساڈھے تے بیغیرتوں اُٹھ جاؤ کی پاکستان دے مولوی وی مر گئے نے سب چور نے | 1 |
|  | 2450 | گھٹیا انسان بکواس کر کے لوگوں کو کیوں گمراہ کرتے ھو | 1 |
|  | 2921 | کون سےکشمیر کی آزادی جس کا سودا نیازی کتے نے امریکہ جا کر کیا | 1 |
|  | 2689 | یہ بھگوڑی بھی ہمارے پیسوں پر حج کرنے گٸ تھی لعنت | 1 |
|  | 2961 | تیری نسل پر لعنت ھے انڈین ایجنٹ ملک حالت جنگ میں تجھے لسانیت کی مرچیں کاٹ رھی ھیں | 1 |
|  | 2456 | پھر ان کنجروں کی بات کر | 1 |
|  | 2244 | کبھی سوچا تھا عمران خان مخالفین کا کیا حال کرے گا؟ | 0 |
|  | 1395 | جوس پینے کے بعد خالی ﮈبے کا پٹاخہ نہ بجائیں بلکہ قینچی سے کاٹ لیں تو قطرے مزید جوس حاصل ہو سکتا ہے | 0 |
|  | 1710 | بہن چود حرامی وارث میر کے ناپاک نطفے تجھے چوری نظر نہیں آتی نوابشاہ کی رنڈی نظر آتی ہے۔ | 1 |
|  | 2504 | پاک فوج کا ایل او سی پر بھارت کو کرارا جواب، افسر سمیت بھارتی فوجی ہلاک | 0 |
|  | 688 | سالہ نمک حرام نمک حرام گینڈے ٹویٹر نظر ڈالو پاکستانيوں نے تمہارا منہ کالا کردیا ہوا ہے | 0 |
|  | 1402 | جس کو اپنی ماں کی ننگی تصاویر دیکھ کے غیرت نا آئی اس دلا نا کہوں تو کیا کہوں | 1 |
|  | 2908 | آج رات بھارتی چوکی تباہ ہونے والی ہے پاکستان کے شاہین سے | 0 |
|  | 1073 | نکل بھوسڑی کے مادرچود گانڈو | 1 |
|  | 93 | بیغرت برگیڈ بھگوڑی | 1 |
|  | 1168 | کیسی نوکری ہے اس مادرچود کی عید کے دن بھی چھٹی نہیں ہے | 1 |
|  | 1187 | تیرا ابا خان تو بک گیا بیغیرتوں تم لوگ بہنوں کے سودے کرنے والے ہو کنجروں | 0 |
|  | 3423 | یہ حالت ہو گئی ہے ان کنجروں کی کہ عید نماز بھی اپنے گھر میں ہی پڑھ رہا ہے | 1 |
|  | 1705 | غزنی اور وردک میں فضائی حملوں اور تصادم کے دوران طالبان عسکریت پسندوں کو بھاری جانی نقصان اٹھانا پڑا۔ | 0 |
|  | 1337 | اس کی ماں کو رنڈی باپ کو حرامی اسکو ہیجڑا بے شرم بے حیاء بے غیرت نہ کہیں تو اور کیا کہیں؟ | 1 |
|  | 302 | بکواس ھے بکواس ہے یہ سب | 0 |
|  | 1066 | بہن چود ہر جگہ پر اپنے غلامی کا ثبوت دیتے ہوں | 1 |
|  | 873 | تیرا ابا خان تو بک گیا بیغیرتوں تم لوگ بہنوں کے سودے کرنے والے ہو کنجروں | 1 |
|  | 1851 | اوئے کھسرے آنا واپس پاکستان بے غیرت تو مرد نئ کھسرا ھے سالے ہجڑے | 1 |
|  | 345 | نسلی کتے کھسری کے بچے | 1 |
|  | 1121 | عمران سرکار کا ایک سال مکمل ہو گیا ہے او… | 0 |
|  | 2566 | ڈھٹائی بے غیرتی کی تمام حد پار کرلی ان کنجروں نے | 1 |
|  | 1986 | یے تم جیسے کنجروں کی وجا سے ھے اگر کشمیر کاسودا انریکا مے ن کرتے ابھی مودی گانڈ مارے گا | 1 |
|  | 3207 | خبردار اگر کسی نے آزاد کشمیر کی طرف دیکھا تو خون پی جاوں گی | 0 |
|  | 1069 | ایسی قوم جس کے پاس اب ندامت بھی نہیں ہے بیان بازیاں مزمتیں اور بس مرثیے | 0 |
|  | 488 | تیرے ناجائز سسر بھڑوے ڈالر نے یہ بکواس کیے تھے ارب ڈالر آئیں گے اگر باہر والا پیسہ پاکستان واپس لے آئیں۔ اس بیغ… | 0 |
|  | 2256 | افسوس ناک واقعہ ہے سیاست دان نہیں یہ سلیکٹڈ کے کام ہیں | 0 |
|  | 2677 | اوئے مریم کے گمشدہ کتورے ،اوہ بیغیرتوں کا اک پورا میگزین، شکل دیکھ اپنی آئینے | 1 |
|  | 2463 | نیازی نے جیل میں مریم کی عزت لوٹ لی پٹواریو اب یہ بیانیہ چلاؤ کشمیر والا تو وڑ گیا | 0 |
|  | 2538 | پاکستان کے جوہری ہتھیار اور مزائل پروگرام نے بھارت کو کس قدر خوف میں مبتلا کر رکھا ہے اس رپورٹ میں سنیں | 0 |
|  | 3276 | تیری ماں دا ٹھوکو خان کُتی ماں دا بچہ ویچ آیا ای کشمیر مادرچودو غدارو تم لوگوں گی گانڈ میں ایسا ڈنڈا | 1 |
|  | 1348 | اب تیری گانڈ مارنے کا ٹائم آنے والا ہے تھوڑا انتظار کرو کنجروں کی ترجمانی کرنے والی گشتی | 0 |
|  | 3055 | وطن فروش | 1 |
|  | 2391 | اگر سندھ حکومت کو ٹیکس نا دیں تو کیا وسیم اختر کو دیں ؟؟؟ جھوٹ بولتے ہیں یہ، حرام کھاتے ہیں | 0 |
|  | 2329 | لٹکاؤ اس مادرچود کو | 1 |
|  | 989 | عجیب گندی عورت ہو تم بے شرم عورت اس میں عمران خان کا قصور کہاں | 1 |
|  | 2964 | بے شرم اسی بکواس کی کمائی کھاتے ہیں سانحہ ساہیوال اس کے باپ کے دور میں ہوا تھا؟ | 0 |
|  | 1634 | بے گناہ زندانوں میں قید اسیران جہموریت کو عید مبارک اللہ پاک بے گناہوں کو قید کرنے والو کو نشان عبرت بنادے | 0 |
|  | 2900 | اوئے دلیا اوئے کتیا اوئے خنزیرا اوئے سئورا او تیری ماں نوں لن | 1 |
|  | 2016 | قادیانی دنیا کا بدترين کافر ہے اور قادیانیوں کو کافر نہ ماننے والے بھی کافر ہے | 0 |
|  | 3257 | اج ایک مرتبہ پہر کوٸٹہ کے نواحی کچلاک میں نماز جمعہ کے دوران بم بلاسٹ ہوا | 0 |
|  | 775 | اور بھائی یہ ملک تو بنا ہی فوج کےلیے ہے ان جرنیلوں کے اگر عوام عللے تللے دیکھ لے تو ان کو… | 0 |
|  | 1686 | ایک کہانی ہے کہتے ہے کہ ایک کسان سے کتا روٹی بھگا کے لے گیا کسان کتے کے پیچھے خوب بھاگا جب تھک کیا تو بیٹ کر کہنے… | 0 |
|  | 730 | جو دھندے تم سندھی بھڑوے کرتے ھو وہی بیان کر رھے ھو گانڈو | 1 |
|  | 139 | سندھ کے عوام کو لوٹنے والے عبرت کا نشان بننے کے قریب۔ جعلی اکاؤنٹس کیس کے مرکزی کردار دبئی کے شہر ناصر عبداللہ لوتھ… | 0 |
|  | 1637 | پھودی | 1 |
|  | 3263 | جس نبی کا سنتے آئے دُشمن کی موت پر بھی | 0 |
|  | 2910 | لن دی بکواس ھے | 1 |
|  | 1240 | یہ کمینے انڈیا کو سبق سکھا رھے ہیں کرتوت دیکھو ان لعنتیوں کے | 1 |
|  | 1765 | کنجری اپنے پیدا کرنے والےمہا کنجروں کے ساتھ | 1 |
|  | 380 | ہجڑے کو ہجڑا نہ کہوں تو کیا کہوں۔ حرام کی اولاد کو حرامی نہ کہوں تو کیا | 1 |
|  | 2606 | اور ان پر ظلم کرنے والوں کو عبرت کا نشان بنا دے | 0 |
|  | 3383 | مراد سعید تو حرامی ہے اور تیرا کتا باپ عمرو نیازی مادرچود ہے تم دونوں گانڈو واشل ہو انڈیا نے تم سب | 1 |
|  | 2084 | اس کی بولتی بند ھے آج کل کیونکہ اس کے اوپر سے ابھی تک ہولی کا رنگ نہیں اترا ہندو کی بچی | 1 |
|  | 2153 | مصیبتیں ہم کو بیدار کرنے کیلئے آتی ہیں، پریشان کرنے کیلئے نہیں جب تک غموں کا اندھیرا نہیں ہوگا خوشیوں کے ستارے نہیں چمکیں گے | 0 |
|  | 1642 | جنرل باجوہ کو سال کی ایکسٹینشن کیا ملی ؟ کتوں نے آسمان سر پر اٹھا لیا۔ | 0 |
|  | 1467 | ایسا ہی ہوتا ہے اور جب ان کسی کی دل اراضی کرتا ہے ہے تو پر مقافات عمل ہوتا ہے اب سنے برداشت کریں | 0 |
|  | 531 | ارے مادرچود مرضی سے کون جاتا ہے جیل | 1 |
|  | 1895 | تیری ماں کے پھودے پے چڑھا ہے خان دلال عورت۔۔۔ بہن چود میرے سامنے ہوتی تو تیری چوت میں لوڑا پیل دینا تھا مادر چود | 1 |
|  | 828 | سوشل میڈیا پہ پاکستانی قوم نے بھارت کو شکست دی ساری پاکستانی قوم نے کشمیر زندگی اور موت کا مسئلہ بنارکھا | 0 |
|  | 2035 | پاک فوج نے بھارتی فوج کے افسر سمیت فوجی جہنم واصل کردیے، بنکر بھی تباہ کر دیے | 0 |
|  | 28 | پیارے پاکستانیوں قمر جاوید اور جیسے حرامزداوں کے اخباری بیانات اور ٹویٹوں پر تالیاں | 1 |
|  | 3139 | ﴿بى گمان کسانی که کلام الله را تلاوت می کنند و نماز را بر پا می دارند، و از آنچه روزی شان داده ایم؛ پنهان و آشکار… | 0 |
|  | 2493 | اگر آرمی چیف باجوا صاحب ریٹائرڈ ہوتا تو انہوں نے بکواس کرنی تھی | 0 |
|  | 2819 | یوں تو دنیا میں سبھی آئے ہیں مرنے کے لیے | 0 |
|  | 2040 | میرا کشمیر واپس کر حرامزادے | 0 |
|  | 1678 | پورا شہر تباہ ہو چکا۔ میں سیاست سے بالا تر ہو کر بات کر رہا ہوں ۔پورے ملک کے صحافیوں سے درخواست ہے کہ وہ کراچی کا د… | 0 |
|  | 2890 | آج دنیا نیوز کے شو ٹو دی پواٸنٹ میں کامران شاہد پان والا نے سٹوڈیو میں ایک انڈین کتے کو مدعو کر رکھا تھا جوپاکستان پر بھونک رہا تھا | 1 |
|  | 2835 | کیسے کر لیتا ہے بہن چود کیسے | 1 |
|  | 3192 | اور پھر خان نے بحثیت مسلمان یہ کہ کر اس کی بکواس کو بکواس ہی رہنے دیا۔ | 0 |
|  | 2533 | پشاور سے لے کر کراچی تک لوگ سراپاِ احتجاج ہیں ہاتھ اٹھائے جھولی اُٹھائے یکجا ہیں منگو چھوڑ دے ہماری جان غلطی ہو گئی اِن سے معاف کر دو | 0 |
|  | 1471 | بکواس بند کرو، چند لوگوں کی وجہ سے اس ملک کا ستیاناس کر دیا ہے، اتنے افسروں میں سے کوئی ایسا نہیں جو چیف بن سکے | 0 |
|  | 2802 | یہ مادرچود بڑا فیمینسٹ نہیں بڑا مادرچود ہے | 1 |
|  | 1940 | تیری ماں کا ننگا ناگن ڈانس کرواں بھین چود گانڈو تیرے بہنوئی ہیں | 1 |
|  | 2710 | بچپن میں ایک کہاوت نانا جان سنایا کرتے تھے که کتے بھونک بھونک کر آسمان سر پر اٹھا لیتے ہیں مسافر اپنی منزل پر پنچ جاتے ہیں | 0 |
|  | 222 | بے غیرتو چور ڈاکو تمھاری اولادیں بھی ان کنجروں کی غلام مریں گی | 1 |
|  | 2228 | به چندسال پیش که نگاه می کنم می گم چه قدر تباه بودم یعنی چند سال دیگه هم همین حسو درمورد الان دارم؟ | 0 |
|  | 3155 | الجہاد الجہاد لبیک لبیک کشمیر بنے گا پاکستان انڈیا کو اور مودی کو یہ پیغ… | 0 |
|  | 1796 | ملت کا خسارہ ہیں یہ اور انکو نشان عبرت بنا دیں اب | 0 |
|  | 3149 | پی ٹی آئی والوں خان نے کشمیر کو بھولا دیا مگر یہ کتا بھونک بھونک کر ےوجہ دلا رہا ہے اس کی حوصلہ افزائی تو بنتی ہے۔ | 1 |
|  | 941 | بکواس بند کر | 0 |
|  | 737 | زمانہ امن میں ہم ہر وقت تیار ہے دشمن کے دانت کھٹے کرنے کلیے زمانہ جنگ میں جنگ کسی مسئلے کا حل نہی ہے | 0 |
|  | 495 | پنڈی کا شیطان بےغیرت انس… | 1 |
|  | 633 | افسوس ناک واقعہ ۔اللہ پاک مسلمانوں میں اتحاد پیدا کرے۔ | 0 |
|  | 1672 | مراد ہم تیرا گانڈ مارے گا سرسوں کا تیلُ لگا کر | 1 |
|  | 3043 | باندھی جی مختلف گوٹن م سنوکر گیم ء جوا سرعام ھلن لگی باندھی پولیس نند ستل نوجوان نسل تباھ ایس پی کان سنوکر گیم بند کرائن جو مطالبو | 0 |
|  | 2370 | آفغانستان میں امریکن فوجی احتجاج کر رہا ہے کہ | 0 |
|  | 1245 | دنیا کا کنجر دلا غدار چور ہرام خور نواز گنجا ہے | 1 |
|  | 221 | میری ہمتیں ابھی جھکی نہیں میرے حوصلے ابھی بلند ہیں مجھے ہار جیت سے غرض نہیں… | 0 |
|  | 2759 | اس انڈین کی ویڈیو دیکھ کر آپ کا دل چاہے گا کہ ہم اپنے یوٹیوبرز کو گولی مار دے وہ بھی ان کی گانڈ میں۔۔۔ | 1 |
|  | 649 | سوات سے تعلق رکھنے والی نسیمہ اختر پر سیکورٹی فورسز کا تشدد اور اب گرفتار کرکے جیل منتقل کرنا شرمناک ہے | 0 |
|  | 265 | کون لوگ ھو تُسی دماغوں میں بھوسا بھرا ھویا ہے کیا جو فالتو کی بکواس کرتے پھرتے ھو | 0 |
|  | 2186 | ایک منجے ہوے سیاست کو عبرت کا نشان… | 0 |
|  | 480 | یہ مادرچود خنزیر کے تخم سے ہیں | 1 |
|  | 2085 | تمام اھل وطن کو عیدالاضحی مبارک | 0 |
|  | 1960 | بالکل ایسے ہی ہے ان نکموں نے تو بھارت کو کہیں کا نہیں چھوڑا | 0 |
|  | 3085 | جہاد سے کوئ انکار نہیں کرتا لیکن اگر زندہ بچ گئے تو بعد میں لاپتہ ہونے سے ڈر لگتا ہے | 0 |
|  | 2607 | اور تیری ماں کی کروں جو چوک میں چُودواتی ہے؟ | 1 |
|  | 3020 | تیری بہن کو تو ابھی بھی ہندو ہی چود رہے ہیں تمہاری ماں تو ہندوں لن ہی پسند کرتی ھے تبھی نیازی نے باڈر ک | 1 |
|  | 2981 | اللہ تعالی آپ کو اور ہمت دے پاکستان کے دشمنوں کو منہ توڑ جواب دینے کے لیے اور اللہ آپ کو اپنی امان میں رکھے | 0 |
|  | 716 | ھیرا منڈی میں کپڑے اتار نے والی اینکر کھال اتار نے کی بات کر رھی ھے طوائف کی بچی | 0 |
|  | 1482 | ریہام خان تیری گانڈ ایسی ماری عمران خان نے کہ گشتوڑی تجھے رے رے کر درد اٹھتا ہے اور یہ درد ہی ہمیں مزہ دیتا ہے | 0 |
|  | 2650 | ککڑ اب اپ کی بک بک کوئی نھی سنتا اور نہ کوئی پڑھتا ھے مزید اب اپ بکواس کرنا بند کر دو | 1 |
|  | 1083 | جھوٹے مکار نام نہاد بےغیرت بےشرم صحافی | 1 |
|  | 175 | لاہور بھارت کی آبی جارحیت سےہزاروں ایکڑپرکھڑی فصلیں تباہ ہوگئی ہیں،سراج الحق | 0 |
|  | 3129 | انڈیا کی کٸ چوکیاں تباہ ، کتے جہنم واصل | 0 |
|  | 2811 | میری گزارش ہے ان یزید کی اولاد کو منہ توڑ جواب دینے کیلئے ایک ٹرینڈ چلانا چاہئے | 0 |
|  | 3295 | حرام زادے بڈھے تیری بیوی اور بیٹیوں کو اٹھا کر کشمیر کے پہاڑوں میں لے جائیں گے بہن چود | 1 |
|  | 2485 | حکمرانوں ،مجاز افسران اورعدلیہ کے سوچنے کا مقام ہے ۔ | 0 |
|  | 3384 | اس ک کے کہنے کا مطلب ہے یہ کہ پاکستان میں روزانہ دھماکے ہوتے رہے ہیں | 1 |
|  | 3039 | بکواس کرتا ہے یہ سیرس لنے کی ضرورت نہیں اس کو | 0 |
|  | 877 | پاک فوج کی جوابی کارواہی میں ایک افسر سمیت بھارتی کتے شمشان گھاٹ روانہ… | 0 |
|  | 49 | یہ ملک کے پاکستان کی عوام اتنی پاگل نہیں ہے کہ کسی کو سمجھ نہ سکے ایک نا ایک اکیس پوری عوام | 0 |
|  | 1353 | لگتا ہے سفینا نے اس عمر میں شیخ رشید کے لوڑے کے چوپے لگائے اور کھڑا نا کر پانے کی خفت میں سزا ملی | 1 |
|  | 2358 | اپوزیشن کا صرف ایک ہی اعلاج ہے ان کی گانڈ میں گولیاں ماری جائیں یہ مادرچود کے بچے اس ملک کا بھلا کبھی نہیں چاہینگے | 1 |
|  | 397 | گانڈو ضمیر فروش تمہارے ابو نے پارلیمنٹ میں بتا دیا ہے ہم انڈیا کے اندرونی معاملات میں مداخلت نہیں کر سکتے | 1 |
|  | 1620 | گل بخاری، تقی، حقانی کالا سور سلمان حیدر اور تیری گانڈ پاکستانی مارے تو بیرونی مداخلت، اور رنڈی کے بچے نواز پشتین نے مودی کو اپنے گھر بغیر ویزہ تیری بہن کے بلاتکار کے لئیے بلایا تھا ؟؟ | 1 |
|  | 2415 | اور زرداری کی ڈیش اینٹ سے بجای گی | 1 |
|  | 2343 | یہ دھرتی تجھ پر لعنت بھیجتی ہے | 0 |
|  | 829 | شورِ تکبیر سے تھرتھراتی زمین جہاد کا نعرہ ایسے لگائیں ساری زمین نہیں پورا کفر ہلے انڈیا کو اور مودی کو یہ پیغام دین… | 0 |
|  | 2627 | یہ بدبخت درندہ انسانوں میں رہنے کے قابل ہی نہیں ہے | 1 |
|  | 3012 | یہ ملک ہے یا کنجر خانہ ہر روز نیا ٹیکس نیا بھتہ اور نیازی حرامی کیوں لوگوں کا کاروبار چھین… | 1 |
|  | 3489 | او کسی گٹر کی پیداوار گشتی امریکہ میں تیرا یار کشمیر کا سودا کر کے آیا ہے ،یہ سب کچھ دنوں میں قوم کے | 1 |
|  | 2232 | منحوس انسان جب سے توں ریلوے کا وزیر بنا ہے رب سے ہی ریلوے کا برا حال ہے | 1 |
|  | 2365 | کشمیر مظالم | 0 |
|  | 2133 | ہمارے حکمرانوں سے بہتر تو حجاج بن یوسف جیسا فاسق و سفاک اعظم تھاااا۔۔۔مانا کہ وہ لعین ہوااا۔۔۔لیکن اس کی غیرت | 0 |
|  | 3364 | ھاھاھا عامر بھائی آپ نے تو بولتی بند کردی بچے کی | 0 |
|  | 2633 | نواز شریف نے کشمیریوں کا ساتھ نہ دیا تو زلیل و رسوا ہو رہا ہے | 0 |
|  | 968 | میرا اکاونٹ سسپینڈ کر دیا گیا تھا | 0 |
|  | 319 | ایسے نہیں ایسے سن زراہ غور سے حرامی | 1 |
|  | 1711 | تم بھی صحافی بننے کا خواب دیکھتے دیکھتے حرامی بن گۓ ہو | 0 |
|  | 2758 | او تیرے پیو خادم لنگڑے دی ماں نوں لن | 1 |
|  | 1108 | اللہ کرے اسے جلد ہی کتے کی موت نصیب ہو اور انڈیا کے کم از کم ٹکڑے ہوں | 0 |
|  | 2924 | کاہنات کا سب سے ظالم عبرت کا نشان بن گیا تو انشااللہ مودی بھی عبرت کی مثال بنے گا | 0 |
|  | 1325 | بیشرم آدمی کچھ نہیں تو اپنے سفید بالوں کا ہی لحاظ کرو ۔ پاکستان کو نوچ کر کھا گئے پھر بکواس کرتے ہو | 0 |
|  | 712 | افسوس ناک مقام ایک مسلمان عورت نے قرآن مجید کو جلا رہی ہے ہندو کرا سمجھ میں آتی ہے لکن مسلمان کر راہیے ایسے | 0 |
|  | 2789 | پٹواری ایسی گندی نسل ہے | 0 |
|  | 89 | پاکستان کا میڈیا کہتا ہے کہ ہم عید سادگی کے ساتھ منائیں گے لیکن اتنی خاموشی اختیار کر لیا آفسوس ناک ہے | 0 |
|  | 1633 | جب سندھ کا پیسہ کھا رہی تھی۔۔بلاول ھاوس میں بھتہ لے رھی تھی۔۔جب عزیر بلوچ کے ھاتھوں کئی لوگوں کا مڈر کروایا | 1 |
|  | 2247 | سر ہمیں پتا ہے انڈین میڈیا ایک نمبر کے دھوکے باز اور بکواس نیوز چلاتے ہیں لومڑی میڈیا انڈین میڈیا | 0 |
|  | 214 | مریم کی گانڈ آج رات خان مارے گا پھر توں ماموں بنے گا اور ناچے گا | 0 |
|  | 2848 | یہ انصافیے یوتھیے کس منہ سے سوال کرتے ہیں کہ نواز شریف نے پچھلے سال میں کیا کیا تم لوگ جواب دو تم نے کیا کیا بیغ… | 0 |
|  | 3428 | کُتے پخائے ہوئے ہیں پاکستانی ٹوئیپس نے | 1 |
|  | 3411 | آج یہ قصائی کروڑ پاکستانیوں کی کھال اتار رہا ہے | 0 |
|  | 3437 | یہ وہی عورتیں ہیں جو سال کی بچیوں کو اپنے گھر پر ملازمہ رکھتی ہیں | 0 |
|  | 2275 | پنڈی کی ٹاپ کی رنڈی سے شادی کی ہے مادرچود کشمیر کا اور سینٹ کے معاملے کا آپس میں کیا تعلق | 1 |
|  | 2631 | کبھی کبھی ہم رشتوں میں لحاظ رکھتے ہوئے بہت سارا زہر اپنے اندر اتار لیتے ہیں | 0 |
|  | 3211 | پاکستان نے ابھی نندن کی پہلے جو طبیعت صاف کی اس پر تو اسے فوجی اعزاز ویر چکرا مل گیا | 0 |
|  | 246 | بھارتی فوجی رونے لگ گئے، کہتے ہیں ہم ہزار روپے تنخواہ والے کیوں بارڈر پر جا کر مریں پاکستان زندہ باد | 0 |
|  | 1692 | مجھے سب پتا ہے یہ گھٹیا مخلوق کبھی نہیں سمجھے گی | 0 |
|  | 2336 | کاش تیرے باپ نے تجھے حلال کھلایا ھوتا تو شیطان کا ساتھی نہ بنا ھوتا یہ تھی تیری تبدیلی خبیث انسان | 1 |
|  | 2690 | ابھے او جعلی آرسطو | 1 |
|  | 2405 | تیری بہن کو چود رہا ہوں ابھی پھر تیرے ابے کی گانڈ مار کے تیری ماں کو چودوں گا | 1 |
|  | 2932 | مئیر کراچی اپنا رونا گانا کرتے لگے ہاتھوں علی زیدی کو بھی طمانچہ رسید گئے | 0 |
|  | 1200 | کیوں ناصر بٹ سے پوچھے یا مریم سے پوچھے پریس کانفرنس میں جو جو موجود تھا ان سب کتوں کو جیل میں ڈالو اور فٹ کا ڈنڈا… | 0 |
|  | 1474 | ہجڑے پہ لعنت | 1 |
|  | 3158 | بلاول بیغیرت زرداری چور | 1 |
|  | 614 | یہ سب اپنے پاس سے بکواس کرتے ہیں۔ | 0 |
|  | 1422 | سب سے پہلے تو اس حرامزادے اور اس کی بدچلن بیٹی کے غریب عوام کا لوٹا ہوا پیسہ نکلوائیں | 1 |
|  | 1466 | ھر کمنٹ میں تمھیں اتنی لعنتیں اور گالیاں پڑتی ھیں کہ آپ کا نام ھونا چاھیئے تھا زندہ درگور آیا۔ | 0 |
|  | 2581 | یااللہ اس افغانی ملعونہ کو اور اس کی دلالی کرنے والوں کو نشان عبرت بنا دیں | 0 |
|  | 1826 | اقوام متحدہ سلامتی کونسل کا ہنگامی اجلاس طلب یہ خبر اس بیغیرت نسل کے منہ پر طمانچہ ہے جو کہتے تھے عمران خان نے کشمیر بھیج دیا | 0 |
|  | 123 | پر مرے ہوئے کے نام پر دیگیں دے دیتے ہیں | 0 |
|  | 1409 | نا صرف کشمیر آزاد ہوگا بلکہ بھارت کشمیر میں ہر گرتی خون اور آنسو کے قطرے کا حساب دیگا انشاءاللّہ… | 0 |
|  | 846 | باقی تم پر اتنی امید نہیں تھی کہ تم ایسا بھی کرو گے۔ | 0 |
|  | 3096 | ضلع نگر میں نگر خاص کے مقام پر سابق شوہر نے اپنی سابق بیوی اور اسکے شوہر پر فائر کھول دیا ۔ کوئی جانی نقصان نہیں ۔ پولیس | 0 |
|  | 1995 | کون اے تو بکواس نا کر | 0 |
|  | 262 | اُلو کے پٹھے یہ تیرے کرنے کے کام نہیں ہیں | 1 |
|  | 2114 | اپنی اس بھگوڑی سے کہو کہ اکاؤنٹ میں اتنا پیسہ کہاں سے آیا، کیس ختم۔ | 0 |
|  | 970 | گانڈ ماراو مادرچود | 1 |
|  | 2064 | حیرانی ہوتی ہے حامد میر جیسے بےغیرت صحافیوں پر۔۔۔ بکواس کرتے ہیں کہ میڈیا کو باندھا جا… | 1 |
|  | 3258 | دھرنے میں گھنگھرو بندھوَا کر خرمستیاں کرتی باجیاں نچوانے والے وائٹ کالر کنجروں کے علاوہ سب دلال ہی تو ہیں | 1 |
|  | 3237 | ماں کی دلالی کرنے والے مادر چود | 1 |
|  | 2027 | آپ کی طرف سے اور نیب fia کی طرف سے انتہائی سست کاروائی اس طرح تو اگلے سال میں بھی کچھ نہیں ہونے وا… | 0 |
|  | 2008 | میرے وطن یہ عقیدتیں اور پیار تجھ پہ نثار کر دوں محبتوں کے یہ سلسلے بےشمارتجھ پہ نثارکردوں میرے وطن میرےبس میں ہو | 0 |
|  | 1494 | اب سمجھ آئی گٹر کو گٹر ھی ریٹویٹ کیوں کرتا ہے مادرچود | 1 |
|  | 931 | تم دونوں کی بکواس ہم روز سنتے ہیں | 0 |
|  | 1078 | جیسے تمہاری ماں بہن مشرف نے چودا وہ تم لوگ زندگی بھر یاد رکھا گے | 1 |
|  | 2801 | نیازی گھٹیا انسان کشمیر کا سودا کر کے منہ کالا کروا چکا ھے | 0 |
|  | 1274 | یوتھیے اپنی اٹھا اٹھا کر کہتے تھے کہ عمران کھسرا خوشخبری دے گا کنجروں او بولو تے سہی | 1 |
|  | 459 | جب کسی کو ایک بار اگنور کریں تو بکواس کرتا ہے جب زیادہ اگنور کرو تو زیادہ بکواس کرتا ہے | 0 |
|  | 2481 | تیری ماں کو چودا ہے لیگیوں نے گشتی علیمہ رانڈ کا بھائی کتے کا بچہ عمران نیازی مادرچود نے کشمیر بیچ دیا | 1 |
|  | 3210 | اے میرے قایئد، جس ملک کے حصول کیلیۓ آپ نے اپنی جوانی اپنی صحت ضایئع کر دی، اپنا دن کا چین راتوں کی نیند حرام کر دی،… | 0 |
|  | 766 | حق کی بات کرنے والوں کوقید جیل مقدمات جلا وطنی سزاؤں عمر قید سے ڈرایا جاتا ھے ہم نے تو سیاست شروع ہی تب کی ھے | 0 |
|  | 990 | گندے انڈے۔ ایوب دور میں پاکستان ایشیا میں سب سے زیادہ ترقی کرنے والا ملک تھا۔ | 1 |
|  | 3161 | کسی گشتی کے بچے حرام زادے باپ کی اولاد حرامی اولاد جب تم لوگ اپنے باپ کت نہیں تو مادر چود بھڑوے حرامی پاکستان کے | 1 |
|  | 781 | عزت کا ایک سال ذلت کے پچھلے چالیس سال سے بہتر ہے | 0 |
|  | 254 | ہے جب بھی چوری کرنا اربوں کی کرنا کبھی روٹی کے لیے چوری نا کرنا ورنہ بے موت مارے جاوُ گے۔ | 0 |
|  | 902 | کون تیری ماں کا ٹھوکو تھا جس نے کشمیری خریت قیادت سے ملنے سے انکار کیا ؟؟ | 1 |
|  | 460 | بہت دلا انسان ھی تو یقین مان۔۔ کشمیر پے سیاست کرنے سے باز نھی آ رھے۔ بے شرم | 1 |
|  | 698 | دو مادرچود لاہور کے جنہوں نے رابعہ خان نامی لڑکی کی عزت لوٹی | 1 |
|  | 202 | تیرا باپ مادرچود بیچ کے آیا ہے حرام کے پِلّے | 1 |
|  | 1429 | خانصاب نے ثابت کیا کہ ان کا ٹیمپریمنٹ ٹیسٹ پلئیرز والا ہے ورنہ کون اتنا تھوک چاٹ اور بوٹ چاٹ سکتا تھا | 0 |
|  | 176 | مادرچود دو دودن ٹرینڈ میں ذلیل ہونے کے بعد آج اپنے باپ نواز شریف کی آپشن شامل ہی نہیں کی | 1 |
|  | 1581 | دن رات ایک کر کے نمک حلالی کی کوشش تو کر رہے ہیں | 0 |
|  | 2530 | یے تم جیسے کنجروں کی وجا سے ھے اگر کشمیر کاسودا انریکا مے ن کرتے ابھی مودی گانڈ مارے گا | 1 |
|  | 2081 | تیری بیوی اسی سپلائی کا حصّہ تھی، منگوایا تو نے لونڈا تھا بُنڈ مروانے کے لیے، لیکن ارشاد نے عورت بھیج دی | 1 |
|  | 507 | تمہاری بکواس نے سمجھا دیا ھے کے تم کسی گندی نسل کے ھو بتانے کی ضرورت نہیں حرام کی اولاد | 0 |
|  | 124 | بھٹو کنجر شرابی بینظیر دلی کرپٹ عورت زرداری اپنی ماں کا یار کھسرا بلاول ایک سے ایک بڑھ کر کرپٹ مادر | 1 |
|  | 1580 | تم بھی صحافی بننے کا خواب دیکھتے دیکھتے حرامی بن گۓ ہو | 1 |
|  | 1532 | یہ ہے ان بیغیرت لوگوں کا کنجر پنا نا یہ خود کام کرینگے نا ہی کسی کو کام کرنے دینگے | 1 |
|  | 1802 | یہ مادرچود دَلہ اپنے ناجائز باپوں کو اپ ڈیٹ دے رہا ہے | 1 |
|  | 1882 | تم نے ثابت کیا ہے کہ خان سے اپنی گانڈ مروا مروا کے تمہاری مت ہی ماری گئی ہے | 1 |
|  | 2969 | کشمیر بنے گا پاکستان زلیل ہو گا ہندوستان | 0 |
|  | 3368 | افغان صوبے غزنی میں سیکیورٹی آپریشن میں طالبان کا ایک ریڈ یونٹ کمانڈر ہلاک ۔ | 0 |
|  | 2290 | سر شاہ غلام قادر کا ن لیگ سے کوئی تعلق ہی نہیں یہ تو بس مفاد پرست انسان ہے شہ غلام قادر نمک حرام ہے | 0 |
|  | 51 | جب تک غیرت تھی تو ایک بہن کے خط پر محمد بن قاسم لشکر لے کر سندھ آ گیا جب بے غیرت ہوئے تو آپنی ہی بیٹی بیچ دی لکھ دی لعنت تیرے جمن تے | 1 |
|  | 180 | جیسی شکل ویسی بکواس | 1 |
|  | 1593 | جس پر احسان کرو اس کے شر سے بچو ۔۔۔۔۔ پوچھنا یہ تھا کہ ایکسٹینشن دینا بھی احسان میں شامل ہوتا ہے کہ نہیں | 0 |
|  | 3288 | اللہ کی لاٹھی تو تم بےغیرتوں پر پڑنی چاہیۓ۔ جو اتنی دور امریکہ تک گۓ صرف کشمیر حوالے کرنے۔ | 0 |
|  | 772 | موت اس کی ہے کرے جس کا زمانہ افسوس یوں تو دنیا میں سبھی آئے ہیں مرنے کے لیے | 0 |
|  | 1212 | جس مادرچود کو پیدا کرنے کے بعد ماں چاٹنا تک پسند نہیں کرتی | 1 |
|  | 2023 | لکھ لعنت تیری شکل پہ ، کسی بھگوڑی ماں کی بچی جب اور کوئی بس نہیں چل سکا چوروں کو آزاد کرانے پر لگے ہو | 1 |
|  | 79 | یہ گندہ انڈہ قلم فروش کیا ثابت کرنے کی کوشش کر رہا ہے۔ | 1 |
|  | 2264 | یہ خبیث انسان جن کا ایجنٹٹ ھے انکی ہی زبان بولے گانا | 1 |
|  | 1324 | بھارت دنیا کا سب سے بڑا دہشتگرد اور اقلیتوں کے لیے غیر محفوظ ملک جس کا منہ بولتا ثبوت کشمیر میں کرفیو ہے | 0 |
|  | 1793 | بھارت نے سالوں میں اتنی ہمت نہیں کی کہ کشمیر پر قابض ہو جاۓ اور پاکستان کو آنکھیں دکھاۓ اور اس طرح کی باتیں کرے… | 0 |
|  | 1448 | پاک فوج کا دشمن کو ایک بار پھر منہ توڑ جواب، | 0 |
|  | 1930 | جس قوم کے حکمران بے غیرت اور بزدل ہو جاٸیں ، اس قوم کی بیٹیاں اسی طرح زلیل و رسوا ہوتی ہیں | 0 |
|  | 2526 | پیارے بچو آج کے دن برصغیر کا مسلمان آزاد نہیں صرف تقسیم ہؤا تھا، | 0 |
|  | 237 | اس کتے کے پلے کو کیوں گورنمنٹ نے اتنی ڈھیل دے رکھی ہے جب ،جس پہ جی چاہے بھونکتا رہتا ہے حرامزاده | 1 |
|  | 2248 | پورےعلاقےمیں دہشت پھیلانےکیلئےبھارتی فوج نےشیطان کو بھی شرما دیا | 0 |
|  | 456 | مریم نواز کا ظالم طاقتوں کو للکارنا ،انصاف کی جنگ ظلم کے سائے میں لڑنا،عوام کی عزت کا مطالبہ کرنا،نواز شریف کے علم… | 0 |
|  | 545 | اب انڈیا کی تین سال اور ماری جائے گی یہ ہوتی ہے جرات ایک اکیلی عورت پورے بھارت کے سامنے ڈٹ گئی ہے اسے موت کا کوئی خوف نہیں ہے | 0 |
|  | 3079 | تُم جیسے بیغیرتوں ، مادر چودوں اور دو ٹکوں کے لۓ مائیں بہنیں بیٹیاں اور بیویاں بیچن… | 1 |
|  | 2235 | میجر تمھارا باپ جنرل عطاء حسنین کیا کہہ رہا ہے یہ سن لیتے بکواس کرنے سے پہلے | 0 |
|  | 3400 | کس قدز بکواس کرتا ہے یه بنده ارسطو صاب آپ نے ہی عوام کو ٹرک کی بتی پیچهے لگایا ہوا تها یہ سب کہہ کر دراصل تم اپنے م… | 0 |
|  | 3242 | تیری ماں تیری بہن کے یار ہیں انڈیا والے | 1 |
|  | 2099 | آج صبح ڈنڈو شاہ منڈا میں سانحہ گوسم کے مقتولین کے گھر حاضری تعزیت کی اور واقعے کی تفصیلات لیں | 0 |
|  | 663 | عمران خان کی جاری سپیچ ان لوگوں کے منہ پر ایک طمانچہ ہے جو کہتے ہیں عمران احمد خان نیازی یہودی ایجنٹ ہے | 0 |
|  | 3463 | احسن صاحب صبر کریں کچھ ناشکرے لوگوں کو یہی سب چاھیئے تھا جو اس وقت مل رہا ھے زلیل ھونے دیں ان لوگوں کو | 0 |
|  | 739 | ابھے گانڈو یہی پرابلم ہے میرے ملک میں جسکے پاس چار پیسے آ گئے حرام یا حلال کے وہ خود کو قانونسے بالا سمجھتا ہے | 1 |
|  | 502 | بلدیاتی اداروں کا کام ایک وفاقی وزیر کروانے لگا۔سارا میڈیا اس ڈرامے پر ڈیبیٹ کررہاھے۔ یہ کیا جانیں… | 0 |
|  | 2949 | شرم آنی چاہیے ان حرامیوں کو یہ بکواس کرنے سے پہلے جھوٹے سالے | 1 |
|  | 994 | وہاں تیری ماں سیتا وائیٹ کو بھی چودتا رہا ہے بہن چُود انسان کُتی کے بچے شرم نہیں آتی | 1 |
|  | 201 | کتی کے بچے قوم کو گمراہ کرنے والے بھڑوے وطن فروشوں کے بوٹ چاٹنے والے تمہارا انجام دنیا عبرت کے طور پر یاد رکھے گی | 1 |
|  | 377 | اگر تب تو نے اشمت سے اپنی چوت نہ پڑوائ ہوتی تو آج تیرے گھر کُتے کا بچہ نا ہوتا مادرچود رنڈی | 1 |
|  | 395 | بھڑوت اعجاز پادری اپنے کتوں کے گلے میں پٹہ ڈال دو ورنہ ہم نے مارنا شروع کیا تو بھاگنے کا بھی موقع نھی ملے گا | 1 |
|  | 1891 | کسی کنجری کے بچے منہ سنبھال کے بات کر جو بھی ہے تُو سامنے آ کر بات کرنے كتی کا پُتر | 1 |
|  | 1068 | ابے ہجڑے اس وقت تمھاری غیرت کہاں تھی | 1 |
|  | 1394 | شکر ہے پیپروں میں یہ سوال نئی آتا کہ لڑکیاں کیسے پٹائی جاتی ہیں؟ ورنہ میرے جیسا معصوم انسان تو ہر سال فیل ہی ہوتا | 0 |
|  | 2513 | کھسرا کھسرا ہوتا ہے | 1 |
|  | 3165 | نبیوں پر کتابیں اُترا کرتی ہیں | 0 |
|  | 2159 | تیری ماں رنڈی تھی تیرے باپ نے تیری ماں کی بلیو وڈیو بنا رکھی تھی اسکو بلیک میل کرتا تھا | 1 |
|  | 1744 | میں نے تو جس پر لکھا اس کو بتایا | 0 |
|  | 385 | جی جی جیسے صبح صبح آپ نے صدر محترم کی طبیعت صاف اوہ سوری دریافت کی | 0 |
|  | 465 | کشمیر نہ کبھی بھارت کا حصہ تھا ، نہ ہے اور نہ ہی بننے دیں گے | 0 |
|  | 1382 | بہت گھٹیا انسان | 0 |
|  | 2898 | بہن چود وراثت میں تو اس کے باپ کی ریال میں جو باقی بچنا تھا وہی ملنا تھا | 1 |
|  | 1377 | وہی جو قطری کے پاس مریم بھیجتا تھا مودی کی ماں کو ساڑھیاں بھیجتا تھا نوازشریف مادرچود | 1 |
|  | 1514 | بےشرم ڈاکو رانی | 1 |
|  | 420 | تیری بیٹی کی کوس مبارک۔ بہن چود ، بیٹی چود، حرامی ، دلال | 1 |
|  | 1179 | شیح راشید اور کتے کے بھونکنے میں کوئی فرق نہی | 0 |
|  | 1045 | ساری عمر ایم کیو ایم اور پیپلز پارٹی نے کراچی کے فنڈز کھائے کتے نسل سال سے کراچی حیدرآباد پر قابض ہو ۔ سارا پیسہ کھاکھا کر بیڑا غرق کردیا | 1 |
|  | 244 | اس کی ہو یا نہ ہو تم لوگوں کی وہ ایسی گانڈ مارے گا نہ، دنیا میں نہ کسی کو بتا سکو گے نہ چھپا سکو گے | 0 |
|  | 315 | بھاڑے کے ٹٹو کشمیر بیچنے والوں کی دلا گیری جاری رکھو۔ | 1 |
|  | 344 | عمران خان نے اپریل کو کھلم کھلا لوٹ مار کیخلاف تحریک انصاف قائم کرکے سیاسی میدان میں قدم رکھا | 0 |
|  | 3259 | کینجھر جھیل کراچی کی پیاس بجھاتی ہے تو کراچی بھی باہر سے آئے کنجروں کی بھوک مٹاتی ہے۔ | 1 |
|  | 3206 | اےربِ کعبه ہمارےملک کو اَمن کا گہوارہ بنادے | 0 |
|  | 3221 | تیری ناپاک ترین فوج ہی اس کو اٹھا کر کے گئی تھی جب اس گشتی کو گولی لگی تھی تو اب بتاء تجھ پر لعنت بھجوں | 1 |
|  | 2180 | یہ نوازشریف اور آصف زرداری کی حکومتی پالیسی کی وجہ سے آج ہم اس مقام پر کھڑے ہیں کہ انڈیا جیسا ملک ہم کو دھمکی دیت… | 0 |
|  | 2554 | ڈیزل اب تک ہوا میں تحلیل ہو چکا | 0 |
|  | 1473 | کشمیریوں تمہارے ہاتھ کے یہ پھتر ہمارے بزدل اور عیاش حکمرانوں کے منہ پر ایک زوردار طمانچہ ہیے | 0 |
|  | 1356 | اور کتوں کے بچوں بینرز تو کیمروں میں دیکھ لیتے ہو | 1 |
|  | 106 | سوشل میڈیا پر پاک آرمی کوانڈیاکے خلاف جنگ ناچھیڑنےپرطعنےدےرہےہیں۔ | 0 |
|  | 761 | پاک فوج کا منہ توڑ جواب، افسر سمیت بھارتی فوجی ہلاک | 0 |
|  | 648 | یہ اسٹیبلشمنٹ بول رہی ہے آج الطاف بھائی کے بنا ایک کتے کو پٹا ڈال رکھنا ناممکن ثابت ہوا | 1 |
|  | 2636 | نطفہ حرام کے کسی چلتی پھرتی آوارہ کھوتی کے بچے کیا ثبوت ہیں تیرے پاس رنڈی کے بچے ایسے فضول بکواس مادر چود کمینے | 1 |
|  | 1354 | بکواس بند کر حرامی کتے | 1 |
|  | 493 | بلاول کے اسمبلی میں پابندی عائد کی جائے تمام کنجروں بے غیرتوں کا نیازی کو بے غیرت کہنے پر احتجاج اور مطالبہ | 1 |
|  | 1655 | مودی میں تیرا خون پی جاؤں گا | 0 |
|  | 1533 | تمہاری ماں کو پیلی ٹیکسی بنایا تم چپ ننگی تصویریں بنا کر بانٹی گھٹیا الزام لگائے تمہاری ماں پر تم چپ | 1 |
|  | 1272 | پاک فوج کی طرف سے آج اگست کو بھارتی فوج کی پوسٹوں کو تباہ کرتے ہوئے کی ویڈیو ۔۔۔۔۔۔ | 0 |
|  | 1616 | جب سے تجھے عمران حکومت نے ایمبیسیڈر کی پوسٹ سے ہٹایا ہے تیرے بک بک اور بکواس میں اضافہ ہوا ہے اور بیویوں کو بکواس ک… | 0 |
|  | 1465 | آصف زرداری کے اندر اس خبیث انسان کا خون دوڑ رہا ہے جو قائد اعظم کے لیے ایسی زبان استعمال کرسکتا ہے | 1 |
|  | 1030 | لعنت ہے کنجر حکمرانوں پر جو خاموش ہیں کل جب انکی بہنوں پر ہاتھ آئے گا تب انکو اس تکلیف کا اندازہ ہوگا | 0 |
|  | 2929 | کون یہ ہے کنجر کا بچہ دلا حقانی نیٹورک کا کُتا لگتا ہے جس انداز سے بھونک رہا ہے بیغیرت کی نسل | 1 |
|  | 3280 | بہن چود پیپلے | 1 |
|  | 3191 | شیدے ٹلی مادرچود جا مر کشمیر تے مادرچود | 1 |
|  | 3104 | اس خبیث انسان کو پاک فوج سے ہی نہیں بلکہ پاکستان سے دشمنی ہے لعنت اس پہ اور اس کی نسل پہ | 1 |
|  | 177 | اگربھارت سےسفارتی تعلقات ختم کیےتھےتوبھارتی کنجروں کوویزےکس نےدیےاورکس نےان کنجروں کودعوت دی؟ | 1 |
|  | 3069 | پٹواری ہے یا جیالا دونوں ہی بہن چود ہیں | 1 |
|  | 2535 | جس کا آپ دفاع کرنے آئے ہیں پہلے اس کی پروفائل تو چیک کر لیں مجھے کسی کیسی گالیاں دی ہیں | 1 |
|  | 3441 | ایسے کتوں کو گانڈ میں گولی مارنی چاہیے | 1 |
|  | 2468 | تیری بھگوڑی ماں۔ بےبے مریم صفدر اعوان | 1 |
|  | 2957 | کشمیری رہنما شہلا رشید کا ہولناک انکشاف | 0 |
|  | 857 | آوے رنڈی کے بچے عید والے دن میری زبان گندی نی کروا میں تیری بہن چود دینی ہے بھڑوے | 1 |
|  | 3458 | او دلے کسی گشتی کی حرامی نسل تیرا بھونکنا بند نہیں ہوگا لیکن کسی دن اس کی طرح عوام کے ہتھے آیا تو مر جاۓ گا | 1 |
|  | 305 | مودی تیرے دن گنے جاچکے ہیں اور جلدی کتے کی موت مرے گا | 0 |
|  | 2337 | ڈیل پوئ کہ ارسطو کی گانڈ مودی مارے گا جبکہ مریم یم یم روایتی طور پر قطری کو پیش کی جائے گی | 1 |
|  | 2399 | ھمیں پورا یقین ہے تم غدار ہے مادر چود اس حالت سے ھم نکل جاینگے | 1 |
|  | 1032 | ابے بھڑوے کی نسل | 1 |
|  | 2998 | کشمیر کو پاکستان کے ساتھ ملائیں یا آزاد ریاست، بس کشمیر کو ہندو بنیۓ سے آزادی دلوائیں۔ وہاں انسانیت کی تذلیل ہو رہی… | 0 |
|  | 1958 | اس کتے کے بچے کھسری کو گولی مار دینی چاہئے | 1 |
|  | 830 | عمران خان کا آذاد کشمیر کی قانون ساز اسمبلی سے زبردست بیان | 0 |
|  | 2150 | بہن چود سارے ملک تباہ کر گیا | 1 |
|  | 611 | پاکستانی انتظار میں ہیں کہ ک… | 0 |
|  | 2077 | منہ میں ڈال لے اس کا بھوسڑی کے گانڈو انڈینز کو میجر صاحب اور اپنوں کو گالی تیرے جیس… | 1 |
|  | 1966 | مودی کتامودی کتامودی کتامودی کتامودی کتامودی کتامودی کتامودی کتامودی کتامودی کتا، کافر کافر کافر تو ہلاک ہو | 0 |
|  | 3268 | براہمن کو چھوڑ کر تقریبا ساری انڈین فوج ہتھیار واپس کرنے والی ہے | 0 |
|  | 662 | مارو ان بہن چودوں کو ورنہ سب ایک ایک کر کے ان چوروں کی حرکتوں کی وجہ سے مار دیے جاؤ گے | 1 |
|  | 1044 | یہ بہن چود بچ کیسے گیا ھے | 1 |
|  | 2615 | قوم کو کرپشن کی بتی کے پیچھے لگا کشمیر کا سودا کرنے والے زلیل انسان | 1 |
|  | 951 | کتنے حرامی ہیں نا تمھارے سالے بوڑھے بیمار باپ کو ایک بار جیل ملنے نہیں آۓ ۔ خود اشتہاری ہیں بوڑھا باپ | 1 |
|  | 3173 | نمونے جتنی اپنی صفائیاں دے رہے ہو اس کا آدھا بھی اگر کام کیا ہوتا نہ تو ایسے زلیل و خوار نہ ہوتے | 0 |
|  | 840 | ہم انکی طرح نہیں جو بچو اور شہریوں پر حملہ کر کہ خود کو بہادر ثابت کریں | 0 |
|  | 1838 | کرکٹ میچوں پر جوئے کی لت نے ہمارے شہر کے متعدد گھرانے تباہ کر کے رکھ دئیے ہیں ارباب اقتدار و اختیار خدارا توجہ دیں | 0 |
|  | 1015 | بلکل صحیح کہا اگر حج الوداع کا خطبہ یاد رہتا تو اس وقت اس طرح زلیل وخوار نا ہو رہی ہوتی | 0 |
|  | 2286 | بیغیرت، زلیل انسان کہاں غائب ہے کتے کی اولاد، اب بھونکنا یاد نہیں؟ | 1 |
|  | 763 | اپنی جھوٹی اور چھوٹی انا پر پاکستان کے مستقبل کو تباہ نہ کرو بلکہ | 0 |
|  | 601 | بہن چود علی وزیر اور محسن داوڑ کے پروڈکشن آرڈر کیوں جاری نہیں کرتے ہوں دلال کے بچے | 1 |
|  | 2896 | علامہ حافظ خادم حسین رضوی صاحب کی طرح اگر کسی نے بھارت کو للکارا ہے تو سامنے لاو | 0 |
|  | 2293 | سمجھ سے بالا تر ھے کہ ای ایس پی ار کے جرنل غفور صاب کسی کتے کے غلط وقت بھونکنے پر ٹویٹیں ٹھوک دیتے ھیں | 1 |
|  | 255 | پاکستان ہی اسلامی دنیا کا حقیقی رہبر ہے | 0 |
|  | 2984 | کسی کتی کی بچے، مادرچود بہت ہی گھٹیا ہو تم، حرام کی پیداوار ہو تم، تمہار… | 1 |
|  | 1225 | میرا دل خون کے آنسو روتا ھے سب دھوکا ھے نا سلامتی کونسل نے کچھ کرنا ھے نا ٹرمپ نے | 0 |
|  | 2198 | جو دھندے تم سندھی بھڑوے کرتے ھو وہی بیان کر رھے ھو گانڈو | 1 |
|  | 551 | عامر لیاقت کا دبے الفاظ میں بلاول زرداری صاحبہ کو کرارا طمانچہ۔۔۔ | 0 |
|  | 1687 | ہر ناکامی کے بعد انسان کے پاس دو راستے ہوتے ہیں | 0 |
|  | 1401 | کشمیر کے سوداگروں کان کھول کر سن لو ہمیں تمارے اس سودا پر لعنت بھیجتے ہے | 1 |
|  | 2779 | یہ ماں یہ بہن یہ بیٹی جس کے ساتھ درندگی کرکے پھانسی دی گئی کشمیر میں اور آجکل یہ کام عروج پر ہے کشمیر میں | 0 |
|  | 3006 | تمام حکمرانوں نے آئ ایم ایف اور ورلڈ بینک کے پاس اس ملک کو گروی رکھ کر خود عییاشیں کی اور عوام کو زندہ درگور کردیا | 0 |
|  | 2794 | تیری ماں کے یار مودی کی بھی گانڈ مارے گے اور تجھ جیسے کتے جو پاکستان پر بھوکتے ہیں ان کی بہن بھی چودے گے | 0 |
|  | 2387 | الو کا پٹا بےغیرت بےشرم زلیل بکواسی انسان پشاور بھی دیکھاو غیرت پکڑو | 0 |
|  | 2479 | اب یہ کیا حرامی پن ہے ؟؟ | 1 |
|  | 1758 | ڈنگر دے بچے تو انسان ہے ہی نہی خبیث | 1 |
|  | 1658 | کرنے دو جو بکواس کرتے ہیں ۔۔۔۔۔ | 0 |
|  | 523 | پاکستانی ٹویپس نے ہندوستانی فوج کے دانت کھٹے کر دیے | 0 |
|  | 3303 | پاگل ہے جو آئیگا خوشبو بلکہ خشبوو لگاکر زندہ درگور ہونے کیلئے | 0 |
|  | 790 | اوئے خبیث النسل انسان تم آج بھی اپنی اور ناکام حکمران کے چرچوں کے چکر میں لگے بیغرت انسان ان کو چھوڑ | 1 |
|  | 1666 | جب اپنے جرائم کا ارتکاب کر لیا، اور جرائم بھی وہ، جو آج تک پاکستان کی تاریخ میں جس نے بھی کیے اسے نشان عبرت بنادیا… | 0 |
|  | 1691 | گشتین صاحب کشمیر کی بات کر لو مادرچود | 1 |
|  | 3047 | شرم سے ڈوب مرنے کا مقام ہے بیغیرتوں کے لئے۔ | 1 |
|  | 3420 | ماں کی کس میں جائے یہ پہلے عوام کا سوچ لو یہ بہن کی لوری تو پھر بھی اچھی جگہ جا رہی ہے یہ دیکھو میرے | 1 |
|  | 425 | بھونک مت تو دلی کی اولآد | 1 |
|  | 358 | روزایی که سر کارم منتظر تعطیلاتم و توی تعطیلات منتظر روز کاری همینقدر تباه | 0 |
|  | 2741 | مادرچود انگریزی تو ڈھنگ سے لکھنا سیکھ لے | 1 |
|  | 334 | مطلب کشمیر جب بھی آزاد ہوا بنگلہ دیش والوں کی طرح جماعت حرامی کے کارندوں کو چن چن کر لٹکا کر نشان عبرت بنا دے گا | 1 |
|  | 3494 | مادرچود دو دودن ٹرینڈ میں ذلیل ہونے کے بعد آج اپنے باپ نواز شریف کی آپشن شامل ہی نہیں کی سب مادرچ | 1 |
|  | 2454 | کشمیر جل رہا ہے کشمیری زندہ درگور ہیں وہ موم بتی مافیا کہاں مر گیا وہ رنگ برنگی ٹوپیوں والے کدھر گئے وہ درباروں پر… | 0 |
|  | 2653 | بھائی صرف اپوزیشن یا انکے فولوورز ھی نہیں یہ نام نہاد سوشل میڈیا ایکٹیوسٹ بھی شامل ھین | 0 |
|  | 2784 | ادارے شخصیات کے محتاج نہیں ہوتے۔ آرمی چیف کو ایکسٹنشن وزیر اعظم ہمیشہ اپنی کرسی بچانےکیلیے دیتا ہے۔ جنگ عظیم کے دور… | 0 |
|  | 2852 | بکواس کر رہا ہے یہ سالہ گندہ انڈا رپورٹنگ کرتا تھا فیس بک پر بیٹھ کر | 1 |
|  | 1840 | اس کتے کے بچے کھسری کو گولی مار دینی چاہئے | 1 |
|  | 353 | بکواس بند کر بھڑوے سالے عمران خان نواز شریف نہیں جو ذاتی مفادات کی خاطر قوم سے جھوٹ بولے گا | 1 |
|  | 1361 | پونچھ سیکٹرز میں کئی اہم بھارتی چیک پوسٹیں تباہ کئی بھارتی فوجیوں کے جہنم واصل ہونے… | 0 |
|  | 2936 | بیچارے یوتھیوں نے پتا نہیں کیا کیا بیچ کر بڑے اہتمام اور جذبے کے ساتھ ڈیم فنڈ میں پیسے جمع کروائے تھے اور وہ خاندان… | 0 |
|  | 1323 | لعنتی مردود مبشر ذیدی کا کام ہی اسلام اور اسلامی احکام شریعت پرتنقیدکرنا ہے | 1 |
|  | 3478 | فوراا پابند سلاسل ھونا چاہئے اس کنجر کو | 1 |
|  | 3145 | مبارک ہو آپکے پڑوس میں نمک حرام ہوا ہے | 0 |
|  | 868 | ن لیگیوں،لفافوں اور غداروں کا باجوہ صاحب کے بطور آرمی چیف تین سال مدت بڑھنے پر بکواس اور بھونکنے کا کام شروع ہو گیا… | 0 |
|  | 1301 | کمال ہے جو بھگوڑے پوری مہاجر قوم کو چھوڑ کر لندن میں سال سے بیٹھے مودی کی یاری کر رہے ہیں۔ جن کی خود | 1 |
|  | 2377 | یہ گشتی دشمن کی آلہ کار ہے | 1 |
|  | 838 | جس طرح پٹواری بکواس کر رہے ہیں اس طرح تو انڈین کتے بھی نہیں کر رہے بہت گھٹیاں سوچ ہے پٹواریوں کی۔… | 0 |
|  | 2602 | تیری پئین دی چھولی گشتوڑ دے بچے گٹر کی پیداوار اب تیرا وقت بھی آچکا ہے تم نے افواج پر طنز کرکے سینکڑوں | 1 |
|  | 3180 | راولپنڈی لائن آف کنٹرول کے تتہ پانی سیکٹر پر بھارتی فوج کی فائرنگ سے سالہ بچے سمیت افراد شہید | 0 |
|  | 2772 | بیغیرت تماشائی قوم | 1 |
|  | 3424 | مودی کتے مار جا تیری ماں نو | 1 |
|  | 696 | گھٹیا عورت اس میں سوال کہاں ہے ؟ | 0 |
|  | 3374 | فخر پاکستان جنرل راحیل شریف ۔ اس سے پہلے شاید پاکستان کو آپکی اتنی ضرورت تھی۔ مگر آج بہت ہے ۔ | 0 |
|  | 1280 | تیرے جسیے بہن چود تب پیدا ھوتے ہین جب تیری مان کتون سے پوری رات چدواتی ھے | 1 |
|  | 83 | نکو ڈی چوک میں ھی الٹا لٹکا کر انکی تشریف پہ چھترول کی جاے ٕ | 1 |
|  | 170 | تو بڑا بہن چود بندہ ہے کسی گشتی کے بچے یہ اداروں کی مرضی ہے | 1 |
|  | 1365 | تیرے جیسے دلال سارا دن بیٹھ کے اپنی بہنوں کے ٹکٹ کاٹتے ہیں اور شام کو پک اینڈ ڈراپ کی سہولت بھی مہیا کرتے ہیں | 1 |
|  | 1635 | اس کو کس مادرچود حرامی نے پاکستان بلوایا اس کی گ میں ڈنڈا ڈال کر اس بھی میکاسنگھ کے ساتھ انڈیا بھیج دو | 1 |
|  | 1092 | ہم نے روایات،رسوم اور مزھب کو گڈمڈ کر دیا ھے۔ | 0 |
|  | 1646 | جب کسی کا پروفیشن سوشل میڈیا پہ ہی زندہ ہو اور وہ اپنی زندگی کا اظہار انہی پلیٹفارمز کو لے کے کر رہا ہو تو پتہ نہیں… | 0 |
|  | 3008 | بابا جی فرماتے ہیں میں کچھ نہیں فرماتا۔ | 0 |
|  | 1156 | کشمیر آزاد کروانے کے لیے اس کی بیٹی کو چوک میں چودا جائے ۔سالا حرامی بہن چود ۔شامی کتا سور | 1 |
|  | 168 | ارسطو شرم کر حیا کر ڈوب مرو تالپور پے تمہارا غیرت جاگا ملک کا بیڑا غرق کرکے تمہارا غیرت نہی جاگا | 0 |
|  | 527 | کتے کے بچے عوام مہنگائی کی وجہ سے زلیل ہو رہی ہے تم پائوں چاٹنے میں مصروف ہو | 1 |
|  | 3479 | نام ہی بدل لوکم از کم اگر ایسی بے تُکی بکواس کرنی ہے۔۔۔ ہمسفر دا پُتر | 0 |
|  | 509 | پاکستان میں چوروں کے لئے پیغام ہے کہ ڈاکہ مارنا ہے تو بڑا ڈاکہ مارو یقین کرو جیلوں میں بھی وی آئی پی ماحول ملے گا | 0 |
|  | 1663 | ہتھیلی سامنے کرو تمہارے ہاتھ پر مہندی سے پھل بوٹے بناوں پھل بوٹے ٹھیک ہیں نا؟ یا پھر جان شاہ لکھوں؟ | 0 |
|  | 128 | جو لوگ بکواس کرتے تھے کہ ابا بچاو مہم شروع ہوئ ہے انکو مبارک ہو آج ابا کو انکے لیڈر نے بچا لیا کیسے نہ بچاتا خود بھ… | 0 |
|  | 1989 | کیوں ہنس کر کرتی ہو میری بےبسی کا مزاق کیا مجھ پہ اعتماد نہیں میں نے تو اپنی جان کو تیرے قدموں کے نیچے رکھ دیا | 0 |
|  | 1576 | اسی معمولی سیلیکٹیڈ نے نواز مادرچود کو لنڈ کے چوپے کروائے ہیں اور تمہاری لیڈرنی کو بھی | 1 |
|  | 491 | گلگت چودہ اگست کا پروگرام دیوار گرنے کے باعث متعدد افراد زخمی ہونے کی وجہ سے منسوخ ہلاکتوں کے خدشات | 0 |
|  | 2940 | اس بہن چود کی گانڈ میں بھی ڈنڈا دو | 1 |
|  | 12 | ھاں بھای نمک حرام کہاں ھو | 1 |
|  | 293 | دلیل کا جواب دلیل سے دیا جاسکتا ہے ہر زلیل کو جواب دینا وقت کا ضیاع ہے | 0 |
|  | 2500 | کون تیری ماں کا ٹھوکو تھا جس نے کشمیری خریت قیادت سے ملنے سے انکار کیا ؟؟ | 1 |
|  | 1540 | جس کو کرسی کی حوس ھو وہ کسی کے لے جہاد نہیں کر سکتا | 0 |
|  | 3125 | اسپتال میں علاج کے دوران ہوئی شخص کی موت | 0 |
|  | 3485 | بھارت سندھ طاس معاہدے کی خلاف ورزی کررہا ھے ۔ | 0 |
|  | 1070 | یہ عورت کس چینل کی خبر ٹویٹ کر رھی ھے بھن چود سب کنجر ھی | 1 |
|  | 47 | بہن کی چوت قادیانی کے اور ماں کو لوڑا دو اس کو جو بھین چود کسی پہ بھی قادیانی کا الزام لگاے | 1 |
|  | 2586 | پاکستان کے بہادر وفاقی وزیر جناب نے ٹویٹر پر بھارتیوں کی نیند حرام کر رکھی ہے | 0 |
|  | 2752 | پٹواری ہے یا جیالا دونوں ہی بہن چود ہیں | 1 |
|  | 2250 | گشتی کے بچے، دروازے کی چابیاں تیرے باپ باجوہ کے پاس ہیں۔ مادرچود اس کا نام لے جس نے تیری ماں چودی ہے۔ بھڑوا | 1 |
|  | 1391 | ن لیگ ایک جماعت نہیں ایک سوچ ہے اور نہایت ہی گھٹیا اور گندی سوچ ہے | 0 |
|  | 1349 | سلیکٹڈ کے منہ پر طمانچہ | 1 |
|  | 972 | ہوگئی بکواس ختم ؟ چل اب نکل یہاں سے ورنہ | 0 |
|  | 2184 | تیری ماں یہ بکواس پڑھے تو اسے ضرور شرم آئے گی ایسا حرام زادی پیدا کرنے پہ | 0 |
|  | 1659 | وسے بڑے بڑے بےغیرت دیکھے پر پاکستانی انپڑھ میڈیا اور منسڑ کنجر بے غیرت پہلی دافعہ دیکھ رہا ہوں | 1 |
|  | 984 | بہن چود پٹواری ۔۔۔خود سے اپنے ابو نواز شریف چور کی تاریخ لکھنے بیٹھ جاتے ہیں۔ | 1 |
|  | 2311 | اے انڈیا والو کان کھول کر سن لو آپ جیسی مرضی سازش کر لو | 0 |
|  | 820 | کچھ لوگ سوال اٹھاتے ہیں کہ اسرائیل بھارت کا ساتھ بار بار کیوں دیتا ہیں | 0 |
|  | 120 | لٹکاؤ اس مادرچود کو | 1 |
|  | 366 | مادرچود بکواس بند کر | 1 |
|  | 135 | یہ قانون ،ریاست اور انسانیت پر سوالیہ نشان اور طمانچہ ہے۔ | 0 |
|  | 2823 | بیغیرتوں بھڑوں کی بستی میں واپس آؤ۔ | 1 |
|  | 1773 | جو نام نہاد لنڈے کے صحافی اور خونی لبرلز دن رات راگ الاپتے کے فوج سارا بجٹ کھا جاتی اور | 0 |
|  | 620 | نکل لوڑے پھیلی فرصت میں نکل بھوسڑیکے مادرچود | 1 |
|  | 2060 | بہار میں ریٹائرڈ آئی پی ایس آفیسر اور بیٹے کی پٹائی | 0 |
|  | 408 | بھارت کا لائن آف کنٹرول پر بلا اشتعال انگیزی اورجنگ بندی کے قوانین کی خلاف ورزی کرنے پر پاک فوج کا منہ توڑ جواب۔ | 0 |
|  | 151 | بکواس نا کریں | 0 |
|  | 2201 | جس شخص سے عید ملا وہ رو رہا ہے۔ چاہے کو مڈل کلاس بزنس مین ہے تو چاہے کوئی ایک عام ملازم | 0 |
|  | 375 | کسی دن پبلک نے یہ حال کرنا ہے تم بُزدل بیغیرتوں کا | 0 |
|  | 2512 | حرامی بہن چود حکومت | 1 |
|  | 478 | انڈین میڈیا جنرل قمر جاوید باجوہ کی ایکسٹینشن پر رنڈی رونا ڈال رہا ہے پاکستان میں پٹواری ، جیالے ، اور لفافہ خور صحافی | 1 |
|  | 2976 | یہ عورت کس چینل کی خبر ٹویٹ کر رھی ھے بھن چود سب کنجر ھی | 1 |
|  | 2426 | اس کتے کا پٹہ کس نے کھول دیا | 1 |
|  | 387 | اس ذات کی قسم جس کے ہاتھ میں میری جان ہے تم نیکی کا حکم دو | 0 |
|  | 2073 | نہ کوئی چاقو ۔ نہ کوئی خنجر مودی کنجر مودی کنجر | 1 |
|  | 1145 | آپ ان کے نام مینٹین کرتی جانا ہم طبیعت صاف کرتے جائیں گے | 0 |
|  | 1953 | یہ تو عمران خان بھی کہا تھا کہ ہم نے وہاں ظُلم کیا… | 0 |
|  | 1186 | بھارتی فوج نے ہسپتال اور سکول پر حملہ کیا ہے بدلے میں پاک فوج نے انکے بھارتی فوجی جہنم واصل، چیک پوسٹ اور بنکرز… | 0 |
|  | 77 | زندہ رہتے ہوئے ہمارے اندر جو مرتا ہے | 0 |
|  | 3302 | حرام پر پلی ہوئی نسل ایسی ہی گھٹیا ہوتی ہے | 0 |
|  | 3380 | اگست تک سیلیکٹڈ وزیراعظم عمران احمد خان صاحب خود مستعفی ھوجائیں۔ ورنہ اکتوبر میں ہم اسلام آباد آئیں گے۔ | 0 |
|  | 1887 | سنا ہے یہ گشتی دماغی مریض بن گئ ہے۔ مریم اورنگزیب گشتی | 1 |
|  | 3477 | یہ جاھل عورت جو کوئی بھی ہے خواہ وہ کتنی ہی پہنچ والی کیوں نہ ہو اسے سخت ترین سزا ملنی چاہئے | 0 |
|  | 2563 | بکواس سن لو اس اردو بولنے والے کی۔ | 0 |
|  | 3243 | میں تو کومنٹ میں اسکو بہت زلیل کرتی ہوں فیسبک کبھی نہیں اڑا یے ٹویٹر اڑ جاتا ہے جلدی | 0 |
|  | 1936 | اور تو مراثی ابھی بھونکنے کے لئے زندہ ہے۔ کے پی کے کتنی لوگ مر گئے ۔۔ مگر تیرے جیسے بیغیرتوں کی غیرت نہیں جاگی۔ | 1 |
|  | 1335 | کتوں کے بھی حقوق ہے | 0 |
|  | 833 | عظمی چنڈال | 1 |
|  | 233 | ہائے میرے اللہ۔۔۔۔اور کتنی قیامتیں دنیا میں ہی دیکھنے کو ملیں گی | 0 |
|  | 14 | گلگت ہیلی پیڈ میں دیوار گرنےکا افسوس ناک وقعہ پیش آیا ہے | 0 |
|  | 814 | مہینہ ہوا لاتعداد کمپلینٹ کے باوجود گھر کے قریب ٹاور میں زونگ فور جی ٹھیک نہیں ہوا۔ | 0 |
|  | 1601 | بھونک مت تو تو کی اولآد | 1 |
|  | 2780 | تیرے جیسے جس مادرچود نے سودا کیا وہ کوٹ لکھپت میں سڑ رہا ہے آج کل ،تو اپنا پچھواڑا بچا جو کسی دن پھٹنے والا ہے حرام | 1 |
|  | 2449 | خان صاحب خدا کے لئے عوام پر رحم آج کل ذرداری اینڈ نواز شریف مولانا ڈیزل ایسی مہم چلی ھے مہنگائی کے نام پر کہ عوام… | 0 |
|  | 3281 | اس کھوتی کے بچے نمک حرام پر سب پاکستانی لعنت کریں | 1 |
|  | 3474 | ابھی گنجن کی اولاد تیرا بھی وقت آجائے گا بڑے بہت خوب بکواسیات کر رہا ہے مادرچود | 1 |
|  | 3103 | پوری مسلم امت کے منہ پر طمانچہ | 1 |
|  | 179 | اہو اب بہت ساروں کی گانڈ میں چالیس ملک امت امت والا کیڑا سٹ مارے گا | 0 |
|  | 2407 | جیسی گانڈو لیڈر شپ ھے ویسے ھی فولورز بھی ہیں ۔ | 1 |
|  | 2953 | بچپن سے سنتے آ رہے ہیں کہ خاص ہی طاقتور ہوتا ہے مگر اگست کو آم سے زیادہ طاقتور کوئی نہ نکلا | 0 |
|  | 1400 | او کسی کھوتی دے پتر مری ہوئی ماں کو کیوں گالیاں نکلواتا ہے بے غیرت تو توں ہے تیرا پورا خاندان ہے | 1 |
|  | 793 | کاش تم میں تھوڑی سی اسلام سے اگائی ہوتی۔ | 0 |
|  | 2909 | کتوں اور کنجروں کی حکومت اور آپ جیسی گشتیاں رنڈیاں وزیر ہوں تو کشمیر کیا کچھ مہینوں میں پاکستان کا بھی سودا ہو جائے گا | 1 |
|  | 2179 | پنڈی کے ہر لڑکے نے اس کی گانڈ ماری ہوئے ہے | 1 |
|  | 1355 | قوم کو ماموں نا بناؤ | 0 |
|  | 1399 | دو مادرچود لاہور کے جنہوں نے رابعہ خان نامی لڑکی کی عزت لوٹی | 1 |
|  | 1612 | کتے کبھی شیروں کا مقابلہ نہیں کرتے یہ بات اپنے دماغ میں بٹھا لینا | 1 |
|  | 3102 | کیا بکواس ھے۔۔۔۔۔۔ | 0 |
|  | 410 | ہمیں اب انتظار کی بجاۓ ان ہندو کتوں کو سبق سکھا دینا چاہۓ | 1 |
|  | 290 | تیرے جیسے جس مادرچود نے سودا کیا وہ کوٹ لکھپت میں سڑ رہا ہے آج کل ،تو اپنا پچھواڑا بچا جو کسی دن پھٹنے والا ہے | 1 |
|  | 1761 | یہ بلو کھسری سمجھتی ہے کہ وزیراعظم پر گھٹیا بکواس کرکے لیڈر بن جاے گا لیکن اس کو یہ نہیں پتہ | 1 |
|  | 543 | اسکی شیخ رشید نے گانڈ ماری ہوگی | 1 |
|  | 726 | یہ گانڈو خود کو سندھی بولتا ھے مگر فخر بلوچ ھونے پر کرتا ھے | 1 |
|  | 418 | اور تیرے جیسے بے غیرت کو بھڑوا کہا جائے ۔۔۔ بہن چود شرم نہیں آتی چوروں کو ڈیفنڈ کرتے ہوئے ، بے حیا انسان | 1 |
|  | 3487 | ان کنجروں گلیوں اور بازاروں میں گھسیٹو | 1 |
|  | 203 | کیا آج زندہ دلان لاھور نیب احتساب عدالت مریم نواز شریف سے اظہار یکجہتی کے لیے پہنچیں گے ضرو پہنچیں گے | 0 |
|  | 1844 | اور جس طرح سے تم بیغیرتوں نے عوام کو بے دردی سے لُوٹا ہے اس سے تم لوگوں کا حرامی پن نظر آتا ہے | 1 |
|  | 376 | جلد یا بدیر دنیا جان لے گی پیٹ کاٹ کر اسلامی دنیا کی الحمدللہ طاقتور فوج پاکستانی فوج ہے | 0 |
|  | 2199 | گالی دینے والوں حرامیوں وقت تمیں معاف نھیں کرینگے انشاءاللہ کتے کی موت مرو گے سب کے سب حؤس کے پجاریوں | 1 |
|  | 1670 | مودی کی پریس ریلیز نے محبوبہ مفتی اور اسکی حواریوں کے منہ پر طمانچہ جڑ دیا۔ | 0 |
|  | 2432 | پکڑ کر اس کی گانڈ مارے نیب صرف گانڈ پھاڑے مار مار کر اور مریم صفدر کی چیخیں نکلوائے | 0 |
|  | 1583 | بھونک مادرچود بھونک | 1 |
|  | 1718 | میں کل سے سن رہا ہوں یہ میڈیا، صحافی اور اینکرز جنرل باجوہ کی ایکسٹینشن کو لے کر بہت بکواس کر رہے ہیں میں ان سے پوچ… | 0 |
|  | 1347 | جن جن کے اکاؤنٹ سسپینڈ ہوئے ہیں سب انکو فالو کریں اور انکے اکاؤنٹ زیادہ سے زیادہ شیئر کر کے | 0 |
|  | 815 | ان سب مراعات کے باوجود اگر ملکی مفاد پر سودے بازی ھوتی ھے تو اس بے شرم زندگی سے موت اچھی | 0 |
|  | 85 | تیری زندگی پہ لعنت آؤ خبیث انسان | 1 |
|  | 372 | فیسبک سوشل میڈیا پہ اتنے ہمدرد لوگ ہیں کہ اپنے حصے کا گوشت۔ بھی غرباء میں تقسیم کر دیتے ہیں | 0 |
|  | 2266 | اس سے پتا چلتا ہے۔ شریف فیملی میں عورتیں اور ہجڑے ہیں بس۔ | 1 |
|  | 371 | جس نے بھی ان چوروں کو عزت دیں اللہ پاک کی ذات انکو عبرت کا نشان بنائے کیونکہ ان درندوں نے میرے ملک کو لوٹا ہے | 0 |
|  | 1664 | جنگ ہمیشہ اپنے قوت بازو پر لڑی جاتی ہے۔ آج کشمیری مائیں بہنیں مدد کے لیے پکار رہی ہیں | 0 |
|  | 881 | پرنٹ الیکٹرونک میڈیا پی ٹی آئ کراچی کے کوآرڈینیٹر عزیر اے صدیقی کے گھر میں چوری کا واقعہ بہت ہی افسوس ناک ہے | 0 |
|  | 597 | آپ بھی تو اپنے اس حسن کے ساتھ نیازی کے بستر کی زینت بنی تھی | 0 |
|  | 2213 | کتی دا پتر لخ دی لعنت | 1 |
|  | 2574 | خان نے سہی کہا تھا کہ میں ان کو رولاؤ گا ان کو تکیف پہنچے گی اوے کنجروں یہ تو پارٹی شروع ہوئی ہے | 0 |
|  | 225 | افسوس افسوس افسوس اقلیت اقلیت کی رٹ لگانےوالےبغیرت کہاں مرگےہیں ان کنجروں یہ ظلم نظر نہیں آرہا | 0 |
|  | 1810 | لکھ دی لعنت تہاڈے منہ چہ کھوتے خور پٹواریوں دی نانی اماں مودی کے یار غدار باپ | 1 |
|  | 1063 | مطلب آپ سکھاؤ گے کہ لڑکیوں کو فیس بُک استعمال نہیں کرنی چاہئے یہ پازیٹو استعمال کرنا چاہئے | 0 |
|  | 948 | کیا بکواس ہے۔ | 0 |
|  | 1208 | لنّت ہو تم جیسے منافق دو ٹکے کی انسان بریانی کی پیچھے بکنے والے | 1 |
|  | 2466 | مودی کے ہندوستان میں جو کچھ ہو رہا ہے، آنکھیں کھول دینے والا زور دار طمانچہ ہے | 0 |
|  | 811 | بس ایک سیدھی سی بات ھے والنٹریز کو چاہیے آفیشل کا اکاونٹ رپورٹ کروا کے سسپنڈ کروائے | 0 |
|  | 2412 | اس بہن چود نظام کو لپیٹ دینا چاہیے | 1 |
|  | 1423 | پپو خالی بکواس ہی کرتا ہے | 0 |
|  | 786 | جنکی انکھوں اور عقل پر پردے پڑے ہوں انکو ہدایت دینا اپنے آپ کو ہلاکت میں ڈالنا ہے | 0 |
|  | 2651 | تو یوں کہہ نا کہ تو لیاری کے گٹر کی پیداوار ہے | 1 |
|  | 3233 | کوس | 1 |
|  | 1623 | بہن چود فیک اکاونٹ والے شرم کرو اب دیکھتے تجھے تیری رپورٹ کرکے | 1 |
|  | 1941 | پاکستان کے قبضے میں ہمارا پائیلٹ ہے پاکستان فوری واپس کرےباقاعدہ پائیلٹ کی تصویر بھی جاری | 0 |
|  | 2985 | سندھ کے باسیوں کو زندہ درگور کرنے والی ڈاکن فریال تالپور کو نیب حکام اسپتال سے جیل منتقل کر رہے ہیں۔ | 0 |
|  | 2850 | مُجھے اپنے اداروں سے گِلہ ہے۔ ان کتوں کو آخر لگام کیوں نہیں ڈالی جاتی؟ | 1 |
|  | 1223 | کسی گشتی کے پتر ہیں یہ حرامی کافر مادرچود | 1 |
|  | 3325 | ایسے ہی ثبوت صیف الرحمن بھی دیتا تھا اور تجھ جیسے بھانڈ شور ڈالتے تھے | 1 |
|  | 13 | قبرستان تجھ جیسے حرامیوں سے بھی بھرے پڑے ہیں | 1 |
|  | 2822 | چہ پدی اور چہ پدی کا شوربہ۔ اور دوسری بات یہ کہ اپنے آپ کو اہم ثابت کرنے کے لیئے… | 0 |
|  | 1098 | جی بالکل انصار عباسی صاحب بھول جائیں ان سلیکٹڈ بیغیرتوں سے کسی اچھے کی امید | 1 |
|  | 2637 | مودی بھڑوا کتے اور حرامی جب بھی کشمیر میں ظلم کرے گا تیری بھارت ماتا کو تباہ کریں گے | 0 |
|  | 2269 | بلاول کے منہ پر اگر مونچھ لگا دی جائے اور کاندھے پر پٹکا رکھ دیا جائے تو کھسرا نہیں کنجروں کا دَلا لگے | 1 |
|  | 1708 | عزت دینے والے بہن کہے بنا بھی دیتے ہیں عزت لوٹنے والے بہن کہ کر بھی لوٹ لیتے ہیں | 0 |
|  | 1506 | کسے دگڑ دلے دی اولاد اتنا بہادر ہے تو بہن نیب کے آگے پیش کرنے سے اچھا تھا خود اپنی پیش کرتا نیب کے سامنے | 1 |
|  | 226 | بلاول زرداری نے ایوان میں کھڑے ھوکر مریم نواز کی گرفتاری پر عمران خان کو بے غیرت کہا | 1 |
|  | 3198 | یہ پاکستان کا وزیر خارجہ بغیرت کتے کابچہ اتنا روک کیوں رہا ہے لعنت ہے اس خنجر پر | 1 |
|  | 2820 | آٶ دعا کرتے ہیں اللہ پاک اس کے غرور اور اس کو جلد سے جلد خاک میں ملا دے۔۔اور اس شخص کو عبرت کا نشان بنادے۔آمین | 0 |
|  | 2309 | ٹوٹل بکواس، | 0 |
|  | 498 | حنا پرویز جیسیاں کیا جانیں کشمیر کے بارے میں ساری عمر نواز فیملی کے تلوے چاٹتی رہی | 0 |
|  | 3200 | کسی گشتی کے بچے کسے کتے کی آوارہ اولاد مادر چود کے بچے تیری گانڈ میں کیڑ | 1 |
|  | 161 | حامد میر پاک آرمی کے خلاف بکواس کرتا ہے ریاست سے یہ اپیل ہے کہ اس پر غرداری کا مقدمہ درج کر کے سزائے موت دی جائے | 0 |
|  | 2112 | ھھھھھھ کمال کی جنگی حکمت عملی ہے نشیوں و کنجروں کی | 1 |
|  | 1636 | تحریکِ T لپاگل P نگڑا L | 0 |
|  | 64 | بیٹی کا دلال چل دفع ہو یہاں سے خبیث انسان بیٹی کا سودے باز | 1 |
|  | 2054 | اگر تیری بات مان لی جائے تو وہ ٹی ٹی کے باوجود عوام کو سہولت دے رہے تھے مفت عل… | 0 |
|  | 1127 | بات احسان جتانے کی نہیں ہے بلکہ بات بیغیرتوں کو غیرت دلانے کی ہے کہ وہ پاک فوج پر بھونکتے ہیں | 0 |
|  | 1535 | غلیظ سوچ رکھنے سے بہتر ہے یہ حجاب اتار دو۔ پردے کے پیچھے چھپا غلاظت کا ڈھیر ہو تم۔ گندی سوچ کا پلندہ۔ گھٹیا لوگ | 0 |
|  | 264 | جب اس کی پکڑ ہوتی ہے انسان تباہ برباد ہوجاتا ہے | 0 |
|  | 333 | گشتی ماں کی گندی اولاد | 1 |
|  | 1359 | بہت اعلیٰ تحریر ہے عدالتی نظام کے منہ پر طمانچہ | 0 |
|  | 3141 | یار واقعی ماننا پڑیگا تم ایک حرامی النسل جانور کی پیداوار هو سمجه گئی مادرچود | 1 |
|  | 1508 | بول نیوز کی اینکر فضا اکبر کا بھی اکاؤنٹ بھارتیوں نے سسپینڈ کرا دیا، اور وجہ یہ ویڈیو بنی، | 0 |
|  | 1914 | اس ملک میں معززین وہ ہے جو اس ملک کو دونوں ہاتھوں سے لوٹ رہےہیں | 0 |
|  | 536 | کرپشن اس کو کہتے ہیں چُبھے کانٹا جو بھگوڑی کو تو لاڑکانہ کا سستا سا کھسرا بیتاب ہوجائے | 1 |
|  | 1297 | آرمی ہیلی پیڈ میں افسوس ناک حادثہ، افراد جاں بحق افراد زخمی، کی حالت تشویشناک ہے، | 0 |
|  | 1967 | لکھ دی لعنت ایسے حرامی اور خبیث جج پہ | 1 |
|  | 2430 | جنرل باجوہ کی توسیع سے پاک فوج کاادارہ مضبوط ہوگایاکمزور؟ | 0 |
|  | 3438 | تیری پئین دی چھولی گشتوڑ دے بچے گٹر کی پیداوار اب تیرا وقت بھی آچکا ہے تم نے افواج پر طنز کیا | 1 |
|  | 1310 | مودی کتے کی موت مرےگا انشاءاللہ مودی کے کتے دم دبا کر باگ جائے گے کشمیر سے | 0 |
|  | 2310 | جس بندے کی گانڈ اُس کا اپنا ماموں مارے اس کی ذہنی حالت ایسی ہی ہو گی | 1 |
|  | 476 | ایٹمی ملک یہ کس قسم کی باتیں کرتے ہیں انڈیا سے ہم چار جنگیں لڑچکے ھیں | 0 |
|  | 534 | ننھے صدام کی شہادت پر وزیراعظم آزادکشمیر راجہ فاروق حیدر نے گہرے رنج و غم کا اظہار کیا | 0 |
|  | 1196 | گورایا تیری ماں کو لن دینے کے لیے مجھے موقع ملا تو میں ضرور دوں گا کتی کے پتر تیری گانڈ میں نہ گولی ماری تو | 1 |
|  | 3120 | اک بات بتاؤ اگر کویی تمہاری گانڈ مار دے تو تم اسکو معاف کر دو گے اور بولو گے اور مارو | 1 |
|  | 1396 | انڈین مقبوضہ کشمیر میں انسانیت کی بڑھتی ھوئی انسانوں کے رکھوالوں کے صاف چہروں پہ طمانچہ ھے | 0 |
|  | 1492 | رزاق داود نے سی پیک منصوبے کو تباہ کرنے کی کوشش کی | 0 |
|  | 218 | اوُئے گدھے کے منہ والے میجر اپنا یہ گٹھیا بکواس بند کر ۔ | 0 |
|  | 1964 | تمہاری ہیجڑا فورس ایک نہتے کے سامنے بکری بنی ہے ڈوب مرو | 1 |
|  | 635 | کتے دیا پتر بھن چود کبھی تو پاکستان کے حق میں بول دیا کر مادر چود | 1 |
|  | 2414 | ایویں مادرچود بڈھا کھوسٹ بلاول کے لن کو منہ مین لیتا رہتا ہے | 1 |
|  | 306 | غدار کی اولاد | 1 |
|  | 332 | بکواس بند کرو تم دو ٹکے کے کنجر کیا ثبوت ہے دو ہمیں | 1 |
|  | 1945 | بھارت کے ایٹمی ہتھیار فاشسٹ مودی حکومت کے ہاتھ لگ چکےہیں، وزیراعظم | 0 |
|  | 1800 | کے الیکٹرک کا حرامی پن عروج پر۔ | 1 |
|  | 508 | کتیا یاد کر | 1 |
|  | 108 | کسی مانی ہیرا منڈی کی سستی گشتی کی اولاد لگتی ہے باپ اس کا ضرور ہیرا منڈی کا بھڑوادلال ہوگا | 1 |
|  | 1554 | بس کر چوتیے بانسوں پہ پرچم چڑھاکر ملک کے نام روشن نہیں ہوتے | 1 |
|  | 930 | زیر نظر تصویر نمبر صاف نظر آرہی ہے کہ ایڈٹ ہوئی ہے | 0 |
|  | 3209 | ارے مادرچود مرضی سے کون جاتا ہے جیل | 1 |
|  | 993 | بے غیرت سے بھی گندا لفظ ہے تو وہ ان کے لئے استعمال کریں ۔۔لعنت بے شمار ان کنجروں پر | 0 |
|  | 1841 | وڈے سائنسدان کتے دے بچے حرامی کی اولاد کشمیر کا سودا کرکے اب بکواس کررہے ہیں تمھاری اوقات حتم ہوگی | 1 |
|  | 2050 | تیرے جیسے مرضئ کتے کے بھونکنے کی کیا اوقات ہے، تیری یا تیرے آقاؤں کی بکواس ملک میں فرقہ واریت نہیں پھیلا سکتی کتے ک… | 0 |
|  | 3162 | ان کنجروں کو عقل نہی آنی یے اپنی ماں رنڈیا سے چدوا کر ہی سکون سے بیٹھیں گے | 1 |
|  | 1008 | کسی گشتی کے بچے کسے کتے کی آوارہ اولاد مادر چود کے بچے تیری گانڈ میں کیڑا | 1 |
|  | 1282 | انڈیا نے ایسی قوم کو للکارا ہے جو نہ موت سے ڈرتے ہیں اور نہ دشمن سے اور ان کی ایک خواہش ہوتی ہے کہ وطن پر جان قربان… | 0 |
|  | 2351 | جیل بھیجنا تو چھوٹی بات ھے ان بیغیرتوں نے ماڈل ٹائوں میں عورتوں کو منہ پر گولیاں مار کر مار دیا تھا | 0 |
|  | 44 | کسی کتی نسل کے ہندو کی اولاد ہو تم بے غیرت تم جیسوں کو چوک پر سنگسار کرنا چاہیے جو انڈیا کے ساتھ ہو | 1 |
|  | 1604 | اب یہ ڈھونک ختم کرو | 1 |
|  | 1155 | آج بلوچستان کے عوام نے کشمیر کے حق میں ریلیاں نکال کر انڈیا کے منہ پہ طمانچہ مارا ہے۔ | 0 |
|  | 2962 | اچھا تو تیری ماں شہباز گل کی باپ نے چودا تھا اور تیرا باوجی کی گانڈ تیرے آقا قادیانی نے ماری تھی | 1 |
|  | 2095 | بہن چود علی وزیر اور محسن داوڑ کے پروڈکشن آرڈر کیوں جاری نہیں کرتے ہوں دلال کے بچے | 1 |
|  | 3405 | نیا مادر چود کو اضافہ ہوگیا کون ہے یہ انجم کتا اس کو سرعام گولی مارنا | 1 |
|  | 3319 | لاول کے جسم کے اندر حرام کی مقدار اتنی زیادہ ھو گئی ھے کے اسے بچپن میں اپنی والدہ کے ہمراہ کوٹ کچہری اور تھانے کے | 1 |
|  | 558 | اچھی چیزیں بولے تو بیغیرتوں۔لوٹیروں۔منی لانڈروں۔چوروں کا ٹولہ | 1 |
|  | 84 | گشتی کے ناجائز بچے ہر بات میں ن لیگ پر کتے کی طرح بھونکنا ضروری ہے | 1 |
|  | 228 | مہا دلے ایسا ہی کچھ نام ہے تیرا تیری بہن کوسرعام چودون کسی کنجر کی اولاد | 1 |
|  | 2022 | کتوں اور کنجروں کی حکومت اور گشتیاں رنڈیاں وزیر پاکستان میں اس وقت کنجروں کی حکومت ہے | 1 |
|  | 2817 | ایک کتورا گورایا ہے جو ابھی تک ہتھے نہیں چڑھ رہا ہڈی ڈال کے پٹا ڈالنا ہے | 1 |
|  | 554 | یاد کرو گی امی کو جو صرف ڈانٹتی ھے ساس کے گھر کام نہیں کرو گی ساس جھاڑو لے کر تمہاری طبیعت صاف کردے گی | 0 |
|  | 2223 | ان لوگوں کو پاسپورٹ چاہیے ہوتے ہیں تو لڑکیوں سے شادی کرلیتے ہیں اور پاسپورٹ ملنے کے بعد انکی غیرت جاگ جاتی ہے بلاول | 0 |
|  | 2257 | کہاں ہے وہ بہن چود بےغیرت ہیجڑا | 1 |
|  | 3048 | کسی جاہل پٹواری کی اولاد حلالی نطفہ تیری طرح بہن چود گھٹیا نہیں | 1 |
|  | 3225 | ڈارمے کررہا ہے مادرچود | 1 |
|  | 1052 | یہ ذلت جس کو ذندگی کہتے ہیں اب ختم ہونی چاہیے | 0 |
|  | 2137 | میرے باپ نے تو تیری امی کی گانڈ بھی ماری تھی وہ تجھے یاد نہیں | 0 |
|  | 3429 | بھارت کی تتہ پانی سیکٹر کے قریب بلااشتعال فائرنگ، جوابی کارروائی میں چھ بھارتی فوجی ہلاک | 0 |
|  | 2556 | میرے چڑھے لن دے اوتے تیرا بابا | 1 |
|  | 2623 | ہم کس حق سے خود کو پہلے گواہ، پ… | 0 |
|  | 2202 | جی اور ہم نے دو دن شدید بیمار رہ کر طبیعت صاف کر لی۔ بڑے شوق اور چاؤ سے آئے تھے | 0 |
|  | 804 | تیرے جیسے اپنی بہن خود چھوڑ کر جاتے ہیں اور تاکید کرکے جاتے ہیں کہ پلیز زرا احتیاط کرنا | 1 |
|  | 616 | اگر آئین اور قانون کے مطابق جیسا کہ عمران خان کہتا ہے ادارے میں بناؤنگا ۔ ایکسٹینشن دینے سے ادارے تباہ ہوتے ہیں… | 0 |
|  | 444 | سالے ہیں پیار سے پلیز نہیں تو بہنوں سے شکائت کریں حرامی پٹواری اور اسی نالائق کی حرامی اولاد ھے جو بلکل اپنی بھگوڑی ماں پر گیا ہے | 1 |
|  | 2765 | پاک فوج نےجوابی کارروائی میں بھارتی فوج کےبنکرز بھی تباہ کیے،ڈی جی آئی ایس پی آر | 0 |
|  | 2942 | اوئے انڈین کتو گولے کیوں مارتے ہو، دم ہے تو اینٹ مار کردکھائیں پھر دیکھنا میرا کپتان اس کا جواب پتھر سے کیسے دیتا ہے | 1 |
|  | 1334 | ہولناک ویرانے اور اذیت ناک سناٹے صرف جنگلوں یا بیابانوں میں ہی نہیں کچھ انسانوں کے اندر بھی ہوتے ہیں۔ | 0 |
|  | 2716 | ثاقب صاحب حکومتی نااہلی کو بے نقاب کرنے پر آپ داد کے مستحق ہیں صیح فرمایا آپ نے ہڈ حرامی میں ہم سب شیر ہیں | 1 |
|  | 1764 | مقبوضہ کشمیر میں ھونے والی مظالم اور انسانی حقوق کے خلاف ورزیاں نام نہاد عالمی امن کی تنظیموں کے منھ پر طمانچہ ھیں… | 0 |
|  | 638 | کرپشن اور کشمیر میں سے ہماری پہلی ترجیح کونسی ہے؟ سب کو علم ہے۔ بس ہم کرپشن کو جڑ سے اکھاڑ کر دم لیں گے | 0 |
|  | 2397 | اور تم کس تربیت کا پتہ بتاتی ہو نام سیدہ والا رکھا ہے اور کوئی بھی ٹویٹ اچھی نہیں | 0 |
|  | 2245 | کوئی وجہ ہے کہ اس مادرچود کا خاندان کتے کی موت مارا گیا۔ | 1 |
|  | 750 | میڈم ریپ زدہ لاشیں ن لیگ کی دفتر سے نکلتے ہے اور زنا کار پی ٹی آئی والوں کو کیو ؟ مادرچود | 1 |
|  | 1207 | اگر بات بیغرت ہونے کی ہے تو پھر بلاول تم سے بڑا بغیرت دنیا میں نہیں ہے بلکہ بغیرت نہیں دلال ہو تم ۔ | 0 |
|  | 3044 | کبھی کوۂی مثبت اور اچھی بات پاکستان کے لۂے یہ لعنتی کردار آپ کو نہيں کہے گا | 0 |
|  | 2361 | خدابخش خروٹہ کابیٹا ذوالقرنین روڈ ایکسیڈنٹ میں جان کی بازی ھار گیا | 0 |
|  | 2638 | یا تو اپنا منہ بند رکھتے اور اگر اب منہ کھولا ھےتو ان ایم پی ایز کو نشانِ عبرت بھی بنائیں۔رپورٹ عوام نے پچھواڑےمیں لینی ہے | 0 |
|  | 2051 | تیری بہن کو تو ابھی بھی ہندو ہی چود رہے ہیں تمہاری ماں تو ہندوں لن ہی پسند کرتی ھے | 1 |
|  | 2877 | اللّه کرے ان لوگوں کی اور صحافیوں کی جو بکاؤ مال ھیں مرنا شروع ھو جائیں وہ بھی درد ناک موت تا کہ دوسروں کو نصحیت ھو | 0 |
|  | 3222 | بکواس بند کر اپنی اور گندے پانی منہ دھو جا کے | 0 |
|  | 1100 | گشتی کے بچے اسُ نے نیچے نام مریم صفدر لکھا ھے | 1 |
|  | 2249 | نیب کو اِس حرکت پر چِھتر پڑنے چاہییں۔ | 0 |
|  | 920 | چوروں اور لفافیوں کو تن کے رکھو | 0 |
|  | 802 | کمینے اور حد سے گٹھیا انسان تو چاہتا کیا ہے کہ پاکستان جنگ کر دے اور بیغرت انسان مودی حرامی کو سبق سیکھا دے | 1 |
|  | 546 | میں سندھ اسمبلی معرض وجود میں آئی اور میں اجلاس بھی ہوگیا یہ طمانچہ اپنے ہی منہ پہ مار لے چانڈیو | 0 |
|  | 1483 | یہ بہن چود کہتا تھا یہ شوبازی ہوتی ہے۔ منافق آدمی اب یہ شوبازی نہیں؟ کیا یہ وزیراعظم کا کام ہے؟ | 1 |
|  | 18 | بےغیرتی میں بلو کھسری اور حرامی پن اور چوری میں زرداری کا اپنا ایک مقام ہے | 1 |
|  | 2308 | ایران خفیہ طور پر خطرناک ترین ہتھیار کی تیاری میں مصروف، سیٹلائٹ تصاویر منظر عام پر آگئیں | 0 |
|  | 1267 | لعنت اس بیغیرت کے منہ پے، کنجروں کے پاس اختیارات ہونے کے با وجود یہ حال ہے، اس بیغیرت کو عوام کو حوصلہ دینا چاہیے | 1 |
|  | 2176 | ڈاکٹر فوزیہ نےاللہﷻ کا کتاب جلایا اللہﷻ انکو دنیا میں سب لوگوں کے سامنے نشان عبرت بنائیں فوزیہ تم انسان نہیں درندے… | 0 |
|  | 2219 | وزیراعظم آزادکشمیر کا اسمبلی اجلاس سے خطاب سن کر دل خون کے آنسو رو رہا ہے | 0 |
|  | 2055 | اس کھسری کی لگتا ھے کشمیر میں لوٹی ھوی عزت کو دوبارہ لوٹُلیا گیا ھے۔ کنجروں اس کا نہلا تو لینا تھا۔ | 1 |
|  | 2884 | دینا کے تمام مذاہب کا مطالع کر کے دیکھ لو دنیا میں اتنا گندہ اور انسانیت سوز اور کوئی مذہب نہیں | 0 |
|  | 2903 | تمام منصوبے پہلے آنے والی حکومتوں نے ہی متعارف کروائے، ماسوائے سیٹیزن پورٹل کے انہوں نے بس تختیاں ہی چ… | 0 |
|  | 3046 | تھیں اور فائدہ مند نہ تھیں۔ اور وہ یہ بھی خوب جانتے تھے کہ جو شخص ان چیزوں کا خریدار بنے گا، آخرت میں اس کا کوئی حص… | 0 |
|  | 1519 | خدا بارڈر پر نظر رکھنے والے ہر فوجی کی حفاظت فرماے اور پارلیمنٹ پر نظر رکھنے والے ہر فوجی کو کتے کی موت نصیب کرے | 1 |
|  | 2687 | بے غیرت حکومت نے آمریت قائم کی ہوئی ہے اس بے غیرت کا انجام بھی بڑا عبرت ناک ہوگا لعنت ہو بےغیرتوں پر | 0 |
|  | 1803 | کسی سرٹیفائیڈ کھوتی کے بچے عالمی عدالت میں جانے کا راستہ دکھانے والا بھی تیرا وہی باپ تھا | 1 |
|  | 824 | سر اس وقت ملک تاریخ کے نازک ترین دور سے گزر رہا ہے دشمن تیار بیٹھا ہے | 0 |
|  | 2119 | دہشت گرد انڈیا مودی کتا | 1 |
|  | 316 | اس کو کس مادرچود حرامی نے پاکستان بلوایا اس کی گ میں ڈنڈا ڈال کر اس بھی میکاسنگھ کے ساتھ انڈیا بھیج دو | 1 |
|  | 2260 | بیغرتو کچھ شرم کرو پیبسی پی کر آپی ماری یم کا خط ڈرائیور تک پہنچانا شرم کرو ڈرائیور کے ساتھ گیراج | 1 |
|  | 2862 | کتے حرامی بہن چود دھشت گرد ایک طرف ھمارے کشمیری بھاٸیوں اور بہنوں کو شہید کرواتے ھو اور اوپر سے عید | 1 |
|  | 1170 | شیح راشید اور کتے کے بھونکنے میں کوئی فرق نہی | 1 |
|  | 1141 | بلاول سکردو ميں ۔خود پی پی پی اور بی ٹيم نون ليگ پريشان جبکہ تحريک انصاف انجوائی کر رہی ہے ۔ | 0 |
|  | 1644 | آپ سے گزارش ہے کہ ہر ایکسٹنشن اچھی نہیں ہوتی | 0 |
|  | 2913 | ان کنجروں سے بس Dچوک میں مجرے کروا لو | 1 |
|  | 1955 | گھسرا گھسرے کی گانڈ مارے تو مزہ کس کو آۓ گا | 1 |
|  | 2960 | نیازی عمران تو جتنی بکواس کرتا تھا تیرے ساتھ اس سے… | 0 |
|  | 595 | اس کتی نسل کو سب پتہ ہے سودا ہو گیا ہے بند کرو یہ ڈرامے بازی کنجرو | 1 |
|  | 2167 | بھائی حرامی ہی بکواس کرتے ہیں پاک فوج کے خلاف۔حلالی نہیں | 1 |
|  | 556 | تیری بہن کو سو کتوں کا ۔۔۔ ذلیل عورت اس حرامی عمران اور بہن چود باجوہ نے کشمیر بیچ دیا ہے تیرا کیڑا | 1 |
|  | 3252 | ابے حرامی اپنی ماں کی ننگی تصاویر پھینکنے | 1 |
|  | 3277 | بکواس بند کر حرامی کتے | 1 |
|  | 2979 | یہ بےغیرت باپ کی بیٹی وینا رنجیتی تو ایک تصدیق شدہ کنجری ہے ایسے کنجروں کو ignore کرنا چاہئے | 1 |
|  | 504 | ھمیں خڑکمر میرعلی وانہ کےواقعات کے شہداء ارمان لونی شہید یاد ھے فاٹا پشتونخواہ میں ھوئے مظالم ق… | 0 |
|  | 2458 | گشتی خانے بھیجنا چاہیے | 1 |
|  | 1248 | اللہ مرحوموں کو جنت الفردوس اور لواحقین کو صبرے جمیل عطا فرماۓ۔ — feeling sad | 0 |
|  | 2552 | آوے رنڈی کے بچے عید والے دن میری زبان گندی نی کروا میں تیری بہن چود دینی ہے بھڑوے | 1 |
|  | 2708 | اب ضرورت ہےتو صرف پیر اعلی جناب کی بددعاوں ک… | 0 |
|  | 3284 | ٹھیک ہے اگر وہ گھٹیا ہیں تو آپ بھی گھٹیا رہیں۔ | 0 |
|  | 1782 | غلطی ہماری ہے،نہ تم جیسوں کوگلے لگاتے نہ آج یہ منہ دیکھنا پڑتا،یہ انڈین مہاجر اتنے حرامی ہیں | 1 |
|  | 939 | جب مسلمان کو کافر سے بھی زیادہ موت کا خوف لاحق ہو جائے تو وہ ہتھیار اٹھاتا نہیں ہتھیار ڈالتا ہے۔ | 0 |
|  | 22 | خنزیر سے بھی پلیت انسان ہے یہ نواز خبیث اور اسکی پوری پارٹی | 1 |
|  | 3472 | چل مونچوں والے کتے پاک فوج پربھونکتا را کے اہجنٹ تیری گانڈ پر گولی مارے گے | 1 |
|  | 2880 | افسوس ناک خبر سرینگر۔۔۔بھارتی فوج نے دو کشمیری صحافیوں عابد حسین اور احمد بٹ کو شہید کردیا۔ | 0 |
|  | 3110 | کشمیر جل رہا ہے اور ہمارے حکمران سو رہے ہیں آخر کب تک وہ اپنی جانیں قربان کرتےرہیں گئے | 0 |
|  | 2875 | لکھ دی لعنت ہوے تیرے اوتون | 1 |
|  | 705 | گشتی ماں کے بچے أج تو بھی باتیں کرتا ہے | 1 |
|  | 839 | یہ بزدلانہ اور بےغیرتانہ الفاظ کسے خوددار حکمران کے نہیں بلکہ زانی اور بدکردار کےہی ہوسکتےہیں | 0 |
|  | 1987 | افغان قوم کو چاہیے کہ وہ اپنے ایسے دشمنوں کو پہچانے جو بجائے اصل قصوروار کی مذمت کرے یہ پاکستان کے خلاف بکواس کر رہ… | 0 |
|  | 579 | تم گھٹیا ہو | 1 |
|  | 2379 | سر ہم ان حرام خوروں کو نہی چھوڑیں گے یے بزدل فوج ہے حملہ کرکے عورتوں کے پیچھے چھپ جاتے ہیں اب یے ایک گولی چلائیں گے… | 0 |
|  | 2216 | گشتی بھکی تےنھیں مر رھی گشتی ماں کا بچہ | 1 |
|  | 2977 | کسی کشتی دیے زبان کو لگام دو | 0 |
|  | 2395 | اور دہشتگرد بھی ہم… | 0 |
|  | 623 | بےغیرتوں کا تخم تو خود ہے بلاول ذوالفقار علی بھٹو کے نام پر کلنک | 0 |
|  | 3093 | تاریخ کا وہ واحد حکمران جس کے اقدامات پر لوگ تنقید نہیں کرتے بلکہ اس کا پرانا کلپ دکھا کر ذلیل کرتے ہیں | 0 |
|  | 903 | کیا گانڈ ماری ہے خان صاحب نے علی زیدی کی | 1 |
|  | 1234 | لگتا ھے آج کتی کا پٹہ کھول دیا ھے کسی نہیں بہن چود بھونکے جا رھی ھے رک جا کنجری کتیا | 1 |
|  | 1565 | کوئی انتہائی گھٹیا عورت ہے یہ۔ | 1 |
|  | 256 | گزرا سال وطن عزیز اور پاکستانی عوام عزت کا ایک سال تھا ساری دنیا میں پاکستان کی کھوئی ہوئی عزت بحال ہوئی ہے | 0 |
|  | 3426 | بھارتی حکومت کان کھول کر سن لے | 0 |
|  | 2174 | مولانا فضل الرحمن اگر تو بھول کر کشمیر کے بارے میں کوئی بکواس کی اور ازادکشمیر داخل بھی ہوا نہ تو اتنے تمارے اگے پ… | 0 |
|  | 1158 | تبدیلی کے فقط ایک سال نے ہمیں مالدیپ، نیپال اور بھوٹان سے بھی نیچے لا پھینکا | 0 |
|  | 1912 | سال سے تم نہیں بھونکے اب اپنی سیاست چمکانے کے لیے حکومت پر حملے کر رہے ہو کتے کے بھوکنے اور تمھارے بھوکنے میں کوئ فرق نہیں | 1 |
|  | 1811 | فیصلہ کر لیا ہے کہ پانچوں میں س… | 0 |
|  | 1739 | برکھا دت شائد نہیں جانتی کہ خان جو بولتا ہے وہ کرکے دکھاتا ہے اور جب مارتا ہے تو رونے نہیں دیتا | 0 |
|  | 2635 | یعنی آپ سمجھتی ہیں کہ یہ کام بھی عمران نہیں کر رہا۔۔۔مہنگائی بڑھ گئی اسٹاک ایکسچینج تباہ۔۔نہ گھر نہ نوکری نہ گورنر… | 0 |
|  | 78 | تمھاری قسمت میں اب صرف اور صرف بکواس کرنا رہ گیا ہے اب تو لگتا ہے تم پاکستان مخالف قوتوں کے ہاتھوں بک گئے ہو | 0 |
|  | 2858 | کشمیر آزاد کروانے کے لیے اس کی بیٹی کو چوک میں چودا جائے ۔سالا حرامی بہن چود ۔شامی کتا سور | 1 |
|  | 957 | نہ دشمن کے دانت کھٹے کیئے نہ کشمیر کو پاکستان بنایا ۔۔۔ ہنہہ | 0 |
|  | 1589 | انڈین فوج کے ظلم کی وجہ سے کشمیر میں بیس دن سے سکول بند پڑے ھیں۔ | 0 |
|  | 3172 | اللہ کرے یہ اچهے صحافی جلد مر جاہیں تاکہ جلد جنت میں جاہیں اس سے پہلے کہ یہ حرامی ھو جایں | 1 |
|  | 110 | روپیہ کی چوری پر برہنہ اور تشدد کرکے قتل کرنے والوں کو شرم تک نہیں آئی ، خدا کا عذاب نہیں ٹوٹا ان پر | 0 |
|  | 2706 | گشتی بہن کے دلے | 1 |
|  | 2460 | جس انسان کی زندگی دلالی اور PC ہوٹل میں دگڑ دلّا بن کے گُزری ہو اُس مادر چود کو ہاشوانی کنجر کہیں | 1 |
|  | 2592 | کسی گشتی ماں کے بچے جو بھی ہیں لیکن یہ دیکھ ہر بندہ لڑنے کے لیے تیار ہے تو بس تیاری رکھ تیرا سر تن سے جدا ہونے والا ہے۔۔ | 1 |
|  | 1628 | جتنا بڑا چیلنج خان صاحب نے ان کو دیا، واقعی اگر کام کرنے پہ آتے تو راتوں کی نیند حرام ہو جانی تھی۔ | 0 |
|  | 2080 | تم سب لوڑے اس وقت جمہوریت کی گانڈ میں سوئے مرے تھے جب ماں ئولنا ڈیزل کشمیر کمیٹی کا چئیرمین تھا | 1 |
|  | 883 | اوئے گشتی کے بچے کتی کے بچے اپنے امی کے یاروں کو خوش کر رہا | 1 |
|  | 590 | گٹر نیوز کے دفتر کو آگ لگائی جاے مادرچود کو | 1 |
|  | 2973 | خان صاحب کب تک جھوٹ بولو گے کیوں اپنے پیشے سے غداری کر رھے | 0 |
|  | 72 | لعنت تیرے ماں باپ پر جو تجھ جیسے حرامی غلام پیدا ہواں ہے ان سے | 1 |
|  | 2505 | پاک فوج نے بھارتی فورسز کے بنکرز بھی تباہ کیے،ڈی جی آئی ایس پی آر | 0 |
|  | 1865 | انسانی جان کا کوئی مول نہیں | 0 |
|  | 193 | السلام و علیکم ہماری ایک اردو پوائنٹ کی میڈیا اینکر ھے جس کا نام کنول آفتاب ھے اتنی گھٹیا اور گندی لڑکی ھے | 1 |
|  | 827 | واقعی نہ چاہتے ہوۓ بھی سچ تمھارے منہ سے نکل گیا اب زرا آنکھیں کھول کر دماغ کو کھول کر دیکھو | 0 |
|  | 3353 | پھر کہتے ہیں ہمیں نمک حرام کیوں کہتے ہو ہماری ہڈی کھا کر ہمیں ہی کاٹتے ہو شکل دیکھو اس حرامی کی شکل دیکھو منحوس | 1 |
|  | 2743 | اوئے گانڈو کے لوڑے | 1 |
|  | 1309 | مراد سعید تو حرامی ہے اور تیرا کتا باپ عمرو نیازی مادرچود ہے تم دونوں گانڈو واشل ہو انڈیا نے تم سب | 1 |
|  | 1252 | اوئے نمک حرام دلے پاک بھارت مزکرات میں سب سے بڑی رکاوٹ ھیں اس نمک حرام کا کچھ نہ کچھ کرنا چاھیے | 1 |
|  | 263 | کسی نے آپ کے گھر والوں کو یرغمال بنایا ہوا ہو اور ان کی عزت لوٹ رہا ہو | 0 |
|  | 3399 | ارب ڈالرز کی بکواس تمھارے لُچے لیڈر کی اس کُڑی کے خسم کے پیو اسحاق ڈار نے کی تھی، جو کا… | 0 |
|  | 2039 | چلیں جی شروع کرتے ہیں یہ ٹرینڈ اور پٹواریوں اور پیپلیوں کی بکواس کا جواب دیتے ہیں | 0 |
|  | 1665 | علی زیدی صاحب اور سندھ حکومت بتاؤ ان کی موت کا ذمہ دار کون ہے | 0 |
|  | 973 | تیری گانڈ مارے بندہ اور رونے بھی نہ دے | 0 |
|  | 2346 | پاکستانی فوج نے بھارتی فوجی چوکی کو کیسے اڑا دیا | 0 |
|  | 1258 | تو کتی ھے گشتی ھے خود تو گندے کام چھوڑ دیں | 1 |
|  | 852 | سمجھا ہی رہی تھی مگر یہ بکواس پر اتر آئے اور مجھے بکواس سننے کی عادت نہیں ھے چاھے سبھی مجھے انفالو کردیں | 0 |
|  | 555 | آج انڈیا کی ریاست ناگا لینڈ نے اپنا یوم آزادی پاکستان کے ساتھ منا کے انڈیا کے منہ پہ ذبردست طمانچہ رسید کیا ھے | 0 |
|  | 163 | ساحر کی گشتی کی جلن ہم سمجھ سکتے ھیں | 1 |
|  | 1371 | پاک فوج نےبھارتی چوکیوں کونشانہ بنایا،ڈی جی آئی ایس پی آر | 0 |
|  | 1942 | ایک نام نہاد جعلی سردار نے آج پریس کانفرنس میں دھمکی آمیز الفاظ کا استعمال کیا جسکی میں مذمت کرتا ہوں | 0 |
|  | 2130 | ان کنجروں کی بنڈ لال رہنی چاہیئے | 0 |
|  | 289 | ممے | 1 |
|  | 1908 | اس گھٹیا ترین زہنیت کی بس مزمت ہی کی جا سکتی ہے | 0 |
|  | 3186 | شوہر لڑکی کی عزت لوٹ رھا اس دوران بیوی اس منظر کی ویڈیو بناتی رہی۔فلم کا عنوان تھا کشمیر بنے گا پاکستان | 0 |
|  | 3036 | کنجری اپنے پیدا کرنے والےمہا کنجروں کے ساتھ | 1 |
|  | 2038 | کوئی بھی منہ اُٹھا کر کسی شریف عورت کو ہیرس کرنا شروع کر دے | 0 |
|  | 2692 | بھارتی میڈیا نے پاکستانی راکٹ حملوں میں تباہ شدہ بھارتی ٹینکرز اور چوکیوں کی تصویریں جاری کر دی ۔ | 0 |
|  | 1943 | تیری پین دس کُوسہ ماریا کسی گشتنی نسل دیا پینڑ لنا بے غیرتا۔ | 1 |
|  | 1340 | کیا بکواس ہے ری ٹویٹ کے ڈر سے انڈیا کشمیر چھوڑ کر بھاگ جائے گا | 0 |
|  | 853 | شکل سے تو تمہاری نسل کتے کی لگ رہی ہے تم جیسے بےحس کنجروں کی وجہ سے ملک کو تباہی کا سامنا ہے | 1 |
|  | 1336 | ہمارے پاکستانی جوان یہ یقین رکھتے ہیں کے ہم اپنے وطن کی خاطر جان دیں گے تو بھی ہم زندہ رہیں گے | 0 |
|  | 3157 | بولتی بند فیس بک کے زبان درازوں کی زبان سیاستدانوں کے سامنے ان کے دانتوں کے پیچھے کیوں چھپ جاتی ہے | 0 |
|  | 2005 | بہن چود کُتّے کے پُتر کھوتے کی نسل | 1 |
|  | 3130 | یہ سارے بےغیرت تو حکمران نہیں بلکہ چور ڈاکو تھے۔ لوٹ مار کررھےتھے۔ | 1 |
|  | 2861 | درفٹے منہ | 0 |
|  | 3357 | بے غیرتی میں بلاول کھسری اور حرامی پن اور چوری میں زرداری کا اپنا ایک مقام ہے | 1 |
|  | 1189 | میری بند آںکھیں بھی تم پڑھ لیتے ہو مجھ کو اتنا جان چکے ھو تم بھی نا | 0 |
|  | 1214 | اوہ کسی کھوتی کے بچو بنا بنایا پاکستان تم کو دیا اور تم بیغرتوں نے اسکا برا حال کر دیا ۔۔ | 0 |
|  | 2853 | اس بزرگ کی طرف دیکھ کے کوئی رحم کھاو کشمیر پر او بیغرت حکمران تیرے شکل پر لعنت جس جس نے بھی اس بیغرت کو ووٹ دیا | 0 |
|  | 2254 | جب مقبوضہ کشمیر میں مسلمان ہی زندہ نہیں بچیں گے تو انڈیا سے خالی کشمیر لے کر وہاں ہم اپنا قبرستان بنائیں گے کیا | 0 |
|  | 567 | دنیا گول نہیں مادرچود ہے | 1 |
|  | 2144 | صحافی اور پشتون تحفظ موومنٹ کے حمایتی خلیل خان محسود کو دن دھاڑے شہید کر دیا گیا اللہ… | 0 |
|  | 553 | زبردست جواب پاک فوج کا | 0 |
|  | 843 | مادرچود صحافیوں کے نام پہ دھبہ ہے تو | 1 |
|  | 1060 | کمینا | 1 |
|  | 2029 | سچے اور اچھے دوست دوستوں کو زلیل کرنے کا موقع جانے نہیں دیتے | 0 |
|  | 907 | بے غیرت انسان کاش تیری ماں بہن اور بیٹی ہوتی کشمیر میں، پھر میں دیکھتا کہ تو کیسے ٹوئٹر پر آنی ماں چُدوا | 1 |
|  | 514 | انسان کو اپنے اوپر غرور اور تکبر نہیں کرنا چاہیے الله کی لاٹھی بڑی بے آواز ہے | 0 |
|  | 3231 | او حرامی نطفے قادیانی ہے تو اور قادیانیوں کو کوئی حق نہیں مسلمانوں کے… | 1 |
|  | 1735 | اور یاد رکھیے کشمیر پر پانچ ٹوئٹ کرنے سے ہی انڈیا کا ایک فوجی مرے گاتین کیے تو فوجی صرف لنگڑا ہوگا مرے گا نہیں | 0 |
|  | 2158 | اس کتے کے بچے کھسری کو گولی مار دینی چاہئے | 1 |
|  | 2490 | پنڈی کی ٹاپ کی رنڈی سے شادی کی ہے مادرچود کشمیر کا اور سینٹ کے معاملے کا آپس میں کیاتعلق | 1 |
|  | 2469 | حسین نواز پاکستان کے عوام آپ کے گانڈ مارے گا | 1 |
|  | 759 | ڈنگر دے بچے تو انسان ہے ہی نہی خبیث | 1 |
|  | 1364 | کراچی کا بیڑا غرق کر کے رکھ دیا پی پی پی نے شہر کو کچرے کے جوھڑ میں تبدیل کر دیا نا خود کام۔کیا نا سٹی گورنمنٹ کو کرنے دیا | 0 |
|  | 1291 | کسی بدکردار ماں باپ کے بچے نیازی کی واشل بکواس بند کرو | 1 |
|  | 188 | نون لیگ کی چوچل میڈیا شوشل میڈیا وچل میڈیا چوکل میڈیا میں جو پھڈے کروا رہا امریکہ کروا رہا ۔ | 0 |
|  | 1707 | پاکستان کا خزانہ لوٹنے والے کرپٹ کنجروں کے علاوہ تمام پاکستانیوں کو عید مبارک | 1 |
|  | 847 | پاک فوج کی بھرپورجوابی کارروائی،افسرسمیت بھارتی فوجی ہلاک،متعددزخمی | 0 |
|  | 690 | بی آر ٹی اس انصاف کی آئینہ دار ھے | 0 |
|  | 640 | آرے بھگوڑی تو کل تک تو بیمار تھی ،،، کسی نے تیری بجا دی تھی آگے اور پیچھے سے ، اور تو آج پھر آ گئی بجوانے | 1 |
|  | 94 | گشتی حرام کی کتیا عورت | 1 |
|  | 2251 | اللہ کرے یہ اچهے صحافی جلد مر جاہیں تاکہ جلد جنت میں جاہیں اس سے پہلے کہ یہ حرامی ھو جایں | 1 |
|  | 1842 | تو بیغیرت ہے مادرچود دلے کنجر کے بچے تم سب چور ہو گشتی کے بچو گندی نالی کے کیڑوں حرام خوروں | 1 |
|  | 3340 | افغان طالبان امریکہ سے مذاکرات تب تک روک دیں جب تک مسئلہ کشمیر حل نہی ہوتا۔ | 0 |
|  | 1738 | کتے کے پتر نیب پہلے دو دفعہ بلا چکی تھی اور بھگوڑی نے کہا تھا میں نہیں پیش ہوں گی کر لو جو کرنا ہے | 1 |
|  | 2302 | کتوں اور کنجروں کی حکومت اور گشتیاں رنڈیاں وزیر | 1 |
|  | 2015 | حاکمِ وقت تیری مردہ ضمیری پر لعنت | 1 |
|  | 1897 | تیری ماں کے یار جہانگیر ترین نے جو قرضہ لیا تھا وہ تیری بہن کے مجرے پر لٹا گیا تھا؟؟ خبیث انسان | 1 |
|  | 520 | مسئلہ کشمیر پر مسلم امہ کی خاموشی قابل افسوس ہے ،امارات مودی کو اعلی ترین سول ایوارڈ دینے جا رہا ہے | 0 |
|  | 1847 | شاہیں،غوری،ابدالی،اللہ کے کرم کے بعد دفعائی بجٹ کا کمال ہے،اللہ کا شکر ادا کریں،ا… | 0 |
|  | 822 | علامہ خادم حسین رضوی نے ہمیشہ کفر کو للکارا ہے ۔دھرنا حضور ﷺ کی ناموس کی خاطر دیا اور فتویٰ گستاخ گیرٹ ویلڈر کےقتل… | 0 |
|  | 3387 | آپ ان کے خون کے ایک قطرے کا بھی قرض نہیں چکا سکتے | 0 |
|  | 582 | کدھر گئے سندھ کے بے غیرت حکمران ان بےغیرت حکمرانوں کو نشان عبرت بنا دے آمین | 0 |
|  | 898 | یہ انڈیا کے لیے کم تھا کہ آصف غفور کی پاکستانی سوشل میڈیا فوج بھی جب انڈیا کے خل… | 0 |
|  | 3131 | او کنجرا بات خوشی کی نہیں تم جیسے لنڈورے کیلیئے ڈوب مرنے کی ھے… | 1 |
|  | 436 | یہ ساری گالیاں نواز شریف کو لگ رہی ہیں کیونکہ سودا اُسی حرامی نے کیا ہے، زرداری کے ساتھ ملُکے | 0 |
|  | 2075 | وڈا میجر سالا بندہ نہ زنانی نری کتے خانی | 1 |
|  | 2349 | کتے نسل کا حجڑہ کیا کہہ رہا ہے۔بےغیرت تو تم ہو | 1 |
|  | 2241 | حرام زادے بڈھے تیری بیوی اور بیٹیوں کو اٹھا کر کشمیر کے پہاڑوں میں لے جائیں گے بہن چود | 1 |
|  | 2043 | جب بھی ہم تاریخ کا مطالعہ کرتے ہیں تو ایک بات واضع طورپرملتی ہے کے آج تک جس میں بھی معصوم لوگوں پہ ظلم کیا وہ نشان… | 0 |
|  | 576 | کتے کے پتر حامد میر جعفر | 1 |
|  | 187 | ہمارے ولی کو شہادت نصیب ہوئی جھوٹی گواہیوں پہ پھانسی دی گئی | 0 |
|  | 1378 | حافظ سیعد صاحب کو رہا کر کے بھارت کے منہ پر ہلکا سا طمانچہ تو مار دو | 0 |
|  | 59 | کنجر خان سب سے بڑا چور ہے | 1 |
|  | 55 | کسی ملک میں کسی خبیث صفت انسان کے ہاتھ طاقت کا آنا شائد اجتماعی گناہ کی سزا ہے | 1 |
|  | 983 | یہ سب بے غیرت لوگ ہیں ان کو صرف اپنی سیاست بچانی ہے بس چور سب مادرچود ان کو کوئی فکر نہیں ہے مسلمانوں | 1 |
|  | 1975 | دو گدھے بھی پاکستان آتے اگر پاکستان سے محبت ہے ان حرامخوروں کو پاکستان سے لوٹ مار کرتے ہیں | 0 |
|  | 2613 | اور میر جعفر جیسے لوگوں کو بھی زلیل و رسوا کر اور پاکستان کو انسے نجات عطا فرما آمین | 0 |
|  | 1859 | اوے کسی آوارہ گشتی کی نسل،تیری ماں نے تجھے یہی دکھایا ہے | 1 |
|  | 272 | آج کا دن ان لوگوں کے منہ پر طمانچہ ہے جو کہتے تھے پاکستان تو ایک سال نہ رہے گا واپس ہمارے پاس آئے گا | 0 |
|  | 1195 | کر دیے گئے اطلاع کے مطابق بھارتی فوجی چیک پوسٹیں چوڑ کر | 0 |
|  | 1050 | کنجر | 1 |
|  | 2988 | تیری گندی نالی کے اندر میں ڈانگ پھیر کر ڈکا کھولتا ہوں جراثیم والے گٹر کی پیداوار | 1 |
|  | 733 | اسلام علیکم دوستوآپ لوگ دشمن کیخلاف جہادکررہےہوکلمہ حق لکھ کردشمن کومنہ توڑجواب دےکراسکی صفوں میں ہلچل مچادی آپ لوگ… | 0 |
|  | 1521 | اف خدایا ۔۔۔۔ دل خون کے آنسو روتا ہے ۔۔۔ وہ اپنے حق کے لیے لڑتے لوگ وہ کافروں کے ہاتھوں مرتے لوگ۔ وہ خون کے آنسو بلکتے لوگ خون کے آنسو رو لاتے ہیں | 0 |
|  | 121 | مرو گے حرامی بچوں | 1 |
|  | 1456 | سودا تو تم لوگ کرتے ہو بے غیرتوں کنجروں دَلیوں حرامیوں تمہاری شکل پر لعنت پڑی ہوئئبے | 1 |
|  | 985 | کتے کیبھونکی جا… | 1 |
|  | 627 | ان کنجروں سے بس Dچوک میں مجرے کروا لو بس بزدل کنجر | 1 |
|  | 2044 | عمران گھٹیا ہے اس کی ساری کابینہ بھی گھٹیا ہے نفرت کے بھی لائق نہیں تم لوگ | 0 |
|  | 3212 | اوئے بہن چود انڈیا ھی چلے جاو کسی گشتی کے بچے | 1 |
|  | 978 | ارشاد بھٹی وہ سیانا کوا ہے جو ہر ٹاک شو میں نون لیگ کے خلاف بکواس بھی کرتا ہے اور پھر ریٹ بڑھانے کے لئے سلیکٹڈ حکوم… | 0 |
|  | 3312 | زاکر کنجروں پہ لعنت اپ غم منایا کرو آپ کے عقیدے میں عید منانا حرام ہے | 1 |
|  | 1134 | با با رحمتا نہ ہوتا تو آج کھا پی کے بیچ کے جا چکے ہوتے خیر آپ کی اپنی سوچ ہے | 0 |
|  | 2714 | دیکھ لے مودی تونے کس قوم کو للکارا ہے | 0 |
|  | 2521 | یقینا اس خبر سے پٹواریوں کی تو نیند حرام ہو گئی ہوگی انہیں دوائی کی ضرورت ہے یہ لیجئے اپنی دوا… | 0 |
|  | 1118 | کیسے بیغیرت پٹواری ہیں اپنی لیڈر پے ہی حوس پوری کر رہے ہیں افسوس | 0 |
|  | 1860 | مریم کی گانڈ آج رات خان مارے گا پھر توں ماموں بنے گا اور ناچے گا | 1 |
|  | 1544 | گھٹیا اور زلیل شخص جب کچھ نہ کر سکے تو وہ زاتیات پر حملہ کرتا ہے | 0 |
|  | 2285 | پکڑ کر اس کی گانڈ مارے نیب صرف گانڈ پھاڑے مار مار کر اور مریم صفدر کی چیخیں نکلوائے | 1 |
|  | 2923 | لگتا ہے حرامی ہندووں تم اپنے باپ ٹیپو سلطان کو بھول چکے ہو جس کے آنے سے تمہاری ماوں کے بچے گر جایا کرتے تھے | 1 |
|  | 2619 | زرداری صاحب ان کی گانڈ مارے گا تھوڑا صبر کر جاؤ | 1 |
|  | 1775 | اوئے مریم کے گمشدہ کتورے ،اوہ بیغیرتوں کا اک پورا میگزین، شکل دیکھ اپنی آئینے | 1 |
|  | 1191 | سب ڈرامےبازی ھےکشمیرکامکمل سوداکردیاگیاھے کچھ دن کےبعد سب بھول جاۓگےکشمیرمیں کیاھورہاھےدن سےوہاں پرکرفیوھےاقوم مت… | 0 |
|  | 1850 | نواز شریف نے ایک خفیہ ہسپتال قائم کر رکھا ہے جہاں ن لیگ میں شامل ہونے کے بعد پٹواری کی عقل والی رگ کاٹ دی جاتی ہے | 0 |
|  | 1617 | جہاں اسمبلیوں میں تیرے جیسے بھونکو ہوں ایسی اسمبلیوں کو آگ لگا دینی چاہیئے | 0 |
|  | 537 | نواز شریف اور مریم بھگوڑی کو پھانسی | 1 |
|  | 3349 | حرام زادوں اگر نواز کی دور اندیشی کو سمجھ جاتے تو اج اتنا زلیل نا ھوتے تمھاری دنیا میں بھی عزت ھوتی | 1 |
|  | 1393 | یہ منظر موجود حکومت کے منہ پر طمانچہ ہے ۔ | 0 |
|  | 313 | اپنی باری کا اِنتظار ضرور کیجئے گا، | 0 |
|  | 1488 | مریم کی گانڈ میں مرچیں دیں گے آج رات اور پھر وہ بتا دے گی کے۔اسے قطری شہزادے نے چار کروڑ | 1 |
|  | 1236 | گٹر نیوز کے دفتر کو آگ لگائی جاے مادرچود کو | 1 |
|  | 1756 | سنی تحریک کے مولویو داڑھی سنت کےمطابق رکھی ہے تواپنے اعمال و افعال کو بھی سنت کےمطابق ڈھالو۔ | 0 |
|  | 1722 | ہیرا منڈی سے بھاگی ھوئی وزارت کے مزے لے رھی ھے | 1 |
|  | 3444 | اہو اب بہت ساروں کی گانڈ میں چالیس ملک امت امت والا کیڑا سٹ مارے گا | 1 |
|  | 2348 | یہ کچھ بھی نہیں کرسکتا سوائے بکواس کے | 0 |
|  | 2274 | اگر ابو نہ بچاتا تو لونڈے کے ساتھ جو بازی ہونی تھی اس نے تو اِک وزیر کے رکارڈ بھی توڑ دینے تھے | 0 |
|  | 2401 | فصلیں تباہ ہوئیں، مویشی پیاسے مرے، ایک ایک اجتماع میں چالیس چالیس رضاکار مارے گئے، بمباریاں ہوتی رہیں، اسکول اور دف… | 0 |
|  | 890 | پاک فوج نے بھارتی چوکیوں کو نشانہ بنایا۔ کارروائی میں دو بھارتی بنکرز بھی تباہ کر دیے گئے | 0 |
|  | 189 | بے غیرت تم اور تمہارا باپ | 1 |
|  | 1201 | ذلیل آدمی اپنی بکواس بند کر | 1 |
|  | 1896 | بھارت کی جانب سے امن کو تباہ کرنے ایک اور کوشش۔۔۔ | 0 |
|  | 1618 | اب بکواس بند کردو اگر فوجی نقل و حرکت رپورٹ ہی کرنی ہے تو ہندوستانی کرو بہن چود | 1 |
|  | 477 | گوریلوں کا انڈین کا نوائے پر حملہ؟ بہت ساری ہلاکتیں فوجی جھڑپ میں بھارتی چیک پوسٹیں تباہ ہوئیں؟ اسلحے کے ذخائر بھ… | 0 |
|  | 2000 | ویسے جو بھی بہن چود یہ ٹرینڈ چلا رہے ہیں کتی کہ بچے کیا نواز زرداری نے کشمیر فتح کر کہ رکھا تھا تبدیلی بیچ آئی کشمیر کو | 1 |
|  | 544 | دریائے سندھ میں بھارت کی طرف چھوڑے گئے پانی کی وجہ سیلاب کی خبر ہے | 0 |
|  | 2785 | عرب اپنی عیاشیوں، ناانصافیوں اور بے اصولیوں کی وجہ سے رب تعالی کے عذاب کے حقدار ٹھریں گے۔ | 0 |
|  | 2429 | پاکستان میں بے غیرتوں کی ایک خاص قِسم پائی جاتی ہے جو ہر سال کی طرح اِس سال بھی یومِ آزادی کا بائیکاٹ کر کے بیٹھے ہے | 0 |
|  | 1992 | میں اسکو اسی لئے دجالی میڈیا لکھتا ہوں، یہ کبھی ایسی خبریں سامنے نہیں لاتے، پیسہ پھینکا جاتا ہے انکے منہ بند ہیں | 0 |
|  | 680 | پاکستان کی سلامتی کونسل میں کامیابی بھارت کے منہ پر مودی کے منہ پر ایسا طمانچہ مارا ہے کہ حوس اڑ گیا ہے | 0 |
|  | 3475 | ایے کتی بس کر دو اب جتنی گالیاں تمہیں ٹویٹر پر پڑتی ہے یہ تمھارے غلط ہونے کا منہ بولتا ثبوت ہے | 1 |
|  | 1094 | جی نواز شرہف نے جناب کو ایجنسی سے اٹھواکر ٹھکائ لگوائ تھی لہذا نواز شریف کا احسان یاد ھے۔ سیٹھی صاحب۔کیا آپ نے عمرا… | 0 |
|  | 1991 | سب سے بڑے مادرچود ہیں یہ دونوں | 1 |
|  | 1204 | مادرچود ایسے بول رہا ہے جیسے نیچے بہت تکلیف ہے تھوووو کتے تجھ پر امریکہ سے سودا منہگا کر کے آۓ ہو | 1 |
|  | 474 | پاکستانیوں ذرا ہوش کے ناخن لو اور حقیقت جان کر جیو | 0 |
|  | 253 | چل جھوٹے خبیث بے شرم انسان | 1 |
|  | 2761 | اوئے دلیا اوئے کتیا اوئے خنزیرا اوئے سئورا او تیری ماں نوں لن | 1 |
|  | 2847 | آس غریب عوام کا پیسہ لوٹ کر باہر کے ممالک میں پراپرٹیاں بنائی گئی اللہ ان کو نشان عبرت بنا امین | 0 |
|  | 1587 | تیرے جیسوں کی بولتی بند رہے گی | 0 |
|  | 1489 | تم بھی تیاری کرو جیل جانے کی بکواس بند ہو جائے گی ساری | 0 |
|  | 312 | آج کے درد ناک واقعی پر انتہائ دکھ ہوا۔اللہ تعالی سے دعا ہیں کہ ان چاروں نوجوانوں کو جنت میں اعلی مقام دے | 0 |
|  | 1719 | جواب تو ہے ہی نہیں سوال کرنا ویسے بھی بری بات ہے چل کنجروں کو ڈیفنڈ کرو | 1 |
|  | 2995 | کتنے کھوتی کے بچے ہیں ذرا شرم نہیں آ رہی الٹا چوروں کو ڈیفنڈ کر رہے | 1 |
|  | 3002 | جو نہیں جانتا اور نہیں جانتا کہ وہ نہیں جانتا وہ بیوقوف ہےاس سے بچ | 0 |
|  | 133 | بیغیرتوں کنجروں کوئی ثبوت ہے نہیں بس بکواس پر بکواس کرتے ہو | 1 |
|  | 2795 | بیغیرتوں کا خون ہے بیغر ت ہی ہوگا | 1 |
|  | 3028 | مراد علی شاہ اس رنڈوے بے غیرت ہجڑے کو مریم کی گود سے نکالو اور شہر کی صفائی پر لگاؤ حرامی کو | 1 |
|  | 915 | مجھے کوئی بتا سکتا ہے آخر کشمیری ماوں بہنوں کیا قصور ہے ان کو دن سے ان کو بھوکا پیاسا رکھا ہوا ہے | 0 |
|  | 722 | اچھا تو آپ سالا صاحب ھیں ظاھر ھے بہن دی ھے تو بہنوئی کی فیور بھی کرو گے | 1 |
|  | 1097 | کتا حرامی کافر قادیانی کافر ہے تھا رہے گا کتا کہاں آیا بتاو اسے واصل جہنم کریں | 1 |
|  | 3310 | پاک آرمی نے متعدد بھارتی چیک پوسٹیں تباہ کر دی دیکھیے خصوصی ڈرون سے بنی ویڈیوز مگر ریٹوئیٹ مت کرنا کہیں مودی دیکھ ن… | 0 |
|  | 2889 | تم خوبصورت لڑکی پانے کیلئے دارھی کاٹ دیتے ہو | 0 |
|  | 2440 | حرامی پن کی انتہا | 1 |
|  | 2667 | اس کھوتی کے بچے کودیکھو اسی لیے تو ہم پٹواری عقل سے فارغ کھوتےکہتےہیں انکو، بھئی عمران خان وزیراعظم ہے | 1 |
|  | 1524 | جب تک ان جیسے نمک حرام لوگوں کو پھانسی نہیں ملے گی تب تک روز نئے نئے كنجر اُٹھیں گے۔ | 1 |
|  | 86 | اس کنجر انسان کو کھسرے کو کوئی روکنے والا نہیں ہے | 0 |
|  | 2007 | ان سب حرام خوروں سے درخواست ہے کہ مجھے یہ سمجھا دیں کہ ارب ڈالر کس طرح سے بنتا ہے | 1 |
|  | 1197 | احساس کریں بکواس نہ کریں | 0 |
|  | 2762 | لٹکاؤ اس مادرچود کو ڈٹ جا اور کھڑا کر کے | 1 |
|  | 2731 | کھاتے کراچی کا ہو بھوکتے زرداری کی طرح او نمک حرام بنارسی ٹھگ | 0 |
|  | 575 | بےغیرت بلاول زنخا اسمبلی میں اُچھل اُچھل کر بھونکتا ہے | 1 |
|  | 2303 | لخ لعنت تم لوگوں پر۔۔۔کنجروں یہ اوپن ایریا ہےیہاں پانی کھڑا ہو ہی نہیں سکتا۔۔ذرا اندرون کراچی دکھاؤ | 1 |
|  | 2854 | دل خون کے آنسو روتا ھے یہ دیکھ کے | 0 |
|  | 1592 | سیدھی طرح بکواس کر نا جو بھی ہے کا کیا مطلب اپنے باپ کا نام بتا دنیا پاکستانی عوام کوبھی پتا چلے ان کا خیر خواہ کون ہے | 0 |
|  | 513 | جھیل نمکین نہیں تو نمک حرامی کا سوال ہی نہیں، ہاں پورا اندرونِ سندھ کراچی ضرور پال رہا ہے، تو نمک حرام تو تم ہوئے | 1 |
|  | 3396 | کسے گندے گٹر کے چوہے | 1 |
|  | 2911 | آپ منہ بند کروانے کا گِلہ کر رہے قوم تو اِن چوروں کے بند ہونے کا انتظار کر رہی | 0 |
|  | 831 | بوببز | 1 |
|  | 505 | اففف اللہ یہ کیسا انسانیت ھے یہ کونسی معاشرے میں ہم رہ رھے ہیں ہمارا معاشرہ کس طرف جارہا ہے | 0 |
|  | 1095 | کوہی کتے کا بچہ ہی ہوگا مادرچود فنکشن ارینج کروانے والا گولی مر دینی چاہے بھارتی فنکار کو بھی اور جو | 1 |
|  | 2155 | اس بکواس شعر میں شاعر دوبارہ کشمیر کمیٹی کا چیئرمین بننے کی درخواست کر کے کہہ رہا ہے کہ ہم وطنوں، اب مزید اپنے پیسو… | 0 |
|  | 2715 | کاش ذلت کا بھی کوئی معیار اور حد ہوتی تو ہم اس شخص سے کہہ سکتے کے تم اتنے ذلیل ہو | 0 |
|  | 1868 | صحافت کا کاروبار لفافہ صحافیوں کی بدولت کنجروں کی رہائش گاہ بن چکا ہے | 1 |
|  | 631 | تحریک انصاف کے تمام کنجروں کو عید مبارک ️ | 1 |
|  | 607 | مانا کہ کشمیری عوام کمزور ھے، لیکن عنقریب ظالم عبرت کا نشان بنیں گے | 0 |
|  | 3097 | مودی خنزیر تو کس کھیت کی مولی ہیں تیرے جیسے بڑے آئے اور مر گئے تجھ کو تو ہم چیٹی کی طرح مسل دیں گے پہ… | 0 |
|  | 2965 | یہ اتنے حرامی ہیں کہ اپنی حرامزدگی کو جسٹیفائی کرنے کے لیے کسی بھی حد تک جاسکتے ہیں | 1 |
|  | 2771 | سسیلین مافیا پہ جج ارشد ملک کی مبینہ وڈیو کا سارا معاملہ الٹ پڑ گیا ۔ شہبازشریف نے مریم پہ اور مریم نے سارا معاملہ… | 0 |
|  | 1669 | بہن نہیں، بیٹی نہیں ماں تو ہو گی۔ کھلی چھٹی ہے گٹر کی پیداوار جا کر ماں سے انجوائے کر | 1 |
|  | 2767 | کنجروں جو تم نے قرض لیا ھے ماں یہکو اسکا حساب دو کہ کہاں گیا؟ | 1 |
|  | 2090 | وزیراعظم صاحب کشمیر کی بیٹیاں ایک اور محمد بن قاسم کی منتظر ہیں ہماری بہنوں کی عزتیں تار تار ہو رہی ہیں | 0 |
|  | 1103 | کشمیر بیچ کے اب وطن پہ ڈرامہ بازیاں کررھے ھیں ب چ کتے سؤر خنزیر | 0 |
|  | 1102 | کیا کبھی کسی نے سوچا ہے کہ اگر بھارتی جوہری ہتھیار کسی آزادی پسند گروہ کے ہاتھ لگ گئے تو اس دنیا میں کیا ہو سکتا ہے | 0 |
|  | 1619 | حرام پے پلے ھوے حرامی۔ | 1 |
|  | 1407 | ایک غریب عورت نے فردوس عاشق اعوان کو جوتا گفٹ میں دے دیا ۔ زیادہ ب… | 0 |
|  | 1531 | چودھری صاحب اب کر گزرو سرکار، وعدے دعوے بہت ھو گئے۔ اب ڈیلیور کریں | 0 |
|  | 1343 | گشتی ماں کی گندی اولاد | 1 |
|  | 2320 | یہ جتنے بھی چاہیے بھونکے ۔لیکن خان نے اسکی گانڈ ماری ہے | 1 |
|  | 2827 | بھارتی افواج کے مسلسل جبر ظلم اور تسلط نے اس خوبصورت وادی کو جو کہ جنت کی طرح ہے جہنم میں تبدیل کر دیا ہے | 0 |
|  | 2001 | یہ بے غیرت فیصلے کرتے رہے وہ کشمیر پر پورا قبضہ کر گیا ہے اور اب بھی یہ فیصلے ہی کرتے رہیں گے بے غیرت لوگوں کی ٹیم جمع کر رکھی ہے | 1 |
|  | 2298 | پاکستان کے آئین و قانون کے منہ پر طاقتوروں ڈاکوؤں کا زور دار طمانچہ خاص کر نیب پر اور موجودہ تبدیلی سرکار پر | 0 |
|  | 1346 | ٹٹے پوشی اتنی کر جتنی برداشت کرسکے | 0 |
|  | 3367 | احترام کرنا تربیت ہے ، کمزوری نہیں اور معذرت کرنا حسنِ اخلاق ہے ، ذلت نہیں | 0 |
|  | 3247 | چوتئیے تو سب ہوتے ہیں لیکن ایک بھگوڑی کو لیڈر اور قوم کی بیٹی کہنے والوں کا اپنا ہی لیول ہے | 1 |
|  | 788 | جاہل اور گھٹیا قوم انڈینز | 1 |
|  | 2316 | شیدے ٹلی مادرچود جا مر کشمیر تے مادرچود | 1 |
|  | 2945 | وہ تم ہو نا ویسے جتنی تیری ماں بہن کو گالیاں پڑتی ہیں غیرت مند ویسے ہی مر جاتا ہے | 1 |
|  | 3440 | بکواس بند کر خنزیرہ | 0 |
|  | 2815 | بھڑوا ہے سالا چوتیا ،پاک فوج پہ بکواس کرتا | 1 |
|  | 3373 | اب کشمیر ایشو پہ کیوں بولتی بند تم لبرل موم بتیوں کی زبان کیوں گونگ ہوگئی کشمیری خواتین پر مظالم کیخلاف | 0 |
|  | 174 | کسی گشتی کے بچے حرام زادے باپ کی اولاد حرامی اولاد جب تم لوگ اپنے باپ کت نہیں تو مادر چود بھڑوے حرامی پاکستان کے | 1 |
|  | 3166 | تاریخ گواہ ھےکےامریکیوں اور یورپی قوموں کےحکمرانوں نےکبھی مسلمانوں کاساتھ نہیں دیا۔ | 0 |
|  | 1819 | کشمیر کا سودا کرنے والے بیغیرتوں پر الللہ کا عزاب نازل ہو لعنت ایسی حکومت پر اور اسکے وزیروں پر | 0 |
|  | 1759 | او بےغیرتوں یہ جو تم نے نیا کھسرا وکیل کیا اسکی مرحومہ ماں کی جو تصاویر… | 1 |
|  | 1949 | ایک بڑے اسٹیڈیم میں لاکھوں کا یہ مجمع ایک اسلامی سکالر کو سننے آئے ہیں۔ | 0 |
|  | 2287 | فلسطین میں نمازِعید کے دوران فاٸرنگ ایک افسوس ناک واقعہ ہے ۔دکھ ہو رہا ہے یہ ظلم دیکھ کر کہاں ہیں امن کے پاسدار | 0 |
|  | 115 | یہ ہیں وہ زرائیع جن کا حق چھین کر پوری دنیا میں پراپرٹی بنائی گئی ہے یا اللہ ان غریبوں کا مال کھانے والوں کو عبرت ک… | 0 |
|  | 167 | پولیس والے گانڈو ہیں ورنہ ہڈی پسلی ایک کر کے رکھ دیتے | 1 |
|  | 3314 | انتہائی افسوس کے ساتھ آج یہ تحریر لکھ رہی ہوں | 0 |
|  | 2732 | کشمیر آزاد کروانے کے لیے اس کی بیٹی کو چوک میں چودا جائے ۔سالا حرامی بہن چود ۔شامی کتا سور | 1 |
|  | 1750 | کیسا غلیظ بہن چود ہے بادامی۔ بھٹو کی تیسری نسل سندھ پہ حکومت کر رہی ہے اور اس کتے کو بلو رانی کو ٹیگ | 1 |
|  | 1598 | سب ڈرامے بازی ہے ملکی چوروں کی ۔ آج کیس ختم کر دو ان کنجروں کی ساری بیماریاں ختم ہو جائیں گی | 1 |
|  | 834 | میرا خیال ہے کہ اب وقت آہ گیا ہیکہ حکومت اور اپوزیشن این آر رو اور دیگر باتیں سایڈ پہ رکھ کر ایک پیج پر آہ جاہیں | 0 |
|  | 1176 | بیغیرت انسان کتے کے بچے | 1 |
|  | 1123 | بلکل ٹھیک ویسے میں نے طنز بھی کیا تھا اور بیغیرتوں کو شرم بھی دلا رہا تھا | 1 |
|  | 3115 | کھوتی کے بچو بس بھونکتے ہی رہتے ہو ہر وقت مولانا فضل الرحمٰن کی پالیسی پر تب تو انڈیا کی ہمت نہیں | 1 |
|  | 2750 | تتہ پانی سیکٹر بھارتی فوج کے دو بنکر تباہ افسر سمیت فوجی جہنم واصل ویلڈن پاک آرمی | 0 |
|  | 424 | وہ تمام بےغیرت کنجر پٹواری اور جہالے جنکو مریم بھگوڑی کی گرفتاری پر بےتحاشہ تکلیف ھوٸی ھے۔ ان بےغیرتوں سے ایک سوال ہے | 1 |
|  | 1976 | میرے باپ نے تو تیری امی کی گانڈ بھی ماری تھی وہ تجھے یاد نہیں | 1 |
|  | 528 | کنجروں کا ٹولہ اور تم نے بھی خون ڈھول بجا ئے اب پارسا بنتے ہو۔ | 1 |
|  | 1558 | اللہ تعالی عثمان بزدار کو بھی ہدایت دے میرے سمیت ۔ اللہ تعالی نے عزت بخشی ھے تو اسے کہیں کچھ کام بھی کر لے۔ | 0 |
|  | 681 | بےغیرت تو تم ہو اپنی بہن کو بازار میں لے آئیے اور کتوں کی طرح مردانگی دکھا رہے ہو لندن سے آگر | 1 |
|  | 2272 | اف توبہ ایسا ظلم تو نمک حرام ہی کر سکتے ہیں | 1 |
|  | 499 | ننگا کر کے کھمانا چاہیے اس دلی کو تاکہ آیئندہ کسی غریب کو ایسے ذلیل نا کریے یہ حرام کی | 1 |
|  | 3187 | یہ حاجی نہیں ہے اور نہ یہ حج کرنے گیا ہے یہ حرامی ہے جو کسی کے ہاتھ بک گیا ویڈیو بنانے کے لیے | 0 |
|  | 3348 | پین دی سری،راج ناتھ سنگھ ہم جانتے ہیں بندر کے ہاتھ ماچس آئی ہے۔ | 0 |
|  | 1076 | تیری ماں کو کوٸ نون لیگی نہی چودتا تھا وہ نمبر دار کی رکھیل تھی تیرے پیدا ہوتے ہی گشتی کو مار دیا گی | 1 |
|  | 1910 | اللہ بہتر جانتا ہے کہ مجھے کتنا جینا ہے مگر جب تک زندہ ہوں اپنی پوری طاقت کے ساتھ وہی کہوں گا جسے سچ سمجھتا ہوں۔ | 0 |
|  | 2516 | فوج نے کونسا تیری گانڈ ماری ہے جو بھونکتا جا رہا ہے نکل تیری زات کا بھیدا ماروں | 0 |
|  | 2869 | مسلمانوں کے ساتھ ساتھ سب بےغیرتوں کو بھی عید مبارک | 1 |
|  | 243 | دو چار دن جیل میں رہنے کے بعد اس کی بولتی بند ہوگئی ہے | 0 |
|  | 2335 | عزت کا ایک سال کس منہ سے کہہ رہے ہو کچھ علم بھی ہے اس عزت کے ایک سال میں کتنی ذلت سے کتنا قرض اکھٹا کیا ہے؟ | 0 |
|  | 3174 | جتنا آپ کو بکواس کر کے ہوتا | 0 |
|  | 1338 | تب یہ مادرچود ایم کیو ایم میں تھا اور اپنا سیاسی چکلہ پی ایس پی نہیں کھولا تھا | 1 |
|  | 190 | یوم سیاہ ، بھارتی فوج کی مقبوضہ کشمیر میں بدترین ریاستی دہشت گردی پراقوام متحدہ کی خاموشی افسوس ناک ہے | 0 |
|  | 977 | سارے سندھی نمک حرام کام چور جس تھالی میں کھاتے ہیں اسی میں چھند کرتے ہیں اسی میں تھوکتے ہیں | 0 |
|  | 1183 | تحریک طالبان کی طرف سے عوامی نیشنل پارٹی کے صوبائی جنرل سیکرٹری سردار حسین بابک کو دھمکی آمیز خط موصول | 0 |
|  | 3195 | واہ اوئے چوتیوں مودی بغیر تھوک لگائے تمہاری گانڈ مار رہا اور تم اب بھی پتہ نہی کونسی امیدیں لگائے بیٹھے ہو ب چ وہ ک… | 1 |
|  | 3154 | کوئی اس بے غیرت کو بتائے اس کی بہن اس جائیداد کی وجہ سے جیل میں ہے جس میں یہ الحمدللہ کہہ کر مفرور ہے | 1 |
|  | 1571 | مجھے تو یہ ان کنجروں کی سازش لگتی ھے۔ | 1 |
|  | 3062 | اللہ ان بے غیرتوں کی ھدائت دے ورنہ ان کنجروں کو اٹھا لے | 1 |
|  | 711 | مادرچود ہم کشمیریوں کا نام مت لے | 1 |
|  | 2867 | لعنت ایسے غداروں پر | 1 |
|  | 2803 | بے غیرت انسان یہ کی اولڈ ویڈیو ہے کراچی میں دھاندلی کے خلاف | 0 |
|  | 1161 | ان محترمہ کو ملک لوٹتے ہوئے شرم نہ آئی کراچی کا حال خراب کرتے شرم نہ آئی سندھ کا بیڑا غرق کرتے شرم نہ آئی | 0 |
|  | 2342 | کپتان نے یوٹرن لیتے ہوئے آج قوم سے بکواس کرنے کا پروگرام کینسل کر دیا کچھ بتانے کو ہے ہی نہیں تقریر تیار کرنے والے… | 0 |
|  | 1607 | یا اللہ۔ اسے روڈ پر جاتے ہوئے ٹرک کے نیچے دے دے تاکہ اس کا سر ٹائر کے نچے آ کر دھماکے سے ہھٹ جائے اور اس کا دماغ ٹر… | 0 |
|  | 2626 | یہ بہت کمینہ انسان ہے۔ | 1 |
|  | 1326 | بےغیرت مودی | 1 |
|  | 29 | تیری بیٹی کی کوس مبارک۔ بہن چود ، بیٹی چود، حرامی ، دلال | 1 |
|  | 1329 | بہن چود گشتی کے بچے اپنی فوج کی | 1 |
|  | 1929 | نیا مادر چود کو اضافہ ہوگیا کون ہے یہ انجم کتا اس کو سرعام گولی مارنا | 0 |
|  | 2639 | پنڈی کے ہر لڑکے نے اس کی گانڈ ماری ہوئے ہے | 1 |
|  | 3297 | جنرل راحیل کےہوتےہوئےانڈیا کی بھی بولتی بند تھی آگر آج اس ملک میں آمن ہے تو نواز شریف اور جنرل راحیل شریف کی وجہ سے ہیں | 0 |
|  | 2885 | کیسی نوکری ہے اس مادرچود کی عید کے دن بھی چھٹی نہیں ہے | 1 |
|  | 975 | کشمیر بیچ دیا تم بےغیرتوں نے | 1 |
|  | 559 | دھمکی آمیز خط طالبان نے ہی بھیجا ہے نا ؟ | 0 |
|  | 3086 | جنرل قمر باجوہ نے بھارتی فوج کو ڈنڈا دیا اور اب آئندہ تین سال ان کے ساتھ کیا کرتے رہیں گے | 0 |
|  | 65 | بیڑا غرق ہو ہمارے الیکشن کمیشن کا جنہوں نے کتوں کو انتخابی نشان شیر دے دیا | 1 |
|  | 3100 | مشاہد کی گانڈ آپ نے ماری ہیں اس گانڈو نے بیمار ہونا ہی تھا | 1 |
|  | 1920 | اوہ مغل اپنی گندے لفافے کے لیے کشمیر پر بکواس بند کر۔وہ ہر پاکستانی کا مسئلہ سوائے تم جیسے کتوں کے | 0 |
|  | 2955 | ابھی تو تم مادرچود فائدہ اٹھانے کی کوشش کر رہی ہو۔ ملک دشمن | 1 |
|  | 3299 | نسلی کنجر ہے اور آج کل کنجروں کے ساتھ ہی تو ہوتا ہے | 1 |
|  | 2419 | جس مادرچود کو پیدا کرنے کے بعد ماں چاٹنا تک پسند نہیں کرتی وہ حرام کا تخم یہاں پاکستان اور اس کے اداروں کے خلاف بھو… | 1 |
|  | 3453 | تحریک انصاف حکومت کی خیبر پختون خواہ میں سال لیکن ہسپتالوں میں سہولیات کا فقدان | 0 |
|  | 3324 | اب وہ تمام مادرچود لبرلز ۔صحاف | 1 |
|  | 1944 | میرے پیارے وطن پر بکواس کرنے والو اپنی خیر مناؤ | 0 |
|  | 784 | انڈین میڈیا اپنے ملک سے وفاداری کررہا جبکہ پاکستانی میڈیا پر جو حالات چل رہے وہ اتنہائی افسوس ناک ہے | 0 |
|  | 1805 | بیغیرت انسان کتے کے بچے | 1 |
|  | 2987 | سوات میں سکول بند ہونے پر ملالہ نے پوری دنیا میں پاکستان کے خلاف بکواس کی تھی ایک ہفتے سے کشمیر میں سکول بند ہیں اب کہاں مر گئی ہے۔ | 0 |
|  | 1723 | اس سے یہی ثابت ہوتا ہے کہ جب تک بیوقوف زندہ ہیں ٹھگ بھوکا نہیں مر سکتا | 0 |
|  | 1086 | بیٹا ، شادی وہ جنگل ہے جہاں بڑے بڑے شیروں کی کھال، ٹیڈی بکریوں نے اُتار ی ہے | 0 |
|  | 143 | اس کے لئے مولانا حمداللہ کو بلانا چاہئے ماروی صرف اس سے ڈرتی ہے جس نے TV ٹاک شو میں اس کی طبیعت صاف کی تھی | 0 |
|  | 1330 | نوازشریف نے امریکی غلامی سے جان چھڑائی | 0 |
|  | 792 | سوات میں سکول بند ہونے پر ملالہ نے پوری دنیا میں پاکستان کے خلاف بکواس کی تھی۔۔۔ ایک ہفتے سے کشمیر میں سکول بند ہیں… | 0 |
|  | 2617 | پاکستان فوج کی بھارت سرحد پر جوابی کاروٸی | 0 |
|  | 783 | چوسنےکو کوٸ اور مناسب چیز نھی ملی ان بیغیرتوں کو اچھی چیزیں بولے تو بیغیرتوں۔لوٹیروں۔منی لانڈروں۔چوروں کا ٹولہ بیغیرتوں کا خون ہے بیغر ت ہی ہوگا | 1 |
|  | 2109 | پھر وہ کچھ بنے نہ بنے نشان عبرت ضرور بنت… | 0 |
|  | 2657 | پہنڑ چودو رسیداں کڈھو بکواس نہ کرو | 1 |
|  | 449 | بے شرمی پرتوگ دی رانہ ا… | 0 |
|  | 3090 | جسکو ٹیگ کررہا ہے نا تو یہ اسی مادرچود حرام النسل گانڈو کی بیٹی ہے | 1 |
|  | 2107 | کس نے بولا تھا چوری کرو مادرچود بھڑوے گن… | 1 |
|  | 2757 | یہاں سب ہیو من راٸٹس والے مر گے ہیں | 0 |
|  | 3261 | ابے مادرچود ۔ سکھ مودی کا فرق سمجھ | 1 |
|  | 1319 | پاک فوج کا منہ توڑ جواب افسران سمیت بھارتی فوجی جہنم واصل کئی مورچے بنکر تباہ | 0 |
|  | 1055 | اور ہمارا آفس کھل چکا ، ہم اپنے دفتر میں بیٹھے مکھی مار اسپرے کرہے ہیں۔ | 0 |
|  | 1924 | ماں سے پوچھ کتی کے بچے، کس گشتے سے مروا کر آئی تھی، تو یہ گشتی تیری بہن پیدا کی | 1 |
|  | 832 | او کسی کنجری کی اولاد قادیانی مسلمان نہیں ہے کتے کے پتر قادیانی اس دنیا کے بدترین اور غلیظ ترین کافر ہے ا… | 1 |
|  | 1709 | مودی تم جو بھی کرلو ہم جانتے ہیں کہ جب انسان ڈوب رہا ہو توہاتھ پاوں مارتا ہے تو یہ تمہارا اخری وق | 1 |
|  | 2279 | کابل میں شادی کی تقریب میں کی جانے والی دہشتگردی کی شدید مذمت کرتے ہیں۔ مالی اور جانی نقصان پر دکھ ہے۔ایسی کاروائیا… | 0 |
|  | 2666 | اس کا مطلب ہے تیری ماں کیسی کتیا کے بچے لعنتی بے غیرت چوتیا مادرچود دھلے | 1 |
|  | 3116 | سب سے بڑے مادرچود ہیں یہ دونوں | 1 |
|  | 3215 | عمران خان نے آنکھ میں آنکھ ڈال کر گھٹنے ٹیک دئیے | 0 |
|  | 2788 | اقوام متحدہ کی رپورٹ کیمطابق انفرا اسٹرکچر کے حوالے سے ممالک میں پاکستان تباہ حالی کے اعتبار سے نمبر پر ہے۔کراچی آپکے سامنے ہے۔ | 0 |
|  | 3328 | دلے کے بچے تجھے عزت راس نہیں آ رہی اپنی ماں کو چود کے پیدا ہعنے والے ماں کے کس زیادہ ٹڑ ٹڑ نا کر | 1 |
|  | 2673 | پتوکی جلسے میں نواز شریف کے شیر نے سٹیج پر آپ کی گانڈ میں اُنگلی دی | 1 |
|  | 3202 | عمران نے سر عام جھوٹ بولے پر وہ نیک کیوں کیوں کے میڈیا یہودو نصارا کے ہاتھ ہمارے لوگوں کو خرم خوری کی عادت ڈال دی گ… | 0 |
|  | 2045 | فالورز بڑھانے والے ٹویٹ اور ایسی ری ٹویٹس کرنے والے مجھے ان فالو کر دیں برائے مہربانی۔ مجھے یہ بکواس نہی چاہیئے اپنی ٹائم لائن پر۔ | 0 |
|  | 2131 | جی بالکل اور الحمدللہ آج وہ دونوں کتے جیل میں ہے | 1 |
|  | 1057 | جھیل نمکین نہیں تو نمک حرامی کا سوال ہی نہیں، ہاں پورا اندرونِ سندھ کراچی ضرور پال رہا ہے، تو نمک حرام تو تم ہوئے | 0 |
|  | 1539 | ظالموں بےغیرت حکمرانو اتنا ظلم کرو جتنا کل کو برداشت کر سکو | 1 |
|  | 369 | انشإ اللہ تمام بددعاوں کے ساتھ فوج اور اسکے ٹٹو قبروں کو جایٸں گے پوری زلت اور عبرت کے ساتھ | 0 |
|  | 3459 | قبرستان ایسے صحافیوں کے ساتھ بھی بھری پڑی ہیں جنہوں نے ساری زندگی صرف منافقت، جھوٹ اور نفرت پھیلانے کا کام کیا اور… | 0 |
|  | 3023 | مجھے معلوم ہے تجھ جیسے ماہا حرامی کی گانڈ جب تک ماری جائے تو بین چود کے بچے کتے کی طرح کاٹتے رہتے ہیں | 1 |
|  | 1289 | سب سے بڑے مادرچود ہیں یہ دونوں | 1 |
|  | 99 | کاش تو نے حرام کی کمائی اپنے بچوں کو نا کھیلاتا تو بیٹا اس کی قیمت نہ ادا کرتا پر افسوس تم بیغیرتوں کو پھر بھی عقل نہیں آنی | 1 |
|  | 2845 | جو بہن بھاٸی اس بغیرت خبیث جاوید اقبال پر لعنت نہیں بھیجے گا وہ ن لیگی نہیں ہوگا اس بغیرت انسان کی منحوس شکل پر | 1 |
|  | 292 | اپ اپنا کام کیا کریں کتے بھونکتے رہتے ہیں | 1 |
|  | 3018 | بہن چود غدار کتے کے پلے اب تیری کےت والی ہو کے رہے گی | 1 |
|  | 2355 | اس دنیا میں ہر شخص اتنا ہی پریشان ہے جتنی اس کی نظر میں دنیا کی اہمیت ہے | 0 |
|  | 1435 | تتہ پانی سکٹر میں انڈین فوجی اور افسر سمیت بکر تباہ | 0 |
|  | 2403 | او خبیث انسان تیری ماں یا بہن کا ہاتھ پکڑنے کو دل کر رہا ہے میرا بھی جیسے اس نے اس عورت کا ہاتھ پکڑا ھے | 1 |
|  | 350 | کتے کے بچے، حرامی سور تو مخبری کرنے بیٹھا ہوا ہے یہاں غدار نسل کے کتے | 1 |
|  | 3091 | یہ تھے حقیقی مہاجر۔ جنہوں نے ہجرت کا دکھ دیکھا اور سندھ کا سکھ بھی | 0 |
|  | 2508 | یہ مادرچود بڑا فیمینسٹ نہیں بڑا مادرچود ہے | 1 |
|  | 3026 | مریم کی گانڈ میں مرچیں دیں گے آج رات اور پھر وہ بتا دے گی کے۔اسے قطری شہزادے نے چار کروڑ کب دیے تھے | 0 |
|  | 21 | کس نے بولا تھا چوری کرو | 1 |
|  | 2555 | لعنت ہے سندھ سرکار پر | 1 |
|  | 1203 | مولیاں اندر کروا کروا کے اس کی گانڈ اس قابل نہیں رھی کہ ماری جاۓ | 1 |
|  | 482 | شیدا ٹلی پیشگوٸیاں کرنے میں مصروف بیغیرت نے ریلوے کا بیڑا غرق کردیا ہے۔ | 1 |
|  | 2212 | اوے بے غیرتوں | 1 |
|  | 3019 | پاکستان اسٹیل ملز کراچی کے سے رٹائرڈ ملازمین کو واجبات کیوں نہیں دئے جا رہے۔ وفات پا چکے | 0 |
|  | 172 | انڈیا نمک حرام قوم ہے | 0 |
|  | 1213 | لعنت اس کتے کے شکل پر | 1 |
|  | 2492 | جناب محترمہ مریم نوازشریف زندہ باد | 0 |
|  | 2800 | اسلام امن کا درس دیتا ھے | 0 |
|  | 1706 | سال سے جس کشمیر کو پاکستان کی شہ رگ بتا کر بچوں کو تعلیم دی جاتی تھی اس شہ رگ پر اب ہندو بنیے نے پاؤں رکھا ہوا ا… | 0 |
|  | 3135 | اوہ کنجر بیٹی فروش | 1 |
|  | 3084 | لولز یہ کی ویڈیو ہے عجیب قوم ہے جو حقائق کے بجائے خیالی دنیا میں رہتی ہے جب ہی زلیل و رسوا ہے | 0 |
|  | 2352 | بہت سے لوگ یہاں بکواس کرتے پھر رہے ہیں کہ عمران خان نے الیکشن سے پہلے کچھ اور وعدے کئیے تھے اور اب کچھ اور کر رہا | 0 |
|  | 3208 | یہ آج جو تم غریب ہو، چور ہو، غیر تعلیم یافتہ ہو | 0 |
|  | 1784 | دیکھ لو ڈیر رنڈین اکیلا شیر کھڑا ہے | 1 |
|  | 360 | ان کی اس مثالی دوستی کی وجہ سے اس جنگل کا شیر ان پر ہاتھ نہیں ڈال سکتا تھا | 0 |
|  | 882 | کنجر جب بغیر ویزے تیرے لیڈر کا یار مودی آیا تھا نواز بٹ کے گھر۔ | 1 |
|  | 1485 | تو بکواس بند کر | 0 |
|  | 3338 | اس گھٹیا حکومت نے قسم کھائی ہوئی ہے کہ کسی بھی موقع پر عوام کو کسی بھی طرح کا ریلیف نہیں دینا | 0 |
|  | 1717 | لعنت تمہاری گٹھیا سوچ پر غلام پیدا ہوۓ غلام ہی جیے اور غلام ہی مرو گے | 1 |
|  | 561 | حرام زادے تیری ماں گشتی نے تجھے شعور دیا باپ سے کس طرح بات کرتے ھیں بوسڑی کے | 1 |
|  | 1528 | اوئے بے غیرت | 1 |
|  | 2993 | کیا یہ سب پہلے معلوم نہیں تھا… اگر معلوم تھا تو پھر امت کے ان محافظوں کی ڈرایئوری کیوں کی گئ | 0 |
|  | 643 | اپنے حرامی باپ نوازشریف اسکی بھگوڑی بیٹی سے پوچھو | 1 |
|  | 3030 | لن چوپ | 1 |
|  | 2028 | لعنت ہے تیرے عورت ہونے پر بیغرت زلیل عورت | 1 |
|  | 1832 | کاش اسرا تمہارے باپ کا کونڈم نہ لیک ہوتا تو تم جیسا خبیث انسان نہ ہوتا | 1 |
|  | 2891 | انڈیا نے مجاہدوں کو للکارا ہے انڈیا کا نام و نشان ہم مٹادیں گے۔ | 0 |
|  | 2605 | مادرچود بکواس بند کر | 1 |
|  | 1043 | جاپان میں الیکٹرک پولز میں جہاں تک انسان کا ہاتھ جاتا ہے وہاں پہ پلاسٹک کوٹنگ کی ہوئی ہے | 0 |
|  | 770 | وہ آزادی جس کو ہم نے فراموش کر دیا غور سے دیکهیں ہم نے آزادی کیسے حاصل کی تهی | 0 |
|  | 1339 | زبان اندر رکھیں گے تو انہیں تکلیف ہو گی… | 0 |
|  | 1144 | لعنت تیرے منہ پر بہن چود نامرد کشمیر کے مظلوم عوام پر ظلم کرتے ہو | 1 |
|  | 2570 | کشمیری بھائیو ان بیغیرتوں سے امیدیں مت لگاو وہ نظر نہیں اتا ان بےغیرتوں کو عوام مر رہی | 1 |
|  | 3467 | عالمی سرحدوں کی وجہ سے ہندو بچے ہوئے ہیں ورنہ اب تک ہم انکی بہن چود دیتے | 1 |
|  | 906 | یہ بہن چود پیسے کے دم پر خدا بنے بیٹھے ہیں۔ | 1 |
|  | 2240 | حق کی بات کرنے والوں کوقید جیل مقدمات جلا وطنی سزاؤں عمر قید سے ڈرایا جاتا ھے | 0 |
|  | 2509 | مونی اور شیلا کے بعد جو سب سے زیادہ بدنام ہوے وہ بلو رانی اور مریم بھگوڑی ہیں | 1 |
|  | 2138 | قبائلی عوام کا ایک دفعہ پھر پاکستان زندہ باد کے نعروں سے گونج پٹنا ان شر پسند عناصر کے منہ پر ایک طمانچہ ہے | 0 |
|  | 1878 | کل رات پاک فوج کےدستےہیوی آرٹلری کے ساتھLOCپر پہنچے جہاں کشمیری عوام نے کشمیر بنےگاپاکستان کےنعروں سےاستقبال کیا | 0 |
|  | 1366 | اے میری جان پروردگار اب ہم امت سے ہم پاکستانیوں سے وہ کام لے لیں جس کام کے لئے ہمیں آزادی ملی۔ | 0 |
|  | 2665 | محمد تیمور شہید کا تاریخی نماز جنازہ لیکن کوئی میڈیا کوریج نہیں | 0 |
|  | 2905 | ان ڈاکٹروں کی گانڈ ماری جانی چاہیے جو کہ رہے تھے کہ فریال تالپر بہت بیمار ھے | 1 |
|  | 1049 | مردار سعید سے صرف بڑی باتیں کروا لو، اس کونی کے اپنے حلقے میں بجلی کا برا حال ہے، لوگ خون کے آنسو رو رہے ہیں | 0 |
|  | 2480 | مریم نواز مریم صفدر مریم قطری یا مریم بھگوڑی | 1 |
|  | 1129 | یہ ان بےشرم لوگوں کے منہ پر ایک طمانچہ ہے جو افغانستان میں پشتونوں کے قتل عام کو جہاد کہتے ہیں | 0 |
|  | 3 | کسی گشتی کے پتر ہیں یہ حرامی کافر مادرچود | 1 |
|  | 3430 | یے سانپ کرائے کا ہے کل کسی اور جگا ہوگامسٹر سلیکٹڈ | 0 |
|  | 1417 | اتنی منافقت کر رہا ہے یہ خنزیر کا بچہ کوئی پلیز ایسے لوگوں کو سرعام گولیاں مار دے خدا کی قسم سب ٹھیک ہو جائے گا | 1 |
|  | 148 | اگر بھارت کے لوگ ۂولناک غُربت سے نکلنا چاہتے ہیں اور امن کی راحتیں سمیٹنا چاہتے ہیں تو موُدی کا گریبان پکڑیں | 0 |
|  | 2031 | مودی سب سے بڑا دہشت گرد ھے حرامی ھے کتا ھے بیغیرت ھے دنیا کاسب سے بڑا بیغیرت اور اور سب بڑا دہشت گرد مودی کتا کنجر حرامی ھے | 1 |
|  | 599 | تیری ماں کو بیچ آئے ہیں دلا ہاراں والے کے پوتے بےشرموں جھُوٹے پروپپگنڈہ کرنا بند کرو | 1 |
|  | 2436 | جس کا کام چل جاتا ہے ، فرعون بن جاتا ہے۔ ان سب حرامیوں کو حوالات میں چمڑے کے چھتر مروانے چاہئیں | 1 |
|  | 235 | حنا کھسرا | 1 |
|  | 2664 | نون اور پیپلز پارٹی کے لوگوں کا پتا کرو مر تو نہیں گئے جس دن سے کشمیر کی بات ہو رہی ہے ان کی بولتی بند ہو گئی ہے | 0 |
|  | 3450 | کاش بلاک کی جگه هلاک کا آپشن لکها هوتا کیونکه کچھ لوگوں کو بلاک کر کے بهی کلیجے کو ٹھنڈک نهیں پڑتی | 0 |
|  | 1107 | پاکستانیو اپنی پاک آرمی کا ساتھ مت چھوڑنا | 0 |
|  | 636 | سولہواں روز ہے کشمیر میں کرفیو کا۔ وہاں کیا گذر رہی ہے ، کچھ نہی معلوم ۔ | 0 |
|  | 3488 | ٹھگ آف دی پاکستان کی ادھوری شھزادی بھگوڑی | 1 |
|  | 654 | لعنتی قسم کے انسان آپس میں لڑو مرو جس کابل تم لوگ ہو چوروں ختم نبوت کے غداروں بابا جی رضوی صاحب کے بارے بولتے ہو | 0 |
|  | 746 | آپ اچھا جواب دیتی ہیں بولتی بند کر دیتی ہیں | 0 |
|  | 1188 | دن رات زلیل ہو کر بھی لعنتیں وصول کر کے بھی حرام خور کو شرم نہیں آتی | 1 |
|  | 2091 | ان قاتل کنجروں کا ایک ہی حل سب کو اکٹھے باندھ کر کراچی سے بیس میل دور سمندر میں پھینک دو | 1 |
|  | 2922 | اور تم جیسے کتوں کی پھانسی سے پہلے گانڈ بھی ماری جائے | 0 |
|  | 364 | مادرچود، اپنی ماں کے جنازے پر آیا نہیں اور بکواس کر رہا ہے | 1 |
|  | 273 | پاکستان سے ہزاروں میل دور بیٹھی ثنا تتلی کی آنکھوں اور اس کے تاثرات آپ کو ملک سے محبت کا درس بھی ملے گا اور حوصلہ… | 0 |
|  | 1785 | سکوں کی نیند تجھے بھی حرام ہوجائے۔ | 0 |
|  | 3061 | جب کوئی بھوک کی وجہ سے چوری کرنے پر مجبور ہو تو چور کے نہیں بلکہ حکمرانوں کے ہاتھ کاٹ دینے چاہیئے۔ | 0 |
|  | 795 | چوتیاپ۔ ٹویٹر پہ آکے بس بکواس کرنا ہوتی ہے۔ پتہ چاہے کچھ بھی نہ ہو | 0 |
|  | 2569 | میں ویڈیو ڈھونڈ رہا ہوں پٹوارنوں کا حلال ڈانس کی پھر میں تیری طبیعت صاف کرتا ہوں | 0 |
|  | 2576 | مادرچود فرنگی چھترا | 1 |
|  | 269 | استغفراللہ العظیم یااللہ بےبس کشمیری اور انڈیا میں رہنے والے مسلمانوں کی حفاظت فرما | 0 |
|  | 3465 | قوم کی بیٹی کون کہتا ہے چوتیئے؟ اُس بھگوڑی کو مادرِ ملت کہتے ہیں وہ چھنال بڑھاپے میں | 1 |
|  | 1273 | یہ بات سچ ہے کہ ہر معاشرے میں اچھے اور بڑے لوگ موجود ہوتےہیں ۔انڈیا میں بھی اچھے لوگ موجود ہیں | 1 |
|  | 785 | مقبوضہ وادی میں آگ اور خون کا کھیل جاری معصوم کشمیریوں کے گھروں کو نذر آتش کر دیا گیا | 0 |
|  | 1171 | ابھینندن کو گرفتارکرنے والے پاکستانی کمانڈو کو ہندوستانی سیکورٹی اہلکاروں نے ہلاک کردیا | 0 |
|  | 604 | کیسے منائیں عید | 0 |
|  | 3287 | کسی سرٹیفائیڈ کھوتی کےبچےعالمی عدالت میں جانےکاراستہ دکھانےوالابھی تیرا وہی باپ تھا میاں جسکے نطفہ ناہنجار کے نتیجے میں پیدا ہوا تھا | 1 |
|  | 2840 | تو گدھے کا بچہ ہے مادرچود | 1 |
|  | 295 | اس گشتی کو میرے حوالے کر دیں | 1 |
|  | 3272 | حرامی حرامی ہوتا ہے باجوہ ہو یا نیازی | 1 |
|  | 2128 | اچھا یہ بتائیں ۔ آپکے دشمن کو یہ نمک کس نے دیا ؟ جس نے پہنچایا ہے وہ کوئ معمولی لوگ تو نہیں اور نا ہی یہ نمک پہنچا… | 0 |
|  | 3414 | مسلم لیگ ن زندہ باد سلکیٹیڈ کے بارے آپ میرے سے زیادہ سمجھدار ہیں | 0 |
|  | 1053 | بہن چود غدار کتے کے پلے اب تیری کےت والی ہو کے رہے گی | 1 |
|  | 1193 | کل کوٸی پوچھے کشمیر کس نے بیچا تھا | 0 |
|  | 2990 | بھارت والودیکھو اب پوری دنیامیں تمھاری یہی حالت ہوگی انڈیا کےاندر بھی اورباہربھی | 0 |
|  | 1993 | جی نہیں یہ بیغیرتوں کے سردار ہیں | 1 |
|  | 1520 | بلوچستان کے غیرت مند اور بہادر لوگوں نے انڈین ترنگے کے اوپر ذبح کر کے مودی کے منہ پر طمانچہ مارا | 0 |
|  | 1117 | لعنت ہو مودی کتے پی | 1 |
|  | 3408 | وہی جو قطری کے پاس مریم بھیجتا تھا مودی کی ماں کو ساڑھیاں بھیجتا تھا نوازشریف مادرچود | 1 |
|  | 2766 | تیرا بھی کوئی باپ انکل چاچا تایا ہے کہ بن مانس کی اولاد ہو گشتی نا ہو | 1 |
|  | 2068 | انڈیا نے عالمی برادری کے منہ پر طمانچہ مارا ہے یہی وقت ہے عالمی برادری کی غیرت کے جاگنے کا | 0 |
|  | 2205 | ان کنجروں نے تجھے صرف یہی سکھایا ہے کہ انڈینز کے آگے ٹانگیں کیسے کھولنی ہیں گشتی عورت | 0 |
|  | 887 | وزیراعظم جناب عمران خان صاحب نے ہندوستان اور پٹواریوں کو ایک بار پھر بڑا سرپرائز دے دیا ایک بار پھر باجوہ ڈاکٹرائن… | 0 |
|  | 2442 | ان کنجروں نے تجھے صرف یہی سکھایا ہے کہ انڈینز کے آگے ٹانگیں کیسے کھولنی ہیں گشتی عورت | 1 |
|  | 2856 | مادر چود رنڈی | 1 |
|  | 692 | لگتا ہے حرامی ہندووں تم اپنے باپ ٹیپو سلطان کو بھول چکے ہو جس کے آنے سے تمہاری ماوں کے بچے گر جایا کرتے تھے | 0 |
|  | 3220 | شاہ صاحب تو پھر انڈیا پہ حملہ کرنے میں دیر مت کرو۔ ہمارا کچھ بھی ہو لیکن ان کنجروں کا سرمایا تو ڈوب جاۓ ۔ شکریہ | 1 |
|  | 1562 | حرام زادے بڈھے تیری بیوی اور بیٹیوں کو اٹھا کر کشمیر کے پہاڑوں میں لے جائیں گے بہن چود | 1 |
|  | 2018 | اس مادرچود کی سنو، تم جا کر ٹی وی شو کرو | 1 |
|  | 697 | بکواس بند کر کتوں کے ساتھ شادیاں کرنے والی اور گائے کا پیشاپ پینے والے | 0 |
|  | 3156 | میئر کراچی وسیم اختر نے کہا ہے کہ سالڈ ویسٹ مینجمنٹ بورڈ شہر کو صاف کرنے میں ناکام ہوچکا، سیوریج نظام تباہ ہے بلدیہ… | 0 |
|  | 1080 | کوئٹہ کچلاک میں نماز جمعہ کی ادائیگی کے دوران دھماکہ پانچ افراد کے شہید اور گیارہ افراد کے زخمی ہونے کی اطلاع | 0 |
|  | 1154 | اپوزیشن کا صرف ایک ہی اعلاج ہے ان کی گانڈ میں گولیاں ماری جائیں یہ مادرچود کے بچے اس ملک کا بھلا کبھی نہیں چاہینگے | 1 |
|  | 1498 | چلیں بھائی پیارے تے سوہنڑے پاکستانیوں سب ایک ایک طمانچہ انڈیا کے منہ پر مارے اور فری خالصتان کا نعرہ لگائیں | 0 |
|  | 3270 | سودا تو تم لوگ کرتے ہو بے غیرتوں کنجروں دَلیوں حرامیوں تمہاری شکل پر لعنت پڑی ہوئئ… | 1 |
|  | 54 | اگر زرا سی شرم ہے تو ڈوب مرو کتے کے غلامو تمہارے باپ کوٹ لکھپت اور نیب کی کسٹڈی میں ہیں | 0 |
|  | 878 | اب ان جیسے میراثی کنجر ہمیں سکھائیں گے کہ سفارتی محاذ پر کارگردگی کیسے ہونی چاہئے۔ | 1 |
|  | 296 | خان صاحب ان چوروں کو اب لٹکا دیں | 0 |
|  | 1871 | مہنگائی عروج پر عوام بہت پریشان | 0 |
|  | 343 | یا اللہ پاکستان مخالف لبرلز اور سیاسی پارٹیوں کو کتے کی موت عطا فرما اور غداروں کے خاندانوں کو عبرت کا نشان بنا دے آمین | 0 |
|  | 400 | کرنل وجے اچاریہ نے مودی سرکار کے منہ پر طمانچہ مارتے ہوئے استعفی دے دیا۔ | 0 |
|  | 529 | تیری دوکان بند هے کیا کب اس نے بتایا بهن چود بھڑوے | 1 |
|  | 2567 | آپ اس کو دگڑ دلا بھی کہہ سکتے ہیں | 1 |
|  | 3117 | یہ ہی باجوہ عمران نیازی کو عبرت کا نشان بننآ ے گا | 0 |
|  | 2218 | سیف اللہ کا بیٹا شدید زخمی زرائع | 0 |
|  | 300 | یہ بکواس پاکستان کیلئے بھی کر اے کفن چور صحافی۔ | 1 |
|  | 1426 | عمران نیازی سال مسلسل بکواس کرتا رہا کہ بیرون ملک پاکستانیوں کے ارب ڈالر پڑے ہیں اور حکومت میں آتے ہیں یہ پیس… | 0 |
|  | 337 | اجازت دی جائے کہ ہم اپنے گھر چلے جائ… | 0 |
|  | 24 | ’غیر یقینی صورتحال معیشت کے لیے تباہ کن ہو سکتی ہے‘ | 0 |
|  | 1454 | گھٹیا عورت اس میں سوال کہاں ہے ؟ | 1 |
|  | 702 | بکواس بند آج اس کتے کا انڈیا میں بھگت رہے ہیں پاکستان اور کشمیر کے مسلمان ایک بیغیرت بدزات جس نے مسلمانوں کو زلیل کیا | 0 |
|  | 1780 | سر اس حرام زادے غدار کی بکواس پڑھ لے اس کا تو کوئی بندوبست کرے | 1 |
|  | 2954 | جیلوں سے ڈرنے والے نہیں مگر آپ کارویہ افسوس ناک ہے،نفیسہ شاہ | 0 |
|  | 3360 | آلو پیاز تو مہنگے سستے ھوتے ہی رھتے ہیں اصل چیز قومی دولت کی لوٹ مار کو روکنا ہیں | 0 |
|  | 437 | پاکستان کے جنرل تو صرف باتوں سے ہی انڈین کتوں کی نیند حرام کر رہے ہے اگر جنگ ہو گی تو کیا گا اندازہ لگانا بہی مشکل ہے | 0 |
|  | 2065 | آپکے موجودہ والد عمران نیازی سلیکٹڈ نے بکواس فرمائی تھی اور آپکے موجودہ | 0 |
|  | 2200 | انگلش میں ٹویٹ کرکے کونسے بہن کے یار کی اطلاع دے رہے ہو | 1 |
|  | 610 | پاک فوج نےبھارتی فوجیوں کو تباہ کر دیا ہے۔ | 0 |
|  | 3164 | بین چود خنزیر ان کنجروں نے سال کے گھنٹے چوری اور کرپشن کی لوٹ مار کی اب ان کو حکومت جس بھی جیل میں ڈالے یہ حکومت کا کام ہے | 1 |
|  | 2907 | شرم کریں کھوتی کے بچے بکواس بند کر۔ کشمیری اور پاکستانی ہی کافی ہیں اس انڈیا کے گند کو ختم کرنے کے لیے | 1 |
|  | 865 | وہ واقع تو بتا جب مولوی نے بچپن میں تیری گانڈ ماری تھی ؟ | 1 |
|  | 714 | بہن چود ہو تم | 1 |
|  | 2124 | لے ہم بھی ہیں صف آراں لے ہم بھی ہیں صف آراں | 0 |
|  | 2326 | کسی گشتی ماں کے بچے مخلوط النسل پہلے اپنا ڈی این اے ٹیسٹ کروا لو | 1 |
|  | 310 | تو بیغیرت ہے مادرچود دلے کنجر کے بچے تم سب چور ہو گشتی کے بچو گندی نالی کے کیڑوں حرام خوروں | 1 |
|  | 298 | لعنتی شیدا کتا انڈیا سے زیادہ تم کتوں نے میڈیا کو یرغمال بنایا ہوا ہے جب کرتوت مودی جیسے ہوں تم بیغیرت ہی ہو گا | 0 |
|  | 1742 | امریکی صدر ٹرمپ نے خود منٹ کی کال کی عمران خان کو صرف کشمیر کے مسئلے پر بات کی، | 0 |
|  | 963 | کیونکہ اب فواد حرامی جیسے کنجر ہمارے حکمران ہیں جو کشمیر کو بیچ آئے ہیں۔ ہمیں تم جیسے بے غیرت نہیں چا ہیے | 0 |
|  | 909 | اگر وقت پڑا تو قوم فوج کے شانہ بہ شانہ لڑے گی۔ پھر آخری گولی آخری نا ہوگی اور نا آخری فوجی آخری۔ دشمن کو پتا چل جاے… | 0 |
|  | 956 | معروف صحافی ملیحہ ہاشمی نے بھارتی ٹی وی پر بھارتی جرنیل کی بولتی بند کر دی | 0 |
|  | 3105 | عثمان ڈار تم اپنی بکواس اپنے پاس رکھو یہ ہسپتالوں کے دورے کرنا سیاستدانوں کے لئے ایک بالکل معمول کی بات ہے | 0 |
|  | 185 | عمران خان نے کن پاگل اور نا اہل لوگوں کو وزارتیں دی ہوئی ہیں | 0 |
|  | 359 | بھارت اس وقت نیو یارک ٹائمز، الجزیرہ سمیت پوری دنیا کے میڈیا کو جھوٹا کہہ رہا ہے | 0 |
|  | 512 | خدا کرپشن کرنے والے ہر سیاستدان کو عبرت کا نشان بنا دے آمین | 0 |
|  | 1500 | یہ مکافاتےعمل ہے۔ ان لوگوں نے مظلوموں کے ساتھ سندھ اور پنجاب میں جو کچھ کیا آج وہی کچھ ان کے ساتھ ہو رہا ہے۔ | 0 |
|  | 1425 | بھارتی میڈیا کہتا ہے کے پاکستان کے سوشل میڈیا میں جو جنگ ہو رہی ہے اس کے پیچھے کا ہاتھ ہے | 0 |
|  | 988 | پہنڑ چودو رسیداں کڈھو بکواس نہ کرو | 1 |
|  | 1243 | بھارتی فوج نے لائن آف کنٹرول پر جنگ بندی معاہدے کی خلاف ورزی کرت… | 0 |
|  | 80 | عالمی سرحدوں کی وجہ سے ہندو بچے ہوئے ہیں ورنہ اب تک ہم انکی بہن چود دیتے | 1 |
|  | 336 | سبکے سب کتے کی موت میں مرے گے کسی بھی روپ میں ا جائے یہ کتے ٹھوک کے رکھ دیے گے اب انشاء اللہ | 0 |
|  | 3041 | تیری بہن کی تو بولتی بند ہوئی پڑی ہے | 0 |
|  | 1072 | اس کٹھپتلی کو کشمیر کاسفیر نہ بننے دینا یہ کمینہ اس کشمیرکا بھی سودہ کر دیگا | 0 |
|  | 1131 | یہ سب کنجر لوگ ہے اور کنجروں کے گھر کنجر ہی پیدا ہوتے ہیں مجاہد نہیں | 1 |
|  | 910 | یہ ہے گشتی کی اولاد ہیرا منڈی کی پیداوار فک یو | 1 |
|  | 2561 | ان کنجروں نے تم کو عزت کرنا نہیں سکھائی | 1 |
|  | 538 | یاد رکھیے اس ملک کے جس نے بھی نقصان پہنچایا وہ عبرت کا نشان بنا ہے اندرا گاندھی اور مجیب الرحمان یاد کریں | 0 |
|  | 2193 | وہ صرف کتے کے بچے تھے۔ | 1 |
|  | 2888 | میڈم ریپ زدہ لاشیں ن لیگ کی دفتر سے نکلتے ہے اور زنا کار پی ٹی آئی والوں کو کیو ؟ مادرچود | 1 |
|  | 3470 | کتنے جسٹس مانگیں گئے اور ہر دفعہ ٹرینڈ ٹاپ پر آیا مگر ہو کیا کچھ بھی نہیں یہاں انصاف ہوتا تو آج یہ… | 0 |
|  | 40 | جو ڈنڈا تیری امی کو قطری نے دیا تھا اسکے بارے میں کیا خیال ہے مادرچود کتی نسل کے | 1 |
|  | 1732 | یہاں صورتحال اس سے نہیں زیادہ افسوسناک تھی ان کنجر پنجابی چوکیددارون نے ملک سے محبت بھی کم کردی اپنے حرامی پن | 1 |
|  | 37 | تیری ماں تیری بہن کے یار ہیں انڈیا والے | 1 |
|  | 1162 | اس گشتی کی شکل پر ابھی بھی وہی نحوست اور لعنت ہے۔ اسکو چھتر پڑنے چاہئیں۔ لاتوں کی ہے باتوں سے نہیں سمجھے گی | 1 |
|  | 2838 | امریکا میں چھوٹا طیارہ گر کر تباہ‘کوئی جانی نقصان نہیں ہوا | 0 |
|  | 661 | زندہ قومیں اپنی آزادی اور زندہ ہونے کا ثبوت قومی تہواروں کو بھرپور طرح سے منا کر دیتی ہیں۔ آٸیے اس یومِ آزادی دنیا… | 0 |
|  | 1261 | جنہوں نے خاموشی سے اپنے پڑوسی ملک افغانستان سے روس اور امریکہ جیسی طاقتوں کو بھاگنے پر مجبور کر دیا، | 0 |
|  | 913 | آفیشل نان آفیشل دونوں ہی جان سے عزیز ہیں یارہ ۔بس پاکستان مسلم لیگ نون کا پرچم ہاتھ میں ہونا چاہیے | 0 |
|  | 2760 | نون لیگی تھوکتے نہیں نیازی کہ پارٹی پرچم پر اور نہ نیازی کہ منہ پر اور نہ نیازی کی ان گھٹیا عورتوں پر | 0 |
|  | 1727 | ہمارا شہر لوٹ رہا ہے کراچی بلاول زرداری بغیرت انسان اب تیری غیرت کہا ہیں کنجر انسان حرام خور کی گندی اولاد | 1 |
|  | 1110 | اس وقت بس سمجھ لو بکواس ہی تھی اب سہی فیصلہ کیا ہے۔ | 0 |
|  | 3473 | کون سےکشمیر کی آزادی جس کا سودا نیازی کتے نے امریکہ جا کر کیا | 0 |
|  | 825 | اسی لئے زلیل بھی تو ہم ہی ہو رہے ہیں پڑوس میں بیٹھے ہیں ہم کشمیر کے اور ہم ٹرینڈ کر کے ساتھ دے رہے ہیں انکا | 0 |
|  | 1786 | فوج کو کہا ان کا پشاب نکل جاے | 0 |
|  | 2139 | کھسرے ملک خانہ جنگی کی حالت میں ہے کشمیر میں روزانہ کتنے لوگ شہید ہو رہے ہیں | 1 |
|  | 863 | سزائیں ایسے نہیں مل جاتیں پہلے سانحے ماڈل ٹاؤن کرانے پڑتے ہیں | 0 |
|  | 2206 | الجہاد الجہاد لبیک لبیک کشمیر بنے گا پاکستان انڈیا کو اور مودی کو یہ پیغام دینا ہے کہ… | 0 |
|  | 609 | جہاں رهیں سلامت و خوش رهیں | 0 |
|  | 2226 | اسلام علیکم دعا ہے آپ سب خیر و آفیت سے ہونگے اللہ پاک ہر دن کامیابی نصیب کرے پاکستان زندہ آباد پاک فوج زندہ آباد | 0 |
|  | 3362 | اور کچھ ہوا ہو یا ناں، ایک سال میں ایک کام ضرور ہوا ہے، اب ہر غیر ملکی ائرپورٹ پر پاکستانیوں سے ایک ہی سوال ہوتا ہے… | 0 |
|  | 169 | بہن چود جرنیلوں نے تیرے بابے کے پچھواڑے میں ڈنڈا کیا دیا تی | 1 |
|  | 423 | گاے کا پیشاب پینے والوں سن لو الحمداللہ ھم مسلمان ہیں ھم موت سے ڈرنے والے نہیں ھم موت کو گلے لگاتے ہیں ۔ | 1 |
|  | 1416 | کراچی میں دن دھاڑے بیوروچیف ٹرائبل ٹائمز خلیل الرحمن محسود کو بے دردی سے شہید کردیا گیا ۔ | 0 |
|  | 2239 | لعنت تیری صحافت پر کھوتی کے بچے ثابت کیا کرنا چاہتے ہو خبیث انسان | 1 |
|  | 3381 | آفرین تیری ڈھٹائ اور بیغیرتی پہ خاندانی چور۔ | 1 |
|  | 3427 | ان دونوں رنڈیوں کو بیچ چوراھے میں ننگا کر کے گانڈ پر گولی مارنی چاھٸے تھی | 1 |
|  | 1952 | کھسرا کھسرا ہوتا ہے | 0 |
|  | 370 | یہ بہن چود عوام کے ٹیکس کے پیسوں اور صدقہ خیرات کے پیسوں سے شوکت خانم کی تشہیر کر رہے ہیں | 1 |
|  | 848 | فلم میں یا گانے میں موجودہ کنجروں اور بے غیرتوں سے کوئی امید نہیں حمایت کی بھی | 1 |
|  | 2334 | کبھی ڈاکے مارتے ہوئے دن اور رات میں تفریق رکھی نہیں نا | 0 |
|  | 1794 | سرکاری گٹر سے بڑا تیرا منہ ہے بھگوڑے | 1 |
|  | 103 | یار افغانیوں کی ماں بہن کی چدائی کرنے کا بہت مزہ آتا ہے بہت سفید | 1 |
|  | 2189 | بےغیرتوں کا تخم تو خود ہے بلاول ۔ ذوالفقار علی بھٹو کے نام پر کلنک | 1 |
|  | 318 | بیٹا بلاول گھٹیا ترین ہے باپ بھی تھا اس کا گھٹیا | 1 |
|  | 1358 | بلکل ٹھیک کہا تو نے رنڈی ہمارے سابق وزیراعظم اب تک مودی کی ٹانگیں توڑ چکے ہوتے | 1 |
|  | 1071 | یہ ہے راج ناتھ سنگھ جب بولتا ہے تو ہر لفظ سے ایک ہنسی کا فوارہ چھوٹتا ہے اور یہ RSS کا سب سے پرانا بھگت بھی ہے | 0 |
|  | 1643 | گٹر کی پیداوار بکاوُ میڈیا صرف لفافہ کی زبان سمجھتا ہے۔ لعنت ہے ایسی صحافت پہ | 1 |
|  | 2686 | کچھ لوگ تحریک لبّیک پاکستان کے پیچھے گالی بد دعا کر رہے ہیں ۔۔۔۔۔۔ کیوں کے لبّیک والے حق ب… | 0 |
|  | 2671 | ایسے گھٹیا دو ٹکے کے جرنلسٹ کو قوٹ کرکے پروموٹ نا کرو جناب | 0 |
|  | 1703 | وہ کنجر اور طوائف دونوں آرمی والوں سے ناراض تھے کیوں کنجروں کی عزت چلی گئ تھی | 1 |
|  | 52 | لعنت ہو ایسے کنجروں پہ خود بیچ کر آئیں ہیں اب ڈرامے بازی کر رہے ہیں اپنی بیٹی کو بیچ دیتا کنجر | 1 |
|  | 2598 | اگر اپنی اور خاندان کی بے عزتی کا خیال ہوتا تو کنجروں | 1 |
|  | 1981 | لیکن اس بد کردار جج کے ایک بدکردار فیصلے کی بھی بات کر لیں جس کی وجہ سے ملک کا تین مرتبہ کا منتخب وزیراعظم جیل کاٹ رہا ہے | 0 |
|  | 1472 | اور تیری ماں اور بونڈی ھی رھے گا مادرچود | 1 |
|  | 2151 | انہیں بھی دو دو لگا دو لیکن اس قوم کو بھی دو دو لگنی چاہئے کہ پوڈری سا بندہ اسی بات کو پکڑ کر پوری قوم کا پُھدو کاٹ… | 0 |
|  | 2777 | سوات کالام کے نوجوان مسکین شہید کے قاتلوں کی گرفتاری پر ہم پنجاب حکومت کے شکرگزار ہیں | 0 |
|  | 426 | میں بھی پنجابی سنی جٹ ہوں اگر اب کسی نے چیف صاحب کے بارے میں بکواس کی تو میں نے احتجاجاً شیخ کو آگ لگا دینی ہے | 0 |
|  | 862 | اوئے گانڈو کے جھریوں والے لوڑے، تو پہلے لکھنا سیکھ لے مادرچود، جُگت بعد م… | 1 |
|  | 734 | ہر پیسے والا طاقت کے نشے میں گھٹیا پن پہ اترتا جا رہا ہے اور آپ لوگ بھنگ پی کے سوئے ہوئے ہو | 0 |
|  | 2632 | یہ بات کہ کشمیر میں مسلمانوں کے نسل کشی کے لئے اسرائیل مدد کر رہا ہے مجھ سےہضم نہیں ہوتی۔ اگر ایسا ہونے جارہا ہے تو انتہائی افسوس ناک عمل ہے۔ | 0 |
|  | 672 | کسی اچھے اردو بولنے والے کو ہی ڈھونڈ لیا ہوتا۔ | 0 |
|  | 125 | سیدبلال حسین شاہ کےوالدمحترم وفات پاگئےنمازجنازہ آج دن بجےبستی حسامہ سادات اداکیاجائےگا | 0 |
|  | 2564 | تمہاری موت پوری دنیا کے لۓ عبرت کا نشان ب… | 0 |
|  | 2234 | گل بخاری، تقی، حقانی کالا سور سلمان حیدر اور تیری گانڈ پاکستانی مارے تو بیرونی مداخلت، اور رنڈی کے بچے نواز پشتین نے مودی کو اپنے گھر بغیر ویزہ تیری بہن کے بلاتکار کے لئیے بلایا تھا ؟ | 1 |
|  | 3329 | میرے باپ نے تو تیری امی کی گانڈ بھی ماری تھی وہ تجھے یاد نہیں | 1 |
|  | 1615 | عمران خان۔ تیری ان کا بھونڈا بہن چود۔ کشمیر تیری ابا کا ارے اگر آپ اپنی ماں کی چودہ ہیں | 1 |
|  | 1799 | آج اگر کالا باغ ڈیم بن جاتا تو پاکستان انڈیا کا چھوڑا ہوا پانی سنبھال لیتا مگر اس ملک میں میر جعفر اور میر صادق جیس… | 0 |
|  | 1135 | بہن چود غدار کتے کے پلے اب تیری کےت والی ہو کے رہے گی | 1 |
|  | 1296 | لگتا ہے یوتھیوں کو ٹویٹ سمجھ نہیں آئی دھڑا دھڑ ریٹویٹ کر رہے میری ٹویٹ کو۔ اردو ترجمہ پڑھ لو بچو کھوتی کے بچو | 1 |
|  | 238 | کیا عجب طاقت ہے پاکستان کی ایک ہی دن میں انڈیا جیسی نمک حرام اولاد پیدا کر دی ہاہاہاہا | 0 |
|  | 1903 | تیری بہن کو چود رہا ہوں ابھی پھر تیرے ابے کی گانڈ مار کے تیری ماں کو چودوں گا | 1 |
|  | 2094 | یہ بکواس اور کٹھ پتلی کا پھیلایا ہوا گند اور ٹرک کی بتی کے سوا اس میں کوئ سچائ نہیں | 1 |
|  | 2937 | چھ مہنوں میں ملک کا بیڑا غرق کیا ان بے غیرتوں نے | 1 |
|  | 1379 | ان کا تو کام ہی ’’حاضر سائیں‘‘ اور ’’میرے آقا کیا حکم ہے‘‘ کہنا ہے۔ لفظوں پہ پابندی، بولن | 0 |
|  | 43 | احساس کرنا چاہیے نہ کہ بکواس ۔ | 0 |
|  | 1772 | نہ کتوں کی زبانیں کاٹی جاتی ہیں اور نہ ہی سؤروں کے ہاتھ انہیں سیدھی گولی ماری جاتی | 1 |
|  | 1889 | جس تشدد سے کم عمر بچے کو چوری کے الزام پر قتل کیا گیا وہ قابل مذمت ہے، دل خون کے آنسو روتا ہے ایسے واقعات دیکھ کر | 0 |
|  | 1019 | اگر تمھارے ہاں پِتا ایسے چوتئے کو کہتے ہیں تو سالو بے غیرتو ڈوب کے مر جاوٙ | 1 |
|  | 392 | انڈیا میڈیا آرمی چیف کی اکیسٹینشن پر چیخ اٹھا ،،،، آرمی چیف کی کن خدمات پر انڈیا پریشان ہے | 0 |
|  | 2464 | انکی بکواس بھی روکی جاے | 0 |
|  | 2258 | ھمیں بغیرت کہنے والے تم پر تو دلالی اور بھڑوہ گری کا انٹرنیشنل ٹیگ لگا ہوا کہ ماں بھی بیچ دیتے ہو | 1 |
|  | 283 | اگر کوئی اپنے انتقام کی آگ بجھا رہا ہے تو یقین جایئے اسکی رفتار مایوس کن ہے ایسوں کو تو جتنی جلدی ہوسکے عبرت کا نشان | 0 |
|  | 2734 | پوری پاکستانی قوم اپنی افواج کے ساتھ شانہ بشانہ کھڑی ہے | 0 |
|  | 1998 | مردان۔۔۔ایک اور چارسالہ اقراء درندگی کا شکار۔۔۔ لاش کھیتوں سے برآم… | 0 |
|  | 2441 | اوے کسی آوارہ گشتی کی نسل،تیری ماں نے تجھے یہی دکھایا ہے، م۔چ | 1 |
|  | 34 | جو یہ کہتے تھے کہ خان کو سیاست نہیں آتی وہ آج دیکھ رہے ہیں | 0 |
|  | 2904 | پنڈدادنخان سالہ معصوم بچی سے ریپ کا ملزم چند گھنٹوں میں گرفتار | 0 |
|  | 3358 | گاں دا موتر پین والا مودی ہو یا امریکی چوڑا ٹرمپ ہو یا ہا لینڈ کا گستاخ ان سب کو ایک ہی م… | 0 |
|  | 856 | بلاول کو کہنے والے کتے تم تو کتے کی طرح بکواس کرتا ہے امریکی صدر کی جوتے پالش کرنے والے کتے ہو | 1 |
|  | 3146 | کسی کنجری کے بچے منہ سنبھال کے بات کر جو بھی ہے تُو سامنے آ کر بات کرنے كتی کا پُتر | 0 |
|  | 3350 | اس بہن چود کی گانڈ میں بھی ڈنڈا دو | 1 |
|  | 8 | تیری بہن کی سری گشتی کی اولاد کنجر کی مِس ٹیک جتنا کوجھا ہے اس سے زیادہ تیری زبان گندی ہےتیری زبان کاٹ کر کُتوں کے | 1 |
|  | 3262 | طوائف آج اس بی بی نے تو حق ادا کر دیا پاکستانی ہونے کا اقوام متحدہ میں کھڑے ہوکر جس طرح بھارت کو ننگا کیا اور کشمیر کا | 1 |
|  | 1038 | معافی مانگو یا نہ مانگو تم جیسی رنڈیوں کو تو وہ سبق سکھا کر چھوڑے گا | 1 |
|  | 326 | لگتا ہے چند برس بعد اس معاملے پر بھی سنی شیعہ فسادات ہوا کریں گے کہ وہ آم دراصل چونسہ تھے، سندھڑی، لنگڑا یا دسہری۔ | 0 |
|  | 2683 | میں ایک ملازم پیشہ شخص ہوں۔ میرا کسی SMT سے کوئی تعلق نہیں۔ کبھی کسی جلسہ میں نہیں گیا۔ ووٹ ہمیشہ نواز شریف کا۔ | 0 |
|  | 3177 | عمران خان کے اردو میں بھونکنے والے کتے تم کو پتہ نہیں کہ اس وقت شہبازشریف کدھر ہے | 1 |
|  | 181 | کبھی آپ نے نوٹ کیا کہ ہیرو کو درد ولن کی پٹائی سے نہیں ہوتا محبوبہ کی دوائی لگانے سے ہوتا ہے | 0 |
|  | 2997 | اس بی بی کو کشمیر بھیجنا چاہیے جہاں ہندوستانی فوج اسکی طبیعت صاف کرتے اور ناجانے کیا کچھ کرتے | 0 |
|  | 2074 | تو بہن چود کتی عید پر بھی بکواس کرتی ھو جرنیلوں کی کتی کسی گھٹیا نسل کی | 1 |
|  | 2301 | آج کل میں اپنے آس پاس کچھ اس قسم کے دانشوروں کو دیکھ رہا ہوں جن کو نہ ہی پالِیٹِکْس کے ل کا پتا ہے اور نہ ہی تاری… | 0 |
|  | 445 | تحریک خالصتان میں سکھ برادری اور ہندو برادری آمنے سامنے۔ ایک ہندو ورکر کی زبردست پٹائی، پولیس نے ورکر کو بچایا۔ … | 0 |
|  | 117 | اس بڈھی کی چیخوں پکار مزا آگیا حرام کھانے والے بے غیرت دلے کسی گندے گٹر کی پیداوار | 1 |
|  | 3445 | پاکستان کی بے غیرت اور گھٹیا ترین سیاسی بدمعاشیہ نے پاکستان میں ڈیم ناں بنا کر بھارت کو موقع دیا | 0 |
|  | 3213 | سر یہ آنکھیں انصاف مانگ رہی ہیں خدارا ریحان کے سفاک قاتلوں کو عبرت کا نشان بنا دیں | 0 |
|  | 2281 | کنجروں کے پاس پیسا آجاۓ تو اپنی اوقات بھول جاتے ہیں ۔۔ یا یہ کسی کنجر کی رکھیل ہے یا کسی کنجر کے رکھیل۔کی بیٹی | 1 |
|  | 2625 | اس ظالم حکومت نے ملک کے ساتھ ظلم کیا ہے پلید کتی کو گولی مارنے کی بجا ئے جیل بھیج دیا | 1 |
|  | 1432 | تیری بہن کو چود رہا ہوں ابھی پھر تیرے ابے کی گانڈ مار کے تیری ماں کو چودوں گا | 1 |
|  | 1610 | یہ حقیقت ٹی وی کی آج تک کی سب سے بکواس ٹویٹ ہے | 0 |
|  | 1501 | بکواس ہی کرے کا خبیث انسان | 1 |
|  | 2920 | ان بیغیرتوں میں شرم وحیا کہاں کیونکہ دلوں اور بیغیرتوں کی حکومت ھے | 1 |
|  | 227 | یہ تو سب۔فی میل نام ہیں۔کوئ لنگڑا آم۔کو ئ بنگالی رس گلا۔کوئ سلاد کا کھیرا۔کوئ لمبا بینگن۔کوئ کلو کا سیب مڑا ۔۔ | 0 |
|  | 472 | بے شک اللہ کا انصاف ہونے پہ غرق ہو جاتا ہے | 0 |
|  | 2655 | بالآخر چند لوگوں کو شرم آنی شروع ہو گئی ہے اور ان لوگوں نے اپنی DP black کر دی ہے لیکن ابھی کافی نمک حرام ہیں | 0 |
|  | 1180 | عجیب گندی عورت ہو تم بے شرم عورت اس میں عمران خان کا قصور کہاں۔ سے ہو | 1 |
|  | 1787 | بہترین نیکی ؛ پریشان حال کی مدد کرنا ہے۔۔ | 0 |
|  | 2340 | میر علی ، جہاں پہ یہ افسوس ناک سانحہ ہوا ہے، غیر ملکی دہشتگرد گروہ، حقانی نیٹ ورک کا ہیڈکواٹر تھا | 0 |
|  | 3493 | یہ کیسے مرد ھے جو سب کچھ عورت پر ڈالتے ہیں منی لانڈرنگ بھی عورتوں سے کرواتے ہیں جائدادیں بھی انہی کے نام رکھواتیں ہ… | 0 |
|  | 158 | ہندووں کی راکھیل | 1 |
|  | 130 | وہ کہتے ہیں نا کہ ایک طوائف نے اپنی آپ بیتی لکھنے کی کیا سوچی کہ شہر کے سارے شریفوں نے خودکشی کر لی۔ | 0 |
|  | 744 | دلّے کُتے حرامی تیری بھین نوں L | 0 |
|  | 2870 | پتوکی جلسے میں نواز شریف کے شیر نے سٹیج پر آپ کی گانڈ میں اُنگلی دی | 1 |
|  | 961 | کشمیر کا سودا تم نے کروایا ہوگا دلالی کر کے ۔۔کیونکہ تم لوگ تو دلال ہو شروع سے دلا ہاراں والے کی نسل سے | 1 |
|  | 1035 | کنجروں کے سر پر سینگ نہیں ہوتے | 1 |
|  | 2593 | بابا جی کہتے ہیں پتر آجکل کے دور میں وہی انسان ثابت قدم رہتا ہے جو ہر کسی کی بکواس کو دل پے نا لے کیونکہ آجکل ہر دو… | 0 |
|  | 1239 | کتی کا بچہ بڑا سائنسدان کتے کے اگے بین بجانے کا کیا فائدہ | 1 |
|  | 3029 | سنا ہے افغانستان میں بھارت کے سفارت خانے پر حملہ سے زیادہ کتے مر چکے ہیں | 0 |
|  | 2902 | اس ملک میں ہر چیز بکاؤ ہے سوائے آم ہے۔ شکریہ محسن پاکستان لنگڑا شریف | 0 |
|  | 1321 | کوئی اس جاہل کو بتا دیں کہ کراچی اور شملہ معاہدہ کے تحت ہم مسلۂ کشمیر کو عالمی عدالت میں نہیں لے کر جا سکتے | 0 |
|  | 3147 | بھٹی تو اپنی بہن مودی کو بھی پیش کر دے جیسے تو نے نیازی کو کی ھوئی ھے تو وہ تیری بھی کال اٹنڈ کر لے گا دلے | 1 |
|  | 885 | لو جی پاک آرمی نے تتہ پانی سیکٹر فائرنگ کے جواب میں انڈین جہنم واصل کر دئے جن میں ایک افسر بھی شامل۔۔۔ زخمی اور… | 0 |
|  | 1700 | السلام و علیکم ہماری ایک اردو پوائنٹ کی میڈیا اینکر ھے جس کا نام کنول آفتاب ھے اتنی گھٹیا اور گندی لڑکی ھے | 0 |
|  | 1437 | تو گدھے کا بچہ ہے مادرچود | 1 |
|  | 1863 | اور ان لوگوں کے مریم کے میڈیا ونگ کے بارے میں کیا کیا فرضی بکواس نہیں کی | 0 |
|  | 3001 | کشمیریوں پر ظلم مزید برداشت نہیں کر سکتے بطور پاکستانی یہ ہمارے منہ پہ ایک طمانچہ ہے اور ہماری سماجی حالت پہ بدترین دھبہ بھی | 0 |
|  | 494 | دو کنجروں کی آپس میں conviction ھو رہی ھے ایک رو رہا ھے دوسرا حوصلہ دے رہا ھے | 1 |
|  | 626 | تجھے ان دونوں حرامیوں کی بہت فکر ھے؟ | 1 |
|  | 1790 | کشمیر بیچ کے اب وطن پہ ڈرامہ بازیاں کررھے ھیں ب چ کتے سؤر خنزیر | 1 |
|  | 479 | تتہ پانی سیکٹر، ہندوستانی فوج کی گولہ باری سے سالہ لڑکے سمیت شہری شہید۔ | 0 |
|  | 2177 | لوگوں کو بھاشن ایسے دیتے جیسے خود فرشتے ھے | 0 |
|  | 859 | خبیث انسان یہ وہ عورت نہیں ھے جھوٹے اور مکار انسان ضمیر فروش کشمیر فروش | 1 |
|  | 1724 | جی بالکل ٹھیک کہہ رہے ہیں دنیا بدل گئی ہے حالات بدل گئے ہیں | 0 |
|  | 236 | بیشک اللہ جسے چاہے عزت دے اور جسے چاہے ذلت دے | 0 |
|  | 2425 | انڈیا میں کوئی ایسی سیاسی جماعت یا ٹی وی چینل نہیں جو اپنی فوج یا راء کےخلاف بکواس کرے ایسی گندی نسل صرف پاکستان میں ہی پائی جاتی ہے | 0 |
|  | 1846 | ووٹ صرف ان بےغیرتوں کو تن کے رکھنے کے لئے دیا ہے | 0 |
|  | 1511 | کتو غدارو کان کھول کر سن لو | 1 |
|  | 2914 | یہ ٹویٹ ان لوگوں کے منہ پر طمانچہ ہے جو کشمیر معاملے پر سعودی عرب اور دیگر عرب ممالک کی طرف دیکھتے ہیں | 0 |
|  | 1788 | انگریزی کی ایک کہاوت ہے۔جس کا ترجمہ یہ ہے | 0 |
|  | 1927 | اب تیری گانڈ مارنے کا ٹائم آنے والا ہے تھوڑا انتظار کرو کنجروں کی ترجمانی کرنے والی گشتی تیری اوقات | 1 |
|  | 1388 | ایسے مستقل مزاج بندے کے ہاتھ نمبر لگا ہے بیسوں نمبر بلاک کر چکی پھر نئی سم سے جان کھانی ہوتی اب سم ہی بدلنی پڑنی ️ ️ ️ | 0 |
|  | 1959 | ذلت کے پچاس سالوں سے بہتر ہے۔ عزت کا ایک سال | 0 |
|  | 2149 | چور تو نہ لکھو اتنی پٹائی پر تو میں بھی ماں لیتا روپے میں نے لئے ہیں | 0 |
|  | 1881 | یہ بھی بیغیرتوں کی نسل میں سے ہے کیا | 1 |
|  | 357 | اس بے غیرت کی حرکت کو نظر انداز نہ کریں۔ اس نے بہت شاطر چال چلی ہے | 1 |
|  | 2865 | یوتھیو دیکھو اور کان کھول کر سنو یہ کتا نیازی کیا بکواس کرتا رہا ہے ماضی میں پاک فوج کے خلاف | 0 |
|  | 2372 | گیس کابل کیوں ادانہیں کیا ڈی سی آفس بہاولپور کا کنکشن کاٹ دیا گیا | 0 |
|  | 2511 | یہ ان بیغیرتوں کو نظر نہیں آتا جو فوج کے خلاف بکواس کرتے ہیں | 1 |
|  | 2793 | مادرچود | 1 |
|  | 2537 | سارے گٹر منہ والے وزیر اور مشیر سُن لو اپنے تھوک کہ چاٹنے والے سیلیکٹڈ نیازی کی بکواس ، تم لوگوں کو کس چیز سے خطرہ… | 0 |
|  | 2770 | فوج بری ہے نا تو لوٹا دو یہ سب بیٹے ان کی ماٶں کو لوٹا سکتے ہو اگر اپنے بیٹے نہیں دے سکتےتو دھرتی کے ان بیٹوں کو گالیاں نا دو | 0 |
|  | 473 | اسان طریقہ یہ کہ ان سے پاکستان زندہ باد کے نعرے لگوائیں اور جس نے جندہ باد لکھ کر جواب دیا سمجھو | 0 |
|  | 430 | تم اور تمہارا زانی لیڈر یہ چوتیا کسی اور کو بنانا، بہن چوُد دنیا جہان کے جھوٹے انسان | 1 |
|  | 381 | بہن چود ہر جگہ پر اپنے غلامی کا ثبوت دیتے ہوں | 1 |
|  | 3056 | وڈے بیغیرت بلکہ سردار ہو بیغیرتوں کے | 1 |
|  | 3389 | بہن چودو کس نے بولا تھا ملک لوٹو اب دھاڑے مارو مادرچود دو | 1 |
|  | 1122 | کوئی اس فحاشہ عورت کی تو اسکی سروس بحال کرواؤ یہ بیچاری بھونک بھونک کہ پاگل ہی نہ ہو جاۓ | 1 |
|  | 2488 | اطلاعات کے مطابق آزاد کشمیر کے مختلف سیکٹر پر بهارتى فوج کی گولا باری جانى نقصان کى بهى اطلاعات | 0 |
|  | 589 | ہمارے مارکیٹیں تم نے تباہ کیے ہمارے مساجد گرا دیے ہمارے سکول کالج تباہ کر دیے ۔۔ | 0 |
|  | 280 | انشاء اللہ کروائیں گے کشمیر آزاد | 0 |
|  | 2620 | آزادی اظہارِ رائے کے دعویدار بھارت کے منہ پر زور دار طمانچہ بھارت نے مقبوضہ کشمیر میں دو فوٹو جرنلسٹ شہید کر دیئے… | 0 |
|  | 3469 | بہن چود تو سبوت دیکھ کر آیا ہے دلے مادر چود کھوتے | 1 |
|  | 1970 | اللہ تعالی بےغیرتوں کو تباہ کرے | 0 |
|  | 516 | زلیل و گھٹیا شخص ہے یہ | 1 |
|  | 1350 | سب مادرچود ہیں کسی کو کشمیر کی فکر نہیں | 1 |
|  | 3278 | مشاہد اللہ جیسا گھٹیا انسان پورے پاکستان میں نہیں دیکھا۔۔کشمیر کے معاملے میں بات کرنے کیلیے آیا تھا یا اجلاس میں گالیاں دینے۔ خبیث | 1 |
|  | 2470 | لعنت ھے تیری بزدلانہ سوچ پر گیدڑ کی طرح زندگی گزاروں اور کتے کی موت مرو بے غیرتوں | 1 |
|  | 1005 | لعنت ہی کافی ہے تجھ پر تم کو وہ وقت بھول گیا جب تم آدھی ننگی ہو کر پوری دنیا میں ٹی وی پر نظر آتی تھی | 1 |
|  | 1142 | يا اللہ جو بھی حکمران رہے، ان معصوم بچوں کا حساب ان سے ليں، چاھے وہ وزير اعظم تھا يا کونسلر ان سبکو اسی جہان ميں ع… | 0 |
|  | 2100 | بکواس نہ کر کتے | 1 |
|  | 2444 | بہن چود کتا بندہ کتی بات ہی کرے گا۔ سالے تیرے گھر بھیج دیتے ہیں | 1 |
|  | 1446 | اپنا منہ بند کر دو منہ والی سپنی | 1 |
|  | 2971 | انڈین وزیر داخلہ کو ایٹم بم چلانے کا شوق ہوا ہے یہ کھوتا سمجھ رہا ہے کہ نواز شریف کی حکومت ہے | 1 |
|  | 1031 | ایک بغیرت کہتا تھا یہ کون ہوتے ہے ہمارے معاملوں میں داخل اندازی کرنے والے | 0 |
|  | 2966 | احتساب مذاق ہے | 1 |
|  | 2088 | یہ ملائشیا نے ماسٹ key دی ہے | 1 |
|  | 1436 | عید کا دوسرا دن حکومت کا دوسرا لطیفہ صدر مملکت اور وزیراعظم نے قوم کوعید سادگی سے منانے کی ہدایت | 0 |
|  | 2267 | غلط بات شہباز شریف نے دورانیہ کا زکر نہیں کیا اور بغیرت کے بچو وہ سن سے پہلے کی بات کررہا تھا | 1 |
|  | 2805 | لیکن پھر بھی پاکستان ترقی نہ کر سکا اور اب تو معاشی طور پر بہت ہی گہری کھائی میں جا گرا ھے | 0 |
|  | 667 | اس میں آپ کا کوئی کارنامہ نہیں ھے تمام ترقیاتی منصوبے ٹھپ پڑے ہیں ملک میں کوئی نئی انڈسٹری قائم نہیں ہو رہی | 0 |
|  | 1645 | میں پنجابی راجپوت فیملی سے تعلق رکھتی ہوں اور ہمارے ہاں تو ہمارے پُرکھوں میں بھی ایسی کوئی رسم نہیں ہے | 0 |
|  | 669 | میڈم بہت گھٹیا کردار ادا کر رہے ہیں اور تاریخ میں ان کا کردار دیکھ کر لوگ میر جعفر میر صادق بھول جائیں گے | 0 |
|  | 1567 | اللہ پاک اس کی شہادت قبول کرے اور انڈیا یاد رکھنا میرے رب کی پکڑ اتنی مضبوط ہے جس میں تم آ چکے ہو پوری دنیا میں ع… | 0 |
|  | 1899 | بہت ہی کوئی حرامی نسل ہیں یہ پٹواری کوئی موقع ہاتھ سے جانے نہیں دیتے | 1 |
|  | 2717 | جنرل آصف غفور کا ایک ٹوئٹ ساری ہندوستانی فوج موتر پینے والے نیتاؤں کی نیند حرام کرنے کے ساتھ انہیں دن میں تارے دک… | 0 |
|  | 2851 | مادرچود صحافیوں کے نام پہ دھبہ ہے تو | 1 |
|  | 2542 | اس کُتے ارشاد بھٹی سے پوچھیں کیا تجزیہ کار کو غیر جانبدار ہونا چاہئے یا نہیں۔ اس جیسے گندے اور بکاوُ صحافی سے | 1 |
|  | 3415 | دلوں خنضیروں کو آپنی اوقات کا پتہ چل گیا ھے آب ان کی لاشیں گرنی نہیں رکیں گی ھر جگہ کنجروں کی لاشیں ھی لاشیں ھوں گی | 1 |
|  | 3294 | پتہ نہیں توازن کی کیفیت اتنی کمیاب کیوں ہے کہ افراد میں یا تو عقلی شکوک کے ڈھیر لگ جاتے ہیں اور یا ایمان کے | 0 |
|  | 6 | ماں سے پوچھ کتی کے بچے، کس گشتے سے مروا کر آئی تھی، تو یہ گشتی تیری بہن پیدا کی | 1 |
|  | 60 | قوم کو تبدیلی کا منجن بیچنے والے کنجروں کے علاوہ سب کو عید مبارک | 1 |
|  | 1769 | دیوار میں چنوا دیں کیا؟ | 0 |
|  | 1387 | بیغیرتوں کا احتجاج کرنے کا اعلان | 0 |
|  | 2487 | وہ اسلیے کہ اسرائیل جانتا ہے کہ اسلامی ممال… | 0 |
|  | 524 | جنکا لیڈر جھوٹا ہو اسکے کارکن بھی جھوٹے ہونگے | 0 |
|  | 2246 | ایک آصف غفور سب انڈین فوج پر بھاری | 0 |
|  | 3092 | ان بیغیرتوں نے تمھیں کتنے کا بیچا کہا ؟ | 0 |
|  | 1058 | یے بھٹی واقعی بیغیرتوں کا پلانٹڈ بندہ ھے۔۔اس کے انداز تکلم سے ھی پتہ چلتا ھے یے صحافی نہی دلا ھے۔ | 1 |
|  | 2148 | بہت جلد دنیا کو ایک عبرت ناک منظر دیکھنے کو ملے گا۔ | 0 |
|  | 2917 | کتی کے بچے قوم کو گمراہ کرنے والے بھڑوے وطن فروشوں کے بوٹ چاٹنے والے تمہارا انجام دنیا عبرت کے طور پر یاد رکھے گی | 0 |
|  | 3057 | بے شرم بے حیا | 1 |
|  | 3292 | چل ہٹ دفعہ ہو کسے کتے کی بچی ملک کے حالات خراب ہیں کشمیر جل رہا ہے اور تجھے سیاست اور اپنے کتے باپ کی فکر ہے | 1 |
|  | 166 | بہت ہی کوئی حرامی نسل ہیں یہ پٹواری کوئی موقع ہاتھ سے جانے نہیں دیتے | 1 |
|  | 904 | عمران نیازی کی مثال اس بندر کےجیسی ھے جو سال تک گاجروں کے اوپر پیشاب کرتا رہایعنی ھرحکومت پر بھونکتا رہااور جب خو… | 0 |
|  | 41 | آج اس سالی موٹر نے سابت کر دیا کے گٹر کی پیداوار ہے غدار کی گندی اولاد ھی نکلی میرے سامنے آ جائے تو اس اوارہ عورت | 1 |
|  | 2182 | ایک تو اسی ڈر سے کشمیر بھاگ گیا ہے کاش کراچی کی عوام ان بیغیرتوں کا گلا پکڑ سکے لیکن وہ بھی برابر کے ذمہ دار ہیں | 0 |
|  | 674 | جاو مادرچود یہ ملک کا بیڑا غرق کردیا تم لوگوں نے | 1 |
|  | 2089 | افسوس ناک واقعہ مردان جانباز میں چار سال بچی گھر سےلاپتہ بچی کی مسح شدہ نعش کھیتوں سے برآمد… | 0 |
|  | 2057 | بکواس نہ کر بے کھسری | 1 |
|  | 606 | یہ بھارت، ممبئی کی تصویر ہے، دیکھیں بارش کے بعد کا منظر کیونکہ وہاں ڈرین سسٹم ہیں اور سڑکیں کھلی ہیں | 0 |
|  | 1211 | تُم بیغیرتوں کی دوزخ میں رہو | 0 |
|  | 1933 | کیا گدھا راگنی شروع کی ہوئی ہے آپ سب گدھا خوروں نے، گدھی کے بچو | 0 |
|  | 2390 | مادر چود رنڈی | 1 |
|  | 3308 | اس ظالم حکومت نے ملک کے ساتھ ظلم کیا ہے پلید کتی کو گولی مارنے کی بجا ئے جیل بھیج دیا | 0 |
|  | 3311 | دنیا گول نہیں مادرچود ہے | 1 |
|  | 3078 | باجوڑ افسوسناک ایکسیڈنٹ پر دل خون کے آنسو رو رہا ہے۔ | 0 |
|  | 275 | جا او کتے بچے سور کی اولاد گانڈو بہن چود | 1 |
|  | 1588 | نازی مودی کی عمران خان کو جواب دینے کی ہمت نہ ہو سکی البتہ کل ٹرمپ کو کال کر کے شکایت لگا دی کہ عمران خان مجھے ہٹلر… | 0 |
|  | 2541 | تم بکنے والے کتے ہو لفافہ صحافی | 1 |
|  | 780 | بھونک مادرچود بھونک | 1 |
|  | 2476 | واہ اوئے چوتیوں مودی بغیر تھوک لگائے تمہاری گانڈ مار رہا اور تم اب بھی پتہ نہی کونسی امیدیں لگائے بیٹھے ہو | 1 |
|  | 1994 | ن لیگ ھر معاملے پر بولتی ھے بس شریفوں کی جائدادوں کا کوئی جواب نھیں | 0 |
|  | 2424 | اس نے کیا تمہاری گانڈ ماری ہے اتنی غیرت دکھاؤ اور وہاں جا کر لڑو بےغیرت | 1 |
|  | 2211 | ان بے شرموں غداروں اور بیغیرتوں کا منہ کالا کر کے گدھے پر بیٹھا کر پورے پاکستان کا چکر لگوانا چاہیے | 0 |
|  | 541 | پکڑ کر اس کی گانڈ مارے نیب صرف گانڈ پھاڑے مار مار کر اور مریم صفدر کی چیخیں نکلوائے | 1 |
|  | 1935 | جو بے گناہ اس کی سزا کاٹ رہا ہے وہ اس کا کیا گناہ ہے | 0 |
|  | 2313 | جنرل قمر جاوید باجوہ سال کیلئے | 0 |
|  | 3439 | لندن میں کشمیریوں اور سکھوں کے ہاتھوں و بھارتیوں کی پٹائی بھارتی بھاگنے پر مجبور ہوگئے | 0 |
|  | 3218 | ایک پٹھان بال کٹا کر آیا بیوی اتنے چھوٹے کیوں کٹا دیے پٹھان اس کے پاس بقایا نہیں تھا تو میں نے کہا کے اور کاٹ دو | 0 |
|  | 379 | بھارتی کتوں کی ہماری فوج کے ہاتھوں تو پٹائی ھو ہی رہء ھے اب تو پوری دنیا میں انکو جوتے پڑ رہے ہیں | 1 |
|  | 3013 | ہمیں اپنے وطن سے بہت پیار ہے پاکستان کے سب غداروں، دشمنوں چوروں لٹیروں اور ان کے ساتھیوں کو چوک پر لٹکا کر عبرت کا… | 0 |
|  | 1405 | یہاں انصاف اور قانون والوں کو سچ بولنے لکھنے والوں کو طاقتور قوتیں منفی ہتھکنڈوں ڈر خوف دہشت ویڈیو اور فائلیں | 0 |
|  | 1862 | آپ تے م ک ل بہن پیش کر دی ھے لیکن تیری غیرت تے تیری بنڈ وچ وڑی ھوئی ھے | 1 |
|  | 2695 | بہن چود کشمیر میں رینجر کہاں سے آ گئ | 1 |
|  | 1961 | درفٹے منہ | 1 |
|  | 615 | دلے تو پہلے اپنے باپ سے پوچھ کہ تو اسی کے نطفے سے ہے یا پڑوسی کے چوتیا انسان مزائل نواز شریف نے بنائے | 1 |
|  | 2578 | جناب بات ذرا منہ سنبھال کے کریں میرا ان سے کوئی جھگڑا نہیں ہے | 0 |
|  | 340 | لعنتی کتا،خبیث کی نسل، نیازی کا پالتو کتا، انسان نما بندر تم شرم کرو یار ۔۔۔تمھاری بکواس سے لوگ تنگ آۓ | 1 |
|  | 1731 | بہت بڑا بہن چود ہے تُو بھوسڑی کے بندر کی اولاد | 1 |
|  | 2259 | یہ میں کیا نیوز سن رہا ہوں بھارت سے بغاوتوں کا سلسلہ جاری یہ تو ہمیں ٹکڑے کرنے چلے تھے اور خود ہو رہے ہیں۔ | 0 |
|  | 929 | مریم صفدر اعوان المعروف بھگوڑی | 1 |
|  | 3266 | پاکستان کی صحافت کا مہا حرامی ٹٹے چُک مادرچود | 1 |
|  | 3320 | مریم کی عیاشی کا نمونہ جنید صفدر حرامی سات ارب کا | 1 |
|  | 2634 | عمران خان وہ بہادر و دلیر لیڈر ہے جس نے اسمبلی کے فلور پر کھڑے ہو کر پہلی دفعہ اسرائیل اور پھر انتہاء پسند تنظیم RS… | 0 |
|  | 2453 | در اصل ملالہ کو گولی لگی ہی نہیں گولی تو دوسری بچی شاید نام اس بچی کا شازیہ تھی | 0 |
|  | 2378 | سب مادرچود ہیں کسی کو کشمیر کی فکر نہیں | 1 |
|  | 2126 | بے شک الله کا انصاف ہونے پہ غرق ہوجاتا ہے | 0 |
|  | 2367 | اسکی طبیعت صاف ہوجائے گی یہ پیسے کا نشہ ہے جو سر چڑھ کر بول رہا ہے | 0 |
|  | 1760 | ہجڑے کو ہجڑا نہ کہوں تو کیا کہوں۔ حرام کی اولاد کو حرامی نہ کہوں تو کیا کہوں | 1 |
|  | 303 | گندے باپ کی گندی اولاد نے آج ایک بار پھر وزیراعظم کے خلاف گندی زبان استعمال کی ہے ۔ ہم انشاءاللہ ان حرامیوں کو عبرت کا نشان بنائیں گے | 1 |
|  | 415 | ترس گئے ہیں ہم تیرے لب سے کچھ سننے کو | 0 |
|  | 492 | اندرونی و بیرونی بے غیرتوں کی بکواس شروع ہونے کو ہے۔ کیونکہ یہ فیصلہ ملک دشمنوں کے لئے موت ہے | 0 |
|  | 2183 | گل مک گئی اے ۔ ہون بکواس کرنا ضروری سی یا عادتوں مجوراو | 0 |
|  | 2944 | یہاں کے تاجر کو آپ زندہ درگور کر رہے ہو۔ | 0 |
|  | 483 | سلامتی کونسل میں کشمیر کی قرارداد پچاس سال بعد پیش کرنے والے پاکستانی مثبت میڈیا کے منہ پر طمانچہ رسید کر دیا ہے | 0 |
|  | 2229 | جب بھٹو کا نواسہ | 0 |
|  | 2723 | افسوس ناک ہے مگر اچھا سبق ہے۔ | 0 |
|  | 3376 | لنڈے کے ارسطو | 1 |
|  | 3274 | دلے تو پہلے اپنے باپ سے پوچھ کہ تو اسی کے نطفے سے ہے یا پڑوسی کے چوتیا انسان مزائل نواز شریف نے بنائے | 1 |
|  | 119 | خان تیری گانڈ بھی مارے گا اور تجھے پتہ بھی نہی چلے گا گھٹیا عورت۔ | 1 |
|  | 2053 | اور جتنی بے عیرتی ہوئ ہے اور ملک کو تباہ کیا ہے اس کے ز مدار پی پی پی اور بد کردار شیلا رضا بے غیرت ہیں | 1 |
|  | 1609 | جنرل باجوہ صاحب کی extentension سے محب وطن خوش جبکہ مودی لیگ اور نونی لیگ بکواس کررہے ہے | 0 |
|  | 1602 | تم مادرچود ہو | 1 |
|  | 908 | نیازی خان کشمیر کو بھارت سے آزادی دلوائےگا یہ نعرہ وہ لوگ لگا رہیں ہیں جنہیں یہ یقین تھا کہ کپتان خودکشی کر لے گا م… | 0 |
|  | 2824 | مادر چود یہ مت سمجھنا کہ توں بکواس کرے گا تو میں تیری بہن کو چھوڑ دوں گا | 1 |
|  | 341 | نریندررمودی قصائی دھشت گرد مکار بے بےغیرت اور آسکی فوج کنجروں کی اولاد ہیں نہتے کشمیری اورعورتوں پر حملہ کرتی ہیں | 1 |
|  | 373 | سالے کروڈ ہونے کے دعوے دار تو ہجڑے نکلے مقابلہ نہی کرسکتے | 1 |
|  | 2544 | سلیکٹڈوزیراعظم کا ایک سال پاکستانیوں کیلٸے ذلت کا سال، اذیت کا سال، رسواٸی کا سال ھے | 0 |
|  | 1833 | انکا شرم سے کوئی تعلق نہیں ہے۔ تھر میں ہی بچے مر رہے ہیں۔نہ۔انکو کیا لینا دینا۔ انکا گاڈ فادر جیل میں ہے | 0 |
|  | 101 | یار جو لوگ آپ سے اختلاف کرتے ہیں ان کو بھی زندہ رہنے کا حق دیں یہ ریاست صرف آپ کے باپ کی جاگیر نہیں… | 0 |
|  | 2330 | بھونک مت تو دلی کی اولآد | 1 |
|  | 76 | کسن نے تیری بہن کو ڈی چوک میں ننگا کیا تھا اب فیصلہ کر کس کو لئے کر جائے گا | 1 |
|  | 2895 | تم جس طرح بھی بھونکتے رہو۔لیکن خان نے تمھاری گانڈ ماری ہے | 1 |
|  | 2518 | مودی کے بھارت کے پاس ایٹمی ہتھیار کس طرح علاقائی امن کے لئے خطرہ ثابت ہو سکتے ہیں | 0 |
|  | 3007 | مریم کے لئے رونے والے کراچی میں لوگوں کو زندہ درگور کرنے والوں | 0 |
|  | 2181 | ٹکے میں بکنے والی کھسری غیرت کی بات کرتی ہے | 1 |
|  | 2641 | انتہائی اعلیٰ گھٹیا اور کچرا نیٹ ورک telenor ہے | 0 |
|  | 2141 | شکل سے ھی بدمعاش لگتی گشتی کی پتر ۔۔ خرامزادی | 1 |
|  | 1560 | تیری گانڈ مارے بندہ اور رونے بھی نہ دے | 1 |
|  | 2560 | آئندہ طاھر مغل کو ھیرا منڈی کا دلال سمجھا جائے کیونکہ یہ شکل سے لگتا بھی ھے بہن چود | 1 |
|  | 2214 | لعنت تم کنجروں اور حرامیوں پر جو تم جیسے گٹری کیڑوں کی وجہ سے پاکستان پر مشکلات آرہی ہیں | 1 |
|  | 1256 | گلگت ہیلی پیڈ ایف سی این اے میں جشن آزادی آزادی کی تقریب کے دوران دیوار گرنے سے افراد دب گیے | 0 |
|  | 1013 | اس کا مطلب ہے تیری ماں کیسی کتیا کے بچے لعنتی بے غیرت چوتیا مادرچود دھلے | 1 |
|  | 1288 | لعنت ہے کنجر حکمرانوں پر جو خاموش ہیں کل جب انکی بہنوں پر ہاتھ آئے گا تب انکو اس تکلیف کا اندازہ ہوگا | 1 |
|  | 884 | بہت ہی کسی گھٹیا قسم کے بیغیرت ہیں | 1 |
|  | 1516 | ابے بے غیرت حرامی اولاد تمہاری بہن کے لئے ہجڑا ذلیل ہورہا ہے اور تم تماشا دیکھ رہے ہو دیکھنے سے یاد آیا گیراج میں دی | 1 |
|  | 1671 | پاکستانی حجاج کرام سرکاری حج سیکم کے تحت ناقص کھانے کی فراہمی پر سراپا احتجاج | 0 |
|  | 602 | دراصل یہ کنجروں اور رنڈیوں کی پارٹی پیپلزپارٹی کراچی والوں سے اپنی دشمنی نکال رہی ہے | 1 |
|  | 386 | کیسا خبیث انسان ہے خود کا بھی بتا دیتا خود کس کا ڈسا ہوا ہے | 1 |
|  | 823 | ہم نےتو سیاست شروع ہی تب کی ھے | 0 |
|  | 1496 | ایرن کا بھی موقف سامنے آگیا کیتے ہیں کہ ہم بھی کشمیری مسلمانوں کے لیے پریشان ہیں | 0 |
|  | 1741 | حرامی پن ہے بھیإ نیازی اس ہی لائق ہے اس کو جتنی زیدہ گالیاں ملیں کم ہیں لوگ سخت مصیبت میں ہیں اور اس کو حرامی پن سوجھی ہوئی ہےاس لعنتی کا حرامی پن چیک کریں | 1 |
|  | 455 | ہاہاہاہا ۔ یہ انڈیا والے بڑے بہن چود کے پُتر ہیں۔ ھماری پاک فوج کے ٹٹو کی اینی بستی | 1 |
|  | 1885 | تیری ماں علیمہ رنڈی عیاشیاں کر رہی ھے شیخوں کے ساتھ | 1 |
|  | 2327 | زلیل و رسوا کرے تو سبق یہ سیکھا | 0 |
|  | 917 | سوشل میڈیا پر اپنے اپنے مفادات کے لئے کچھ سیاسی و غیر سیاسی طبقوں کے پالتو معزز شہریوں پر سرِ عام بھونکتے ہیں | 1 |
|  | 137 | کوئیٹہ کچلاک دینی مدرسہ کے مسجد میں نماز جمعہ کے بعد بم دھماکہ متعدد افراد شہید درجنوں زخمی | 0 |
|  | 1062 | خار باجوڑ کا ایک موٹر کار کاغان جاتے ھوئے سین میں جاگری ھے جسکے نتیجے میں چار جوان جان بحق ھو چکا | 0 |
|  | 2283 | انتہائی بکواس قسم کی سروس ہے پی آئی اے کی۔ چائینہ سے آو گے تو زلیل وخوار ہوگے۔اوپر سے سامان کی ٹینشن الگ سے | 0 |
|  | 872 | گھٹیا ملک گھٹیا لوگ | 1 |
|  | 2321 | کتا ڈرامے بازی کر رہا ہے سندھ کا بیڑہ غرق کر دیا ان کنجروں نے | 1 |
|  | 2319 | وہاں تیری ماں سیتا وائیٹ کو بھی چودتا رہا ہے بہن چُود انسان کُتی کے بچے شرم نہیں آتی ابھی بھی اپنی ماں | 1 |
|  | 198 | بارڈر پر تمام کشیدگی ختم، انڈیا نے ہتھیار ڈال دئیے اور یوں جمہوریت جیت گئی اور پورے پاکستان کو کشمیر کی آزادی مبارک ہو | 0 |
|  | 650 | مىں تىرى بهن کا ىار هوں تىرى ماں کا ٹھوکوں هوں مادر چود | 1 |
|  | 542 | یہاں تو اب کتے بھونک بھی رھے ہیں اور کاٹ بھی رھے ہیں | 0 |
|  | 2796 | انور لودھی پیدا ہی گناہ کمانے کیلئے ہوا ہے لفافہ کا نہیں پتا کس کس کو ملتا رہا ہے | 1 |
|  | 1858 | نئ یقین کرنا اور کچھ ؟ دفع ھو جاؤ خبیث انسان تم | 1 |
|  | 1624 | یوتھیوں سے بڑا کوئی جاہل ہو ہی نہیں سکتا | 0 |
|  | 3000 | پاکستان پہلے ایک غریب ملک ہے ۔۔ اوپر سے یہ انڈیا کی وجہ سے دفاع پہ پاکستان کو بے تحاشہ پیسے خرچ کرنا پڑ رہے ہیں جو… | 0 |
|  | 618 | اگردس چلتےتھےتوآدھےبچتے تھے۔خاندان کےبہت سےبچھڑ گۓ۔نانو جان صرف ایک چاندی کا ہارتھاجو ساتھ لاٸ باقی سب خالی ہاتھ۔ | 0 |
|  | 3142 | آج اس سالی موٹر نے سابت کر دیا کے گٹر کی پیداوار ہے غدار کی گندی اولاد ھی نکلی میرے سامنے آ جائے تو اس اوارہ عورت کو بتا دوں | 1 |
|  | 3432 | جی بیٹا میں تماری گانڈ ہم نے بہت اچھے طریقے سے ماری تھی | 0 |
|  | 3138 | یا اللہ پاک میرے گراں قدر لیڈر میاں شہباز شریف صاحب کو صحت و شفاء کاملہ عطا فرمائے | 0 |
|  | 232 | اللہ کرے یہ اچهے صحافی جلد مر جاہیں تاکہ جلد جنت میں جاہیں اس سے پہلے کہ یہ حرامی ھو جایں | 0 |
|  | 1584 | لعنت ہے اگر ایک اور نمک حرام شامل ہوگیا مبارک ہو نونیو واقعی غلامی تمہارا مقدر ہے | 0 |
|  | 2654 | حقیقت ٹی وی والے نے پھر علماکرام کے خلاف بکواس کردی ـ | 0 |
|  | 304 | سندھ حکومت نے صوبے کا خاص کر کراچی کا بیڑا غرق کر دیا ہے۔ | 0 |
|  | 641 | بہن چود سارے ملک تباہ کر گیا | 1 |
|  | 2543 | یہ پوری حکومت کنجروں اور رنڈیوں کی ہہ | 1 |
|  | 2195 | ہم سب کو الله سے توبہ کرنی چاہیے اور کبھی کوئی بڑا بول نہیں بولنا | 0 |
|  | 279 | اور تم جیسے کتوں کی پھانسی سے پہلے گانڈ بھی ماری جائے | 1 |
|  | 3466 | فطرت برحق کی طرف سے اسلامی پاکستان کے قوم فروش جرنیلوں کے حرامی پن پر انسان نماء ایک کھوتے نیازی کو اس منافق | 1 |
|  | 1074 | کون کُتے کا بچہ کہتا تھا کہ تم سال کے لیے آتے ہو اور ہم نے ہمیشہ رہنا ہے اینٹ سے اینٹ بجا دیں گے | 1 |
|  | 539 | بہن چود اے کی ایٹم اے بندہ | 1 |
|  | 1855 | اب کیا کرنے لگے ہیں مادرچود | 1 |
|  | 468 | قصئہِ مختصر بلاول جیسے نابالغ ہمارے لیے کُتے کے پِلے کا درجہ رکھتے ہیں | 1 |
|  | 2525 | جب پورا سال کام نہ کرو اور زلیل ھوتے رہو تو عید کے دن ایسا کرنا پرتا ھے جناب نمبر بنانے کے لئے | 0 |
|  | 3342 | مارخور زندہ باد | 0 |
|  | 3140 | جنگ موتہ میں ایک لاکھ کفار کے مقابلے میں حضور نبی کریم ﷺنے ہزار صحابہ اکرا… | 0 |
|  | 362 | ان بے شرموں غداروں اور بیغیرتوں کا منہ کالا کر کے گدھے پر بیٹھا کر پورے پاکستان کا چکر لگوانا چاہیے | 0 |
|  | 3484 | لعنت تم کنجروں اور حرامیوں پر جو تم جیسے گٹری کیڑوں کی وجہ سے پاکستان پر مشکلات آرہی ہیں | 1 |
|  | 3064 | اور آج دنیا بھر میں ان جیسا کوٸی زلیل نہیں سمجھا جاتا | 0 |
|  | 836 | یہ بہن چود نے بھی وڈیو بنائی ھے وہ بڑا حرامی سوور کی اولاد گشتی دا بچہ اے | 1 |
|  | 2078 | چپ مادرچود بالکل چپ | 1 |
|  | 966 | بھائی ان کی سب دوکانوں کو سیل کرو ۔ ان کی بدماشی نکالو، یہ لوگ ایک روپے کا بھی ٹیکس نہیں دیتے اور روزانہ لاکھوں روپ… | 0 |
|  | 2991 | پاکستان کے دانت کھٹّے کرنے والے ونگ کمانڈر ابھینند کو ملے گا ’ویر چکر‘ | 0 |
|  | 1363 | اس کتے حرامزادے، دلے، دلال، بغیرت، لعنتی، کھوتے کی طبیعت سب لوگ صاف کریں تا کہ اس دو ٹکے کے جاہل کو اپنی اوقات کا پتہ چل جائے | 0 |
|  | 2787 | کچھ دن حوالات اور جیل میں گزارے گی تو طبیعت صاف ہو جاے گی | 0 |
|  | 3033 | ایسی دھمکی آمیز وال چاکنگ پشاور پختونخوا اور پورے ملک میں جگہ جگہ دیکھنے کو ملتی ہے | 0 |
|  | 3366 | داد بھی تھا اس کا حرامی اور ماں تھی رنگ رَسیا | 1 |
|  | 2072 | ان ڈاکٹروں کی گانڈ ماری جانی چاہیے جو کہ رہے تھے کہ فریال تالپر بہت بیمار ھے | 1 |
|  | 2660 | یہ بارش کا پہلا قطرہ ہے ؛ اس کھوتو خان اور اسکے ابا کو جب تک جوتے مار کر نہی نکالیں گے تب تک یہ جائیں گے | 1 |
|  | 976 | ارشاد بھٹی تو ایک خبیث اور کمینہ انسان ہے | 1 |
|  | 2983 | سیلیکٹڈ اعظم کے کتو ماں کے انتقال پر ہی توبہ کر لیتے غداروں کنجروں کا ساتھ دینے سے | 1 |
|  | 309 | اوئے لونڈے کتے اپنی بکواس بند کرو حرامی | 0 |
|  | 1816 | آپکی گردن کی قریب ترین ہتھیار آپ کی زبان ہے،الفاظ کے چناؤ کا خیال کیا کرو | 0 |
|  | 2523 | گشتی کے بچے، دروازے کی چابیاں تیرے باپ باجوہ کے پاس ہیں۔ مادرچود اس کا نام لے جس نے تیری ماں چودی ہے۔ بھڑوا | 1 |
|  | 3321 | رگ ہی نہیں ان کا نفس بھی کاٹ دیا جاتا ہے تاکہ تاحیات ہیجڑے کی فخریہ زندگی گزار سکیں | 0 |
|  | 111 | مودی کو کتا کہنا کتے کی توہین ہے پتہ نہی یہ زلیل کس نسل کا جانور ہے | 0 |
|  | 995 | تتہ پانی سیکٹر پر بھارتی بلااشتعال فاہرنگ | 0 |
|  | 2185 | بھارتیوں کا پروپیگنڈہ ہے کہ پاکستانیوں کے ہر اکاؤنٹ کےپیچھےآئی ایس پی آر کا ہاتھ ہے | 0 |
|  | 1688 | بریکنگ نیوز پاک فوج نے بھارتی فوج کے افسر سمیت فوجی جہنم واصل کردیے، بنکر بھی تباہ کر دیے | 0 |
|  | 1495 | بی این پی کے مرکزی رھنما نواب امان اللّه زھری سال پوتے سمیت شہید کر دے گۓ | 0 |
|  | 2596 | اک بات بتاؤ اگر کویی تمہاری گانڈ مار دے تو تم اسکو معاف کر دو گے اور بولو گے | 1 |
|  | 61 | میں مج آرمی چیف کی مدت ملازمت میں توسیع کا خیر مقدم۔کرتا ھوں ۔ اور اس غیرت شخص پر بھیجتا ھوں جو… | 0 |
|  | 3203 | مسلسل بارش جہاں آبادی پر اثر انداز ہورہی ہے،وہیں قبرستان جہاں ہمارے پیارے مدفون ہیں،عبرت کا نشان بنے ہ… | 0 |
|  | 1027 | میری بیوی تیری ماں ہے نا گشتی ہی ہوٸ نا بہن چود اسی لیۓ تو کہتا ہوں تو گشتی کا بچہ ہے | 1 |
|  | 393 | پشتون ایک غیرت مند قوم ھے پنجابیوں کی طرح دوغلا پالیسی نہیں کرتا ضرورت کے وقت کھوتے کو باپ نہیں بناتے… | 1 |
|  | 3468 | مودی کے اقدامات پر بھارتی ڈاکٹروں کے دل خون کے آنسو رونے لگے | 0 |
|  | 2116 | بین چود، امارا بس چلے تمہارا اتنا گانڈ مارے تم کو صحیح غلط پتہ چل جائے | 1 |
|  | 3081 | کشمیر کے معاملے میں حکومت کی خاموشی بہت افسوس ناک ہے | 0 |
|  | 3273 | گشتی اور رنڈی کے بچے نواز شریف کی اصلی اولاد لندن میں بیٹھی مزے کر رہی ہے اوت تم جیسی رنڈی کے بچے | 1 |
|  | 3335 | حیرانی ہوتی ہے حامد میر جیسے بےغیرت صحافیوں پر بکواس کرتے ہیں کہ میڈیا کو باندھا جا رہا ہے | 1 |
|  | 3253 | حیرانی ہے کہ حامد میر غدارِ وطن کے گلے میں پاکستانی خفیہ ایجنسیاں ابھی تک پٹا کیوں نہی ڈال رہی | 1 |
|  | 1281 | ہم اپنی افواج کے قدم سے قدم ملا کر دشمن کو عبرت کا نشان بناہیں گے افواج پاکستان کے لیے دعاہیں کریں اسلام کا قلعہ پا… | 0 |
|  | 223 | ایک انڈین میجر کی ذہنی حالت سے اندازہ لگائیں کے وہ کس قدر حواس کھو بیٹھا ہے صرف ٹویٹر کی فوج سے یہ اتنا خوفزدہ ہے ت… | 0 |
|  | 2841 | ایسی گندی نسل صرف پاکستان میں ہی پائی جاتی ھے | 0 |
|  | 150 | پچاس سال بعد سلامتی کو نسل کا اجلاس اسکا کریڈٹ صرف عمران خان کو جاتا ہے بس اللہ کرے کشمیریوں کو آذادی ملے | 0 |
|  | 2609 | گشتی کے بچے | 1 |
|  | 1682 | رج جن لوگوں کو کاٹ رہا تھا ان سب نے تبدیلی کو ووٹ دیا اور آج اپنے نقصان کا ملبہ غیر اہم چیزوں پر ڈالتے دکھائی دیتے ہیں ماننا نہیں بس | 0 |
|  | 322 | آپ جیسے حرامی انسان سے تو بہتر ہیں ویسے تعلق کیا ہے شاہ صاحب اور پاکستان سے خبیث انسان | 1 |
|  | 122 | وہ کون کتے کا بچہ تھا جو کہتا تھا پورے ملک میں آگ لگادو | 1 |
|  | 1088 | تیری ماں کے یار مودی کی بھی گانڈ مارے گے اور تجھ جیسے کتے جو پاکستان پر بھوکتے ہیں ان کی بہن بھی چودے گے | 1 |
|  | 2152 | روز کی وہی مصیبت، نیند نہیں آتی حرام خور | 0 |
|  | 3354 | پاک فوج نے ایل او سی پر فائرنگ کی ، کوئی جانی نقصان نہیں ہوا ، بھارت کا الزام | 0 |
|  | 3290 | غریبوں کے علاقوں میں جاو کسی گشتی ماں کے حرامی بیٹے | 1 |
|  | 1549 | کچھ بتانے کو ہے ہی نہیں تقریر تیار کرنے والے بھی پریشان | 0 |
|  | 2225 | اپنی باجی مریم کی تصویر ایک دن بعد ہی اتار دی ہے تونے بس اتنی محبت تھی اپنی باجی بھگوڑی سے | 1 |
|  | 1776 | تیری گانڈ میں بنا تھوک کے کب دوں گشتی رن | 1 |
|  | 3480 | اسی فوج کی وجہ سے تم جیسے غدار بکواس کرنے کے قابل ہیں، ورنہ انڈیا کب کے تمھارے پرخچے اڑا چکا ہوتا | 0 |
|  | 619 | عمران خان نے انڈیا پر چڑھائی کردی کشمیر فتح کرلیا ہن مودی دی نیند حرام ہونا تے بندی اے | 0 |
|  | 2467 | او حرام زادے حرام کے پیسے پے پلنے والے کتے اپنی اوقات میں رہ سالے گٹر کی پیداوار تیری اوقات کیا ہے | 1 |
|  | 2621 | جاو مادرچود یہ ملک کا بیڑا غرق کردیا تم لوگوں نے | 1 |
|  | 1486 | او کسی گٹر کی پیداوار گشتی امریکہ میں تیرا یار کشمیر کا سودا کر کے آیا ہے | 1 |
|  | 1447 | عوام کے سامنے آگیا جس دن اسکی طبیعت صاف ہو جاے گی | 0 |
|  | 3014 | اس کتے کے پلے کو کیوں گورنمنٹ نے اتنی ڈھیل دے رکھی ہے جب ،جس پہ جی چاہے بھونکتا رہتا ہے حرامزاده | 1 |
|  | 632 | تو جو جس کا حادثاتی لونڈا ہے سب کو پتہ اگر اس کے خلاف بولو تو تیری تشریف میں آگ لگ جاتی ہے | 0 |
|  | 1911 | سر جی میں نے آپ کو بولا کی… | 0 |
|  | 1457 | انتہائی افسوس ناک واقع ہے پر آپ جیسے لفافہ صحافی ھر بات میں بغض بھٹو نکالنے کا کوئی موقع جانے نہیں دیتے | 0 |
|  | 3232 | ایک یہ مادرچود کا بچہ کھلا چھوڑا ہوا ہے کتے کی طرح بھونکتا رہتا ہے۔ | 1 |
|  | 1974 | مودی سب سے بڑا دہشت گرد ھے حرامی ھے کتا ھے بیغیرت ھے | 1 |
|  | 1091 | پچھلے ایک سال میں ہینڈسم باندر نیازی نے اپوزیشن پر بکواس کرنے کے سوا کچھ نہ کیا پورے ایک سال میں صرف دس بل پیش ھوۓ… | 0 |
|  | 411 | گھٹیا قوم ہے ہندو | 1 |
|  | 3169 | دل باغ باغ ہو جاتا ہے ان کنجروں کی آہ و بکا سن کے۔ | 1 |
|  | 1344 | یہ وہ گشتی ماں کا ہے جس کی اپنی بسجی دن رات دھرنوں پر مجرا کرتی تھی۔ لخ لحنت تم پر | 1 |
|  | 3094 | یہ ہے گشتی کی اولاد ہیرا منڈی کی پیداوار فک یو | 1 |
|  | 2611 | کتی کے بچے وطن فروشوں کے ٹاوٹ کیسی آزادی کیسا جشن کشمیر بیچ دیا حرام زادے تمہارے باپ باجوہ نے ایک نشئی کو فرنٹ مین بنا کر | 1 |
|  | 2933 | فرق جان کر جیو | 0 |
|  | 3080 | یہ سچ میں بہت مادرچود | 1 |
|  | 2282 | مودی نے جو ایکشن لینا تھا لے لیا اور کشمیر کو انڈیا کا حصہ بنا لیا جو پاکستان نکا اٹوٹ انگ تھا، شہ رگ تھا چہ رگ تک… | 0 |
|  | 1599 | اس چاٹ سے لگتا ہے نمبر ون تو کے پی کے ہے۔ | 0 |
|  | 3334 | وندر ڈی جی خان کمپنی کہ سامنے ہنڈا اور پراڈو کے دمیان خوفناک تصدم | 0 |
|  | 2253 | یدی کے اپنی کالی بوتھی تو ڈی پی پے دکھا نہیں سکتا اور بھوکے پاکستانی ہیں | 1 |
|  | 1547 | خالی برتن ہمیشہ آواز کرتے ہیں ۔۔۔۔ | 0 |
|  | 3365 | ویسے زرداری دور میں تو تم بھی ادھر سے ہی بھونکتے تھے، آج ادھر سے بھونک رہے ہو | 1 |
|  | 1192 | دلوں میں وہ بھی رہتے ہیں جو دنیا چھوڑ جاتے ہیں | 0 |
|  | 81 | چل بہن چود کنجری او رنڈی کی اولاد اب تک چوروں ڈاکوں کو سپورٹ کررہے ییں | 1 |
|  | 821 | ڑنڈی کے بچے اوقات میں رہ آواز نیچے کر کے بات کر مریم کے کتے بھڑوے پولیس نے ایک ڈنڈا دکھایا بھاگ گۓ | 0 |
|  | 3254 | تم انتہائ گھٹیا انسان ھونے کے ساتھ ساتھ جھوٹے بھی ھو | 1 |
|  | 2105 | کشمیر میں مسلمانوں کی مساجد بند کروا دینا اور عید نماز نہ ادا کرنے دینا ایک افسوس ناک حرکت ہے جس کی مزمت کرتا ہوں | 0 |
|  | 113 | کتنوں کے گندے خون کا نتیجہ ہے تو گشتی کتی حرامزادی لعنت تجھ پر | 1 |
|  | 752 | یہ کتیا نسل کی سور کی بچی جان بوجھ کر فوجی کو مشتعل کرنا چاہ رہی تھی کہ کچھ ہلا گلا ہو تاکہ اسکو مزید حرامی پن مچان | 1 |
|  | 3176 | بیغیرت کنجروں کا اجتماع کبھی بھی ایسے کنجروں کو جواب نہیں دینا چاہئے ۔ بیٹیوں کی شادی پر ناچ گانا کنجروں کا ہی رواج ہے | 1 |
|  | 66 | بکواس بند کر بھڑوے سالے۔۔۔عمران خان نواز شریف نہیں جو ذاتی مفادات کی خاطر قوم سے جھوٹ بولے گا | 1 |
|  | 1529 | مہنگائ ہو تو وزیر اعظم چور ہوتا ہے۔ ڈالر ۔ پشاور کھڈا میٹرو۔ جنگ میں بھی آرمی چیف کی توسیع نہیں ہوئ لیکن ابو تین… | 0 |
|  | 2778 | غرورِ امیراں میں جینے والو۔ ڈرو اس وقت سے جب آپ کو اللہ کی لاٹھی آ پکڑے گی اور آپ عبرت کا نشان بنا دیئے جاؤ گے | 0 |
|  | 142 | اک بات بتاؤ اگر کویی تمہاری گانڈ مار دے تو تم اسکو معاف کر دو گے اور بولو گے | 0 |
|  | 1948 | سنا ہے پی ٹی ائی میں شامل ہوتے ہی بندے کی غیرت والی رگ کاٹ دی جاتی ہے | 0 |
|  | 470 | انڈیا میں کوئی ایسی سیاسی جماعت یا TV چینل نہیں جو اپنی فوج یا RAW کےخلاف بکواس کرے | 0 |
|  | 569 | ہٹ گشتی دے بچے لن تیری ماں نو پہن دی کُس یاوا دلہ کاکو نو لون ای اپنی بکواس کول رکھ تو ایڈا باجی یاوا دانشور | 0 |
|  | 1957 | تم لوگ جتنی مرضی بکواس کر لو پاک فوج ہماری جان ہے ہماری شان ہے ہمارا مان ہے تم لوگ دو ٹکے کے لوس اور کر بھی کیا سکتے ہو | 0 |
|  | 3066 | اس ہجڑے کتے کو پٹہ ڈالنے کا وقت آیا چاھتا ہے | 1 |
|  | 1389 | یا اللہ کشمیری عوام پر ہندو ظلم و بربریت کی انتہا اور ہماری سپاہ کہیں سوئ پڑی ہیں؟ | 0 |
|  | 1515 | کھسرے ملک خانہ جنگی کی حالت میں ہے کشمیر میں روزانہ کتنے لوگ شہید ہو رہے ہیں | 1 |
|  | 1328 | اس نے بہت گندی بکواس کی یہ بھی کم لگ رہا مجھے | 0 |
|  | 230 | یہ بتاتے ہوئے تمہیں شرم سے مر جانا چاہیے بے غیرت بہن کو گلی گلی میں بھیج رہے ہو باپ جیل میں مر رہا ہے | 1 |
|  | 2603 | پاکستانیو ان کنجروں کی چیخیں انجوائے کرو۔ ہماری طرف سے اُن کنجروں کی ماں بہن ایک کریں | 1 |
|  | 1253 | زرداری صاحب ان کی گانڈ مارے گا تھوڑا صبر کر جاؤ | 0 |
|  | 307 | بے شرم انسان کشمیر کے لوگ بھی مر رہے ہے منہ کھولنا ہےتو ادھر کھول بہن چود بھڑوا گانڈو | 1 |
|  | 1916 | اوئے گانڈو کے لوڑے | 1 |
|  | 3112 | جو کہتے تھے کے علامہ خادم حسسیں رضوی دین پر سیاست کرتا ہے انکے منہ پر زبردست طمانچہ | 0 |
|  | 725 | کسے کتی دیا پترا تیری سزا یہی ہے تیری نسل تیرے ساتھ ہی ختم ہوجائے گی وہ دس دن سے کرفیو میں ہیں اور تو جشن منا رہا ہے | 1 |
|  | 1004 | جھوٹ بول رہی ہے یہ گشتی لعنت ایسی گشتی پر | 1 |
|  | 239 | عزت کا ایک سال ذلت کے سال سے بہتر ہے عزت کا ایک سال | 0 |
|  | 3031 | چوتیے اپنی شکل دیکھ اور اپنی بکواس، تُو جتنا بھی بھونک لے میجر غفور کے جوتے برابر بھی پہنچ نہیں سکتا | 0 |
|  | 1303 | بھائی تم نے تو اسے میڈل دے دیا آنر کا یہی تو وہ چاھتا ھے کے کنجروں کی محفل میں کنجر ھی لگے ️ | 1 |
|  | 1648 | پرویز رشید صاحب کیا شاعرانہ کتے خانی کیتی جے | 1 |
|  | 490 | جب اس وقت کا صدر آرمی کی گاڑیوں کے بنائے جانے والے پرزوں کا معائنہ نواز شریف کی موجودگی میں ک… | 0 |
|  | 2873 | پاک فوج کا ایل او سی پر ہندوستانی فوج کو منہ توڑ جواب ایک افسر سمیتہندوستانی فوجی مردار چوکیاں بنکر تباہ DGISPR ویلڈن پاک آرمی | 0 |
|  | 3136 | بھارت کی فائرنگ پاکستانی شہید | 0 |
|  | 3021 | آپکا کام صرف بک بک کرنا ھے | 1 |
|  | 2164 | ابھی ان بیغیرتوں کے وٹ یعنی بل سیدھے کیے جا رہے ہیں فائنل پھانسی کی سزا تو انشاءاللہ ماڈل ٹاؤن کیس میں ہی ہو گی | 0 |
|  | 1182 | یہ کنجری عورتیں ہیں ان بےغیرتوں کو ننگا کر کے چوک پر کھڑا کر دو یہی ان بےغیرتوں کی پہچان ہے | 0 |
|  | 1450 | یہ بہن چود سیاسی قیدی نہیں ہیں یہ حرامخور نسل کے حرامی غدار ہیں ان کی تو لاشیں بھی نہیں ملنی زندہ تو بہت دور کی بات ہے | 1 |
|  | 1857 | اب پھدکنا بند کر گشتی کے بچے ورنہ کتے کی موت مرے گا | 1 |
|  | 2063 | بےغیرتوں کا تخم تو خود ہے بلاول ذوالفقار علی بھٹو کے نام پر کلنک | 1 |
|  | 50 | چند کتے سرخے | 1 |
|  | 2640 | کھوتی کے جاہل بچو کمپین آج سے شروع نہیں کی، | 1 |
|  | 1022 | تجھ سے بڑا خبیث اور گانڈو نہیں دیکھا دلے انسان جھوٹے فریبی لعنتی انسان | 1 |
|  | 92 | پوری قوم خوش ہے جنرل باجوہ کے مزید تین سال رہنے سے یہ اللہ کا کرم ہے | 0 |
|  | 2386 | ایک ڈاکو دفعہ وزیر اعظم بنا اور لوگ کچھ چوتھی دفعہ کے لیے بھی رنڈی رونا کر رہے تو شرمندگی نہیں | 1 |
|  | 2482 | تیری ماں کی چوت کنجری کا بچہ بہن چود سور کی اولاد بیغیرت دلال ۔۔۔۔۔لفافہ حرامی | 1 |
|  | 3244 | تیری گانڈ جس جس نے ماری ہے اس کو تمغا ملے گا شکل سے ہی گانڈو لگتا ہے بغیرت | 1 |
|  | 866 | ناصر عباس شھید کہا کرتے تھے کہ ہم کیا کریں جس دن آل محمد ع کے ہاں خوشی ہوتی ہے اسی دن ان کے ہاں کوئی ہلاک ہو جاتا ہے۔ | 0 |
|  | 593 | واھ باپ کا مال سمجھا ہے ایک بے غیرت ڈاکو لٹیرے جرائم پیشہ کو دوسرون کےساتھ زبردستی سفر کرنا | 1 |
|  | 2168 | بےشرم کنجر لوگوں جواب تو ہمیں بھی نہیں دیا کسی کھسری کے بچے تو ہمارا جواب دے | 1 |
|  | 1591 | ابھے گانڈو یہی پرابلم ہے میرے ملک میں جسکے پاس چار پیسے آ گئے حرام یا حلال کے وہ خود کو قانونسے بالا سمجھتا ہے | 1 |
|  | 658 | بکواس مت کرو | 0 |
|  | 729 | سچ تو یہ ہے کہ ہم بولتے ہیں اور بہت بولتے ہیں اور اس کا زیادہ حصہ ایسا ہوتا ہے جسے کسی ہچکچاہٹ کے بغیر بکواس کہا جائے | 0 |
|  | 1205 | کتے کے بچے، حرامی سور تو مخبری کرنے بیٹھا ہوا ہے یہاں غدار نسل کے کتے | 1 |
|  | 522 | افواج پاکستان جزبہ ایمان جزبہ شہادت اور قوم کی دعاوں کے ساہے میں ہیں کسی کو پریشان ہونے کی ضرورت نہیں ہماری افواج ہ… | 0 |
|  | 2140 | اس کتی عورت کے ساتھ جو ہوا اچھا نہیں ہوا بہت کم ہوا جو اپنے ملک کی فوج کے بارے میں بکواس کرتی ہے | 1 |
|  | 2807 | غدار انسان امریکہ رہ کر تجھے کیا لگتا ہے تو محفوظ ہے مردہ ضمیر انسان اللہنکی پکڑر سے تو کہی بھی نہیں نچ سکتا عبرت ک… | 0 |
|  | 3449 | تو کھوتی کے بچو، ہٹاو نا انکو ، کدھر ہیں تمہارے ھڈ حرام عملہ جس کا کام ہی تاروں کی سیٹنگ… | 1 |
|  | 382 | پونچھ سیکٹرز میں کئی اہم بھارتی چیک پوسٹیں تباہ | 0 |
|  | 1808 | مادرچود تیری بھی فیملی ہے؟ | 1 |
|  | 2642 | بہن چودون تمہاری اولادیں بھی اللہ کرے ایسے ہی کرنٹ لگنے سے مریں اور ساتھ میں تم بھی کتے کی موت مرو مادر چود انسان | 1 |
|  | 3486 | پتہ ہے تو ٹویٹ پر بکواس لکھنے سے پہلے خود کچھ پڑھ لینا چاہیے | 0 |
|  | 1036 | چوری کرو تو اربوں کی نہیں تو عبرت کا نشان بنا دئیے جاو گے | 0 |
|  | 1449 | اگر بیغیرتوں کے سر پے سینگھ ھوتے تو آج بلاول بارہ سنگھا ھوتا۔ کھسرا کھسرا ہوتا ہے | 1 |
|  | 1250 | خان تیری گانڈ بھی مارے گا اور تجھے پتہ بھی نہی چلے گا گھٹیا عورت | 1 |
|  | 199 | یہ وہ مادرچود ہیں جن کی پارلمنٹ کی بلڈنگ بھی پاکستان نے بھیک میں بنا کے دی تھی | 1 |
|  | 1190 | اسی کتے تے پٹواری اک برابر | 1 |
|  | 1259 | گھٹیا انسان بکواس کر کے لوگوں کو کیوں گمراہ کرتے ھو | 0 |
|  | 521 | پاکستان آرمی کا تتہ پانی سیکٹر پر انڈین آرمی پر کامیاب حملہ انڈین آرمی کے فوجی جہنم واصل اور بنکر تباہ | 0 |
|  | 2610 | فوجی اگر کام کرتا ہے تو… | 0 |
|  | 2012 | دل جیتنا ہمدردی اور درد محسوس کرنا اگر نہیں آتا تو سیکھو نیوزی لینڈ کی وزیر اعظم سے | 0 |
|  | 2698 | سپورٹ چنارفری ڈائیلائسزسنٹربھمبر | 0 |
|  | 2117 | مادر چود پورا بیان سن لو میر زلیل کی چکلے نے آدھا بیان نشر کیا لعنت تمہارے شکل پر کتے سب سے بڑا غدار تم لوگ ہو | 1 |
|  | 2382 | ابھے چپ بہن چود | 1 |
|  | 2887 | عمران فاروق کیس کے بے گناہ ملزم معظم علی کا زندہ درگور گھرانہ کیسے جشن آزادی منائے؟ | 0 |
|  | 922 | کون تیری ماں کا ٹھوکو تھا جس نے کشمیری خریت قیادت سے ملنے سے انکار کیا ؟؟ | 0 |
|  | 2584 | پٹواریو کتے کی نسلو | 1 |
|  | 2733 | اور تم بھی پوچھ لو۔ | 0 |
|  | 2886 | اس بہن چود نظام کو لپیٹ دینا چاہیے | 1 |
|  | 2280 | فوجی اور سویلین قیادت مکی مسائل حل کرنے میں مکمل ناکام۔ | 0 |
|  | 421 | مدثر میں تمہارا خون پی جاوں گی اگر میرے اکاونٹ کو کچھ ہوا | 0 |
|  | 1631 | بلاول بیغیرت زرداری چور کس کو بیوقوف بنا رہے ہیں آج پیپل پالٹی ن لیگ کے بناۓ کیسز میں اور ن پیپل پالٹی دور کے بناۓ گۓ تھے | 1 |
|  | 433 | ابھی گنجن کی اولاد تیرا بھی وقت آجائے گا بڑے بہت خوب بکواسیات کر رہا ہے مادرچود | 1 |
|  | 1369 | گولی ماریں گے سالے کو ۔۔۔ | 1 |
|  | 1748 | آج کوئی اس کتے کے بچے کو بتاو… | 1 |
|  | 1715 | اس حرامی کو بھی جیل میں بند کر کے اس کی گانڈ ماری جائے اور ویڈیو ڈارک ویب پر بیلن روپوں کی بک جانے سے | 1 |
|  | 1694 | کتے حرامی بہن چود دھشت گرد ایک طرف ھمارے کشمیری بھاٸیوں اور بہنوں کو شہید کرواتے ھو | 1 |
|  | 2812 | کس منحوس درندے کا نام لے دیا ۔ جو قوم کو دن رات لوٹ لوٹ کر کنگال کر گیا ۔ | 0 |
|  | 1362 | ایک بڑا کنجر دوسرا چھوٹا کنجر دونوں سالے گٹر کی پیداوار ہیں جتنا نقصان ان حرامیوں سے پاکستان کو پہنچا ہے اتنا کسی | 1 |
|  | 2328 | لعنت ہٸے بیغیرت کنجروں کے گھر میں عورتیں نہی ہوں گی | 1 |
|  | 1143 | مادرچود دلے اس اپنے باپ کو بھی سن کیا کہتا ہے | 1 |
|  | 622 | اس کو کون دفنائے گا اس کو ممی بنا کے چوک پر رکھا جائے گا عبرت کے نشان کے طور پر | 0 |
|  | 1821 | ذلت و رسوائی کی کوئی انتہا نہیں ہوتی ہے جب کبھی شرو ع ہوجائے تو نیست و نعبود کر کے ہی دم لیتی ہے | 0 |
|  | 3027 | امریکی اخبار میں شائع ہونے والا ذلّت آمیز کارٹون۔ | 0 |
|  | 2624 | تم مادرچود ہو | 1 |
|  | 2170 | نئے پاکستان کے آفیشل چینل سے لانچ کر دیا گیا شکریہ کتے کے بچے نیازی شکریہ | 1 |
|  | 9 | کراچی میں پانچ سے دس لوگ بارشوں کی وجہ سے جاں بحق ہوئے اور میڈیا نے آسمان سر پہ اٹھا لیا | 0 |
|  | 416 | آج مودی کی نفرت کے زہر میں پلے کُتے ایک نہتی… | 0 |
|  | 1322 | وہ صرف کتے کے بچے تھے۔ | 1 |
|  | 349 | ذلیل آدمی اپنی بکواس بند کر | 0 |
|  | 2357 | مریم نواز کو میاں نواز شریف کے سامنے گرفتار کیوں کیا؟ | 1 |
|  | 2446 | کچے گھروں سے پکے مکانات اور اوپر سے نیچے تک ملک میں سوروں کا راج ہے | 1 |
|  | 634 | ابے بھڑوے بنی گالا کے کتے جب بات کروگے زیر ہی اگلوگے | 1 |
|  | 2739 | خان نے سہی کہا تھا کہ میں ان کو رولاؤ گا ان کو تکیف پہنچے گی اوے کنجروں یہ تو پارٹی شروع ہوئی ہے | 1 |
|  | 747 | پاک فوج نے بھارتی فوج کے افسر سمیت نجس جانور جہنم واصل کردیے،،، بنکر بھی تباہ کر دیے۔ | 0 |
|  | 1316 | ناران میں لینڈ سلائیڈنگ کیوجہ سے گاڑی گہری کھائی میں جاگری خار سے تعلق رکھنے والے چار افراد جاںبحق… | 0 |
|  | 574 | تبدیلی کی برسی نہیں چوروں کی برسی جو ملک کو بھی کھا گۓ لوٹ کر اور اس کی فوج کے خلاف بکواس کرتے ہیں سستے لونڈے تے لونڈیاں | 0 |
|  | 753 | حرام خوروں سے ایسے دلال بہتر ہیں۔ دن رات ایک کر کے نمک حلالی کی کوشش تو کر رہے ہیں | 1 |
|  | 2347 | آپ کی حکومت نے کشمیر کا سودا کیا اب دعا سے کشمیر آزاد کرلیتے ھو انڈیا حکومت کو سخت پیغام دو۔ | 0 |
|  | 2524 | تم یار بہت بڑے بے غیرت ھو ۔اپنی والدہ کا ھی خیال کرو | 1 |
|  | 1564 | تو تو ہندو کے ٹکڑوں پر پلنے والا کتا ہیں | 1 |
|  | 723 | ویے اپ اپنے اختیارات کو استعمال کرنے ھوےء سوموٹو لے لیتے ہیں | 0 |
|  | 1237 | رنڈی کے بچے یہ تیرا اور تیرے لندن والوں کا کام ہے تیری گانڈ ماری ہے کیا مصط | 1 |
|  | 3238 | دو مادرچود لاہور کے جنہوں نے رابعہ خان نامی لڑکی کی عزت لوٹی | 1 |
|  | 287 | تجھ جیسے غداروں کو بے نقاب کردیا جائے گا بہت جلد اور تمہارے آقاؤں انڈیا ازرائیل اور امریکہ کو عبرت کا نشان بنادیا ج… | 0 |
|  | 2411 | باپ دادا افغانستان کا، ماں جموں کی، بیٹا پاکستان کا نمک حرام، جس نے پاکستان کا نمک کھا کے پاکستان سے وفا نہ کی | 0 |
|  | 1743 | اس وقت تین سب سے بڑے مسائل ہیں جن کے لیے فوری اقدامات اٹھانے کی ضرورت ہے | 0 |
|  | 3071 | آخرکار کوئی تو سچ بولا | 0 |
|  | 3230 | زندگی کا سب سے بڑا نقصان موت نہیں ہے | 0 |
|  | 1807 | جس بندے کی گانڈ اُس کا اپنا ماموں مارے اس کی ذہنی حالت ایسی ہی ہو گی | 0 |
|  | 2474 | یا اللہ جو جانتے بوجھتے کہ یہ نااہلوں کا ٹولا ہے | 0 |
|  | 1557 | قوم یوتھ کا بس نہیں چل رہا کہ لکھیں کہ توسیع کے فیصلے کے بعد، اسرائیل، بھارت، امریکہ، افغانستان کے آرمی چیفس کی خوف… | 0 |
|  | 1537 | ریحان کی ٹی شرٹ پر ایسا فقرہ لکھا تھا جو اس ملک کے حکمرانوں،اشرافیہ،بیورو کریٹ سے لے کر ہر اس شخص کے منہ پر طمانچہ… | 0 |
|  | 1585 | چپ گشتی کے بچے ہم بھولے نہیں جب نواز UN میں برہان وانی کا کیس لڑو رہا تھا تو تیرے جیسے مادرچود ذات کے | 1 |
|  | 1629 | کیونکہ اب فواد حرامی جیسے کنجر ہمارے حکمران ہیں جو کشمیر کو بیچ آئے ہیں۔ ہمیں تم جیسے بے غیرت نہیں چا ہیے | 1 |
|  | 1823 | سن لے بے غیرت مودی | 1 |
|  | 3413 | چوتیے اپنے مالکوں کی جیل سے چیخیں سن کر بھی تجھے عقل نہیں آئی | 0 |
|  | 330 | مریم صفہدر کو بھول گیا نمک حرام | 1 |
|  | 1460 | لیکن صرف گرفتاری کافی نہیں ب… | 0 |
|  | 104 | یہ بہت بکواس کرنے لگ گئے ہیں اپنی باری ان کو لال مرچیں لڑتی ہیں گائے کا پیشاپ پینے والی گندی قوم | 0 |
|  | 964 | یہ بیغیرتوں کے ٹولے کا ادنا سا پیادہ ہے یہ وہی کچھ بک رہا ہے جو اسکو ڈکٹیٹ کیا گیا ہے | 0 |
|  | 503 | بےغیرتوں بے شرموں۔ کراچی ڈوب رہا ہے۔ تم نے کیا کر لیا ؟ | 0 |
|  | 145 | لعنتیوں کنجروں بھیڑیوں نسلی کتوں کے درمیان شیر لیٹا ہے | 1 |
|  | 1206 | بہن چود کے پترو چوتیا سمجھا عوام کو۔ لاہور مینے لگے سرکاری پوسٹروں سے کشمیر نکال کر اب پئن یوا رہے ھو | 1 |
|  | 1582 | کسی کے ساتھ غلط کر کے اپنی باری کا انتظار ضرور کرنا کیونکہ قسمت جب طمانچہ مارتیہے تو وہ منہ پر نہیں روح پر لگتا ہے۔ | 0 |
|  | 1852 | کسی کنجر دی اولاد نے | 1 |
|  | 1651 | کچھ لڑکیوں میں تو ماشاءاللہ اتنا ایٹیٹیوڈ ھوتا ھے کہ اگر انہیں مچھر بھی کاٹ لے تو مچھر کو پکڑ کہ انگلش میں پوجھتی ہیں | 0 |
|  | 251 | نریندر مودی تمہیں یہ عید کا پیغام دیتے ہوئے ڈوب مرنا چاہئیے،گجرات میں دو ہزار مسلمانوں کا | 0 |
|  | 70 | بھارت کے سارے اوچھے ہتھکنڈوں کے بعد جب کچھ بھی ہاتھ نہیں آرہا تو پانی چھوڑ کر ہماری فوج کو ادھر مصروف کرنے کے پلان… | 0 |
|  | 2774 | سنا ہے بچپن میں تو اپنی بہن چلاتا تھا | 1 |
|  | 2438 | کتے کی موت تم لوگ مرو گے | 0 |
|  | 1434 | ایک طرف دھرتی کے بہادر سپوت نوجوان نے جان کا نذرانہ دے کر اپنے جہاز کو وطن دشمنوں کے پاس نہیں جانے… | 0 |
|  | 666 | ھم پنجابی معذرت کے ساتھ اپنا رویہ تبدیل کرنا ھوگا سارے وڈیرے زندہ درگور کر کے ایک پنجابی قوم بننا ھوگا | 0 |
|  | 741 | جو بہن چود حمید گل یوتھیے کنجر کا حامی ہے سامنے آۓ تاکہ اس کو بھی بلاک کر کے گند صاف کر دوں | 1 |
|  | 441 | سن اوے مودی انڈیا کے کنجر دلال کتی چور اب پاکستانی قوم میں پہلے سے زیادہ جزبہ ہے | 1 |
|  | 1277 | اگر مدرسے کے بچوں کو منہ کھول کر دھشت گرد کہا جاسکتا ہے، تو بلاشبہ کالجوں میں ڈانس کرنے والوں کو کنجر کہا جاسکتا ہے | 1 |
|  | 2751 | تتہ پانی سیکٹر پر بھارتی فائرنگ سے شہری شہید جس میں ایک سال کا لڑکا بھی تھا ڈی جی آئی ایس پی آر کے مطابق پاکستا… | 0 |
|  | 2515 | ایکسٹنشن پہ دِلی اور دلے یکساں پریشان ہیں | 1 |
|  | 320 | ببلو گانڈ سے سر نکالو اور دیکھو دنیا میں کیا ہو رھا ہے۔ ورنہ اسی حالت میں مارے جاؤ گے۔ | 1 |
|  | 1938 | انسان کی سب سے بڑی نا کامی اسی وقت شروع ہو جاتی ہے جب وہ کسی انسان سے امیدیں جوڑتا ہے ۔ | 0 |
|  | 1270 | اچھا دھمکیاں مل رہی ہیں | 0 |
|  | 2806 | چوروں کے گھر چوری کرنے گیا تھا یہ بھکاو کتا مگر ہاتھ کچھ نہیں آیا بھگوڑا | 1 |
|  | 3184 | کتنا خبیث انسان ہے تو | 1 |
|  | 2103 | ہجڑے کنجر کٹپتلی ہے سب سالے | 1 |
|  | 1424 | ھجڑے کا منہ دیکھو | 1 |
|  | 958 | ہم مسلمان موت سے نہیں ڈرتے ہم اپنے حقوق اور اپنی انا کےلئے جان کی بازی لگاتے ہیں۔ انڈیا تیار رہو | 0 |
|  | 3447 | پیسے بنانے کی مشین بنا کے رکھا ہیے اس گھٹیا طبقے نے | 0 |
|  | 3351 | اس بندے میں غیرت ہوتی تو اپنی بے عزتی پہ کلیپنگ کرتا ؟ نسل در نسل بے غیرتوں کو چن چن کر وزیر بنایا گیا ہے | 0 |
|  | 1613 | کیا کوٸدوست تصدیق کر سکتا ہے کہ سلکٹیڈ اعظم نے اسے او زلفی بخاری کو سرکاری خرچے پر کشمیر ریلی کے لیے لندن نہیں بھیج… | 0 |
|  | 3385 | پاک فوج کا ایل او سی پر بھارت کو کرارا جواب، افسر سمیت فوجی ہلاک | 0 |
|  | 1504 | سعودی عرب نے انڈیامیں سرمایہ کاری کی بہت اچھاکیا | 0 |
|  | 3003 | افسوس ناک واخوشاب سرگودھا روڈ پر جھیل کے قریب دو موٹرسائیکل آپس میں ٹکرانے سے دو افراد جاں بحق، | 0 |
|  | 1463 | ٹیپو سلطان نے انگریزوں اور ہندو مراٹھوں کے خلاف بہادری سے لڑتے ہوئے ہرمیدان میں شکست سے دوچار کیا | 0 |
|  | 969 | او کھوتے خور پٹواری یہ جس کی تصویریں لگا کر تو پٹواریوں کو بیوقوف بنا رہی ہے میں اسے اچھی طرح جانتا ہوں | 0 |
|  | 2643 | لو جی کشمیر ہمارا گیا شہ رگ ہماری کٹی تکلیف ہمیں ہوئی اور ہمارے ہی کسی نمک حرام نے بلایا میکا سنگھ کو تقریب میں | 0 |
|  | 2685 | اپنی گند اٹھاؤ رنڈی کے بچے بہن چود بھڑوے | 1 |
|  | 1375 | کسی گشتی کے بچے کسے کتے کی آوارہ اولاد مادر چود کے بچے | 1 |
|  | 1600 | احباب گردےکےبغیرانسان زندہ نہیں رہ سکتااوریہ گردہ مشین ھےجو گردےکی جگہ کام کرتےھوئ | 0 |
|  | 153 | یہ عورت بہت گھٹیا قسم کی ہے اس نے ایک لڑکی پر بہت بری طرح تشدد کیا ہے | 0 |
|  | 3113 | حرام خور کا پیٹ دیکھو ایک دفع شراب پی کے بھی پکڑا گیا تھا اس کو تو سرے عام کوڑے اس وقت تک… | 1 |
|  | 809 | دگڑ دلی حرام خور نانی اماں ڈرامے باز بھگوڑی مریم صفدر | 1 |
|  | 986 | غدار کی اولاد | 1 |
|  | 2618 | اب کا لنگڑا گھوڑا کس کام کا۔ دشمن اناج کا۔ | 0 |
|  | 959 | یہ پٹواری کون ہے مجھے بتائیں میں اس کی طبیعت صاف کروں آپ دل چھوٹا نہ کریں پلیز | 0 |
|  | 1345 | ریاست کو چاہیے کہ اس کتے کو لگام ڈالیں یہ ہر روز ریاستی اداروں پر بکواس کرتا ہے اب یہ دن بہ دن حد کراس کر رہا ہے | 0 |
|  | 1262 | گُل بُخاری گشتی تھی گشتی ھے گشتی رھے گی کر لو جو کرنا ھے | 1 |
|  | 901 | حکمران اب بھی یہى بکواس کر رہے ہیں کہ اگر هم جنگ ہار گئے تو ؟ | 0 |
|  | 874 | چار دن کی چاندنی پھر اندھیری رات ہے۔۔ اس مثال کے مطابق تمام چور آج نشان عبرت بنے جیلوں میں پڑے ہیں۔ | 0 |
|  | 1965 | چور ہے سالی ڈاکو کی بہن | 1 |
|  | 2196 | دو ٹھگوں کی جوڑی اک ہیجڑا اک بھگوڑی | 1 |
|  | 7 | اوئے بہن چود۔۔۔۔۔ یہ کیا؟؟؟؟ | 1 |
|  | 501 | کھوتی کے بچو تمہارے دور میں سال پاکستان کا وزیر خارجہ ہی نہیں تھا اس سے بڑی نااہلی اور کیا ہو سکتی ہے | 0 |
|  | 682 | وہ خود ایک پرانے جوتے کی صورت میں زندہ درگور ہے | 0 |
|  | 755 | حیرت ہے محض سیاسی اختلافات کی بنا پر گالیاں بکے جا رہے ہیں | 0 |
|  | 2400 | غریبوں کا خون چوس کر تمام ترقیاتی کام بند کروا کر ظاہر ہے تجارتی خسارہ کم ہی ہو گا باقی پر بھی بولتے نا منافق انسان | 0 |
|  | 496 | فوج نے کونسا تیری گانڈ ماری ہے جو بھونکتا جا رہا ہے نکل تیری زات کا بھیدا ماروں | 1 |
|  | 422 | کیا گانڈ ماری ہے خان صاحب نے علی زیدی کی | 1 |
|  | 1178 | اپ پچھلے دس سالوں سے گھٹیا ترین زبان استعمال کر رہے ہیں شرم اتی ہے اب | 0 |
|  | 2975 | گشتی حرام کی کتیا عورت | 1 |
|  | 1499 | میرا گھر مکمل طور پر تباہ ہوا ہے کچھ گولے پاکستان آرمی کی طرف سے لگے تھے اور کچھ اسمانی بارش کی وجہ سے تباہ ہوا ہے… | 0 |
|  | 2956 | ہاں جی کُتی کے بچے کیا بنا تیری اس ماں کا تیری ماں پی ٹی آئی سے ہے | 0 |
|  | 3275 | مراد سعید تو حرامی ہے اور تیرا کتا باپ عمرو نیازی مادرچود ہے تم دونوں گانڈو واشل ہو | 1 |
|  | 1412 | مودی کتے مار جا تیری ماں نو | 1 |
|  | 1818 | نمک حرام تیری پوری نسل ہے جو آج ہمارے اجداد کے بنائے ہوئے ملک پر قابض ہوکر بیٹھے ہو سندھی انگریزوں کے غلام | 0 |
|  | 3379 | آج میرے سالہ بھتیجے نے مجھےبتایا ہماری اچھی فوج انڈیاکی گندی فوج کی پٹائی کرے گی اورانکوبھگادے گی۔ | 0 |
|  | 3336 | تمہیں پتا ہے ملک نے کیا ٹویٹ کی ہے علی زیدی کے بارے میںاب جب تم چاہتے ہو کہ اسکی طبیعت صاف کر دی جائے | 0 |
|  | 1061 | گشتی بیٹی کا غدار وطن کتا باپ لعنت ہے تم کتے کے بچے کتے | 1 |
|  | 2980 | بندہ لا لوے جتی تے مارے بکواس نہ کر کتے | 1 |
|  | 510 | تمہیں کیا کچھ نہیں کہا خبیث انسان تم تو مرے ھوے ضمیر کے ہو | 1 |
|  | 2545 | اہو اب بہت ساروں کی گانڈ میں چالیس ملک امت امت والا کیڑا سٹ مارے گا | 1 |
|  | 1762 | یہ ہے پٹواریوں کی زبان جو آرمی چیف کی ملازمت میں توسیع پر بکواس کریں گے ۔۔ | 0 |
|  | 1398 | کیپٹن سندھڑی اور میجر جنرل چونسا شریف | 0 |
|  | 3181 | تم بھی صحافی بننے کا خواب دیکھتے دیکھتے حرامی بن گۓ ہو | 1 |
|  | 897 | اصل بات یے هے که نون لیگ غازی ممتاز کو شهید کرایا اور غستاخوں ملک سے… | 0 |
|  | 2191 | اے دشمن دین تو نے کس قوم کو ہے للکارا | 0 |
|  | 32 | خادو لنگڑا سوراں یدا | 0 |
|  | 2996 | ﺍﺳﻼﻡ ﻭﻋﻠﯿﮑﻢ ﺍﮮ ﺍﻟﻠﮧ ﻣﺠﮭﮯﺍﺱ ﺩﻥ ﺭُﺳﻮﺍ ﻧﮧ ﮐﺮﻧﺎ ﺟﺲ ﺩﻥ ﻟﻮﮔﻮﮞ ﮐﻮﺩﻭﺑﺎﺭﮦ ﺯﻧﺪﮦ ﮐﯿﺎﺟﺎﺋﮯﮔﺎ | 0 |
|  | 1493 | یہ ہے ان بیغیرت لوگوں کا کنجر پنا نا یہ خود کام کرینگے نا ہی کسی کو کام کرنے دینگے | 1 |
|  | 1720 | آہ سچ ہے کے انسان تو زندہ مگر انسانیت مر چکی | 0 |
|  | 184 | ببلو گانڈ سے سر نکالو اور دیکھو دنیا میں کیا ہو رھا ہے۔ ورنہ اسی حالت میں مارے جاؤ گے۔ | 1 |
|  | 3073 | اور۔ آپ نے تو بہت کچھ کیا عوام کے لیے سالوں ایک اچھا ہسپتال تک تو تم لوگ سال میں بنا نہ سکے اور بکواس کرتے ہو ایک… | 0 |
|  | 1530 | ہم فردوس عاشق اعوان کی اور قریشی کی بکواس سن سن کر تنگ آچکے ہیں | 0 |
|  | 2629 | لگتا ہے یہ مادرچود سرپرائز بھول گئے اس کو دوبارہ یاد دلانا پڑے گا | 1 |
|  | 2506 | جب سے زاٸد کشمیری مسلمان لڑکیوں کو پلید بھارتی فوجیوں نے اٹھایا ہو گا۔ اسکے بعد کیا ہوا ہو گا۔ سوچنے کی ہمت نہی… | 0 |
|  | 598 | لعنت تیرے اور تیرے پاپا پر گندی لچی اولاد گٹر کی پیداوار بھگوڑی نواز | 1 |
|  | 191 | کھوتی کے بچو بس بھونکتے ہی رہتے ہو ہر وقت مولانا فضل الرحمٰن کی پالیسی پر تب تو انڈیا کی ہمت نہیں… | 0 |
|  | 2813 | مراد سعید تو حرامی ہے اور تیرا کتا باپ عمرو نیازی مادرچود ہے تم دونوں گانڈو واشل ہو انڈیا نے تم سب ک… | 1 |
|  | 435 | کنجروں کا دور ختم ھو چکا ھے اور یقیناً تیرا انجام انہیں پہنڑ دلالوں کیساتھ ھو گا | 0 |
|  | 1695 | بکواس کر رہی ہو گیراج والی کی پیداوار خان نے کہا اگر تم کہتے ہو میں حملہ کر دوں تو ٹھیک ہے | 1 |
|  | 1360 | بہن چود گانڈو نسل پہلے نواز شریف کی چاٹتا تھا پھر اس کی اولاد کی اور اب اس کی اولاد… | 1 |
|  | 1014 | بہن چود کنجر بیغیرت بیغیرت بہن چود کنجر بیغیرت بیغیرت بہن چود کنجر بیغیرت بیغیرت بہن چود کنجر بیغیرت | 1 |
|  | 721 | اس سے پوچھ لینا باقی کوئ تصلی چاہیئے وہ بھی کر دوں گا | 0 |
|  | 431 | سب سے بڑے گٹر کے کیڑے تو تم ہو وا شل ، جو دھرنے میں پیدا ہوئے تھے اور وہیں ڈی چوک میں ہی تمہاری بہن نے بھی پیش کروائی تھی | 1 |
|  | 570 | پرچم کا تقدس تو جان سے بھی زیادہ عزیز ہے۔ ان کنجروں کے مُنہ پر تھوکنا بھی زیادہ باعث خوشی ہے۔ | 1 |
|  | 210 | عمران کی حکومت آتے ہی اسلام اور اہل اسلام پر حملے شروع ہو گئ | 0 |
|  | 707 | تو اگر اپنے باپ کا ہے تو سامنے آ کر بات ہے کتے کے بچے تیری بہن کو بلاول ہاؤس میں لے جاکر چک کروایا ہے پوچھ لینا بغیرت انسان | 1 |
|  | 2700 | تیری گانڈ میں بنا تھوک کے کب | 1 |
|  | 281 | تیرا باپ مادرچود بیچ کے آیا ہے حرام کے پِلّے۔ | 1 |
|  | 129 | عید قربان کے موقع پر بکواس کرنے والے یورپین لوگوں کو اسپین میں بطخ کا گلا گھونٹ کر مارنے کا تہوار بھی دیکھں | 0 |
|  | 2646 | خانصاب نے ثابت کیا ان کا ٹیمپریمنٹ ٹیسٹ پلئیرز والا ہے | 0 |
|  | 2013 | ان کنجروں کے بس کی بات نہیں ہے۔ کراچی کو اس کے اصل وارث کے حوالے کرو۔ | 1 |
|  | 550 | جناح کو پتہ چل گیا تھا کہ ہندو کتنی بغیرت قوم ہے مسلمانوں کا جینا حرام کر دے گی | 0 |
|  | 2935 | کیا سندھ میں بجلی کا نظام بالکل ختم ہوگیا ہے ؟ جب سے آیا ہوں پنکھا چلتا نہیں دیکھا ۔۔ | 0 |
|  | 1299 | پہلے سویت یونین اور اب نیٹو پچاس ممالک کی افواج افغانڈو اغوانیوں کو ماؤں کو چود رہے۔ | 1 |
|  | 2142 | یہ سارے مادرچود گرفتار ہونے کے بعد ہی بیمار کیوں ہوتے ہیں۔ | 1 |
|  | 2288 | کنواری رنڈی | 1 |
|  | 2017 | خان کی مخالفت میں ساری اپوزیشن بکھر کر رہ گئی | 0 |
|  | 3318 | جو دھندے تم سندھی بھڑوے کرتے ھو وہی بیان کر رھے ھو گانڈو گھوڑا رے گھوڑا کے علاوہ تم لوگو… | 1 |
|  | 1869 | یہ صرف عورتوں سے لڑنے جوگے ہیں | 0 |
|  | 2465 | تیرے بعد کس نے باطل کو تیرے لہجہ میں للکارا حیدری | 0 |
|  | 224 | ماں کی کس میں جائے یہ پہلے عوام کا سوچ لو یہ بہن کی لوری تو پھر بھی اچھی جگہ جا رہی ہے | 1 |
|  | 1696 | مادرچود دلے اس اپنے باپ کو بھی سن کیا کہتا ہے | 0 |
|  | 231 | کچھ لوگ اگرکشمیرمیں ہوتےتوبڑےآرام سے انڈین آرمی کو کہتےسرہمیں جوتیاں مارو کیونکہ اسلحہ اور طاقت تو آپکےپاس ہے | 0 |
|  | 361 | لو جی لال ٹوپی والے نے تو اپنا فرض ادا کر دیا ٹوئیٹ کر کے جہاد میں اپنا حصہ ڈال دیا ہزاروں آر ٹی لائیکس بھی مل گیا | 0 |
|  | 1894 | اسلام اور مسلمانوں کی فکر میں دانشواران قوم و قائدین ملت کی راتوں کی نیند حرام ہو چکی ہے، | 0 |
|  | 1951 | کیا اس نوجوان کا جرم اتنا بڑا تھا کہ اسے مار مار کر موت کے گھاٹ ہی اتار دیا جاتا؟ | 0 |
|  | 2892 | یوٹرن لیتے ہوئے آج قوم سے بکواس کرنے کا پروگرام کینسل کر دیا | 0 |
|  | 114 | یہ کس مادرچود کے دل کی بھڑاس نکالی گئی؟ | 1 |
|  | 2747 | بھوکا ننگا بھکاری زرداری | 1 |
|  | 1150 | مادر چود کشمیر بیچنے والوں کے بھڑوے بھروے عوام کو پاگل سمجھ رکھا ہے کتے کے بچے کشمیر بیچ کر اب اس بکواس کا مطلب | 0 |
|  | 826 | ماں اگر زندہ ہو تو وہ آفات سے بچاتی ہے اور مرنے کے بعد بھی اللہ سے اپنی اولاد کی خیر مانگتی ہے | 0 |
|  | 854 | جنرل باجوہ پاک آرمی کے زبردست لیڈر ہیں، چین… | 0 |
|  | 2630 | سردار صاحب بھڑکیں مارنا بند کریں | 1 |
|  | 367 | اوہ کسی کھوتی کے بچو بنا بنایا پاکستان تم کو دیا اور تم بیغرتوں نے اسکا برا حال کر دیا ۔۔ | 1 |
|  | 27 | سعودی عرب کے بادشاہ روزہ دورے پر منگل والے دن انڈیا کا دورہ کر رہے ہیں جن کو پاکستان ہونا چاہیے تھا | 0 |
|  | 563 | اوئے لونڈے کتے اپنی بکواس بند کرو حرامی | 1 |
|  | 1241 | کیسی کتی اپوزیشن ہے کہ مسلئہ کشمیر پے بھی ان حرامزادوں کو سیاست سے شرم نہیں آتی | 1 |
|  | 1685 | ملعونہ کو بیرون ملک بھیجنے والا اور ڈیم فنڈ کے مد میں معصوم بچوں کی پاکٹ منی ھڑپ کرنے والا سلاخوں کے پیچھے | 0 |
|  | 1374 | کھسرا کھسرا ہوتا ہے | 1 |
|  | 1594 | تمہاری تو اوقات ہی نہیں مادر چود فوج کو سامنے لا کر like لیتا ھے خنزیر کی اولاد جتنا یہ ملک تمہارا ھے | 1 |
|  | 842 | او مادرچود | 1 |
|  | 1638 | گشتی تیرا کوئی تعلق اسلام سے دور دور تک بھگوڑی رن دگڑ دلی حرام خور نانی اماں ڈرامے باز بھگوڑی مریم صفدر قطری کی عیاشی کا سامان | 1 |
|  | 2557 | یہ سائینس نہیں | 0 |
|  | 384 | چل بہن چود دفع ھو۔۔۔۔پوری دنیا کو اس وقت کشمیر کی پڑی ھے | 1 |
|  | 1767 | اس منصوبے کے باعث چمکنی سے حیات آباد تک ہزار پودے، سینکڑوں قدیمی درخت اور تاریخی مقامات متاثر ہوئے۔ حیات آباد، ی… | 0 |
|  | 2577 | سبکے سب کتے کی موت میں مرے گے کسی بھی روپ میں ا جائے یہ کتے ٹھوک کے رکھ دیے گے اب انشاء اللہ | 1 |
|  | 586 | بہن چود اے کی ایٹم اے بندہ | 1 |
|  | 1111 | جس کسی نے دنیا کا سب سے بڑا بہن چود بیغرت دیکھنا وہ وہ اس نیازی گشتی کے بچے کو دیکھ لے | 1 |
|  | 1579 | بات ىه هے که جب هم بھارتى چائے والوں کا منه توڑ رهے هوتے هىں تو پىچھے سے پٹوارى اپنى بکواس شروع کر دیتے هىں اور کپت… | 0 |
|  | 2776 | او بیغیرت حکمرانوں شرم سے ڈوب مرو بیغیرتو آخر کس بات کا ڈر ہے تمہیں خدا کی قسم دل خون کے آنسو روتا ہے | 1 |
|  | 3355 | یہ لے اپنی امی جان کی کُس میں تن دے کسی گشتی کے بچے۔ مادرچود، حرام کے پلے | 1 |
|  | 1075 | بے غیرتوں اور کنجروں کا ٹولہ ہے | 1 |
|  | 2947 | کتی کے بچے مل تو ایسے رھے ھو جیسے نسلی غلام ھو ان کے لعنت ہے تم پے کتے پاک آرمی پے بونک رہا ہے حرام کی اولاد | 1 |
|  | 1059 | کھوتی دیا پترا بکواس بند کر ؟؟؟ | 1 |
|  | 3153 | کشمیر ہمارا ہے اور انڈیا کے بھی ٹکرے کرے گے | 1 |
|  | 1012 | ہم مسلمان شہادت کے لیے جیتے ہیں | 0 |
|  | 3160 | تو اگر اپنے باپ کا ہے تو سامنے آ کر بات ہے کتے کے بچے تیری بہن کو بلاول ہاؤس میں لے جاکر چک کروایا ہے پوچھ لینا بغیرت انسان | 0 |
|  | 2462 | کہاں ہیں پٹواری، کھوتا کھا کھا کہ ان کے دماغ بند ہو گئے ہوئے ہیں، لعنتی عورت صرف ٹویٹر پر بکواس ہی کر سکتی ہے، بڑی… | 0 |
|  | 2878 | تم بس کتے کی طرح مریم نواز کی گاڑی کے پیچھے دوڑا کرو | 1 |
|  | 1136 | ٹوئٹر کا میرا اکاونٹ بار بار لاک کرنا افسوس ناک ہے۔ | 0 |
|  | 3194 | سب نے کافی کچھ کہہ دیا مبشر زیدی کو امید ہے موصوف کی طبیعت صاف ہوگئی ہوگی ورنہ مجھے ہی کہنا پڑتا کہ مبشر دُر فٹے مُو تیرا | 0 |
|  | 2123 | گاوں باجا محلہ حاجی خیل سے افسوسناک خبر ۔۔۔۔۔۔۔۔۔ دو فریقین کے مابین فائرنگ کا تبادلہ ہوا ۔۔۔۔ نصیر خان جاں بحق | 0 |
|  | 1734 | سر اس حرام زادے غدار کی بکواس پڑھ لے اس کا تو کوئی بندوبست کرے | 0 |
|  | 2146 | اس کی گانڈ پر اتنے لیتر مارے جائیں کہ یہ سیدہ جہنم رسید ہو۔ | 1 |
|  | 624 | زینب کی عزت لوٹ کر مارنے والا عمران بھی زینب کے جنازے میں شریک تھا ۔ | 0 |
|  | 1115 | بغیرتا تمہارے پاس سال تھے تونے کیا کرا اور کلبوشن کیس تمہارے دور کا تھا کتی کے بچے | 1 |
|  | 2371 | سسستی گشتی دا پتر | 1 |
|  | 1265 | حکمران ہاتھ پر ہاتھ رکھے بیٹھے نہ رہیں۔ | 0 |
|  | 685 | کپتان کو کتے پسند ہیں اور کتوں کو کپتان | 0 |
|  | 1630 | تم جیسے کنجر کس منہ سے دوسروں کو بے غیرت کہہ سکتے ہیں ۔ | 1 |
|  | 3074 | ہماری آنکھوں کے تارے یہ فوجی جوان ہمارے | 0 |
|  | 1662 | ابھی تو تم مادرچود فائدہ اٹھانے کی کوشش کر رہی ہو۔ ملک دشمن | 1 |
|  | 1314 | شنگلی پائیں سے تعلق رکھنے والا قطم خان کا بھائی دائم خان لالا ولد باعث خان وفات ہو گئے۔ | 0 |
|  | 2814 | مجھے ہزارفیصد یقین ہےکہ تمھارے چہرے کے پیچھے وہ گشتی ہوگی جس کو ماں اور بہن سمیٹ ایڈز کا مرض ہے | 1 |
|  | 3236 | خیر اگلی بار جب ایسی بکواس کرنے کا سوچو تو اپنے اداکار… | 0 |
|  | 2110 | یہ ہے ان بیغیرت لوگوں کا کنجر پنا نا یہ خود کام کرینگے نا ہی کسی کو کام کرنے دینگے | 1 |
|  | 1779 | مشرف نے تیرے جیسی رنڈی کو اپنے فارم ہاوس میں بلا کے مجرا کرواتا تھا گشتی بیغیرت غدار | 1 |
|  | 1956 | UNSC کا آج کا اجلاس پاکستان کی سفارتی فتح ہے | 0 |
|  | 645 | بہن چود کُتّے کے پُتر کھوتے کی نسل | 1 |
|  | 1835 | یہ بھوسری کے ساراے مل کر ہم کو چھوٹیا بنا رہے ہیں مادرچود کے بجے | 1 |
|  | 2699 | آج پوری دنیا میں انسانیت پسند لوگ بھارت کی آزادی پر بلیک ڈے منا کر بھارت کے منھ پر طمانچہ مار رھے ھیں | 0 |
|  | 2622 | صحیح بات یہ وہی نمک حرام ہے جو کراچی کے حصہ کا پانی چوری کرتے ہے اور کراچی والوں کو ہی بیچ دیتے ہے۔ | 0 |
|  | 1681 | جس خبیث کو اپنی ماں پر رحم نہ ایا وہ بے ُسرہ بے شرم ہمارے خان کے بارے میں بکواس کرتاہے | 1 |
|  | 2070 | خان تیری گانڈ بھی مارے گا اور تجھے پتہ بھی نہی چلے گا گھٹیا عورت | 0 |
|  | 2978 | یہی ایک امید تھی مگر یوتھیوں کے گدھے وجیراعجم نے اسکے بھی تمام تر ڈاکو منٹ امریکیوں کے حوالے کر دئیے | 0 |
|  | 2839 | اب تیری گانڈ مارنے کا ٹائم آنے والا ہے تھوڑا انتظار کرو کنجروں کی ترجمانی کرنے والی گشتی | 1 |
|  | 665 | کوئی مرد کا بچہ موجود نہیں تھا اتنے لوگوں میں سارے ھجڑے کھڑے ہیں | 1 |
|  | 2925 | نیا پاکستان کے جھوٹے نعرے ہم سے کیوں لگوائے جب حرکتیں وہی ن لیگ والی ہی کرنی تھی | 0 |
|  | 2071 | کوئی شک نہیں پورا دلا ہے | 1 |
|  | 1469 | او بد بخت انسان کیوں بکواس کرتا ہے | 1 |
|  | 2398 | لعنت ھے تیری بزدلانہ سوچ پر گیدڑ کی طرح زندگی گزاروں اور کتے کی موت مرو بے غیرتوں | 0 |
|  | 3175 | جب سے گورمے بیکری سے چھپکلی نکلی ہے گورمے اور یہ سائیکو جرنلسٹ تحریک انصاف کیخلاف بکواس کر رہے ہیں | 0 |
|  | 1621 | تو بہن چود کتی عید پر بھی بکواس کرتی ھو جرنیلوں کی کتی کسی گھٹیا نسل کی | 1 |
|  | 1440 | کون کہتا ھے مرشد عمر بھر نبھاہ کیجیے بس آئیے بیٹھیے تباہ کیجیے فناء کیجیے | 0 |
|  | 2672 | پاک فوج کاایل او سی پربھارتی سیکیورٹی فورسزکومنہ توڑجواب | 0 |
|  | 485 | وہ کان کھول کرسن لے باجوہ صاحب نے تین سال کہیں نہیں جانا یہ ڈنڈا یاد رکھنا | 1 |
|  | 31 | لعنتی لوگ | 1 |
|  | 799 | پایان روز یه خالی عظیمی هست که باید با یه نفر به اشتراک گذاشته بشه | 0 |
|  | 1209 | لعنتی مردود مبشر ذیدی کا کام ہی اسلام اور اسلامی احکام شریعت پرتنقیدکرنا ہے | 0 |
|  | 806 | یہ بہن چود نے بھی وڈیو بنائی ھے وہ بڑا حرامی سوور کی اولاد گشتی دا بچہ اے | 1 |
|  | 109 | ریاستی اداروں، ملک وطن کے خلاف جو بکواس کرے ادارے اسے گرفتار کریں | 0 |
|  | 2897 | دوستو یہ رنڈی اس قابل نہیں کہ اسکے بکواس سنو | 0 |
|  | 1627 | یہ بہن چود سیاسی قیدی نہیں ہیں یہ حرامخور نسل کے حرامی غدار ہیں ان کی تو لاشیں بھی نہیں ملنی زندہ تو بہت دور کی بات ہے | 1 |
|  | 3317 | ھمیں بغیرت کہنے والے تم پر تو دلالی اور بھڑوہ گری کا انٹرنیشنل ٹیگ لگا ہوا کہ ماں بھی بیچ دیتے ہو | 1 |
|  | 899 | انڈیا والو کان کھول کر سن لو تمہارے لئے ہمارا ایک شیخ رشید ہی کافی ہے | 0 |
|  | 819 | دونوں ہی مادرچود ہیں | 1 |
|  | 2011 | امید ہے اتنے سارے ایک ہی طرح کے جوابات بڑھ کر تمہاری طبیعت صاف ہوگئی ہو گی | 0 |
|  | 3124 | یہ حرامی سارا ملک گروی رکھ کر لوٹ کھسوٹ کرکے اجڑا ہوا ملک خان کے حوالے کر گئے تھے تاکہ خان ناکام ہو جائے | 1 |
|  | 1507 | او کسی چمکیلے بڑکیلے لنڈ کو چوسنے کی خواہش کرنے والی گشتوڑی نوازشریف بڑوے کے بیان سن | 1 |
|  | 1090 | تیرا بھی کوئی باپ انکل چاچا تایا ہے کہ بن مانس کی اولاد ہو گشتی نا ہو | 1 |
|  | 777 | کسی رنڈی کی اولاد ہو تیرے جیسے اپنی باجی پیش کر کے سائیکل کرائے پر لیتے ہیں | 1 |
|  | 2238 | تمھاری یہ کُتے والی بکواس پچھلے سال سے سُن رہے ہیں لہذا اپنا گند سے بھرا منہ بند کر لو زلیل پٹواری | 1 |
|  | 297 | کوکین نیزی کیا یہ ملک کنجر خانے کے لیے بنا تھا یہاں کنجروں کو خود ویزے لگوا کہ دے کر بلایا جاتا ہے | 1 |
|  | 2134 | بہن چود پٹواری ۔۔۔خود سے اپنے ابو نواز شریف چور کی تاریخ لکھنے بیٹھ جاتے ہیں۔ | 1 |
|  | 919 | کسے ٹیکسی دے | 1 |
|  | 1020 | پاکستان کی صحافت کا مہا حرامی ٹٹے چُک مادرچود | 1 |
|  | 3054 | بے غیرت حکومت نے آمریت قائم کی ہوئی ہے اس بے غیرت کا انجام بھی بڑا عبرت ناک ہوگا لعنت ہو بےغیرتوں پر | 1 |
|  | 2157 | لفظ بکواس بہترین لگا ایسی صورتحال میں | 0 |
|  | 2187 | یہ ہندو تو نسلوں سے نمک حرام ہیں پھر بھی ہم ان کو نمک دے رہے ہیں۔ ۔۔۔۔۔ان کا فوری طور پہ نمک بند کر دینا چاہے۔ | 0 |
|  | 2691 | انار کلی تو پھر بھی اپنے وقت پر دیوار میں چُنوا دی جائے گی | 0 |
|  | 552 | کچھ تصویریں بولتی نہیں بلکہ چیختی ہیں کہ ڈرامہ سیریل بند کرو | 0 |
|  | 1656 | اے تے شکلوں ای کوٸی گشتی لگدی اے۔ | 1 |
|  | 286 | وزیراعظم آزاد کشمیر کی تقریر سن کر دل خون کے آنسو رو رہا ہے | 0 |
|  | 3344 | ڈائیلاگ ہی اسکا اصل حل ہے مودی نے اگر گھٹیا حرکت کی ہے تو دنیا ساری دیکھ رہی ہے | 0 |
|  | 1224 | برداشت کرنا یعنی وہ بات جس کے بدلے میں آپ کچھ کہہ نہیں سکتے کچھ کر نہیں سکتے ۔ | 0 |
|  | 2999 | سر ان کی اور ان جیسوں کی طبیعت صاف کریں قانون کا نفاذ لازم کر کے | 0 |
|  | 3127 | انڈین آرمی کو انشاءاللہ تعالی اللہ پاک عزت پوری دنیا میں عبرت کا نشان بنا… | 0 |
|  | 220 | ایک سال پہلے جب خاتم النبیین کہتے ہوئے عمران خان کے پسینے چھوٹ گئے تھے، سمجھنے والے اسی وقت سمجھ گئے تھے | 0 |
|  | 2837 | اس سے بڑی ذلت اور کیا ہو گی کہ کفار مسلمانوں کے فیصلے کیا کریں | 0 |
|  | 1737 | اور ہم نے کرپٹ اپوزیشن کو دیوار میں چنوا دیا ہے | 0 |
|  | 587 | بیغیرتوں کی ٹولیاں بس اور کچھ نہیں | 0 |
|  | 1125 | ایسے مادرچود یوتھئے ہیں کہ کشمیر ہاتھہ سے نکل رہا ہے لیکن یوتھئے ابھی بھی کشمیر کی ثالثی پر نیازی کی واہ واہ کررہے… | 1 |
|  | 953 | چپ مادرچود بلکل چپ، جن کتوں کا نام تو لے رہا ہے نا، انکا مالک بھی وہی ہے جو تیرا ہے۔ تجھ جیسے کُتے دلے کے منہ نہیں لگتے ہم | 0 |
|  | 2968 | وزیر احتساب اور ایف بی آر کے ہیڈ نے کہا باہر پڑے دو سو ارب کی کہانی بکواس تھی، تو جو جو حرامی اس بارے بکواس کرتے رہ | 1 |
|  | 352 | بہن چود تیری دیدی چور ہے | 1 |
|  | 57 | ان بیغیرتوں نے تو اپنی بہن پیش کی ھوئی تھی آج بلو کھسری نے بھی اپنی بہن پیش کر دی ھے بلاول حرامزادہ زرداری فراڈن کی گرفتاری | 1 |
|  | 748 | ہان صرف تیر نہین کو چھوڑ کر گشتی کے بچے | 1 |
|  | 2360 | یہ سب کنجر لوگ ہے اور کنجروں کے گھر کنجر ہی پیدا ہوتے ہیں مجاہد نہیں | 1 |
|  | 1147 | جناب آپ انڈیا کو جو معلومات دے رہے ہیں ہیں وہ ان کو معلوم ہے ہم سے زیادہ ترقی یافتہ ہے آئی ٹی کے میدان میں | 0 |
|  | 3462 | نبی کتابیں لکھا نہیں کرتے | 0 |
|  | 157 | گشتی اور رنڈی کے بچے نواز شریف کی اصلی اولاد لندن میں بیٹھی مزے کر رہی ہے | 1 |
|  | 3352 | واقعی سب کے سب مادرچود ایک صفحےپر ہیں | 1 |
|  | 588 | بٹھی کتے خنظیر مادرچود یہ وقت نہیں ہے کے تو ابھی بھی ان پر وار کصے اپنے باپ پر کر یا اس کے حواریوں | 1 |
|  | 1988 | ھولناک ویرانے اور اذیت ناک سناٹے جنگلوں یا بیابانوں میں نہیں انسانوں کے اندر بھی ھوتے ھیں۔ | 0 |
|  | 1093 | صائمہ گشتی کے دلال بھائی جس طرح تیرا لیڈر منی لانڈرنگ کے لئے مریم بھگوڑی بیٹی کا استمال کرتا ہے اسی تو | 1 |
|  | 629 | راولپنڈی اسلام آباد میں آزادی کا جو جشن منایا جا رہا ہے اسے دیکھ کر میرا دل خون کے آنسو رو رہا تھا | 0 |
|  | 1934 | تیری بہن نہی بہن چود باپ کی چدائ سے مکر گیا مادر چود کی اولاد | 1 |
|  | 368 | بہن چود سارے ملک تباہ کر گیا | 1 |
|  | 3229 | مراد ہم تیرا گانڈ مارے گا سرسوں کا تیلُ لگا کر | 1 |
|  | 1283 | کسی بھی انسان کی جب تک ضمیر آزاد نہ ہو تب تک یوم آزادی منانا بکواس ہے | 1 |
|  | 1622 | ایک تو چوری اوپر سے سینہ زوری بےشرم قسم کے انسان شرم نہیں آتی | 1 |
|  | 399 | اگر تب تو نے اشمت سے اپنی چوت نہ پڑوائ ہوتی تو آج تیرے گھر کُتے کا بچہ نا ہوتا مادرچود رنڈی | 1 |
|  | 652 | ابھی کنجروں کے حوالے کر دیں تمھارا چورن نہیں بکنے والا بچو ۔جا شاباش جا کے گانے گا اپنی بیہودہ آواز میں۔آج کیلئے اتنا کافی ہے | 1 |
|  | 937 | اگرآپکے ملک میں سرمایہ کاری دوست ماحول ہوتا تووہ پاکستان میں کرتا | 0 |
|  | 557 | ہم نے انتہا پسندی کی تمام حدود کراس کر دی ہیں ریحان کا قتل طمانچہ ہے۔ | 0 |
|  | 2173 | سابقہ حکومتوں بارے نیازی صاحب کے ادا کیئے گئے الفاظ آج ان کی اپنی حکومت اور ساکھ تباہ کرنے میں اہم کردار ادا کر رہے ہیں | 0 |
|  | 1973 | بہن چود دلے اسُ ٹیم توُ روز ٹی وی روز بھونکتا تھا کہ میں پنامہ لیگ لے کے آیا ہوں اب تیری بہن نوں لن وڑ گیا اے | 1 |
|  | 1740 | بکواس بندکرو اور عورت کارڈ مت کھیلو کس نے کہا ہے چوریاں کرو اور fake اکاؤنٹ بناؤ تم تو ویسے ہی بری گھٹیا عورت ہو | 1 |
|  | 2608 | یہ دیکھ رنڈی کی نسل ، یہ مادرچود غدار وطن ہے | 1 |
|  | 876 | تیری ماں دا ٹھوکو خان کُتی ماں دا بچہ ویچ آیا ای کشمیر مادرچودو غدارو تم لوگوں گی گانڈ میں ایسا ڈنڈا… | 1 |
|  | 3339 | لن کی اکلوتی اولاد دفعععععععععع ہوجااااا، مجھ پہ نہ بھونک بھڑوے، مادرچود | 1 |
|  | 943 | ايسی گھٹيا حرکت کرنے پر گورنمٹ کو ايکشن لينا چاھيے اور ايسے لوگوں کو سزا ملنی چا ھيے۔ | 0 |
|  | 1982 | یعنی تبدیلی نہیں آئی ایک انتقام لینے والا گروپ سلیکٹرز لیکر آئے ہیں جتنے آپ نے نام گنوائے ہیں سب جیل میں ہیں | 0 |
|  | 1625 | واقعی سب کے سب مادرچود ایک صفحے پر ہیں | 1 |
|  | 1139 | غیور بیٹی ریاست کے خلاف نعرے نہیں لگاتی اس گشتی کو جیل میں ڈالو | 1 |
|  | 1985 | مادرچود، دلے کی بچی زنا کی پیداوار یہ دیکھ تیرے حرامی باپ نے کیسے پاکستان اور کشمیر سے غداری کی تھی۔ حرام کی اولاد | 1 |
|  | 308 | لیں جی ایک تو بھارت سے ڈر ک… | 0 |
|  | 800 | جا او کتے بچے سور کی اولاد گانڈو بہن چود | 1 |
|  | 921 | نمک حرام یوٹیوبرز اور ایکڑز جو ابھی تک اس ڈر سے کچھ نہیں بولے | 1 |
|  | 414 | یہ کتے کا بچہ کیا بکواس کر رہا ہے ذرا ملاحظہ فرمائیں کہ کشمیر اور پاکستان انڈیا کا حصہ ہیں | 0 |
|  | 46 | کیوں جھوٹ بول کر اپنے آپ پر لعنت ڈالوا رہی ہو | 0 |
|  | 242 | اسلام میں ہتھیار اٹھانے والے اور دین کو نقصان پہچانے والوں کی سرکوبی کیلیئے جہاد فرض ہے۔ | 0 |
|  | 3076 | شاباش عمران خان آپ نے اپنے دانشمندی ، دلیری اور مخلصانہ کوششوں سے کشمیر کا مسئلہ عالمی مسئلہ بنا دیا۔ | 0 |
|  | 923 | پاک آرمی نے ہندو فوجی جہنم واصل کر دیے اور بنکر تباہ کر دیے | 0 |
|  | 2317 | اس کنجر انسان کو کھسرے کو کوئی روکنے والا نہیں ہے | 1 |
|  | 724 | کتنے حرامی ہیں نا تمھارے سالے بوڑھے بیمار باپ کو ایک بار جیل ملنے نہیں آۓ ۔ خود اشتہاری ہیں بوڑھا باپ | 1 |
|  | 48 | اگر بیغیرتوں کے سر پے سینگھ ھوتے تو آج بلاول بارہ سنگھا ھوتا۔ کھسرا کھسرا ہوتا ہے | 1 |
|  | 20 | تاریخ میں یاد رہے گا کہ پاکستان کے ڈی جی آئی ایس پی آر میجر جنرل آصف غفور صاحب نے پورے بھارت کی نیند حرا… | 0 |
|  | 757 | بڈھا خوسٹ بڑ بڑ کرنے والا خبیث انسان | 1 |
|  | 1312 | بلو رانی کس گندی نسل کے کتے بہن چود حرام خور کی گندی اولاد کنجر انسان بغیرت انسان | 1 |
|  | 1269 | آج تک سوائے انسان کے کسی ذی روح نے اپنے مستقبل سے مایوس ہو کر خودکشی نہیں کی | 0 |
|  | 96 | تم جس طرح بھی بھونکتے رہو۔لیکن خان نے تمھاری گانڈ ماری ہے | 1 |
|  | 1441 | جب آپ کی چوت ماری جا رہی تھی تب کا آپ کو پتہ ہے یا نہیں | 0 |
|  | 3464 | مودی کو گالی دینے سے کشمیر آزاد ہوگا؟ | 0 |
|  | 217 | اس ببر شیر نے لگتا ہے تیری بہن کو چود دیا ہے دلے۔ | 1 |
|  | 1906 | اسلۓ کہ تمھارا آقا کے آگے لیٹا ھوا تھا۔ بیغیرتوں کچھ تو آپنی عاقبت کا بھی سوچ لو | 0 |
|  | 1290 | لخ دی لعنت تیڈے منہ تے بے غیرت نمک حرام | 1 |
|  | 1721 | مودی کی یہ بات جیالوں اور پٹوریوں کے منہ پے طمانچہ ھے کہ جو کہتے ہین کشمیر کاز کو عمرن نقصن دے رہا | 0 |
|  | 63 | کب سندھ میں بٹھو مرے گا اورکب ان بےغیرتوں اورحرام خوروں سے پاکستان کو نجات ملے گی | 0 |
|  | 2874 | کنجروں اور غداروں کا غیرت سے کوئی تعلق نہیں ھوتا ۔ اس لئے غیرت وغیرہ دلانے کا کچھ فائدہ نہیں | 1 |
|  | 855 | اوئے بہن چود انڈیا ھی چلے جاو کسی گشتی کے بچے | 1 |
|  | 335 | نکل لوڑے پھیلی فرصت میں نکل بھوسڑیکے مادرچود | 1 |
|  | 1979 | ان بیغیرتوں کوالله کا خوف نہیں ہے | 1 |
|  | 467 | کامیاب خارجہ پالیسی کی وجہ سے دنیا میں پاکستان کے حصے میں عزت آئی اور انڈیا کے حصے میں ذلت آئی | 0 |
|  | 2197 | مولیاں اندر کروا کروا کے اس کی گانڈ اس قابل نہیں رھی کہ ماری جاۓ | 1 |
|  | 796 | تو جائو مرو جہاد کرو بک بک کرنا جہاد نہیں منافق بنے بیٹھے ہو اگر علاج معلوم ہے تو کرتے کیوں نہیں | 1 |
|  | 2300 | اس بچے کو مارنے کا حق ان دونوں کو کس نے دیا تھا کیا قانون پاکستان سے ختم ھوگیا ھے | 0 |
|  | 2663 | ابھی کافی بے شرم اور نمک حرام باقی ہیں جو یوم سیاہ نہیں منا رہے | 0 |
|  | 3204 | گوادر کے ماہی گیر پہلے سے پریشان ہیں اب اور زیادہ پریشان کردیے گئے | 0 |
|  | 74 | اچھا آپ نے بہن دی ہوئی ہے عابد شیر علی کو پھر تو آپ کی عزت کرنا بنتی ہے | 1 |
|  | 2938 | ہاں جی کُتی کے بچے کیا بنا تیری اس ماں کا تیری ماں پی ٹی آئی سے ہے | 1 |
|  | 861 | بھارت کی نسلیں بھی یاد کریں گی ک اس نے کس قوم کو للکارا تھا ۔ بھارت کے ٹکڑے صرف پاکستان ک ہاتھوں ہی لکھے ہیں۔ | 0 |
|  | 2265 | پنجاب سے لوگوں کو منتخب کرنے والے میرے… | 0 |
|  | 30 | بیٹا تو یہ اپنی بکواس اپنے پاس رکھو | 0 |
|  | 2122 | آرمی چیف جنرل قمر جاوید باجوا دشمن کے حملوں کو منہ توڑ جواب دینے کی صلاحیت رکھتا ہے اللہ اس کو لمبی عمر عطا فرمائے آمین | 0 |
|  | 2745 | ہہ ستی گشتی کا ماں کا بچہ ہے یہ ایک ماں سے ھوتا تو اسی کھبی بات نہیں کرتا۔ | 1 |
|  | 3305 | تواڈی اک واری فیر ضرورت جے | 0 |
|  | 1041 | کا برانڈ ایمبیسیڈر یا نمائندہ بننے کے لیے یا تو کنجر ہونا ضروری ہے یا کنجری ہونا | 1 |
|  | 2237 | یے عورت کے نام پر بدنامی کا دھبہ ہے | 0 |
|  | 2501 | اس سے بھی نکال دونگا اور اسکی طبیعت صاف کروں گا | 0 |
|  | 3331 | ففتھ جنریشن وار کی پہلی جیت ڈی پی کالے کرکے دشمن کے دانت کھٹے کردیے | 0 |
|  | 2115 | تیری ماں چودوں تیرا اماں کا یار ھندؤں کی آنکھوں میں آنکھیں ڈالنے والے برھا… | 1 |
|  | 2915 | اپنی گشتی ماں سے پوچھ عاشق راجپوت کون ہے وہ بھی بتاے گی تھے پیدا کرنےیں حصہ بھی ہے۔ | 1 |
|  | 2375 | کہ کاش آج کوئی باغیرت مسلمان حکمران ہوتا تو کوئی گاۓ کا پیشاب پینے والا انہ ہوتا | 0 |
|  | 69 | لوٹا سیاستدانوں نے اور کھال اتار رہا ہے عوام کی | 0 |
|  | 1771 | پاکستان کے دشمنوں کو عبرت کا نشان بنا دے | 0 |
|  | 457 | سیدوالا دریاٸے راوی پل کی رابطہ سٹکیں تعمیر نہ ہونے کے باعث راستے پر پانی جبکہ مسافر پانی سے گزر رہے | 0 |
|  | 1077 | اس ببر شیر نے لگتا ہے تیری بہن کو چود دیا ہے دلے۔ | 1 |
|  | 2233 | ایک معصومانہ سوال سے ہیں۔ آپ نے ابو بدل لیا ہے مولانا فضل الرحمن کو کن کن چیزوں سے نوازا جا رہا یے | 0 |
|  | 2163 | کنجری کا بچہ ہرامی دلا عدالتیں جھوٹی ہیں | 1 |
|  | 2562 | اچھا تو تیری ماں شہباز گل کی باپ نے چودا تھا اور تیرا باوجی کی گانڈ تیرے آقا قادیانی | 1 |
|  | 2353 | اوئے مادرچود ہیجڑو تم سے تو ہیجڑے اچھے ہیں جو کبھی عورت ہہ ہاتھ نہیں اٹھاتے انشاءاللہ تمہارا وقت بہت | 1 |
|  | 2972 | پی ٹی آٸ حکوت کو ڈی چوک کے دھرنے کے بعد کنجروں کی حکومت تو سب ہی کہتے تھے | 1 |
|  | 2428 | کوہی کتے کا بچہ ہی ہوگا مادرچود فنکشن ارینج کروانے والا گولی مر دینی چاہے بھارتی فنکار کو بھی | 1 |
|  | 1431 | بہن چود کنجر بیغیرت بیغیرت بہن چود کنجر بیغیرت بیغیرت بہن چود کنجر بیغیرت بیغیرت بہن چود کنجر بیغیرت بیغیرت بہن چود | 1 |
|  | 708 | یہ جوتا چاٹ بھٹی اور اس کے للو بھانجے کے منہ پر طمانچہ ھے ۔ | 0 |
|  | 2104 | ریاست عملی طور پر کنجروں کو تھوپ دی گئی ھے ان دیکھنے کو یہی کچھ ملے گا | 1 |
|  | 1380 | چوتیے تو تم ہو کسی کنجر کی نسل ، مادرچود بیچا تو تمہارے چور گنجے نے کشمیر کو | 1 |
|  | 328 | چل مادرچود | 1 |
|  | 2106 | اللہ بھگوڑی مریم جو اج جیل میں ہے ایسی چور، دھوکے باز، جعلساز، بھگوڑی، اور ماں کی بیماری پر سیاست کرنے والی بیٹی، بہن کسی کو نہ دے | 1 |
|  | 36 | بہت برا کیا آپ لوگوں کہ رات بجے لے کر گئے ۔۔ سالی کتی کو ننگا کر کے گھسیٹ کر مار مار کر جیل لے کر جاؤ | 1 |
|  | 3456 | مقبوضہ جموں و کشمیر میں صورت حال بہت پریشان کن ہے، سویڈش وزیر خارجہ | 0 |
|  | 3372 | لعنتی نشی کتا بیغرت سلیکٹڈ زانی کنجھر خبیث ذلیل منافق جھوٹا قادیانی دلا حرامی پالتو ثور | 1 |
|  | 564 | تو کوئی بڑا بہن چود ہے۔۔۔۔کسی کتی کے بچے۔۔۔کیوں اپنی ماں چدوانی یے تم نے سالے | 1 |
|  | 2491 | دلال ھے یہ بہن چود | 1 |
|  | 2118 | مادر چود تواپنی ماں کو چودنے والا یوتھیا ہے کسی خنزیر کی اولاد | 1 |
|  | 2032 | یہی غنڈا گردی لگائی ہوئی ہے بیغیرتوں نے جب جس کو دل چاھا اٹھا لیا، لوکپ میں ڈال فیا ، یا گولی مار دی، | 1 |
|  | 2494 | تمہیں کیا مل رہا ہے ایسی گھٹیا بکواس کر کے | 0 |
|  | 346 | بگڑیاں بنانے والا ،اولادیں دینے والا ،رزق دینے والا زندگی موت دینے والا عزت ذلت دینے والا صرف ایک ھی | 0 |
|  | 2831 | ہر اُس شخص کی گانڈ مارے گیِ فوج جو پاکستان سے غداری کرے گا، چاہے وہ کسی بھی قوم سے ہو، ہر غدار کو انجام تک پہنچایا جائے گا | 1 |
|  | 3446 | اس بزرگ کی طرف دیکھ کے کوئی رحم کھاو کشمیر پر او بیغرت حکمران تیرے شکل پر لعنت جس جس نے بھی اس بیغرت کو ووٹ دیا | 1 |
|  | 584 | ہمارے آنسو خون کے یہ داغ کیسے مٹا سکیں گے۔۔۔ | 0 |
|  | 1390 | ایک چوری اوپر سے سینہ زوری | 1 |
|  | 3375 | آج سے پہلے تک یہ بیغیرت نکما جھوٹا کوکینی کیا بکواس کرتا تھا لیکن آج اپنے آقا کو ایکسٹینشن دیکر بوٹ چاٹنے کی انت… | 0 |
|  | 808 | یہ کیا بکواس بکتے ہو | 0 |
|  | 1954 | پیشاب پینے والوں اپنی بکواس بند رکھو نہیں تو ہم پاکستانی تمہاری بکواس کے ساتھ ساتھ تمہارہ گانڈ بھی بند کر دیں گے | 0 |
|  | 131 | پاکستانی افواج ہرقسم کی مہم جوئی کامنہ توڑ جواب دینے کی صلاحیت رکھتی ہیں، | 0 |
|  | 1652 | اور رہی بات اگنور کرنے کی تو وہ نہیں ہوتا کوئی پاک فوج کے خلاف بکواس کرے۔ | 0 |
|  | 95 | گشتی کی بچی کو بتاٶ اب کیوں بند کیا ہے رنڈی کی اولاد | 1 |
|  | 3060 | اوئے کھسرے تُو وہی ہے نا جس کے آباء نے خالصتان تحریک کے لوگوں کی لسٹیں انڈیا کو دی تھی | 1 |
|  | 1563 | کسی ایک مادرچود نے ڈھنگ کا جواب نہیں دیا انڈیا کو | 1 |
|  | 617 | لگتا ہے سفینا نے اس عمر میں شیخ رشید کے لوڑے کے چوپے لگائے اور کھڑا نا کر پانے کی خفت میں سزا ملی | 1 |
|  | 1397 | تیرا پورا خاندان دلا لعنت ہے تیری گندی زبان پہ یہی کچھ سیکھا تم نے ؟ | 1 |
|  | 992 | مت بکواس کرو | 0 |
|  | 515 | افواج پاکستان کی جوابی کاروائی میں افسر س… | 0 |
|  | 1768 | ہم تیار ہیں پاکستان آرمی زندہ باد | 0 |
|  | 637 | یا اللہ جس جس نے پیارے وطن کے ساتھ کھلواڑ اور غداری کی ،اسکو نشان عبرت بنا | 0 |
|  | 996 | اسٹیبلشمنٹ نے مریم نواز کے آگے ہتھیار ڈال دیے۔ | 0 |
|  | 2842 | ڈبہ پیر نے پوری پاکستانی قوم کو اپنے زندہ درگور مزارعین سمجھا ہوا ہے | 0 |
|  | 1837 | کمینہ بغیرت جو منہ میں آۓ بکواس کرتا ہے حرام خور منہ پہ مٹھاس پیٹھ پیچھے بکواس | 1 |
|  | 571 | کتے کی موت مرو تم لوگ سارے لعنت ہے تیری پیدائش پر بیغیرت | 1 |
|  | 35 | اس بے شرم کی بھی سن لو ۔۔ چل دفع ہو جاؤ لنڈے کے بدمعاش | 1 |
|  | 639 | تو بہت ہی کسی گشتی کا بچہ ہے مادرچود | 1 |
|  | 3498 | قسم لے لیں ان کنجروں کو جو مرضی کہیں میں ناراض نہیں ہوتا | 1 |
|  | 2354 | آئندہ طاھر مغل کو ھیرا منڈی کا دلال سمجھا جائے کیونکہ یہ شکل سے لگتا بھی ھے بہن چود | 1 |
|  | 1829 | آج کے دن لندن اور نیو یارک میں پاکستانی کمیونٹی ناچ ناچ کر جشنِ آزادی مناتی ہے | 0 |
|  | 3492 | یا اللہ پاکستان کو ایسا لیڈر عطا کر جو جہاد کے جزبے سے سرشار ہو ۔جو مظلوم کشمیری مسلمانوں کو آزاد کروا سکے ۔ | 0 |
|  | 1876 | حافظ سعید صاحب کی رہائی کے لئے آواز بلند کرو ورنہ ظلم کے پہاڑ ٹوٹیں | 0 |
|  | 2857 | آج کل کتے بہت بھونک رہئے ہے ہر آنے جانے والے کو کاٹ رہئے ہے اب ان کتوں کو مارنے کا وقت ہوا چاہتا ہے | 1 |
|  | 3101 | اللہ تعالی کی ذات پر ایمان اور توکل علی اللہ ، ایٹم بم ، ساری افواج ، اور ھر قسم کے اسلحہ سے طاقتور ھتھیار ھیں | 0 |
|  | 1230 | اس مادرچود نے تو اپنی ماں کو بھی چیلنج کر دیا تھا کہ ثابت کرو تمہارا خاوند ہہی مرا باپ ہے | 1 |
|  | 1950 | بے شک صحیح کہا کہ مردوں سے لڑے۔ بلاول ہجڑے سے کیا لڑنا بلاول حرامزدہ زرداری | 1 |
|  | 284 | اور جس طرح سے تم بیغیرتوں نے عوام کو بے دردی سے لُوٹا ہے اس سے تم لوگوں کا حرامی پن نظر آتا ہے | 1 |
|  | 3499 | شرم آنی چاہیے ان حرامیوں کو یہ بکواس کرنے سے پہلے جھوٹے سالے | 1 |
|  | 2749 | مگر آپ صاحبان کو سوچنا چاہیے کہ یہ سب چور ھے کوئی شرافت کی وجہ سے جیل نہیں جارہیں ہیں | 0 |
|  | 2270 | گل تم خود بیغرت ہو اور دوسرے بیغرت کو کوٹ کر رہی ہو۔ | 1 |
|  | 208 | ابھی تو تم مادرچود فائدہ اٹھانے کی کوشش کر رہی ہو۔ ملک دشمن | 0 |
|  | 1120 | کتے بڑوے تو بچپن میں گانڈو رہا ہے بے غیرت دلال لعنت تیرے پہ | 0 |
|  | 1042 | یا اللہ دل خون کے آنسو رو رہا ہے یا اللہ کشمیر کی غیب سے مدد کر دے یا اللہ بہت قربانی ہو گئی انکی یا اللہ سن لے | 0 |
|  | 3346 | وہ اس لیے کہ کل کو کوٸی یہ کہ نہ سکے۔کہ دیکھو مجھے تو پاکستانی عوام نے زلیل تو نہیں کیانہ۔اس خاندان سے | 0 |
|  | 719 | ارشادبھٹی بوٹ پالیش کر رھا ھے اور بکواس کر رھا ھے صحافت کا غلیظ ترین کردار ھے یہ شخص | 1 |
|  | 171 | اے سب تیرے کتے دے بچے لوہار کا کام دلہ نے بہت راز بتایا بے غریت | 1 |
|  | 3435 | مادرچود فرانس والی فیسلٹیاں بھی دو کراچی میں ۔۔ بیغیرت آدمی ۔۔ | 1 |
|  | 3095 | میجر جنرل آصف غفور صاحب، ہم سب پاکستانی آپ کے تہہ دل سے مشکور ہیں آپ ہی کی وجہ سے آج دشمنان پاکستان اور دشمنان اس… | 0 |
|  | 1978 | جس انسان کی زندگی دلالی اور PC ہوٹل میں دگڑ دلّا بن کے گُزری ہو اُس مادر چود کو ہاشوانی کنجر کہیں | 0 |
|  | 1755 | کتی کے بچے مل تو ایسے رھے ھو جیسے نسلی غلام ھو ان کے لعنت ہے تم پے کتے پاک آرمی پے بونک رہا ہے حرام کی اولاد | 1 |
|  | 1148 | کشمیر خون کے آنسو رو رہا ہے | 0 |
|  | 1210 | جس کا کام چل جاتا ہے ، فرعون بن جاتا ہے۔ ان سب حرامیوں کو حوالات میں چمڑے کے چھتر مروانے چاہئیں | 1 |
|  | 3251 | یہ بھی عمران خان کا کارنامہ ہے اس نے کنجروں چوروں ڈاکوؤں اور انکی ناجائز اولادوں کو بھی غیرت کا درس دلا دیا ہے | 1 |
|  | 1553 | اگست پر آپ بھارت کو کیا پیغام دینا چاہیں گے؟ میرا یہ پیغام ھے کہ کشمیر سے نکل جاو ورنہ کتے کی موت مارے جاو گے کشمیر ہمارا ھے | 0 |
|  | 1877 | یہ کراچی کی تصویر ہے جب رینجر کراچی میں اپریشن کر رہی تھی یہ مریم قطری کا دگڑ دلا لگتا ہے جس نے ٹویٹ کیا ہے | 1 |
|  | 2676 | مادرچود نیب کی گاڑی کے آگے لیٹا کیوں نہیں تو | 1 |
|  | 749 | اس کتے حرامزادے، دلے، دلال، بغیرت، لعنتی، کھوتے کی طبیعت سب لوگ صاف کریں تا کہ اس دو ٹکے کے جاہل کو اپنی اوقات کا پتہ چل جائے | 1 |
|  | 462 | اب بکواس بند کردو اگر فوجی نقل و حرکت رپورٹ ہی کرنی ہے تو ہندوستانی کرو بہن چود | 1 |
|  | 803 | آپکا زونگ g g کیسا چل رہا ہے۔ میرا تو بکواس چل رہا ہے بار کمپلینٹ کر چکا انباکس میں بولا کر خاموش ہو جاتے ہیں۔۔ | 0 |
|  | 3052 | اس لڑکی کو جو بھی کہئے کم ہے | 0 |
|  | 1138 | جب تک تیرا منہ بند نہیں ہوگا حالات ایسے ہی رہینگے | 1 |
|  | 999 | تم کراچی میں آکر کیوں پلتے ہو اپنی جھیل کے ساتھ اپنے دیہات میں جاکر دفن ہو۔۔ دفعہ دور | 0 |
|  | 1166 | بھونک کتے کی طرح حرامزادے | 0 |
|  | 1968 | بےشک یہ بےغیرتوں کا خاندان ھے | 1 |
|  | 2514 | پاک فوج اور عوام ہمیشہ ساتھ ساتھ ہیں اسی بات سے دشمن کی راتوں کی نیند حرام ہو چکی ہے۔ | 0 |
|  | 461 | دیکھیے ہندوستان کی وہ خطرناک ہتھیار جو دشمن کے چھکے چھڑا دیں گے جس کے چلنے کے بعد آرمی کو دم دبا کر بھاگنا پڑے گا | 0 |
|  | 3271 | بھارتی میڈیا نے پاکستانی راکٹ حملوں میں تباہ شدہ بھارتی ٹینکرز اور چوکیوں کی تصویریں جاری کر دی | 0 |
|  | 294 | ہم زندہ بندے کو روٹی نہیں دیتے | 0 |
|  | 3126 | لعنت اللہ تیرے جمن تے گٹر کی پیداوار | 1 |
|  | 2713 | یہ رنڈی پاکستان وآپس آئے تو عوام کو چاہیئے جہاں بھی نظر آئے اس کو پکڑ کہ اس رنڈی کی گانڈ کا کیڑا مارے | 0 |
|  | 2585 | کھاتے کراچی کا ہو بھوکتے زرداری کی طرح او نمک حرام بنارسی ٹھگ | 1 |
|  | 687 | ابے جاہل انسان قبر میں تیری ٹانگیں ہیں ، پھر بھی خباثت سے باز نہیں آ رہا | 1 |
|  | 2550 | کبھی اس ڈرپوک بھگوڑے کمانڈو کا بھی پوچھ لیں جو جیل کے خوف سے فرار ھو گیا تھا۔ | 1 |
|  | 2658 | سؤر کی اولاد نے اپنی ماں اور بہن ن کو دی ہوئی کسی دگڑ دلی ماں کے دگڑ دلے بچے کی اُس گندگی کا نتیجہ ہے | 1 |
|  | 765 | یہ دھرنے کی پیداوار کیا جانیں کہ رشتے کیا ہوتے ہیں | 0 |
|  | 2614 | یہ ہمارے سات س… | 0 |
|  | 285 | کسی نے بیڑہ تو اٹھایا کام کرنے کا پیسے کے ساتھ ہی سہی ہو تو جائے گا۔ حکومت سندھ کو تو کوئی پھوٹی کوڑی | 1 |
|  | 1112 | پیشاب پینے والوں اپنی بکواس بند رکھو نہیں تو ہم پاکستانی تمہاری بکواس کے ساتھ ساتھ تمہارہ گانڈ بھی بند کر دیں گے | 1 |
|  | 888 | پنجاب ہوتا تو کو کہہ دیتے مگر… | 0 |
|  | 1939 | کشمیر پر قبضہ ہو گیا اور تم ادھر کیا کر رہے ہو ۔ | 0 |
|  | 1181 | بہن چود حرامی وارث میر کے ناپاک نطفے تجھے چوری نظر نہیں آتی نوابشاہ کی رنڈی نظر آتی ہے۔ | 1 |
|  | 116 | پہلوں کو ملا ہے جو اس بچے کو انصاف ملے گا ؟ نقیب اللہ، طاہر داوڑ کا خاندان، وہ ساہیوال والے تین بچے، اور نجانے کتن… | 0 |
|  | 2457 | جا او کتے بچے سور کی اولاد گانڈو بہن چود | 1 |
|  | 1815 | یا اللہ ٹویٹر پر ہمارے پیارے نبی ﷺ کی شان میں گستاخیاں کرنے والوں کو دنیا اور آخرت دونوں میں عبرت کا… | 0 |
|  | 2738 | کتے کبھی شیروں کا مقابلہ نہیں کرتے یہ بات اپنے دماغ میں بٹھا لینا | 1 |
|  | 250 | اگر یہ لاٹھی آپ جیسے کنجروں پہ نہیں چلی تو انڈیا پہ بھی نھیں چلے گی | 1 |
|  | 1843 | تبدیلی سرکار کا انتہائی افسوس ناک کارنامہ۔۔۔ تاریخ میں پہلی مرتبہ پی ٹی وی پہ خطبہ حج نہیں دکھایا | 0 |
|  | 1302 | بکواس کرنے سے باز نہیں آنا۔ناسور | 1 |
|  | 10 | یہ تو اس وقت سوچنا چاہییے تھا جب عوام کو دونوں ہاتھوں سے لوٹا، عمران خان کو بیچ میں کیوں گھیٹ رہے ہو | 0 |
|  | 2066 | گانڈ لگنی پٹنے پرساد لگی بٹنے | 1 |
|  | 2798 | آپ کو بتانا یہ تھا کہ کا بھٹو یا حکومت سندھ سے کوئی تعلق نہیں | 0 |
|  | 1661 | سچ کہتے ہیں کنجروں کی اپنی عزت تو ہوتی نہیں اور دوسروں کی اچھالتے رہتے ہیں حرام کی اولاد دنیا والوں کے لئیے عذاب ھے | 1 |
|  | 1383 | اس مادرچود نے تو اپنی ماں کو بھی چیلنج کر دیا تھا کہ ثابت کرو تمہارا خاوند ہہی مرا باپ ہے | 1 |
|  | 945 | تیری اطلاع کے مطابق قائد کے بعد کسی بہن چود سیاست دان نے بنگال کی طرف توجہ ہی نہیں دی جس… | 1 |
|  | 383 | نام ہے نورانی لیکن ہے مادرچود لعنتی | 1 |
|  | 2263 | یوتھیئے پہلے بدتمیز بنے، فیر کمینے، فیر بدمعاش۔۔۔ ہن پاگل ای ہو گئے نے | 1 |
|  | 5 | تو پھر ایک جرنل کے تین سال پر اتنی بکواس کیوں؟ | 0 |
|  | 2437 | یہ پیپلز پارٹی بیغیرتوں کیمینوں بے شرم اور بے حیاوں کا ٹولا ہے | 1 |
|  | 605 | کپتان کے لاکھ گھر بنانے پر اعتراض نہیں لیکن پہلے وزیرستان کے تباہ شدہ گھر بنانے ہوں گے۔ ہم اس بات کی اجازت نہیں… | 0 |
|  | 2129 | ایک یورپی ملک میں رہنے والے بندے نے میری جنجوعہ کے ساتھ پھڈے پہ مجھے بلاک انبلاک کر دیا تھا ۔۔ہن اوہ جنجوعہ دے ۔۔SS لا کر شکایتیں لگا رہا ھے | 0 |
|  | 1185 | جی اوئے گشتی کے بچے، مریم کے کتے،بلو رانی کے کتے | 1 |
|  | 1831 | صاحب کا لیڈر اج کل مریم نواز کی زبان بولنے لگا ہے | 0 |
|  | 997 | غازی بھائی کیوں بیغرت صحافیوں کو غیرت دلا کے ان کے لفافوں پر لات مار رہے ہیں ۔ | 1 |
|  | 1002 | بیٹا تو یہ اپنی بکواس اپنے پاس | 0 |
|  | 1568 | گورایا تیری ماں کو لن دینے کے لیے مجھے موقع ملا تو میں ضرور دوں گا کتی کے پتر تیری گانڈ میں نہ گولی ماری تو | 0 |
|  | 19 | سال سے کہاں مرا ہوا تھا یہ CM صاھب ؟ ابھی برسات کے مینڈک کی طرح نکل اپنی ویڈیو بندوانے لگے بھن چود | 1 |
|  | 3361 | مودی وحشی درندے تم انسان کے نام پر ایک حیوان ہوں جس کا دل پتھر کا ہے | 0 |
|  | 1235 | وہ پاکستانی یوٹیوبرز جنکے بکواس سارا سال ہمیں سننا پڑتا ہے،لیکن جب پاکستان پہ کوئی کڑا وقت آتا ہے تو انکو انڈیا کے… | 0 |
|  | 1749 | مودی کے دوست نے طوطا پالا، مودی جب بھی دوست کےگھر جاتا طوطا چلانے لگ جاتا او حرامزادہ بیغیرت آ گیا۔ | 1 |
|  | 2753 | پہلے چہروں کی سرجری کروائی جاتی تھی اب بھی کروائی جاتی ہے | 0 |
|  | 1699 | دن والے دھرنے کے دوران بھی شدید بارشوں سے پشاور خیبر پختونخواہ میں کتنے افراد اپنی جان سے ہاتھ دھو بیٹھے | 0 |
|  | 1404 | عزت پے سودا کیا ھر انسان تیری طرح دلا نہیں | 1 |
|  | 1116 | اس بہن چود کی گانڈ میں بھی ڈنڈا دو | 1 |
|  | 1376 | گشتی کے بچے یہ بتاؤ | 1 |
|  | 1509 | بہت ہی بےغیرت انسان یو تم تمہارے ڈاکو نواز نیں پاکستان بیچ دیا اور تم چپ رہے یہ عمران ہے جو کشمیر… | 1 |
|  | 2941 | تم شام وسحر یہ… | 0 |
|  | 1458 | عید منانی ہے یا چدائ کا کام کروانا ہے جو میاں اور بھگوڑی کے نہُہونےُسےُاداس ہو؟ بہت ہی غلط نطفہ ہے تمہارا کھسری۔ | 1 |
|  | 566 | تم جیسے کنجروں کی وجہ سے ھوا ھے قبر والے لوٹے | 1 |
|  | 2220 | چل جھوٹے لعنتی بکواس کر رہے ہو یہ کراچی ہے اور یہ رینجر اہلکار ہے | 1 |
|  | 1227 | بھارت کا سیکولر چہرہ بے نقاب۔ | 0 |
|  | 506 | تیری اوقات رنڈی عورت جتنی بھی نہیں گشتی سفینا گشتی عورت کو الٹا لٹکاو | 1 |
|  | 1119 | یہ ہے RSS کی وہ غلیظ ترین رنڈی عورت جس نے کہا ہے کہ ہندو لڑکے Groups بنا کر مسلمانوں کے گھروں پر حملہ کریں | 1 |
|  | 981 | لعنتی نشی کتا بیغرت سلیکٹڈ زانی کنجھر خبیث ذلیل منافق جھوٹا قادیانی دلا حرامی پالتو ثور | 1 |
|  | 2356 | بکواس بند کر حرامی کتے | 0 |
|  | 2536 | یہ قبروں کی کمائی کھانے والے پیرنیوں فقیروں تعویز دھاگوں جادو ٹونے کرنے والے جو شرک کے گنہگار ھیں انکو عبرت ناک سزا دینی چاہیے | 0 |
|  | 2711 | بس یہی بکواس ہے خود کوئی برا نہیں بنتا بس دوسروں پر ڈال دینی ہے ذمہ داری اپنی ڈیوٹی کوئی نہیں | 0 |
|  | 886 | ملک دہشت گرد سٹیٹ بن چکا ہے | 0 |
|  | 1229 | سور کا بچہ | 1 |
|  | 2418 | جب کوئ پاک فوج پر بکواس کرتا یے تو ان شہیدوں کے لواحقین پر کیا گزرتی ہوگی ۔۔۔۔۔ عقل اور دل کے اندھوں کو شاید اس تصو… | 0 |
|  | 701 | ڈیل پوئ کہ ارسطو کی گانڈ مودی مارے گا جبکہ مریم یم یم روایتی طور پر قطری کو پیش کی جائے گی | 1 |
|  | 2844 | یہ جو ملک بیچنے کی بکواس کر رہے ہیں بندا ان سے پوچھے | 0 |
|  | 2127 | یہ لڑکا اس حالت میں ہمارے معاشرے کا عکس ہے۔ ہمارے معاشرے کی کمر پر بھی پینٹ نہیں ہے | 0 |
|  | 974 | بد بخت، کمینے، گھٹیا، کس ذلیل گھر میں پلے بڑھے ہو | 0 |
|  | 3398 | پاک فوج کی جوابی کارروائی میں بھارتی فوج کے بنکرز بھی تباہ،آئی ایس پی آر | 0 |
|  | 489 | نے ہندوستان کے اسلحہ ڈپو کی تھوڑی دیر پہلے گانڈ مار دی اب پاکستانی توپیں تھوڑی تھوڑی دیر کے بعد ان ہندوؤں کی پورا سال انتظار کر کے ایک رات میں پورے سیزن کی گانڈ مار دی | 1 |
|  | 642 | میرے فالور میں سب سے زیادہ پی ٹی آٸی والے شکر کوٸی ن لیگی نہیں کیونکہ ن لیگ والے میری فوج خلاف بکواس کرتے ہیں جب پی… | 0 |
|  | 321 | ایسے نہیں ایسے سن زراہ غور سے حرامی | 0 |
|  | 837 | دنیا کا سب سے گھٹیا دھرم ہندوؤں کا ہے | 1 |
|  | 3088 | ھاھاھا پاکستانی لڑکیاں پاپا کی گڑیا جو بن جاتی ہیں توبہ ایسے کوئی نخرے ہو جاتے ہیں | 0 |
|  | 2816 | کس قدر عجیب صورت حال ہے جو بھی بھارت کے مظالم کے خلاف آ واز بلند کرتا ہے اسی کا اکاؤنٹ سسپنڈ کر دیا جاتا ہے افسوس ناک صورت حال ہے یہ تو۔ | 0 |
|  | 3286 | ہٹلر نے ظلم کیا عبرت کا نشان بن گیا ظلم کی ابتدا ء خوف اور انتہا عبرتناک ہوتی ہے | 0 |
|  | 1650 | جن لوگوں کو کھال اتارنے میں دقت پیش آرہی ہو وہ اپنی بیویوں سے رجوع کرسکتے | 0 |
|  | 879 | دنیاپور ٹریفک حادثے میں جانی نقصان | 0 |
|  | 736 | جو دماغ پر راج کرے اسے مار خور کہتے ہیں جو دل پر راج کرے اسے آئی ایس پی آر کہتےہیں جو بھارت کی نیند حرام کر دے اسے… | 0 |
|  | 1503 | وہ فیملی پر حملہ کرتا ہے وہ ہیجروں کی طرح ہوتا ہے | 0 |
|  | 2951 | تیری گانڈ جس جس نے ماری ہے اس کو تمغا ملے گا شکل سے ہی گانڈو لگتا ہے بغیرت | 1 |
|  | 1937 | بہن چود چوتیا انسان غداری کرنے کے بہت طریقے اتے تجہے | 1 |
|  | 2341 | سوور کی بچی پین چود گشتی | 1 |
|  | 1905 | لعنتیوں | 1 |
|  | 577 | یہ بلڈی سویلین کیا بکواس کر رہے ہیں | 0 |
|  | 2958 | یہ بہن چود پیسے کے دم پر خدا بنے بیٹھے ہیں | 1 |
|  | 3279 | تجھے چود نا دونگا، ماں کو لن لینے | 1 |
|  | 933 | اوقات انڈین کی | 0 |
|  | 2522 | یار افغانیوں کی ماں بہن کی چدائی کرنے کا بہت مزہ آتا ہے بہت سفید ہوتی ہیں | 1 |
|  | 612 | یہ نمک حرام فضل الرحمن٫ محمود اچگزئی او بزنجو کہاں غرق ہیں؟ کوئی اس نمک حرام مفرور حرام کی نسل کو پوچھے دنیا میں تیرےجیسے بھڑوے بھی ہیں کیا جو خود بھاگ گئے ہیں | 0 |
|  | 2529 | بہن چود لبرل یہی سب کچھ پاکستان میں چاہتے ہیں | 1 |
|  | 1980 | تمھارا گزشتہ کل ہاتھ سے نکل گیا اور آنے والےکل کا کوئی اعتبار نہیں | 0 |
|  | 2455 | نا بھوک لگ رہی نا پیاس دل الگ پریشان ہے خواب بھی عجیب آ رہے نا خوشی محسوس ہوتی ہے نا غم بس بکواس ترین دن ہیں | 0 |
|  | 1917 | فٹے منہ ایسی دلالی پر ایسے ہیرو پر اور ایسے باپ پر جو اپنی بیٹی کو گوروں کہ ہاتھ دے آۓ | 0 |
|  | 2192 | مادرچود ہم کشمیریوں کا نام مت لے | 1 |
|  | 2701 | اور تیرے جیسے بے غیرت کو بھڑوا کہا جائے ۔۔۔ بہن چود شرم نہیں آتی چوروں کو ڈیفنڈ کرتے ہوئے ، بے حیا انسان | 1 |
|  | 1313 | تو بھی بچے زبیر بلوچ کے کھانے اڑاتا رھا ھے، کنجروں کے ساتھیو نمبر نہ بنایا کرو | 1 |
|  | 3293 | چھترول جیالوں کی ھوتی ہے تکلیف پٹواریوں کو ھوتی ہے یہ کونسی ہیر رانجھا شروع کی اِن کنجروں نے | 1 |
|  | 1016 | پیدل معصوم بچے کو بچانے کی وجہ سے حادثہ، کتنے جان سے ہاتھ دھو بیٹھے؟ جانیے | 0 |
|  | 671 | شیریں مزاری بھی پریانکا چوپڑا کیخلاف میدان میں آ گئیں | 0 |
|  | 2579 | پاکستان تحریک انصاف میں جو انصاف چھپ کے بیٹھا ھے لولہ لنگڑا | 0 |
|  | 3401 | مشرف دور میں جب کراچی میں MQMکا سکہ چلتا تھا تو وہ لوگ بھی یہی بات کرتے تھے قربانی اللہ کے لیے اور کھال MQMکو دینی… | 0 |
|  | 2394 | اور وہ بھارت کی شکل میں ہوگا۔ جب بھارت کو عبرت کا نشان بنا دیا… | 0 |
|  | 3072 | وقاص علوی کسی گشتی کی حرامی نسل سے ہے کتے کے پلے تیری ماں کو پتہ نہیں ہوگا تیرا باپ کون تھا | 1 |
|  | 452 | نطفہ حرام کے کسی چلتی پھرتی آوارہ کھوتی کے بچے کیا ثبوت ہیں تیرے پاس رنڈی کے بچے ایسے فضول بکواس مادر چود کمینے | 0 |
|  | 1331 | بے شرم انسان کشمیر کے لوگ بھی مر رہے ہے منہ کھولنا ہےتو ادھر کھول بہن چود بھڑوا گانڈو | 1 |
|  | 1491 | پاکستانی فوج نے آج پہل کی اور بھارتی فوجیوں سمیت ایک افسر کو جہنم واصل کیا۔ دو بنکر مکمل تباہ کیے۔ | 0 |
|  | 2087 | یہ پیارا پرچم عزیم پرچم جیسی کچھ ضمیر فروش جھکانا چاھتے ھے | 0 |
|  | 3407 | اب جاؤ الٹا لٹک کے کر چیلا کاٹو درباری تم صرف بدعوں سے کام چلاؤ گے بھگوڑے۔ | 1 |
|  | 2445 | تم افغانیوں کی گاںڈ پر لات مار کر افغانستان بھیجنے کا وقت ہوا جاتا ہے | 0 |
|  | 912 | کتے کے بچے کتنے پیسے ملے اپنے باپ سے تجھے | 1 |
|  | 1605 | حامد میر غدار ہے اس کو فوری سزا دی جائے تاکہ باقی غداروں کےلئے عبرت کا نشان بن جائے | 0 |
|  | 403 | بے غیرت ہجڑا اور اس کا باپ | 1 |
|  | 2860 | اس کو بھی پتہ ہوتا سننے والے گدھے ہیں جو مرضی بکواس کر لو انہوں نے یہی کہنا شیر | 0 |
|  | 3476 | کچھ دن سے کےخلاف سوشل میڈیا پر ان کےبیٹےکی ہندو لڑکی سے شادی کو لےکر ملیحہ لودھی کی حب الوطنی پر سوال… | 0 |
|  | 1087 | لعنت تیرے منہ پر بہن چود نامرد کشمیر کے مظلوم عوام پر ظلم کرتے ہو | 1 |
|  | 1713 | ابي چل مادرچود | 1 |
|  | 3193 | حرام پے پلے ھوے حرامی | 1 |
|  | 2799 | ہندوستان آج بھی ابوالکلام آزاد جیسے غلام ذہنیت مسلمانوں سے بھرا پڑا | 1 |
|  | 3114 | کبھی کبھی ایکسٹنشن ٹینشن بھی دے جاتی ہے اور یہ سسٹم… | 0 |
|  | 1089 | کشمیر بنے گا پاکستان | 0 |
|  | 1418 | بہت ہی پُر مزمت اور افسوس ناک واقع ہے یہ اللہ اس ملک پر رحم کرے ۔ اور گدے کی حکمرانی سے نجات ملے ، آمین | 0 |
|  | 2764 | ہاہاہا میرا سالہ اپنی باجی کی پدی صاف کر رہا ہے آج تیرا بہنوئی آ رہا ہے تیری گانڈ بھی مارے گا | 1 |
|  | 1332 | مادر چود کشمیر بیچنے والوں کے بھڑوے بھروے عوام کو پاگل سمجھ رکھا ہے کتے کے بچے کشمیر بیچ کر اب اس بکواس کا مطلب | 1 |
|  | 2154 | ایک لفظ نہیں بہت سارے ہیں مثلاً کُتا، بے غیرت، زانی، کنجر،زکوتہ چور، دلا، بہن چُود، سُور | 1 |
|  | 464 | یہ دلّا آدمی رقص کر کر کے آگے کھڑی حجیانیوں کو پتھر مار رہا ہے | 1 |
|  | 1300 | انڈیا کشمیر میں بچوں تک کو مار رہا ہے اور کُچھ بیغیرتوں کو جانوروں کی قربانی پر درد اُٹھ رہا | 0 |

 Go

## Footer

© 2022 GitHub, Inc.

### Footer navigation

- Terms
- Privacy
- Security
- Status
- Docs
- Contact GitHub
- Pricing
- API
- Training
- Blog
- About

You can’t perform that action at this time.

You signed in with another tab or window. Reload to refresh your session.
You signed out in another tab or window. Reload to refresh your session.
