# Supplementary material for: Detection of offensive terms in resource-poor language using machine learning algorithms
Source: PeerJ Comput Sci. 2023 Aug 29;9:e1524. doi: 10.7717/peerj-cs.1524 (PMC10496005; doi:10.7717/peerj-cs.1524)
Supplement: Supplemental Information 2 — This dataset is gathered from Youtube comments. It contains 2 columns, one column contains the comment in Urdu and target column that contains 0 and 1 to represent if the comment is abusive or not. In Experimental section results dataset 2 is used in scenario 1 as test dataset and in scenario 2 as training dataset. [file peerj-cs-09-1524-s002.htm]

Resource-Poor-Language-Urdu-Dataset/Dataset 2.csv at main · owais4321/Resource-Poor-Language-Urdu-Dataset · GitHub


Skip to content


Toggle navigation

Sign up

- Product

  - Actions

    Automate any workflow
  - Packages

    Host and manage packages
  - Security

    Find and fix vulnerabilities
  - Codespaces

    Instant dev environments
  - Copilot

    Write better code with AI
  - Code review

    Manage code changes
  - Issues

    Plan and track work
  - Discussions

    Collaborate outside of code
  - Explore
  - All features
  - Documentation
  - GitHub Skills
  - Blog
- Solutions

  - For
  - Enterprise
  - Teams
  - Startups
  - Education
  - By Solution
  - CI/CD & Automation
  - DevOps
  - DevSecOps
  - Case Studies
  - Customer Stories
  - Resources
- Open Source

  - GitHub Sponsors

    Fund open source developers
  - The ReadME Project

    GitHub community articles
  - Repositories
  - Topics
  - Trending
  - Collections
- Pricing

- In this repository

  All GitHub
  ↵

  Jump to
  ↵

- No suggested jump to results

- In this repository

  All GitHub
  ↵

  Jump to
  ↵
- In this user

  All GitHub
  ↵

  Jump to
  ↵
- In this repository

  All GitHub
  ↵

  Jump to
  ↵

Sign in

Sign up

{{ message }}

owais4321
/
**Resource-Poor-Language-Urdu-Dataset**
Public

- Notifications
- Fork
  0
- Star
   0

- Code
- Issues
  0
- Pull requests
  0
- Actions
- Projects
  0
- Security
- Insights

More


- Code
- Issues
- Pull requests
- Actions
- Projects
- Security
- Insights

Permalink

main

Switch branches/tags


Branches
Tags

Could not load branches


Nothing to show

{{ refName }}
default
View all branches

Could not load tags


Nothing to show


{{ refName }}
default
View all tags

# Name already in use

A tag already exists with the provided branch name. Many Git commands accept both tag and branch names, so creating this branch may cause unexpected behavior. Are you sure you want to create this branch?

 Cancel
 Create

## Resource-Poor-Language-Urdu-Dataset/**Dataset 2.csv**

 Go to file

 

- Go to file
  T
- Go to line
  L
- Copy path
- Copy permalink

This commit does not belong to any branch on this repository, and may belong to a fork outside of the repository.

Cannot retrieve contributors at this time

2171 lines (2171 sloc)
213 KB

Raw
  Blame

Edit this file

E


Open in GitHub Desktop

- Open with Desktop
- View raw
- Copy raw contents
   Copy raw contents

   Copy raw contents

   Copy raw contents
- View blame

This file contains bidirectional Unicode text that may be interpreted or compiled differently than what appears below. To review, open the file in an editor that reveals hidden Unicode characters.
Learn more about bidirectional Unicode characters

Show hidden characters


|  | tweet | target |  |  |  |  |  |  |  |  |  |  |
| --- | --- | --- | --- | --- | --- | --- | --- | --- | --- | --- | --- | --- |
|  | کہ کے لے لی شام دلے کی | 1 |  |  |  |  |  |  |  |  |  |  |
|  | اب اگر اس نے کچھ جواب دیا تو اس کی گانڈ مار دیں گے | 1 |  |  |  |  |  |  |  |  |  | 1109 |
|  | اب ان چوتیوں نے وہ جو کنسرٹ ہو رہا تھا وہ بھی کینسل کر دیا | 1 |  |  |  |  |  |  |  |  |  | 1060 |
|  | اب ان کی گانڈ میں ہاتھ ڈال کر انتڑیاں نکالے گا کیا | 1 |  |  |  |  |  |  |  |  |  |  |
|  | اب تک سکی سب سے اچھی ویڈیو ہے | 0 |  |  |  |  |  |  |  |  |  |  |
|  | اب تک کا سب سے برا مزاق | 0 |  |  |  |  |  |  |  |  |  |  |
|  | اب تو اتنی مار دی تیری بھائی نے کہ اب تو شادی والے جملے قابل نہیں رہا | 1 |  |  |  |  |  |  |  |  |  |  |
|  | اب لوڑا آگیا ہے یا للی کا ٹوپا | 1 |  |  |  |  |  |  |  |  |  |  |
|  | اب وہ اپنی گانڈ کا زور لگائے گا اس ویڈیو کو بند کروانےمیں | 1 |  |  |  |  |  |  |  |  |  |  |
|  | اب وہ چوتیا کو دکی بھائی بین کر دے گا | 1 |  |  |  |  |  |  |  |  |  |  |
|  | ابرار یہ سب ہندو کلچر میں نہیں ہے یہ سب بکواس ویڈیو بناتے ہیں یہ لوگ | 0 |  |  |  |  |  |  |  |  |  |  |
|  | ابلیس اوئے دلے نازک صورتحال فر ابیلس دلے | 1 |  |  |  |  |  |  |  |  |  |  |
|  | ابلیس ایک اچھا نام ہے | 0 |  |  |  |  |  |  |  |  |  |  |
|  | ابلیس کی نئی والی ویڈیو دیکھو اس نے اپنی گانڈ میں ڈنڈا دیا ہوا ہے | 1 |  |  |  |  |  |  |  |  |  |  |
|  | ابلیس کینیڈا میں جا کر اپنی اوقات بھول گیا ہے سالے کو اردو تو بولنی آتی نہیں شام گانڈو کو | 1 |  |  |  |  |  |  |  |  |  |  |
|  | ابلیس نام ہے اس بہن کے لوڑے کو | 1 |  |  |  |  |  |  |  |  |  |  |
|  | ابلیس ہے ہی چوتیا ہا ہا ہا حچاب | 1 |  |  |  |  |  |  |  |  |  |  |
|  | ابھی آپ نے یہ چوتیاپا تو کر لیا ویڈیو بنا کراور آپ کو بہت شہرت بھی ملے گی لیکن کیا ہوگا اگر یہ چوتیاپا کر کے تجھے اور کچھ نہ ملا تو | 1 |  |  |  |  |  |  |  |  |  |  |
|  | ابھی بھڑوے کے بچے تیل کی بوتل دے تو اپنی ماں کو دے گانڈو ویسے معزز حجاب اور آگے گالیاں | 1 |  |  |  |  |  |  |  |  |  |  |
|  | ابھی بھی اس رنڈی کے بچے کو صیح طرح سے بےعزت نہیں کیا دکی بھائی نے | 1 |  |  |  |  |  |  |  |  |  |  |
|  | ابھی تک اس کی گانڈ میں یہ پیسے چلے گئے ہوں گے | 1 |  |  |  |  |  |  |  |  |  |  |
|  | ابھی سبسکرائب کیا میں نے اس گانڈو کا پیج | 1 |  |  |  |  |  |  |  |  |  |  |
|  | ابھی میں نے پردیسی لڑکی کی ایک ویڈیو دیکھی ہے تیرے والی جو دنچک پوجا پر تھی | 0 |  |  |  |  |  |  |  |  |  |  |
|  | ابھیشیک اگر وقت ملے تو مرا چینل بھی دیکھ لینا | 0 |  |  |  |  |  |  |  |  |  |  |
|  | ابے اس کا بھی کچھ کر لے | 0 |  |  |  |  |  |  |  |  |  |  |
|  | ابے او جھوٹے ابلیس ماں کی چوت دفعہ ہو یہاں سے | 1 |  |  |  |  |  |  |  |  |  |  |
|  | ابے آپس کی بات ہے تو یہ چینل کیسے چلا رہا ہے | 0 |  |  |  |  |  |  |  |  |  |  |
|  | ابے بار کا لوڑا ہے تو تو نے بھی تو کوئی کام کی بات کی ہے تو بھی بار کا لوڑا ہے اور وہ بھی گانڈو ہے | 1 |  |  |  |  |  |  |  |  |  |  |
|  | ابے بہت اعلیٰ ویڈیو ہے ایسے ہی ویڈیو بناتے رہو | 0 |  |  |  |  |  |  |  |  |  |  |
|  | ابے بہن چود تو اس کے چینل کو بند کروانے کا کہ رہا ہے لعنت ہے تم پر | 1 |  |  |  |  |  |  |  |  |  |  |
|  | ابے بھوسڑی کے پہلے آئینہ میں جا کر اپنی شکل تو دیکھ چوتیا ٹٹی جیسی شکل ہے تیری | 1 |  |  |  |  |  |  |  |  |  |  |
|  | ابے بھوسڑی کے زیادہ شوق ہے بال ووڈ کا تو انڈیا ہی چلا جا | 1 |  |  |  |  |  |  |  |  |  |  |
|  | ابے تجھے ردعمل چاہیے شام اور فراگی کا تا کہ تو ابھی مشہور ہو جائے گانڈو تو بھی وہی حرکتیں کر رہا ہے بھڑوے | 1 |  |  |  |  |  |  |  |  |  |  |
|  | ابے تیری ماں کی چوت بھوسڑی کے تیری گانڈ پھاڑ دوں گا اگر کچھ کہا تو | 1 |  |  |  |  |  |  |  |  |  |  |
|  | ابے جاہل جانور میں تیری بات کر رہا ہوں تو اپنے چینل کے لیے ہر کسی کا لوڑا اپنی گانڈ میں لینا چھوڑ دے | 1 |  |  |  |  |  |  |  |  |  |  |
|  | ابے جلتے کیوں ہو ہاہاہا تو نے بھی تو اپنے چینل پر گالیاں بین کی ہوئی ہیں کتے جیسی تو شکل ہے تیری | 1 |  |  |  |  |  |  |  |  |  |  |
|  | ابے جھوٹے شام کے ٹٹے دفعہ ہو غرق ہو شام کی ماں کی چوت | 1 |  |  |  |  |  |  |  |  |  |  |
|  | ابے چوتیے تو اپنی ویڈیو کی طرف توجہ دے دو نمبر طریقے سے شہرت حاصل کرنے والا | 1 |  |  |  |  |  |  |  |  |  |  |
|  | ابے چوتیے کتنا آسان ہے نا کسی کی ویڈیو کو روسٹ کرنا اپنا کام دیکھ اور ان کا کام دیکھ صرف مائیک لے کر ٹٹی کرنا آاسان ہے | 1 |  |  |  |  |  |  |  |  |  |  |
|  | ابے چوتیے کے ڈھکن کیسے فالتو ویڈیو بناتا ہے | 1 |  |  |  |  |  |  |  |  |  |  |
|  | ابے چوتیے لعنت ہے تجھ پر | 1 |  |  |  |  |  |  |  |  |  |  |
|  | ابے چوتیےانہوں نے تیرا نام کہاں لیا ہے جو ہر بات اپنے اوپر فٹ کر رہا ہے | 1 |  |  |  |  |  |  |  |  |  |  |
|  | ابے دکی زیادہ چوتیاپا نہ کر اور بولنے سے پہلے سوچ لیا کر فراڈ ہو گا تیرا باپ فراڈ ہوتی تیری ماں | 1 |  |  |  |  |  |  |  |  |  |  |
|  | ابے سالے چوتیے تو اپنے آپ کو سمجھتا کیا ہے | 1 |  |  |  |  |  |  |  |  |  |  |
|  | ابے شکل تو تیری بھی کسی بندر سے کم نہیں ہے میں کوئی شام کا فائن نہیں ہوں | 1 |  |  |  |  |  |  |  |  |  |  |
|  | ابے کالے ابھی تک ویڈیو اپ لوڈ کیوں نہیں کیں | 1 |  |  |  |  |  |  |  |  |  |  |
|  | ابے کمینے تو سالے چوتیا ہے پوری بات تو پڑھ کہ کس کے لیے لکھا ہے | 1 |  |  |  |  |  |  |  |  |  |  |
|  | ابے کیری آج سے پہلے کبھی تو نے سستے نشے کیئے ہیں | 0 |  |  |  |  |  |  |  |  |  |  |
|  | ابے کیری تو پکانا بند کر اب تو بور کرنے لگا ہے تو | 0 |  |  |  |  |  |  |  |  |  |  |
|  | ابے گانڈ سے پاگل بھوسڑے کی تیری گانڈ جلتی ہے وہ وہاں بیٹھ کر پاکستان کا سب سے بڑا یوٹیوبر ہے | 1 |  |  |  |  |  |  |  |  |  |  |
|  | ابے لوڑو تو ہے کون چوتیا فضول میں بھونک رہا ہے تجھے کہ کون رہا ہے کچھ کرنے کو | 1 |  |  |  |  |  |  |  |  |  |  |
|  | ابے لونڈے میں دم نہں ہے دیکھ لے | 1 |  |  |  |  |  |  |  |  |  |  |
|  | ابے مادرچود لنڈ تو لینے آیا ہے یہاں پر وہ بھی اپنی بہن کے لیے | 1 |  |  |  |  |  |  |  |  |  |  |
|  | ابے یہ نعمان کون ہے اور کدہر ہے | 0 |  |  |  |  |  |  |  |  |  |  |
|  | اپنا سپر سٹار کیری | 0 |  |  |  |  |  |  |  |  |  |  |
|  | اپنا کاپی کیا ہوا چینل دیکھ لے اور اپنی ویڈیوز بھی | 0 |  |  |  |  |  |  |  |  |  |  |
|  | اپنا وقت ان جیسے جوتیوں لوگوں پہ مت لگاؤ اپنا کام بہتر کرو اور ان سے نمبر لے جاؤ | 1 |  |  |  |  |  |  |  |  |  |  |
|  | اپنا ویڈیو کا ویو اور گانے کا ویو دیکھ گانڈو زمین اور آسمان کا فرق ہے اپنی اوقات دیکھ اور اس کی اوقات دیکھ کر بات کر | 1 |  |  |  |  |  |  |  |  |  |  |
|  | اپنی پسندیدہ ویڈیو دیکھیں | 0 |  |  |  |  |  |  |  |  |  |  |
|  | اپنی ماں کو تو بچالے بہن کے لوڑے تیری ماں کو چود گیا | 1 |  |  |  |  |  |  |  |  |  |  |
|  | اپنی ماں کو دے جا کر مادر چود پھر بھی نیہں ملا تھا جو ادھر آگیا لینے | 1 |  |  |  |  |  |  |  |  |  |  |
|  | اپنی مرضی سے سنیں ویڈیو کا میوزک شروع ہو گیا | 0 |  |  |  |  |  |  |  |  |  |  |
|  | اتار کے رکھ دی بہن چود کی | 1 |  |  |  |  |  |  |  |  |  |  |
|  | اترو سن کے وہ گوبھی والی آنٹی مر گئی ہوں گی بہت اچھا بھائی | 0 |  |  |  |  |  |  |  |  |  |  |
|  | اتنا اچھا ویڈیو کیری نے پہلی بار ڈالا ہے | 0 |  |  |  |  |  |  |  |  |  |  |
|  | اتنا پسند نہیں آیا اور چھوٹا بھی تھا ان کے کونٹینٹ سے ہمارا کونٹینٹ جدا ہونا چاہیے | 0 |  |  |  |  |  |  |  |  |  |  |
|  | اتنا مزہ نہیں آیا | 0 |  |  |  |  |  |  |  |  |  |  |
|  | اتنی گندی تو انعم کی بھی نہں ہوئی جتنی شام کی ہو گئی پر جو بھی کہا دکی بھائی نے صیح کہا | 1 |  |  |  |  |  |  |  |  |  |  |
|  | اتنی گھٹیا ویڈیو زندگی میں پہلی بار دیکھا ہے | 0 |  |  |  |  |  |  |  |  |  |  |
|  | اتنی محنت وہ بھی گانڈو کے لیے جو پہلے سے گانڈو ثابت ہو گیا | 1 |  |  |  |  |  |  |  |  |  |  |
|  | اتنی مزاحیہ نہیں تھا لیکن کام اچھا تھا | 0 |  |  |  |  |  |  |  |  |  |  |
|  | اج گیم نہیں کھیلا کیا جو کارٹون لگا دئیے غصہ دلا دیا | 0 |  |  |  |  |  |  |  |  |  |  |
|  | اجے بہت چوتیا ہے | 1 |  |  |  |  |  |  |  |  |  |  |
|  | اجے بھائی اب لاکھوں ہوں جائیں گے کیری بھائی کے لئیے لائیکس | 0 |  |  |  |  |  |  |  |  |  |  |
|  | اجے بھائی پردیسی لڑکی نےآپ کی ویڈیو پر ردعمل دیا ہے تو آپ اس کے ردعمل پر ویڈیو بنائیں | 0 |  |  |  |  |  |  |  |  |  |  |
|  | اجے تمہارے کمنٹ نےمیری ویڈیو سے زیادہ لائیکس حاصل کیے ہیں | 0 |  |  |  |  |  |  |  |  |  |  |
|  | اجے چوتیا | 1 |  |  |  |  |  |  |  |  |  |  |
|  | اجے کا دوسرا نام لنڈ ہے | 1 |  |  |  |  |  |  |  |  |  |  |
|  | اجے گانڈو ہے | 1 |  |  |  |  |  |  |  |  |  |  |
|  | اجے مین مجھے آپ کے دانتوں سے پیار ہو گیا ہے | 0 |  |  |  |  |  |  |  |  |  |  |
|  | اجےمجھے آپ کی آواز آپ کا سٹائل بہت اچھا ہے مجھے آپ بہت پسند ہیں | 0 |  |  |  |  |  |  |  |  |  |  |
|  | اچھا بھائی یہ تو چلتے ہیں | 0 |  |  |  |  |  |  |  |  |  |  |
|  | اچھا دکی بھائی شام چوتیا ہے | 1 |  |  |  |  |  |  |  |  |  |  |
|  | اچھا کیا بھائی گانڈ میں واقعی ڈنڈا دے دیا آپ نے ان کو | 1 |  |  |  |  |  |  |  |  |  |  |
|  | اچھا کیا یار تم نے صیح اوقات یاد دلائی تم نے اس بہن چود کو سالے | 1 |  |  |  |  |  |  |  |  |  |  |
|  | اچھاہے چوتیا ہے عرفان جنجوے کبھی ایسا نہں سنا | 1 |  |  |  |  |  |  |  |  |  |  |
|  | اچھی گانڈ ماری آپ نے اسکی اپنے لوڑے سے | 1 |  |  |  |  |  |  |  |  |  |  |
|  | اچھی ویڈیو تھی اگر کوئی گوگل سے پیسے کمانا چاہتا ہے تو میرے چینل کو دیکھیں | 0 |  |  |  |  |  |  |  |  |  |  |
|  | اختتام ضرور بہت اچھا ہوگا | 0 |  |  |  |  |  |  |  |  |  |  |
|  | اخیر حوصلہ ہے تیرا تو چیتا ہے ماور اس بھوسڑی کے کی گانڈ | 1 |  |  |  |  |  |  |  |  |  |  |
|  | اخیر کردی ہے اس دلے نےجھوٹی ویڈیوز بنا بنا کر میں گانڈ مار دوں گا اب ان کی | 1 |  |  |  |  |  |  |  |  |  |  |
|  | اخیر ہوگئی صیح بجائی اس دلے کی بھائی نے | 1 |  |  |  |  |  |  |  |  |  |  |
|  | ادریس چوتیا ہے | 1 |  |  |  |  |  |  |  |  |  |  |
|  | ادھار کارڈ کو لنک کرو ہاہاہاہا | 0 |  |  |  |  |  |  |  |  |  |  |
|  | ارفع عظمت شام بھوسڑی کا ہے | 1 |  |  |  |  |  |  |  |  |  |  |
|  | ارے اجے بھیا آپ سے مل کر آئے اس کا مطلب کہ ہم بھی ان سے مل گئے | 0 |  |  |  |  |  |  |  |  |  |  |
|  | ارے بھائی ارے بھائی بہت اچھا آگے بڑھو چوتیا تو بنا نہں ہو گا | 1 |  |  |  |  |  |  |  |  |  |  |
|  | ارے بھائی ایک گروپ میں ہی سب لوگ ہوتے ہیں ابھی تک بہت اچھا ہے | 0 |  |  |  |  |  |  |  |  |  |  |
|  | ارے بھائی بھائی بھائی آخری سپر لائیک | 0 |  |  |  |  |  |  |  |  |  |  |
|  | ارے بھائی تم پاگل ہو میں اپنی ہنسی نہیں روک پا رہا | 0 |  |  |  |  |  |  |  |  |  |  |
|  | ارے بھائی کہاں ہے یار اگلی ویڈیو کب آرہی ہے | 0 |  |  |  |  |  |  |  |  |  |  |
|  | ارے دکی بھائی لعنت بھیج اس چوتیا پر | 1 |  |  |  |  |  |  |  |  |  |  |
|  | ارے وہ کالی چرن کو ولن کا کردار ادا کرنا چاہیے تھا | 0 |  |  |  |  |  |  |  |  |  |  |
|  | ارے یار اپنی دل کی بات چھین لی قسم سے بہت گالیاں دی تھیں میں نے اس گانڈو کو بعد میں اس نے مجھے ڈیلیٹ کر دیا | 1 |  |  |  |  |  |  |  |  |  |  |
|  | ارے یار بہت بہن چودی کرتے ہو یار کیا بندے ہو تم | 1 |  |  |  |  |  |  |  |  |  |  |
|  | اس بار تو آپ نے اس جھوٹے کی گانڈ بند کر دی اس وقت کہتا تھا کہ پاکستان آؤں گا اب بھوسڑی کا کہتا ہے کہ پاکستان نہں آئے گا | 1 |  |  |  |  |  |  |  |  |  |  |
|  | اس بار کچھ کام زیادہ ہی خراب ہو گیا لاکھوں لوگوں نے مجھے ناپسند کیا ہے | 0 |  |  |  |  |  |  |  |  |  |  |
|  | اس بہن چود کی نسل کو ننگا کر کے مارنا چاہیے | 1 |  |  |  |  |  |  |  |  |  |  |
|  | اس بہن یکی کو سپورٹ کرتے ہو تم بہت ہی چوتیا ہو | 1 |  |  |  |  |  |  |  |  |  |  |
|  | اس بھڑوے ادریس کو اور گندا کرنا تھا | 1 |  |  |  |  |  |  |  |  |  |  |
|  | اس بھوسڑی والے کو پکڑ کر اس کی گانڈ میں ڈنڈا مارو اور فراگی کی گانڈ الگ سے مارنا | 1 |  |  |  |  |  |  |  |  |  |  |
|  | اس بے غیرت کو کیا حق بنتا ہے کہ وہ ہم پاکستانیوں کو کچھ کیے | 1 |  |  |  |  |  |  |  |  |  |  |
|  | اس پاگل انسان کو اس کے گھر والے کیسے جھیل رہے ہوں گے ان کی ہمت کی داد دیتا ہوں | 0 |  |  |  |  |  |  |  |  |  |  |
|  | اس پر ویڈیو بنا دے بھائی بہت تکلیف میں ہے بھائی یہ بندہ | 0 |  |  |  |  |  |  |  |  |  |  |
|  | اس جھوٹی ویڈیو دیکھنے سے بہتر تھا کہ سدھارت کی گانڈ مار لیتے | 1 |  |  |  |  |  |  |  |  |  |  |
|  | اس جیسے چوتیا لوگ ہی ہوتے ہیں جو پاکستان اور اسلام کی بے عزتی کرواتے ہیں | 1 |  |  |  |  |  |  |  |  |  |  |
|  | اس چوتیا بے غیرت حرامی کتے شام کو کتنی جوتیاں پڑنی چاہیں | 1 |  |  |  |  |  |  |  |  |  |  |
|  | اس چوتیا کا جا کر چینل دیکھو تو پتہ چل جائے گا کہ یہ سالا ذہنی مریض ہے | 1 |  |  |  |  |  |  |  |  |  |  |
|  | اس چوتیے کو چھوڑو اور اس گاںڈو کوپکڑو | 1 |  |  |  |  |  |  |  |  |  |  |
|  | اس چوتیے نے صیح بولا کہ ڈول کے آنے کے بعد گناہ کم ہو جائے گا | 1 |  |  |  |  |  |  |  |  |  |  |
|  | اس دلے نے کاپی رائٹ لگا دینا ہے اس کی گانڈ جل جانی ہے | 1 |  |  |  |  |  |  |  |  |  |  |
|  | اس رنڈی کے طوخم کا بھائی پاکستان میں رہتا ہے اویٹر کا کھبا ٹٹہ | 1 |  |  |  |  |  |  |  |  |  |  |
|  | اس سے پہلے تیری ویڈٰیوز پر لائیک جاتے تے اب رپورٹس جائیں گیں | 0 |  |  |  |  |  |  |  |  |  |  |
|  | اس طرح کے کمنٹس مجھے اور کیری کو اشتعال دلاتے ہیں | 0 |  |  |  |  |  |  |  |  |  |  |
|  | اس کا انتظار تھا اور تم نے کردیا اور ویسے سینڈ شاہ میرے سکول سے تھا | 0 |  |  |  |  |  |  |  |  |  |  |
|  | اس کا علاج اس کی گانڈ میں تیل کی بوتل ڈال کر ہوگا | 1 |  |  |  |  |  |  |  |  |  |  |
|  | اس کا کوئی حق نہں بنتا کہ اس کو سبسکرائبر ملیں لیکن پھیر بھی اس کو سبسکرائبر مل گئے | 0 |  |  |  |  |  |  |  |  |  |  |
|  | اس کا ماں باپ کون ہے اور یہ رنڈی خود کون ہے | 1 |  |  |  |  |  |  |  |  |  |  |
|  | اس کا نہ باپ ہے اور نہ ماں ہے کھسرا ہے شام | 1 |  |  |  |  |  |  |  |  |  |  |
|  | اس کہ نہ ماں ہے نہ ابو یہ تو کھسرا ہے بہن چود | 1 |  |  |  |  |  |  |  |  |  |  |
|  | اس کو تو پوری طرح سے گانڈ مار دی | 1 |  |  |  |  |  |  |  |  |  |  |
|  | اس کو جاری رکھو اور اگر ٹائم ملے تو دنچک پوجا پر ویڈیو لازمی بناؤ | 0 |  |  |  |  |  |  |  |  |  |  |
|  | اس کو جاری رکھو یار نیا سال مبارک ہو | 0 |  |  |  |  |  |  |  |  |  |  |
|  | اس کو ختم کرو بھائی برا میوزک | 0 |  |  |  |  |  |  |  |  |  |  |
|  | اس کو دیکھ کر میں راوالپنڈی سے ناران تک گائیڈ لوں گا | 0 |  |  |  |  |  |  |  |  |  |  |
|  | اس کو سنجیدہ مت لیں یو یو بنتا ویڈیو ہماری سب سے بہتر ویڈیو ہے | 0 |  |  |  |  |  |  |  |  |  |  |
|  | اس کو شیخ گورمیت نے ڈائریکٹ کیاہے | 0 |  |  |  |  |  |  |  |  |  |  |
|  | اس کو کہتے ہیں اصلی مواد شام ادریس بھوسڑی کے بہن چود | 1 |  |  |  |  |  |  |  |  |  |  |
|  | اس کو کہتے ہیں بناء تیل لگائے گانڈ مارنا مزہ آگیا دکی بھائی | 1 |  |  |  |  |  |  |  |  |  |  |
|  | اس کو ویشنو ماتا کے بارے میں مزاق کرنے کے بعد ایک آدمی نے اس کوسبق سیکھایا سٹیشن پر | 0 |  |  |  |  |  |  |  |  |  |  |
|  | اس کے آڈیشن کے وقت مجھے لگا کہ اس کو کہیں دیکھا ہے پھیر مجھے آپ کی ویڈیو یاد آگئی | 0 |  |  |  |  |  |  |  |  |  |  |
|  | اس کے بعد شام کی گانڈ پھٹ جائے گی | 1 |  |  |  |  |  |  |  |  |  |  |
|  | اس کے پاس تو دونوں ہیں پر کیری کو ڈسک پسند ہے | 0 |  |  |  |  |  |  |  |  |  |  |
|  | اس کی ایسی کی تیسی کون ہے یہ سالی مارو اس کو بھائی کی ویڈیو چوری کرتی ہے | 0 |  |  |  |  |  |  |  |  |  |  |
|  | اس کی باتوں میں وزن ہے کہ تم یار گالیاں بہت نکالتے ہو اور وہ بھی انے واہ | 0 |  |  |  |  |  |  |  |  |  |  |
|  | اس کی تو نکل پڑی | 0 |  |  |  |  |  |  |  |  |  |  |
|  | اس کی گانڈ ماروں میں جب میں اپنے ٹٹے شیو کرتا ہوں تو اس کی گانڈ کی طرح ہو جاتے ہیں | 1 |  |  |  |  |  |  |  |  |  |  |
|  | اس کی لائیک کا مطلب ہے کہ ویڈیو کو دیکھیں | 0 |  |  |  |  |  |  |  |  |  |  |
|  | اس کی ماں چود کے پھینک دی دکی نے | 1 |  |  |  |  |  |  |  |  |  |  |
|  | اس کی ماں چودی پڑی ہے لن کی شکل والا مادر چود | 1 |  |  |  |  |  |  |  |  |  |  |
|  | اس کی ماں کا لوڑا | 1 |  |  |  |  |  |  |  |  |  |  |
|  | اس کی ماں کی آنکھ بہن چود | 1 |  |  |  |  |  |  |  |  |  |  |
|  | اس کی ماں کی بہن چود مادر چود ماں کا چھولا چوتیا گانڈو | 1 |  |  |  |  |  |  |  |  |  |  |
|  | اس کی ماں کی چوت اس کو کہو کر لے جو کرنا ہے | 1 |  |  |  |  |  |  |  |  |  |  |
|  | اس کی ویڈیوز مت بنا بھائی سر درد ہونے لگا ہے | 0 |  |  |  |  |  |  |  |  |  |  |
|  | اس گانڈو کا علاج شوکت خانم میں کروا کر ہسپتال کو گنا نہں کرنا | 1 |  |  |  |  |  |  |  |  |  |  |
|  | اس گانڈو نے سب سے پہلے میرا فیس بک پیچ بند کیا اور پھیر اپنے آپ کو پاکستانی کہتا ہے شرم آنی چاہیے اس گانڈو کو | 1 |  |  |  |  |  |  |  |  |  |  |
|  | اس گانے کا سب سے اچھا حصہ بہت مشہور تھا | 0 |  |  |  |  |  |  |  |  |  |  |
|  | اس گانے کو سننے کے بعد چین سموکر اس کو مار دیں گے | 0 |  |  |  |  |  |  |  |  |  |  |
|  | اس گیم کا نام پلیز | 0 |  |  |  |  |  |  |  |  |  |  |
|  | اس لڑکی کو زہر دے دو کس نے سچ کہا پیار اندھا ہوتا ہے | 0 |  |  |  |  |  |  |  |  |  |  |
|  | اس لنک پر کلک کر کے ایپلیکشن ڈون لوڈ کریں اور میرا کوڈ استعمال کر کے رجسٹر ہو جائیں | 0 |  |  |  |  |  |  |  |  |  |  |
|  | اس لنک کو بھی چیک کریں مہربانی فرما کر | 0 |  |  |  |  |  |  |  |  |  |  |
|  | اس لونڈیا کو شا م ادریس کا ٹٹہ بھی لائن مارے گا نا تو یہ اس کے ساتھ بھی ڈیٹ پر جائے گی چوتیا پاکستانی عوام | 1 |  |  |  |  |  |  |  |  |  |  |
|  | اس مردے جسم کو کیا کرنا ہے لوٹ لوسالوں کو | 1 |  |  |  |  |  |  |  |  |  |  |
|  | اس میں کوئی برائی نہیں ہے تبدیلی کے لیے اچھا ہے | 0 |  |  |  |  |  |  |  |  |  |  |
|  | اس نے اصل میں اس ویڈیو کے ردعمل میں ایسا کیا ہے | 0 |  |  |  |  |  |  |  |  |  |  |
|  | اس نے تو اس کی ماں بہن ایک کر کے رکھ دی میں نے کبھی ایسی ویڈیو نہں دیکھی بھائی | 1 |  |  |  |  |  |  |  |  |  |  |
|  | اس نے کچھ نیا کرنے کی کوشش کی ہے | 0 |  |  |  |  |  |  |  |  |  |  |
|  | اس نے کسی کے گانے کے بارے میں نہیں بولا اور تو نے اس کی گانڈ لے لی | 1 |  |  |  |  |  |  |  |  |  |  |
|  | اس نے لگا دئیے لوڑے | 1 |  |  |  |  |  |  |  |  |  |  |
|  | اس ویڈیو پر لاکھوں ویوز بنتے ہیں | 1 |  |  |  |  |  |  |  |  |  |  |
|  | اس ویڈیو کا امین بھائی سے کوئی لینا دینا نہیں ہے | 0 |  |  |  |  |  |  |  |  |  |  |
|  | اس ویڈیو کو دیکھیں بہت مزاحیہ ہے | 0 |  |  |  |  |  |  |  |  |  |  |
|  | اس ویڈیو کے بعد تو میں پکا | 0 |  |  |  |  |  |  |  |  |  |  |
|  | اس ویڈیو کے لیے آپ کا شکریہ اللہ آپ کو بہت ساری کامیابیاں دے | 0 |  |  |  |  |  |  |  |  |  |  |
|  | اس ویڈیو میں اتنا مزہ نہیں ہے | 0 |  |  |  |  |  |  |  |  |  |  |
|  | اسد، اشیش اور سر کیا حال ہیں | 0 |  |  |  |  |  |  |  |  |  |  |
|  | اسلم بہت بڑا کنجر ہے اور ساتھ میں رنڈی فراگی بھی | 1 |  |  |  |  |  |  |  |  |  |  |
|  | اسے تیرے بعد الفاظ والی ویڈیو دیکھا | 0 |  |  |  |  |  |  |  |  |  |  |
|  | اسے دیکھنے کے بعد میں بنگالی رہنے میں ڈر رہا ہوں | 0 |  |  |  |  |  |  |  |  |  |  |
|  | اسے کہتے ہیں گانڈ پھاڑ کر ہاتھ میں دینا تم نے اچھی طرح اسکا کام کیا | 1 |  |  |  |  |  |  |  |  |  |  |
|  | اشانت شرما سب سے بہتر تھا | 0 |  |  |  |  |  |  |  |  |  |  |
|  | اصلی نہیں ہیں | 0 |  |  |  |  |  |  |  |  |  |  |
|  | اعلیٰ بہن چود چود کر رکھ دیا ہے | 1 |  |  |  |  |  |  |  |  |  |  |
|  | اکبر نے انڈیا کے بچوں کے لیئے عطیات اکٹھے کیئے اور معذرت بھی کی | 0 |  |  |  |  |  |  |  |  |  |  |
|  | اکتوبر میں اس ویڈیو کا ٹائٹل دارو دارو سے تبدیل ہو گیا ہے وہ واپس آ گئی ہے | 0 |  |  |  |  |  |  |  |  |  |  |
|  | اگر اس ویڈیو پر اس نے احتجاج کیا تو قسم سے میں نے اس کی گانڈ مار دینی ہے | 1 |  |  |  |  |  |  |  |  |  |  |
|  | اگر اس ویڈیو کو پروموٹ کرنا چاہتے ہو تو بہن چودو اس کو شیئر کرو | 1 |  |  |  |  |  |  |  |  |  |  |
|  | اگر آپ سب کیری کے فائن ہو تو اس کمنٹ کو سب سے ذیادہ لائیکس دو | 0 |  |  |  |  |  |  |  |  |  |  |
|  | اگر آپ کو روسٹنگ پسند ہے تو آؤ میرا چینل جوائن کرو اور مزاحیہ ویڈیوز سے لطف اٹھاؤ | 0 |  |  |  |  |  |  |  |  |  |  |
|  | اگر بندہ خود شریف ہو تہ وہ دوسروں کی باتوں پر بہن چودی نہں کرتا آپ کی سب باتیں ٹھیکھ ہیں | 1 |  |  |  |  |  |  |  |  |  |  |
|  | اگر تم اس ویڈیو کو ناپسند کرتے ہو تو تم چوتیا ہو | 1 |  |  |  |  |  |  |  |  |  |  |
|  | اگر تم کو وہاں کچھ کام پڑا تو تیری ادھر جانے میں گانڈ پھٹ جائے گی تیری | 1 |  |  |  |  |  |  |  |  |  |  |
|  | اگر تم کیری مناتی کے بہت بڑے فا ئن ہو تو مجھے ٹک ٹاک پر فالو کریں | 0 |  |  |  |  |  |  |  |  |  |  |
|  | اگر شام ادریس لاکھوں لائیکس لے گا تو وہ دونوں شادی کر لیں گے ہمیں چوتیا بنا رہے ہیں | 1 |  |  |  |  |  |  |  |  |  |  |
|  | اگر ویڈیو میں گالیاں بالکل نہ ہوتیں تو اچھا رہتا | 0 |  |  |  |  |  |  |  |  |  |  |
|  | اگر یہ جادو والا گھر ہے تو یہاں بہت سارے جن رہتے ہوں گے | 0 |  |  |  |  |  |  |  |  |  |  |
|  | اگر یہ مشہور ہے تو سانڈیساہ مشہور ترین ہے | 0 |  |  |  |  |  |  |  |  |  |  |
|  | اگلے سطح کی بہن چودی ہے | 1 |  |  |  |  |  |  |  |  |  |  |
|  | الٹی آرہی ہے | 0 |  |  |  |  |  |  |  |  |  |  |
|  | الگ ہی بہن چودی پھیلاتا ہے مگر اچھا کام ہے | 1 |  |  |  |  |  |  |  |  |  |  |
|  | الگ ہی بہن چودی ہے | 1 |  |  |  |  |  |  |  |  |  |  |
|  | الگ ہی قس مے بہن چود ہو تم | 1 |  |  |  |  |  |  |  |  |  |  |
|  | اللہ الماس کو ہدایات دے اپنا چینل چلانے کے لیے دوسروں کی عزت سے کھیل رہا ہے | 0 |  |  |  |  |  |  |  |  |  |  |
|  | الماس جیکب آپ کے اوپر ویڈیو بنائی ہے | 0 |  |  |  |  |  |  |  |  |  |  |
|  | امیر کی کھوتی غریب کا جھونپڑا تیری ماں کا بھوسڑا | 1 |  |  |  |  |  |  |  |  |  |  |
|  | ان بن تن کا تولا شام ابلیس کی گانڈ میں لوڑٖا | 1 |  |  |  |  |  |  |  |  |  |  |
|  | ان جیسے فراگی بھوسڑی کے اپنی اوقات دیکھا رکھی ہے | 1 |  |  |  |  |  |  |  |  |  |  |
|  | ان دونوں کو بول دو کہ دودھ مانگو گے تو کھیر دیں گے پیسے مانگو گے تو چیر دیں گیں سستا تھوک لگا کر ان کی گانڈ | 1 |  |  |  |  |  |  |  |  |  |  |
|  | ان سب کی ماں چود دی دکی بھائی نے | 1 |  |  |  |  |  |  |  |  |  |  |
|  | ان سب کینیڈا والوں کو گانڈ کی کینسر ہے | 1 |  |  |  |  |  |  |  |  |  |  |
|  | ان کی بہن کی گھسی کنجری دا پتر پھٹے ہوئے کنڈم کی اولاد | 1 |  |  |  |  |  |  |  |  |  |  |
|  | ان گانڈ چودوں کو روک سالے حجاب کا مزاق اڑا رہے ہیں | 1 |  |  |  |  |  |  |  |  |  |  |
|  | ان لوگوں کو پتہ بھی نہں کہ یہ روسٹ لفظ کا کیا مطلب ہوتا ہے ان کو صرف اس لفظ سے نفرت ہے | 0 |  |  |  |  |  |  |  |  |  |  |
|  | انتہائی زہر ہے بھائی کی ویڈیو اعلٰی بھائی اور سالگرہ مبارک ہو | 0 |  |  |  |  |  |  |  |  |  |  |
|  | انٹرنیٹ کے ذریعے سے میں تمہارے چینل کو جانتا ہوں | 0 |  |  |  |  |  |  |  |  |  |  |
|  | انجالی وہ بہت پیاری ہے نا | 0 |  |  |  |  |  |  |  |  |  |  |
|  | اندر باہر کرو شام کھسری چوتیا | 1 |  |  |  |  |  |  |  |  |  |  |
|  | انڈیا کا ناصر خان جان | 0 |  |  |  |  |  |  |  |  |  |  |
|  | انڈیا کو گالی دے رہا ہے تو تم کو کچھ فرق نہیں پڑ رہا ہے | 0 |  |  |  |  |  |  |  |  |  |  |
|  | انرجی ڈرنک کو لسی بنا دیا | 0 |  |  |  |  |  |  |  |  |  |  |
|  | انہی چوتیاپا کی وجہ سے ین کو ان سبسکرائب کر دیا ہے | 1 |  |  |  |  |  |  |  |  |  |  |
|  | اہ بہت پاگل پن تک کا کردار تھا اب اس میں | 0 |  |  |  |  |  |  |  |  |  |  |
|  | او دلا | 1 |  |  |  |  |  |  |  |  |  |  |
|  | او دلے رنڈی کے بچے چت ماریں گے تیری تو کون ہوتا ہے شام کو جھوٹا کہنے والا | 1 |  |  |  |  |  |  |  |  |  |  |
|  | اودے شٹکر یہ کیسے متعلقہ ہے | 0 |  |  |  |  |  |  |  |  |  |  |
|  | اور اچھا کام ہے آپ کا تم بی بی سے اچھی اور جلدی ویڈیوز دے رہے ہو | 0 |  |  |  |  |  |  |  |  |  |  |
|  | اور اس پر بناؤ ویڈیو | 0 |  |  |  |  |  |  |  |  |  |  |
|  | اور اس چوتیے بہن کے لوڑے کو اتنا روسٹ کر کہ اس کا چینل خود ہی بند ہو جائے | 1 |  |  |  |  |  |  |  |  |  |  |
|  | اور ایک بات کیا تمہارا نام اجے نگر ہے | 0 |  |  |  |  |  |  |  |  |  |  |
|  | اور آپ پر وہ کیوں نہں ویڈیو روسٹ کرتا کیوں کہ اس کے لیے جھوٹ کا سہارا لینا پڑتا ہے وہ گانڈو عقل کے ساتھ تو کچھ لکھتا نہیں ہے | 1 |  |  |  |  |  |  |  |  |  |  |
|  | اور آج کل شادی والا ہی چوتیاپا چل رہا ہے ان کا | 1 |  |  |  |  |  |  |  |  |  |  |
|  | اور تیری ماں دکی کی گانڈ چٹوانے والی دکی کی گانڈ | 1 |  |  |  |  |  |  |  |  |  |  |
|  | اور جیری سے ابھی تک نہں ملے بھائی | 0 |  |  |  |  |  |  |  |  |  |  |
|  | اور سالگرہ بھی مبارک ہو | 0 |  |  |  |  |  |  |  |  |  |  |
|  | اور مست غضب بنایا | 0 |  |  |  |  |  |  |  |  |  |  |
|  | اور ہاں دکی بھائی اس نے اپنے چینل پر گانڈوپن تونہیں کیا | 1 |  |  |  |  |  |  |  |  |  |  |
|  | اور ہماری مزاحیہ ویڈیو لازمی دیکھیں اور اچھی لگے تو لازمی سبسکرائیب کریں | 0 |  |  |  |  |  |  |  |  |  |  |
|  | اور وہ گرنیڈ پھینک سکتاتھا اس کو اپنے آپ کو مارنے کی ضرورت نہیں | 0 |  |  |  |  |  |  |  |  |  |  |
|  | اور یار میں ہنس ہنس کر رو پڑا | 0 |  |  |  |  |  |  |  |  |  |  |
|  | اور یہ بندہ لڑکا ہے یا لڑکی ہے کنفیوشن ہے | 0 |  |  |  |  |  |  |  |  |  |  |
|  | اوسم کیری بھائی آئی لو یو | 0 |  |  |  |  |  |  |  |  |  |  |
|  | اولام کے داخل ہونے پر کون سا گانا شروع میں چلایا تھا | 0 |  |  |  |  |  |  |  |  |  |  |
|  | اوم پرکاش بڑھیا ہے روسٹ کی وجہ سے وہ زیادہ مشہور ہو گیا اس سے دونوں کا فائدہ ہے | 0 |  |  |  |  |  |  |  |  |  |  |
|  | اوم پرکاش شرما کی لے لی | 0 |  |  |  |  |  |  |  |  |  |  |
|  | اومے تمہاری سب سے مزاحیہ ویڈیو ہے | 0 |  |  |  |  |  |  |  |  |  |  |
|  | اونام گپتا بےوفا ہے | 0 |  |  |  |  |  |  |  |  |  |  |
|  | اوہ اب یہ بھی دیکھنا پڑے گا | 0 |  |  |  |  |  |  |  |  |  |  |
|  | اوہ بھائی تم جانتے ہو بھوج پوری کے گانوں کے بارے میں تم کسی معاشرے کو تنقید نہں کر سکتے | 0 |  |  |  |  |  |  |  |  |  |  |
|  | اوہ بھائی جی | 0 |  |  |  |  |  |  |  |  |  |  |
|  | اوہ میرے خدا آپ کی یہ ویڈیو بہت اچھی ہے | 0 |  |  |  |  |  |  |  |  |  |  |
|  | اوہ میرے خدا شکریہ آپ نے مجھے جواب دیا | 0 |  |  |  |  |  |  |  |  |  |  |
|  | اوہ میرے خدا کا دیکھنا تھا اس کا | 0 |  |  |  |  |  |  |  |  |  |  |
|  | اوہ میرے خدا میں بڑبڑا رہا ہوں تم نے یہ کیسے کیا تمہیں یہ کرنے کا موقع کیسے ملا میں حیران ہوں کہ یہ اصلی ہے یا میں خواب میں ہوں | 0 |  |  |  |  |  |  |  |  |  |  |
|  | اوہ میری گانڈ میں ٹیکے ہا ہا ہا ہا | 1 |  |  |  |  |  |  |  |  |  |  |
|  | اوہ نہں میں کافی لیٹ ہوں | 0 |  |  |  |  |  |  |  |  |  |  |
|  | اوہم پرکاش سرما کا انڈین آئڈل آڈیشن | 0 |  |  |  |  |  |  |  |  |  |  |
|  | اوئے باز آجاکوڑی کی ٹکی کا فائن منہ بند کر اپنا میں نے تیرے ساتھ بات نہں کی گدھے جئیسی شکل ہے تیری | 1 |  |  |  |  |  |  |  |  |  |  |
|  | اوئے بھائی میں کب سے تلاش کر رہا ہوں میوزک صیح بج رہا ہے نا | 0 |  |  |  |  |  |  |  |  |  |  |
|  | اوئے چوتیے شاید تجھے پولیو والا ٹیکہ نہں ملا | 1 |  |  |  |  |  |  |  |  |  |  |
|  | اوئے دکی تو چوتیا تمیز کر وہ نہ بھی کرے تو تجھے کیا | 1 |  |  |  |  |  |  |  |  |  |  |
|  | اوئے دلے ایک نمبر ویڈیو | 1 |  |  |  |  |  |  |  |  |  |  |
|  | اوئے کمینے دکی سن زرا تو نے لکھا کہ چھوتے یوٹیوبر کی کوئی عزت نہیں کرتا لیکن گانڈ مروانے میں تو ان کی عزت کرتا ہے | 1 |  |  |  |  |  |  |  |  |  |  |
|  | اوئے گانڈو عمرا ن کو بیچ میں مت لا | 1 |  |  |  |  |  |  |  |  |  |  |
|  | اوئے ماروں گا ہماری ماں کے لوڑے | 1 |  |  |  |  |  |  |  |  |  |  |
|  | اوئے یار دکی تو کس چوتیاپا میں پڑ گیا ہے بہت بورنگ ویڈیو ہے یار | 1 |  |  |  |  |  |  |  |  |  |  |
|  | اے بھائی آئی لو یوتمہارے کام کے لئے میں بھی پہلے تھا ان کا فائن لیکن بہن چود مادر چود نے اتنا چوتیاپا دیکھایا کہ بس میں کیا کہوں | 1 |  |  |  |  |  |  |  |  |  |  |
|  | اے روپالی پکڑ میری ڈالی | 0 |  |  |  |  |  |  |  |  |  |  |
|  | اے روپالی کا ہالی ووڈ ورژن دیکھو | 0 |  |  |  |  |  |  |  |  |  |  |
|  | ایسے کون پھانسی لگاتا ہےزمیں پر رکھے ہوئے پنکھے میں رسی دال کر | 0 |  |  |  |  |  |  |  |  |  |  |
|  | ایسے کیوں لگ رہا ہے کہ کیری کی پھٹی ہوئی تھی ٹام کروز کے سامنے سالے انڈین ہی رہو گے سالے تم | 1 |  |  |  |  |  |  |  |  |  |  |
|  | ایسے گانے بنانے والوں کو جیل بھیج دینا چاہیے | 0 |  |  |  |  |  |  |  |  |  |  |
|  | ایسے لگ رہا ہے جیسے کوئی چوتیا کوئی سکیم ویڈیوز دکی بھائی کی بھیج رہا ہے | 1 |  |  |  |  |  |  |  |  |  |  |
|  | ایسے لوگوں کی اور گانڈ مارو تیل لگا کر | 1 |  |  |  |  |  |  |  |  |  |  |
|  | ایسی ویڈیو بنا تیرے ہی نا | 0 |  |  |  |  |  |  |  |  |  |  |
|  | ایک اور بھی چوتیا پروان چڑھ رہا ہے | 1 |  |  |  |  |  |  |  |  |  |  |
|  | ایک بات بتا کیری تم ہر دن بیمار ہی رہتا ہےکیا | 0 |  |  |  |  |  |  |  |  |  |  |
|  | ایک جو اپنی روح کو اپنی شہرت کے لیئے فروخت کر دیتا ہے | 0 |  |  |  |  |  |  |  |  |  |  |
|  | ایک شام اور دوسرا لوگان پال ان دونوں سے بڑا چوتیا اور کوئی نہں | 1 |  |  |  |  |  |  |  |  |  |  |
|  | ایک طرف اس کا چتیاپا اور دوسری طرف آپ کی گندی باتیں اتنا اگر کوئی مرے خلاف بولتا تو قسم سے اس کی گانڈ لیرولیر کردیتا | 1 |  |  |  |  |  |  |  |  |  |  |
|  | ایک لمبے عرصہ کے بعد ایک اچھی ویڈیو | 0 |  |  |  |  |  |  |  |  |  |  |
|  | ایک نمبر کا بہن چود ہے بھائی یہ میں کہ رہا ہوں اب تو مار کے دیکھا | 1 |  |  |  |  |  |  |  |  |  |  |
|  | ایک نمبر کا چوتیا ہے شام | 1 |  |  |  |  |  |  |  |  |  |  |
|  | ایک نمبر کا چوتیا ہے شام مجھے تو اس بھوسڑی والے کی شکل بھی پسند نہیں ہے | 1 |  |  |  |  |  |  |  |  |  |  |
|  | ایک ویڈیو میں تو نے میوزک کو پروموٹ کیا تھا اب یوزر بہت سارے تجھے پروموٹ کر رہے ہیں بالکل | 0 |  |  |  |  |  |  |  |  |  |  |
|  | آ بھوسڑی والوں چائے پیتے ہیں | 1 |  |  |  |  |  |  |  |  |  |  |
|  | آپ سے گزارش ہے کہ جتنا ہو سکے زیادہ سے زیادہ اس ویڈیو کو نا پسند کریں کیوںکہ یہ بھڑوا بھوجی پوری کا مزاق اڑاتا ہے | 1 |  |  |  |  |  |  |  |  |  |  |
|  | آپ کا کام بہت بہت اچھا تھا | 0 |  |  |  |  |  |  |  |  |  |  |
|  | آپ کا مقصد مکمل ہو گیا | 0 |  |  |  |  |  |  |  |  |  |  |
|  | آپ کو لاکھوں لائیکس ملیں گیں تم بہت اچھے ہو | 0 |  |  |  |  |  |  |  |  |  |  |
|  | آپ کون سا سافٹ وئیر استعمال کرتے ہو اپنی ویڈیوز میں بتاؤ نا بھائی | 0 |  |  |  |  |  |  |  |  |  |  |
|  | آپ کے ہاتھ میں کیا ٹیٹو بنا ہوا ہے | 0 |  |  |  |  |  |  |  |  |  |  |
|  | آپ کی ٹھگ زندگی کا بیک گراؤنڈ آتا جاتا ہے | 0 |  |  |  |  |  |  |  |  |  |  |
|  | آپ کی گانڈ میں کھجلی کیوں ہو رہی ہےکچھ بھی کریں تو ڈرامہ یہ لوگ | 1 |  |  |  |  |  |  |  |  |  |  |
|  | آپ کی ویڈیوز میں اب پہلی جیسی بات نہیں رہی | 0 |  |  |  |  |  |  |  |  |  |  |
|  | آپ میں سے جو بھی اس کے بلاگ دیکھتا ہے تو آج اس کے بلاگ میں گندی گندی گالیاں ڈالیں | 1 |  |  |  |  |  |  |  |  |  |  |
|  | آپ نے تو اس کی تنگ کر کے لے لی اب بھی اگر وہ اس ویڈیو کو دیکھ کر اور ویڈیو بناتا ہے تو لعنت ہے اس پر | 1 |  |  |  |  |  |  |  |  |  |  |
|  | آپ نے تو شام کی گانڈ لے لی شاباش بھائی | 1 |  |  |  |  |  |  |  |  |  |  |
|  | آتش اجالا کنسلٹ | 0 |  |  |  |  |  |  |  |  |  |  |
|  | آج تو تونے خان کے بارے میں بتا کر آنکھیں کھول دیں او دلے | 1 |  |  |  |  |  |  |  |  |  |  |
|  | آج سے ٹیم نے یہ فیصلہ کیا ہے کہ ویڈیو سبسکرائب اور پسند نہ کرنے والی گانڈ مٰیں ڈنڈا دیں کیا تیری گانڈ میں ڈنڈا دوں میں | 1 |  |  |  |  |  |  |  |  |  |  |
|  | آج کل اس کمینے نے ملین سبسکرائبرز کا نیا رونا شروع کیا ہوا ہے | 1 |  |  |  |  |  |  |  |  |  |  |
|  | آج گانڈ کھلے گی ابلیس کی یقین مانو آج شام کی گانڈ مارنے کا وقت آگیا ہے | 1 |  |  |  |  |  |  |  |  |  |  |
|  | آج میں لگ رہا ہوں سیکسی | 0 |  |  |  |  |  |  |  |  |  |  |
|  | آج والا اتنا مزہ نہیں آیا اس لیے کوئی لائیک نہں | 0 |  |  |  |  |  |  |  |  |  |  |
|  | آخر پر کون سا گانا استعمال ہوا ہے | 0 |  |  |  |  |  |  |  |  |  |  |
|  | آخر کار گیم کھیلنا دوبارہ سے شروع ہوگا | 0 |  |  |  |  |  |  |  |  |  |  |
|  | آخر کارسب سے زیادہ بولنے والوں کوجواب مل ہی گیا | 0 |  |  |  |  |  |  |  |  |  |  |
|  | آخر گیا یہ ہے چوتیا جھوٹا انسان آپ کی ویڈیوز بہت پیاری ہیں حقیقت میں بہت پیاری ہیں | 1 |  |  |  |  |  |  |  |  |  |  |
|  | آخر میں آپ کا دیکھنا بہت پیارا لگا | 0 |  |  |  |  |  |  |  |  |  |  |
|  | آخر میں آجے کا یہ کلپ بھہت اچھا تھا | 0 |  |  |  |  |  |  |  |  |  |  |
|  | آخر میں وہ بندہ بہت شریف بننے کی کوشش کر رہا تھا | 0 |  |  |  |  |  |  |  |  |  |  |
|  | آخر میں وہ چیٹ کا گانا سب سے بڑھیا لگا | 0 |  |  |  |  |  |  |  |  |  |  |
|  | آخرکار پتہ چل گیا کہ اس کی گانڈ ویسے ہی بچپن سے بہت کھلی ہے تیل لگانے کی بھی ضرورت نہں پڑتی | 1 |  |  |  |  |  |  |  |  |  |  |
|  | آخرکار تم نے سب سے اچھا ویڈیو بنا ڈالا | 0 |  |  |  |  |  |  |  |  |  |  |
|  | آخری ویڈیو کے گانے کوپورا کر کے اس کا لنک بنا دو ڈون لوڈ کے لیے گانا بہت اچھا ہے | 0 |  |  |  |  |  |  |  |  |  |  |
|  | آریام تجھے پیدا کرنا بھی میرے باپ کی غلطی لگتی ہے | 0 |  |  |  |  |  |  |  |  |  |  |
|  | آریان چل بھاگ تو وہی ویڈیو دیکھ نا جا کر کیوں دیکھتے آتے ہو | 0 |  |  |  |  |  |  |  |  |  |  |
|  | آسمانی لنڈ کو کھٹی لسی کی پچکاری دی جائے | 1 |  |  |  |  |  |  |  |  |  |  |
|  | آفریدی چپ کر چوتیے | 1 |  |  |  |  |  |  |  |  |  |  |
|  | آکاش کمار اس مزاحیہ ویڈیو کو دیکھں اور لنک پر کلک کریں اور مہربانی کر کے سبسکرائب کریں | 0 |  |  |  |  |  |  |  |  |  |  |
|  | آگےسے ہم پاکستانی اور انڈین تمہارے سب فائن مل کراس کی ماں چود دیں گے | 1 |  |  |  |  |  |  |  |  |  |  |
|  | آن لائن گروٹیچ چیک آؤٹ میرا چینل اور امتحان | 0 |  |  |  |  |  |  |  |  |  |  |
|  | آؤ بھوسڑی والوں چائے پیتے ہیں | 1 |  |  |  |  |  |  |  |  |  |  |
|  | آئےیوراما تم ہم کو کیا کیا دیکھاتا ہےیہ میچ تو کافی دلچسپ ہے | 0 |  |  |  |  |  |  |  |  |  |  |
|  | بابا مر جائے گا اس کو دیکھیں | 0 |  |  |  |  |  |  |  |  |  |  |
|  | باپ باپ ہوتا ہے | 0 |  |  |  |  |  |  |  |  |  |  |
|  | باپ کا بھائی کا ٹی سیرز کا سب کا بدلہ لے گا یہ | 0 |  |  |  |  |  |  |  |  |  |  |
|  | بات کرنا پڑتی ہے کہ کیسے وہ ایک بہت بڑے سٹار کا انٹرویو کر سکتا ہے بغیر کسی تجربہ کے | 0 |  |  |  |  |  |  |  |  |  |  |
|  | باراک اوباما لوڑا | 1 |  |  |  |  |  |  |  |  |  |  |
|  | باس مہربانی کر کے ملائیکہ کے بارے میں کچھ بتائیں | 0 |  |  |  |  |  |  |  |  |  |  |
|  | بالکل اسی طرح تم بھی دکی دلے ہو گانڈو | 1 |  |  |  |  |  |  |  |  |  |  |
|  | بالکل بھائی یہ پرانا استعمال کیا ہوا کھسرا ہے سب کو چوتیا سمجھتا ہے صیح کھول کے رکھ دیا آپ نے اس کو | 1 |  |  |  |  |  |  |  |  |  |  |
|  | بالکل صیح کہا میرے بھائی گانڈ میں ٹیکے لگوا کے ہی گورا ہوا ہے کتے کا بچہ | 1 |  |  |  |  |  |  |  |  |  |  |
|  | بالکل صیح ماں بہن ایک کی کے بھوسڑی کے کی | 1 |  |  |  |  |  |  |  |  |  |  |
|  | بائیوگرافی بی بی کے بعد کیری | 0 |  |  |  |  |  |  |  |  |  |  |
|  | ببن چوتیا ہے سالا | 1 |  |  |  |  |  |  |  |  |  |  |
|  | بچو بہت دیر کے بعد ویڈیو ڈال رہا ہے | 0 |  |  |  |  |  |  |  |  |  |  |
|  | بخشی بھائی ہم نے آپکا چینل سبسکرائب کر دیا ہے اب آپ جلدی سے واپیس ہمارے چینل کو سبسکرائب کرو | 0 |  |  |  |  |  |  |  |  |  |  |
|  | برا لگا تو سوری | 0 |  |  |  |  |  |  |  |  |  |  |
|  | برا نہ منانا تجھے بہت چوتیا بنایا ہے | 1 |  |  |  |  |  |  |  |  |  |  |
|  | برا نہ منانا حمزہ بھائی | 0 |  |  |  |  |  |  |  |  |  |  |
|  | برا وقت برا وقت ان پر آتا ہے جو برے ہوتے ہیں اور یہ تو عرفان بھائی ہے یہ تو اچھا بچہ ہے | 0 |  |  |  |  |  |  |  |  |  |  |
|  | برابر کر دیا سب کچھ بھائی نے چوتیاپا کرتے تھے یہ لوگ | 1 |  |  |  |  |  |  |  |  |  |  |
|  | برچودی اور چت مرانی بہن کی لوڑی باس دا چودی | 1 |  |  |  |  |  |  |  |  |  |  |
|  | برچودی خود گالی دے رہی ہے دوسروں کو بولتی ہے | 1 |  |  |  |  |  |  |  |  |  |  |
|  | برقع والی کو کیوں دیکھ رہا ہے ہماری طرف دیکھ | 0 |  |  |  |  |  |  |  |  |  |  |
|  | برہان حسن بھائی لیکن انڈیا کو لوسر بولنے والا بھوسڑی کا ہوتا کون ہے | 1 |  |  |  |  |  |  |  |  |  |  |
|  | برے ہیں ہم لوگ کیا کریں لوگ بھوسڑی کے بار بار پوچھتے رہتے ہیں کیسے ہیں آپ لوگ کیسے ہیں آپ لوگ | 1 |  |  |  |  |  |  |  |  |  |  |
|  | بری بات ہے تو بری بات ہے | 0 |  |  |  |  |  |  |  |  |  |  |
|  | بری بات ہے وہ مزاحیہ تھا | 0 |  |  |  |  |  |  |  |  |  |  |
|  | بڑا ہی بہن چود ہے بھائی تو ہمارے ساتھ بہت جمے گی تیری | 1 |  |  |  |  |  |  |  |  |  |  |
|  | بڑے خان بھائی جان یہ پاگل ہے جو ابھی ابھی شام کا فائن ہوا ہے | 1 |  |  |  |  |  |  |  |  |  |  |
|  | بڑی تو انگریز صیح کہا بھوسڑی کی | 1 |  |  |  |  |  |  |  |  |  |  |
|  | بڑی گانڈ جلتی ہے تم لوگوں کی میرا لن چوتیا آدمی بس کر بھڑوے | 1 |  |  |  |  |  |  |  |  |  |  |
|  | بڑی لمبی کر دی اس بار پہ پرجو بھی ہو سچ ہی کہا ہے چوتیا ابلیس اور ہاں محترم حجاب | 1 |  |  |  |  |  |  |  |  |  |  |
|  | بس اک چیز سوچتا ہوں اکژ کہ قیامت کے دن کیا منہ دیکھائیں گے | 0 |  |  |  |  |  |  |  |  |  |  |
|  | بس ایک بات میں کیری کے لیے کہنا چاہتا ہوں کہ گانڈ مارو اس سالے کی ٹکا کر | 1 |  |  |  |  |  |  |  |  |  |  |
|  | بس بکواس کرنے کو دے دو | 0 |  |  |  |  |  |  |  |  |  |  |
|  | بس بھائی جھلی عوام | 0 |  |  |  |  |  |  |  |  |  |  |
|  | بس بھائی یہ سب فالتو چیزیں ہیں عروج پر ہیں اس بہن چودی سے تو صیح میں کھل گیا بھائی لوگ میرا چینل دیکھ لو | 1 |  |  |  |  |  |  |  |  |  |  |
|  | بس بیک گراؤنڈ میوزک بدلو اور خوفناک منظر کی بھی گانڈ مار لو | 1 |  |  |  |  |  |  |  |  |  |  |
|  | بس ٹانگیں کھینچو ایک دوسرے کی | 0 |  |  |  |  |  |  |  |  |  |  |
|  | بس چیک کرو شہزاد بھائی | 0 |  |  |  |  |  |  |  |  |  |  |
|  | بس شام کی تو تو نے گانڈ ہی گندی کر دی | 1 |  |  |  |  |  |  |  |  |  |  |
|  | بس کر بہن چود | 0 |  |  |  |  |  |  |  |  |  |  |
|  | بس کر پہلے اب کیا رولائے گا | 0 |  |  |  |  |  |  |  |  |  |  |
|  | بس کر دے بھائی اب اس چوتیے کو نہیں دیکھں | 1 |  |  |  |  |  |  |  |  |  |  |
|  | بس کر رلائے گا کیا پگلے | 0 |  |  |  |  |  |  |  |  |  |  |
|  | بس کریار تیرے یہ چوتیے کی ویڈیو پھر سے مت بنانا | 1 |  |  |  |  |  |  |  |  |  |  |
|  | بس ہو گیا بھائی تیری چوتیا گری | 1 |  |  |  |  |  |  |  |  |  |  |
|  | بس یار ایسی ہی ویڈیو بنائی کر | 0 |  |  |  |  |  |  |  |  |  |  |
|  | بس یہ آخری بات نے دل جیت لیا لیکن نظام تعلیم بھی اسی کے ہاتھ میں ہے | 0 |  |  |  |  |  |  |  |  |  |  |
|  | بس یہ تو چاہیے تھا اب شئیر کروں گا لوگوں کو | 0 |  |  |  |  |  |  |  |  |  |  |
|  | بس یہی ویڈیو کا ہی انتظار تھا وہ بھی پورا ہو گیا اور ویڈیو میں بھی مزہ آگیا | 0 |  |  |  |  |  |  |  |  |  |  |
|  | بشریٰ آپ ابلیس کی طرف داری کیوں کر رہی ہو | 0 |  |  |  |  |  |  |  |  |  |  |
|  | بشریٰ آصف قریشی تو چوتیا ہے | 1 |  |  |  |  |  |  |  |  |  |  |
|  | بعد میں جو اس کی گانڈ ماری ہے اس کے لئیے شکریہ | 1 |  |  |  |  |  |  |  |  |  |  |
|  | بغیر کسی واسطے کے بن گئی جو کہ مدد کر رہا ہے | 0 |  |  |  |  |  |  |  |  |  |  |
|  | بکواس ترین یوٹیوبر ہے لعنت بھیجو اس پر | 1 |  |  |  |  |  |  |  |  |  |  |
|  | بکواس چیک کرو شام ادریس کی ایک اور ویڈیو ایک اور چتیاپا | 1 |  |  |  |  |  |  |  |  |  |  |
|  | بکواس مت کر کتے باز آجا بوتھی نہیں دیکھی اپنی جیسے شیشہ ٹوٹ جاتا ہوگا | 1 |  |  |  |  |  |  |  |  |  |  |
|  | بگ باس کا بگ باس واپس آگیا ہے | 0 |  |  |  |  |  |  |  |  |  |  |
|  | بگ باس کی مارنی ہے کیا | 0 |  |  |  |  |  |  |  |  |  |  |
|  | بگ باس والے تو اس کو بھی بلا لیں گے | 0 |  |  |  |  |  |  |  |  |  |  |
|  | بگ باس ویڈیو کہاں ہے | 0 |  |  |  |  |  |  |  |  |  |  |
|  | بگ بیش میں تجھے کچھ بولا لوڑے | 1 |  |  |  |  |  |  |  |  |  |  |
|  | بلغم سے شادی کر لے بیٹا | 0 |  |  |  |  |  |  |  |  |  |  |
|  | بن چو نائٹ کو بھی | 0 |  |  |  |  |  |  |  |  |  |  |
|  | بنتے برا مان جائے گا رے | 0 |  |  |  |  |  |  |  |  |  |  |
|  | بندے منگواؤ اور اس مادر چود کو اٹھاواؤ کینیڈا سے اور اس کو پاکستان میں لا کر چچا مجبور کے پاس چھوڑ دو | 1 |  |  |  |  |  |  |  |  |  |  |
|  | بنڈ پھاڑ دی بہن چود کی | 1 |  |  |  |  |  |  |  |  |  |  |
|  | بنڈ مار دے اس بہن چود کی | 1 |  |  |  |  |  |  |  |  |  |  |
|  | بنگالی ہونے کے ناطے میں آپ کو بتا دوں کہ آپ کا ردعمل بہت خراب ہے | 0 |  |  |  |  |  |  |  |  |  |  |
|  | بہاریوں کی تو تم لوگ گانڈ مار لیتے ہو | 1 |  |  |  |  |  |  |  |  |  |  |
|  | بہت اچھا اس کی گانڈ بند کردو ہم تمہارے ساتھ ہیں | 1 |  |  |  |  |  |  |  |  |  |  |
|  | بہت اچھا اوے کیری | 0 |  |  |  |  |  |  |  |  |  |  |
|  | بہت اچھا آپ نے ان کو ظاہر کیا لوڑے شام اور مینڈک کی للی کی منہ والی فراگی کو | 1 |  |  |  |  |  |  |  |  |  |  |
|  | بہت اچھا بھائی تمہاری ویڈیوز بہت پسند آئیں اب تو تم خوابوں میں بھی آرہے ہو | 0 |  |  |  |  |  |  |  |  |  |  |
|  | بہت اچھا کام تھا آپ کا بھائی شاباش | 0 |  |  |  |  |  |  |  |  |  |  |
|  | بہت اچھا کام تھا شاباش میں اس گانڈو کا فین تھا لیکن تم نے میری آکنھیں کھول دیں | 1 |  |  |  |  |  |  |  |  |  |  |
|  | بہت اچھا کام ہے جناب | 0 |  |  |  |  |  |  |  |  |  |  |
|  | بہت اچھا گانڈ پھاڑ کر رکھ دی کالا ٹٹہ اور اسک کے بائیں طرٖف کا ٹٹہ فراگی | 1 |  |  |  |  |  |  |  |  |  |  |
|  | بہت اچھا گانڈ مار دے ان بہن چودوں کی کسی کنجری کا شام | 1 |  |  |  |  |  |  |  |  |  |  |
|  | بہت اچھا یار اس دلے کو رلانے کے لیے سالا بھوسڑی والا جھوٹا | 1 |  |  |  |  |  |  |  |  |  |  |
|  | بہت اچھا یار اس کی اور گانڈ مارو | 1 |  |  |  |  |  |  |  |  |  |  |
|  | بہت اچھاخیال ہے بھائی | 0 |  |  |  |  |  |  |  |  |  |  |
|  | بہت اچھی کیری تم اپنا اچھا کام جاری رکھو | 0 |  |  |  |  |  |  |  |  |  |  |
|  | بہت اچھی ویڈیو لیکن بکواس ہے یہ سب | 0 |  |  |  |  |  |  |  |  |  |  |
|  | بہت اچھی ویڈیو ہے اور میں اس کو دیکھ رہا ہوں | 0 |  |  |  |  |  |  |  |  |  |  |
|  | بہت اچھی ویڈیو ہے کیری بھائی اتنے دن کے بعد گردا آڑا دیا | 0 |  |  |  |  |  |  |  |  |  |  |
|  | بہت اعلیٰ بہن چودی کی ویڈیو اور تھمبنل ڈال کر کیا پاگل بنایا سب کو | 1 |  |  |  |  |  |  |  |  |  |  |
|  | بہت اعلیٰ یار اس طرح ہی گانڈ مارتے رہو اس طرح کے لوگوں کی | 1 |  |  |  |  |  |  |  |  |  |  |
|  | بہت اعلی بھائی اس کو اور بہتر کرو | 0 |  |  |  |  |  |  |  |  |  |  |
|  | بہت اعلی جواب تو بھوسڑی کے باز آجا اپنی ان حرکتوں سے | 1 |  |  |  |  |  |  |  |  |  |  |
|  | بہت افسوس ہوا اس بار شام بہن چود ایک جھوٹا اور چوتیا انسان ہے اس کے لیے تو ایسے الفاظ بہت کم ہیں | 1 |  |  |  |  |  |  |  |  |  |  |
|  | بہت افسوس ہوا بھائی گھس کے پھٹ گیا شام کی گانڈ میں | 1 |  |  |  |  |  |  |  |  |  |  |
|  | بہت انتظار کروا دیا بہت اچھا | 0 |  |  |  |  |  |  |  |  |  |  |
|  | بہت برا ایک تو اتنے عرصہ کے بعد ویڈیو ڈالا ہے اوپر سے خاص بھی نہیں لگی تو نے بولا تو اس لئیے لائیک بھی کر دیا | 0 |  |  |  |  |  |  |  |  |  |  |
|  | بہت برا وقت تھا جب دکی گانڈو نے ہم سب سے زیادہ اشتہا رات لیے | 1 |  |  |  |  |  |  |  |  |  |  |
|  | بہت بڑا حدف ہے اور اس کو حاصل کرنے میں ٹائم لگے گا | 0 |  |  |  |  |  |  |  |  |  |  |
|  | بہت بڑھیا ویڈیو | 0 |  |  |  |  |  |  |  |  |  |  |
|  | بہت بہت زیادہ ایکٹنگ ہو گئی اس ویڈیو میں | 0 |  |  |  |  |  |  |  |  |  |  |
|  | بہت بہت مبارک ہو بھائی تم بہت اچھے ہو میرے پاس آپ کی اس ویڈیو کے لیے کوئی الفاظ نہیں ہیں | 0 |  |  |  |  |  |  |  |  |  |  |
|  | بہت بہن چودی کرتے ہو یار | 1 |  |  |  |  |  |  |  |  |  |  |
|  | بہت چوتیا آدمی ہے یہ سالا | 1 |  |  |  |  |  |  |  |  |  |  |
|  | بہت چودی بہت الگ ہی لیول کا ویڈیو تھا | 1 |  |  |  |  |  |  |  |  |  |  |
|  | بہت خراب ویڈیو بورنگ | 0 |  |  |  |  |  |  |  |  |  |  |
|  | بہت خوب یار اس بار کی ویڈیو تو بہت غضب تھی مزہ آگیا | 0 |  |  |  |  |  |  |  |  |  |  |
|  | بہت دلچسپ بھائی میں ابھی پاکستان گیا ہوں پر تیرے جیسے لونڈےباز نہں ملا مجھے | 1 |  |  |  |  |  |  |  |  |  |  |
|  | بہت دنوں بعد بھائی | 0 |  |  |  |  |  |  |  |  |  |  |
|  | بہت دنوں کے بعد ایسا ویڈیو آیا بہت ہنسی آئی | 0 |  |  |  |  |  |  |  |  |  |  |
|  | بہت زیادہ تیز تو نہیں تھی لیکن تیز تھی | 0 |  |  |  |  |  |  |  |  |  |  |
|  | بہت زیادہ مبارک ہو ویڈیو | 0 |  |  |  |  |  |  |  |  |  |  |
|  | بہت سیکسی اور پیاری لڑکیوں کو ٹک ٹاک پر دیکھو اور میرا چینل فٹافٹ سبسرائب کرو | 0 |  |  |  |  |  |  |  |  |  |  |
|  | بہت شاندار ویڈیو بنائی ہے آپ نے بھائی | 0 |  |  |  |  |  |  |  |  |  |  |
|  | بہت شاندار ویڈیو ہے یہ | 0 |  |  |  |  |  |  |  |  |  |  |
|  | بہت صیح ویڈیو بنائی اس بار آپ نے ان کی ان لوگوں نے حد کر رکھی تھی | 0 |  |  |  |  |  |  |  |  |  |  |
|  | بہت صیح ویڈیو تھی گانڈ مار اس سالے گانڈو کی | 1 |  |  |  |  |  |  |  |  |  |  |
|  | بہت عزت ہے انڈیا کی طرف سے اس کو جاری رکھو کیری ہے ہی چوتیا سالا جھوٹا کہیں کا | 1 |  |  |  |  |  |  |  |  |  |  |
|  | بہت عظیم ویڈیو ریپر ہے بھائی | 0 |  |  |  |  |  |  |  |  |  |  |
|  | بہت عمدہ یار ہنس ہنس کے پیٹ درد کر رہا ہے بہرحال نسا کا مطلب آسام میں نشہ ہے | 0 |  |  |  |  |  |  |  |  |  |  |
|  | بہت عمدو مزاحیہ چینل تھا | 0 |  |  |  |  |  |  |  |  |  |  |
|  | بہت غلط اور چبل بندہ ہے بہن چود وہ یہ الگ بات ہے کہ حجاب اچھی ہے بہت | 1 |  |  |  |  |  |  |  |  |  |  |
|  | بہت فارغ اخیر چوتیاپا تھا | 1 |  |  |  |  |  |  |  |  |  |  |
|  | بہت کمینہ پن دیکھایا ہے اس گانڈو نے | 1 |  |  |  |  |  |  |  |  |  |  |
|  | بہت کیری بھائی تم بہت عظیم ہو | 0 |  |  |  |  |  |  |  |  |  |  |
|  | بہت گندی کر دی اس کی دکی بھائی لو یو مزہ آگیا | 1 |  |  |  |  |  |  |  |  |  |  |
|  | بہت گندی کر دی یار تو نے اس گانڈو کی | 1 |  |  |  |  |  |  |  |  |  |  |
|  | بہت لمنے عرصے ک بعد اتنی اچھی ویڈیو دیکھنے کو ملی مجھے یقین ہے کہ تم کیمرے کی پیجھے بہت اچھا کام کرتا ہے؎ | 0 |  |  |  |  |  |  |  |  |  |  |
|  | بہت محبت بہت پیار آپ کے لیے بہت صیح گانڈ ماری آپ نے اس کی دکی بھائی | 1 |  |  |  |  |  |  |  |  |  |  |
|  | بہت مختصر ویڈیو تھی لیکن بہت اچھی تھی | 0 |  |  |  |  |  |  |  |  |  |  |
|  | بہت مست ویڈیو ہے آکر چاہے پی لو | 0 |  |  |  |  |  |  |  |  |  |  |
|  | بہت ہی اچھا حصہ تھا | 0 |  |  |  |  |  |  |  |  |  |  |
|  | بہت ہی اعلیٰ پاکستان سے | 0 |  |  |  |  |  |  |  |  |  |  |
|  | بہت ہی اعلی یار کینڈین چوتیے کی پیروی کنا بند کریں | 1 |  |  |  |  |  |  |  |  |  |  |
|  | بہت ہی چبل انسان ہے یے سالا چوتیا ابلیس | 1 |  |  |  |  |  |  |  |  |  |  |
|  | بہت ویڈیو شاندار بنائی آپ نے اور آپ کو سالگرہ مبارک ہو | 0 |  |  |  |  |  |  |  |  |  |  |
|  | بہترین بھائی واقعی اس نے صیح کہا عوام کو چوتیا بنایا ہوا ہے | 1 |  |  |  |  |  |  |  |  |  |  |
|  | بہترین وضاحت کی آپ نے جو آج تک کوئی بھی نہں کر سکا | 0 |  |  |  |  |  |  |  |  |  |  |
|  | بہن چود اپنی اوقات پتہ نہں ہے ابے تو سچ بول دے بھوسڑی کے | 1 |  |  |  |  |  |  |  |  |  |  |
|  | بہن چود اتنے ڈسلائیکس ابھی بھی اس ابلیس چوتیے کے فائن نہیں سمجھے | 1 |  |  |  |  |  |  |  |  |  |  |
|  | بہن چود اگر تم پاکستانیوں کو چوتیا کہتے ہو اور پھیر اپنے آپ کو پاکستانی بھی کہتے ہو | 1 |  |  |  |  |  |  |  |  |  |  |
|  | بہن چود بنال لگا لینا اس لیئے تم بھوسڑی کے پاکستانی بہن چود پنجاب کے بارے میں بات کرتے ہو | 1 |  |  |  |  |  |  |  |  |  |  |
|  | بہن چود چوتیا ہے شام گانڈو | 1 |  |  |  |  |  |  |  |  |  |  |
|  | بہن چود دکی بھائی بہت اعلی | 1 |  |  |  |  |  |  |  |  |  |  |
|  | بہن چود دلا کنجر دا پتر دلا تو | 1 |  |  |  |  |  |  |  |  |  |  |
|  | بہن چود سالا اس نے ایک اور چبل مار دی | 1 |  |  |  |  |  |  |  |  |  |  |
|  | بہن چود سالے جھوٹے ابلیس کی گانڈ مار دی تونے | 1 |  |  |  |  |  |  |  |  |  |  |
|  | بہن چود سالے کسی نے فراگی کے ساتھ بدتمیزی کی میں اس کی ماں بہن ایک کردوں گا | 1 |  |  |  |  |  |  |  |  |  |  |
|  | بہن چود عمران خان کو کچھ نہ کہے ورنہ تیری ماں چود لوں گا | 1 |  |  |  |  |  |  |  |  |  |  |
|  | بہن چود کی بائیک تو میں چلاتا ہوں چوتیے کی | 1 |  |  |  |  |  |  |  |  |  |  |
|  | بہن چود کی گانڈ پھاڑ دی سچھ میں نفرت ہوگئی ہے اس سے | 1 |  |  |  |  |  |  |  |  |  |  |
|  | بہن چود کیا ریپ کیا بھائی نے | 1 |  |  |  |  |  |  |  |  |  |  |
|  | بہن چود گانڈو کی کیا گانڈ ماری آپ نے بہن چود ساری زندگی یاد رکھے گا | 1 |  |  |  |  |  |  |  |  |  |  |
|  | بہن چود ماں کا لن بھوسڑی کا مادرچود | 1 |  |  |  |  |  |  |  |  |  |  |
|  | بہن چود مزاقیہ متفق ہوں آپ سب سے میں | 1 |  |  |  |  |  |  |  |  |  |  |
|  | بہن چود میں پہلے شام کا فائن تھا پر اب بہن چود کی گانڈ مار سالے کی | 1 |  |  |  |  |  |  |  |  |  |  |
|  | بہن چود نے لفظ بھی بین کیے ہوئے ہیں میں نے اس کی ویڈیوز پر لکھنا تھا | 1 |  |  |  |  |  |  |  |  |  |  |
|  | بہن چودی کا اصل مزہ آگیا | 1 |  |  |  |  |  |  |  |  |  |  |
|  | بہن چودیاں ذیادہ ماری ہوئی ہیں بھوسڑی کے والے نے | 1 |  |  |  |  |  |  |  |  |  |  |
|  | بہھت زیادہ ہو گیا دکی بھائی مان لیا کہ ادریس چوتیا ہے ٹھیک ہے پر اب یہ ویڈیو بنا کر تم میں اور اس میں اور اس میں کیا فرق رہ جائے گا | 1 |  |  |  |  |  |  |  |  |  |  |
|  | بھاگ چوتیے بھاگ سالے | 1 |  |  |  |  |  |  |  |  |  |  |
|  | بھاگ سالے چوتیے تمہیں پاکستان میں رہنے کا کوئی حق نہیں | 1 |  |  |  |  |  |  |  |  |  |  |
|  | بھائی اب میں سیٹھی کی گانڈ پھاڑوں گا میں | 1 |  |  |  |  |  |  |  |  |  |  |
|  | بھائی اپنے کام کو جاری رکھو ورنہ ان جیسے لوگ پاکستان کو پھدو لگاتے رہیں گے | 1 |  |  |  |  |  |  |  |  |  |  |
|  | بھائی اپنی سالگرہ مبارک ہو اپنی حفاظت کریں اللہ آپ کی مدد کرے | 0 |  |  |  |  |  |  |  |  |  |  |
|  | بھائی اپیل سے پہلے وہ بھاگنے پر تیار ہے اگر وہ بھاگ گئی تو کون زمہ دار ہے شرم کر چوتیا | 1 |  |  |  |  |  |  |  |  |  |  |
|  | بھائی اتنے مزے آرہے تھے اتنی جلدی ختم ہو گئی ویڈیو | 0 |  |  |  |  |  |  |  |  |  |  |
|  | بھائی اتنی دیر سے کیوں | 0 |  |  |  |  |  |  |  |  |  |  |
|  | بھائی اتنی لیٹ کیوں آتی ہیں تیری ویڈیوز | 0 |  |  |  |  |  |  |  |  |  |  |
|  | بھائی اتنی میری اوقات نہیں کہ میں کچھ بول سکوں پر اس موضوع پر بات کرنے کا شکریہ | 0 |  |  |  |  |  |  |  |  |  |  |
|  | بھائی اجے وکی پیڈیا کے صفحہ پر اپکی بے عزتی ہو گئی ہے ذرا پڑھ کر دیکھ | 0 |  |  |  |  |  |  |  |  |  |  |
|  | بھائی اختتام کرکر دیا ہم تمہارے ساتھ ہیں تھوڑا ہٹ دیکھیں گے ان چوتیوں کو پاکستان میں | 1 |  |  |  |  |  |  |  |  |  |  |
|  | بھائی اخیر کر دی آپ نے میں آپ کی شروع سے ویڈیوز دیکھ رہا ہوں آپ نے کبھی بھی شام چوتیا کو کچھ نہں کہا | 1 |  |  |  |  |  |  |  |  |  |  |
|  | بھائی ادریس اصلی ہے یا چوتیا ہے سالا | 1 |  |  |  |  |  |  |  |  |  |  |
|  | بھائی اس بار زیادہ ویڈیوز ڈال اور مجھے اپنے کمپیوٹر کی تفصیل بتا اور اپنے عینک کا نمبر بھی بتا دے | 0 |  |  |  |  |  |  |  |  |  |  |
|  | بھائی اس پر بنا دو یار فراز چائے پلاؤ | 0 |  |  |  |  |  |  |  |  |  |  |
|  | بھائی اس کا ایک اور حصہ ہے | 0 |  |  |  |  |  |  |  |  |  |  |
|  | بھائی اس کریٹو دلے کی سپیلنگ ٹھیک کر کے دلا بولو مثال کے طور پر او دلے او دلے او دلے | 1 |  |  |  |  |  |  |  |  |  |  |
|  | بھائی اس کو آگے بڑھاؤ | 0 |  |  |  |  |  |  |  |  |  |  |
|  | بھائی اس کو کمنٹ میں چوتیا لکھتے ہیں | 1 |  |  |  |  |  |  |  |  |  |  |
|  | بھائی اس کی دوسری ویڈیوآگئی ہے لہذا ہم دوسرے روسٹ کے لیے انتظار کر رہے ہیں جتنا جلدی ہو سکے پلیز | 0 |  |  |  |  |  |  |  |  |  |  |
|  | بھائی اس کی گدھے کی گانڈ جیسی شکل ہے دفعہ کر اور گانڈ مار اس کی اپنے کام پر توجہ دو | 1 |  |  |  |  |  |  |  |  |  |  |
|  | بھائی اس کی ویڈیو میں مادرچود لکھ کر دیکھو چلتا ہی نہں | 1 |  |  |  |  |  |  |  |  |  |  |
|  | بھائی اس ویڈیو کا ایکس ویڈیو بنا دے مجھ کو تو بہت مزہ آ ئے گا | 0 |  |  |  |  |  |  |  |  |  |  |
|  | بھائی اس ویڈیو کو دیکھنے کے بعد سب کو حق ہے تیرا چینل ان سبسکرائب کرنے کا تو روسٹ ہی کر بھائی | 0 |  |  |  |  |  |  |  |  |  |  |
|  | بھائی اس ویڈیو میں مزہ نہیں آیا لیکن کوشش اچھی تھی | 0 |  |  |  |  |  |  |  |  |  |  |
|  | بھائی اسی طرح کی ویڈیوز بناؤ اس والے میں اتنا مزہ نہں آیا | 0 |  |  |  |  |  |  |  |  |  |  |
|  | بھائی اگر ہم نہں کہیں گے تو تم پھیر بھی ڈال ہی دو گے | 0 |  |  |  |  |  |  |  |  |  |  |
|  | بھائی ان لوڑوں لوگ کو ان کے حال پر چھوڑ دو کوئی فائدہ نہیں | 1 |  |  |  |  |  |  |  |  |  |  |
|  | بھائی انتا غصہ ہاہاہا گانڈ مار دی شام کی | 1 |  |  |  |  |  |  |  |  |  |  |
|  | بھائی انتی گندی کر دی | 1 |  |  |  |  |  |  |  |  |  |  |
|  | بھائی اور ایک نیا چوتیاپا شروع کردیا اس گانڈو نے آج | 1 |  |  |  |  |  |  |  |  |  |  |
|  | بھائی اور تھوڑا زیادہ کردے جدابھائی میں بھر جاتا ہوں ہنس ہنس کر | 0 |  |  |  |  |  |  |  |  |  |  |
|  | بھائی اوقات میں رہو ذیادہ اچھا رہے گا نہیں تو کسی دن مل گیا نا غلطی سے پسے جاؤ گے | 0 |  |  |  |  |  |  |  |  |  |  |
|  | بھائی ایک اور کسی جگہ کا ٹور بنا دے | 0 |  |  |  |  |  |  |  |  |  |  |
|  | بھائی ایک بات بتاؤ کیا آپ پنجابی ہو | 0 |  |  |  |  |  |  |  |  |  |  |
|  | بھائی ایک بات بولوں بھائی میری ویڈیوز کو شیئر کر دیں پلیز | 0 |  |  |  |  |  |  |  |  |  |  |
|  | بھائی ایک بار ہیلو ہی کردو | 0 |  |  |  |  |  |  |  |  |  |  |
|  | بھائی ایک گجراتی ویڈیو ہے دنیا جلے تو جلے اس پر ایک ویڈیو بناؤ | 0 |  |  |  |  |  |  |  |  |  |  |
|  | بھائی ایک ہفتہ ہونے کو آگیا نئی ویڈیو کب ڈالو گے میں آپ کی نئی ویڈیو کا انتظار کر رہا ہوں | 0 |  |  |  |  |  |  |  |  |  |  |
|  | بھائی ایک ویڈیو گرل فرینڈ اور بوائے فرینڈ کے بارے میں بھی بنا | 0 |  |  |  |  |  |  |  |  |  |  |
|  | بھائی آپ بہت اچھا کام کر رہے ہو آپ ہی صرف ان کی بجا سکتے ہو بھوسڑی کے سستے اداکار | 1 |  |  |  |  |  |  |  |  |  |  |
|  | بھائی آپ رہنے دو تو پہلے والی کیری نہیں رہا مت بناؤ یار ویڈیوز اب تو سچ میں الٹی آگئی | 0 |  |  |  |  |  |  |  |  |  |  |
|  | بھائی آپ سعید اجمل جیسے لگتے ہو | 0 |  |  |  |  |  |  |  |  |  |  |
|  | بھائی آپ سکوٹر کو شوٹ کریں اگر آپ سمجھ گئے ہوں تو | 0 |  |  |  |  |  |  |  |  |  |  |
|  | بھائی آپ کہاں تھے | 0 |  |  |  |  |  |  |  |  |  |  |
|  | بھائی آپ کو ضرور دیکھنا چاہیے دیوانی تو دیوانی | 0 |  |  |  |  |  |  |  |  |  |  |
|  | بھائی آپ کے لیے گالیاں کتوں کی طرح بھونکنا آسان ہے اپنے چینل پر آپ کا چینل تو بس ایک سڑا ہوا چتیاپا ہے | 1 |  |  |  |  |  |  |  |  |  |  |
|  | بھائی آپ کے مجھے کھونے کا دکھ ہے | 0 |  |  |  |  |  |  |  |  |  |  |
|  | بھائی آپ کی ٹی شرٹ بھہت اچھی ہے | 0 |  |  |  |  |  |  |  |  |  |  |
|  | بھائی آپ گیم پلیئر ہو سر درد ہو جاتا ہے | 0 |  |  |  |  |  |  |  |  |  |  |
|  | بھائی آپ لائیو کیوں نہیں آرہے پبجی پر | 0 |  |  |  |  |  |  |  |  |  |  |
|  | بھائی آپ نے اس گشتی کے بچے کی گانڈ مار کی میرا مشن پورا کر دیا ہے زندہ باد | 1 |  |  |  |  |  |  |  |  |  |  |
|  | بھائی آپ نے پھاڑ دی اس کی | 0 |  |  |  |  |  |  |  |  |  |  |
|  | بھائی آپ نے لا محدود جنگ کا ٹرائلر دیکھا ہے | 0 |  |  |  |  |  |  |  |  |  |  |
|  | بھائی آج اچھی لی ہے تم نے اس گانڈو کی چوتیا لوڑو کہیں کا ٹوپے کے منہ والا | 1 |  |  |  |  |  |  |  |  |  |  |
|  | بھائی آج تو اس کو پورا ڈنڈا ڈال دیا اب وہ مزاق میں ڈنڈا نہں لے گا چوتیا ہے وہ چوتیا | 1 |  |  |  |  |  |  |  |  |  |  |
|  | بھائی آج کل اتنا گھناؤنا ویڈیو کیوں بناتے ہو | 0 |  |  |  |  |  |  |  |  |  |  |
|  | بھائی آج لائیو سٹریم کر لے | 0 |  |  |  |  |  |  |  |  |  |  |
|  | بھائی آخر کار ایک ماہ کے بعد ویڈیو ڈال دیا کرہم ٹویٹ ویڈیوز کو دیکھ کر ہی دنیا میں جیتے ہیں | 0 |  |  |  |  |  |  |  |  |  |  |
|  | بھائی آخر میں لباس والی ویڈیو مست ہے زیادہ ایڈیٹنگ نہ کیا کر | 0 |  |  |  |  |  |  |  |  |  |  |
|  | بھائی آخری گانا مست تھا کیا غضب لکھا آپ نے | 0 |  |  |  |  |  |  |  |  |  |  |
|  | بھائی آگ لگا دی آپ نے | 0 |  |  |  |  |  |  |  |  |  |  |
|  | بھائی آنکھیں ترس گئی تیرے دیکھے ہوئے ویڈیوز | 0 |  |  |  |  |  |  |  |  |  |  |
|  | بھائی بار بار ہماری بھی مزاحیہ ویڈیو دیکھ لو مزہ آجائے گا اچھی لگے تو سبسکرائب کرو | 0 |  |  |  |  |  |  |  |  |  |  |
|  | بھائی بگ باس پر بھی بناؤ | 0 |  |  |  |  |  |  |  |  |  |  |
|  | بھائی بلوپر ڈال زیادہ بہتر لگے گا | 0 |  |  |  |  |  |  |  |  |  |  |
|  | بھائی بہت اچھی لگی ویڈیو | 0 |  |  |  |  |  |  |  |  |  |  |
|  | بھائی بہت اعلیٰ آپ نے تو سخت لونڈوں کا سر اونچا کر دیا | 1 |  |  |  |  |  |  |  |  |  |  |
|  | بھائی بہت اعلیٰ قسم کی ویڈیو تھی کمال کردیا | 0 |  |  |  |  |  |  |  |  |  |  |
|  | بھائی بہت صیح لایئک کریں اگر آپ راضی ہوں | 0 |  |  |  |  |  |  |  |  |  |  |
|  | بھائی بہت صیح یار بلکل | 0 |  |  |  |  |  |  |  |  |  |  |
|  | بھائی بہت غصے میں لگتے ہو آج ہربن واقعی میں چوتیا ہے | 1 |  |  |  |  |  |  |  |  |  |  |
|  | بھائی بہت مشکل کام تھا لیکن بہت اچھا تھا | 0 |  |  |  |  |  |  |  |  |  |  |
|  | بھائی بھائی بالکل الگ نکال کے لیٹا ہے کیری بھی | 0 |  |  |  |  |  |  |  |  |  |  |
|  | بھائی پلیز روسٹ اشیش چھن چلانی | 0 |  |  |  |  |  |  |  |  |  |  |
|  | بھائی پہلے بتا رہا ہوں میرے لیے بھی بھائی ایک بنا دے | 0 |  |  |  |  |  |  |  |  |  |  |
|  | بھائی پھاڑ دی آپ نے اور میں شام چوتیے سے زیادہ فراگی کی عزت کرتا ہوں | 1 |  |  |  |  |  |  |  |  |  |  |
|  | بھائی پی کے کا پرینک ایک چینل ہے ذرا اس کی کلاس لو ویڈیو ضرور بناؤ | 0 |  |  |  |  |  |  |  |  |  |  |
|  | بھائی پیسے کتنے کما لیتے ہو | 0 |  |  |  |  |  |  |  |  |  |  |
|  | بھائی پیوالی کی ویڈیو بنا جلدی سے اس کی ماں کا شاکا لاکا بوم بوم | 1 |  |  |  |  |  |  |  |  |  |  |
|  | بھائی تم تھیک کہتے ہو یہ سالا ہے ہی بھوسڑی کا میں بولتا ہوں تیری بہن کتے کی بچی تیرا سارا خاندان مادرچود | 1 |  |  |  |  |  |  |  |  |  |  |
|  | بھائی تم سب سے اچھے ہو لیکن جلدی ویڈیو اپ لوڈ کیا کروں | 0 |  |  |  |  |  |  |  |  |  |  |
|  | بھائی تم عظیم ہو تمہاری سب ویڈیوز بہت اچھی ہوتی ہیں کیا تجھے لاکھوں لائیکس ملنی چاہیں | 0 |  |  |  |  |  |  |  |  |  |  |
|  | بھائی تم میرے بہت قریب ہو | 0 |  |  |  |  |  |  |  |  |  |  |
|  | بھائی تم نے اس کو بہت مست چیلنج کیا | 0 |  |  |  |  |  |  |  |  |  |  |
|  | بھائی تم نے محنت کی لیکن وہ لیول نہیں تھا جو ہر بار ہوتا ہے ویڈیو ختم ہوتے ہوتے پک گئیے ہم | 0 |  |  |  |  |  |  |  |  |  |  |
|  | بھائی تم ہمیشہ شاندار ہو | 0 |  |  |  |  |  |  |  |  |  |  |
|  | بھائی تھوڑا زیادہ ہوگیا | 0 |  |  |  |  |  |  |  |  |  |  |
|  | بھائی تھوڑی کم گالی بولو | 0 |  |  |  |  |  |  |  |  |  |  |
|  | بھائی تو خود چتیا پا ہے نفرت کرتے ہیں تو اچھے لگتے ہیں مگر میرے بھائی تم تو خود چوتیا ہو | 1 |  |  |  |  |  |  |  |  |  |  |
|  | بھائی تو سب ٹائٹلز کب سے دینے لگ گیا لیکن اچھا ان کے لیے جو ہندی نہیں سمجھتے | 0 |  |  |  |  |  |  |  |  |  |  |
|  | بھائی تو سنجیدہ تھا لیکن اس میں کوئی خاص مزہ نہیں آرہا تم اور دوسرے ان کو کھا گئے | 0 |  |  |  |  |  |  |  |  |  |  |
|  | بھائی تو کتوں کی طرح تپا ہوا ہے مار گانڈ ان بہن چودوں کی مت چھوڑ ان سالوں کو | 1 |  |  |  |  |  |  |  |  |  |  |
|  | بھائی تو کہاں ہے | 0 |  |  |  |  |  |  |  |  |  |  |
|  | بھائی تو مست ویڈیو بناتا تھا مجھے تم سے پیار ہے ویڈیو بھائی کا مست بنا تھا | 0 |  |  |  |  |  |  |  |  |  |  |
|  | بھائی تو مل مجھے ایک بار کہیں بھی بس مل مجھے باہر جہاں بھی ملے گا تو تجھے سلام دوں گا | 0 |  |  |  |  |  |  |  |  |  |  |
|  | بھائی تو نے بات صیح کی ہے اب یہ بندہ بہن یکی کا باجا بجائے گا ضرور | 1 |  |  |  |  |  |  |  |  |  |  |
|  | بھائی تو نے تو آخیر کر دی ویسے یہ بندہ ہے ہی چوتیا | 1 |  |  |  |  |  |  |  |  |  |  |
|  | بھائی تو نے تو گانڈ ہی مار لی اس دلے کی | 1 |  |  |  |  |  |  |  |  |  |  |
|  | بھائی تو نے روسٹ کر دیا یار | 0 |  |  |  |  |  |  |  |  |  |  |
|  | بھائی تو نے کیا کھا کر ویڈیو بنایا اور اس کی گانڈ کے پیچھے کیوں پڑا ہے اور آج کی یہ ویڈیو سپر تھی یار | 1 |  |  |  |  |  |  |  |  |  |  |
|  | بھائی تیرا بھائی ویڈیو ٹرینڈینگ میں ہے | 0 |  |  |  |  |  |  |  |  |  |  |
|  | بھائی تیرا کام منہ میں ڈالنا ہے اور گانڈ سے نکالنا ہے | 1 |  |  |  |  |  |  |  |  |  |  |
|  | بھائی تیرا لطیفہ تو سب سے اچھا تھا لیکن پلیز فٹ پاتھ والے لوگوں پر مزاق مت کیا کریں | 0 |  |  |  |  |  |  |  |  |  |  |
|  | بھائی تیرائی کا بھی ویڈیو روسٹ کر | 0 |  |  |  |  |  |  |  |  |  |  |
|  | بھائی تیری ویڈیو بہت پسند آئی سچ میں آخری کلپ میں بہت مزہ آیا | 0 |  |  |  |  |  |  |  |  |  |  |
|  | بھائی تیری ویڈیو بھی ٹرینڈینگ پر ہے تجھ کو کیسا محسوس ہورہا ہے | 0 |  |  |  |  |  |  |  |  |  |  |
|  | بھائی تیری ویڈیو کو پسند کیا مزۃ آگیا بھائی | 0 |  |  |  |  |  |  |  |  |  |  |
|  | بھائی ٹائم لے کر گانڈ ماری ہے لو انڈیا سے | 1 |  |  |  |  |  |  |  |  |  |  |
|  | بھائی ٹھرک مہتا کا الٹا چشمہ مہربانی | 0 |  |  |  |  |  |  |  |  |  |  |
|  | بھائی ٹیلنٹ ہے کہاں کی عزت کرے کوئی کی ایکسپریشن تھا | 0 |  |  |  |  |  |  |  |  |  |  |
|  | بھائی جان اس بار آپ نے بہن چود دی ہے اس کی | 1 |  |  |  |  |  |  |  |  |  |  |
|  | بھائی جان یہ کتا پھر کوئی ماں چودکی کا پارٹ کرے گا | 1 |  |  |  |  |  |  |  |  |  |  |
|  | بھائی جو کر سکتا ہے وہ کر نا مطلب تو گانا کیوں گا رہا ہے | 0 |  |  |  |  |  |  |  |  |  |  |
|  | بھائی جی شام کی گانڈ پھاڑ دی آپ نے | 1 |  |  |  |  |  |  |  |  |  |  |
|  | بھائی جی یہ تو بہت جلدی ٹرینڈ پر آگیا | 0 |  |  |  |  |  |  |  |  |  |  |
|  | بھائی چل مارو اس رنڈی کے بچے بہت ہیں اس دنیا میں جب ان کی ماں چودی گئی نہ یوٹیوب پر پھر یہ بیٹھ کر روئے گا | 1 |  |  |  |  |  |  |  |  |  |  |
|  | بھائی چلا تم رہے ہو گانڈ میری پھٹ رہی ہے | 1 |  |  |  |  |  |  |  |  |  |  |
|  | بھائی چھوڑ نا اب اس چوتیےکا پیچھا اپنی مزاحیہ ویڈیوز بناتا رہ | 1 |  |  |  |  |  |  |  |  |  |  |
|  | بھائی دلوں کو مارنے والے | 0 |  |  |  |  |  |  |  |  |  |  |
|  | بھائی دم ہے تو مجھےروسٹ کر کے دیکھا | 0 |  |  |  |  |  |  |  |  |  |  |
|  | بھائی دنچک پوجا کے نئے گانے کو بھی روسٹ کرو نا | 0 |  |  |  |  |  |  |  |  |  |  |
|  | بھائی دیپک کے پیچھے آپ کا ہاتھ ہے | 0 |  |  |  |  |  |  |  |  |  |  |
|  | بھائی ڈاؤن لوڈ کاآپشن کیوں نہں آرہا | 0 |  |  |  |  |  |  |  |  |  |  |
|  | بھائی ذاکر خان پر بھی ایک ویڈیو بناؤ | 0 |  |  |  |  |  |  |  |  |  |  |
|  | بھائی ریزیڈنٹ ایول کو لائیو کر نا | 0 |  |  |  |  |  |  |  |  |  |  |
|  | بھائی زوم ان والا مست ہے | 0 |  |  |  |  |  |  |  |  |  |  |
|  | بھائی سارہ خان اور راکھی ساونت کو بھی روسٹ کر ان دونوں کا ویڈیو ہے اس کو روسٹ کر بھائی | 0 |  |  |  |  |  |  |  |  |  |  |
|  | بھائی سب سے اچھی ویڈیو اس بہن چود کی کیوں کی کی اس کی گانڈ میں کیڑا ہے | 1 |  |  |  |  |  |  |  |  |  |  |
|  | بھائی سچ میں مزہ آگیا اور آخر میں جو گانا تم نے گایا ہے وہ آج سے میرا پسندیدہ گانا ہو گیا ہے | 0 |  |  |  |  |  |  |  |  |  |  |
|  | بھائی سنا اس کو ٹھیک طرح سے میں اس کی ویڈیوز دیکھتا تھا تھوڑی سی اب توہر ویڈیو پر ڈسلائیک دیتا ہوں چوتیا شام | 1 |  |  |  |  |  |  |  |  |  |  |
|  | بھائی سونی کے گانے کو روسٹ کر | 0 |  |  |  |  |  |  |  |  |  |  |
|  | بھائی سیلفی والا منظر بہت اچھا تھا | 0 |  |  |  |  |  |  |  |  |  |  |
|  | بھائی شاندار اس لڑکی کی واٹ لگا دی | 0 |  |  |  |  |  |  |  |  |  |  |
|  | بھائی شاندار بہت ہی شاندار | 0 |  |  |  |  |  |  |  |  |  |  |
|  | بھائی شاندار ویڈیو ہے لیکن پھر ڈرنک کر کے گر جانے والا منظر مجھےبالکل پسند نہیں آیا | 0 |  |  |  |  |  |  |  |  |  |  |
|  | بھائی شاید وہ جوہن سینا نہیں جوہنی سنز بول رہا ہے | 0 |  |  |  |  |  |  |  |  |  |  |
|  | بھائی صاحب سب سے بڑھیا ویڈیو ہے ابھی تک کی | 0 |  |  |  |  |  |  |  |  |  |  |
|  | بھائی صرف ایک ویڈیو کیوں ڈالتی ہو ہر مہینہ | 0 |  |  |  |  |  |  |  |  |  |  |
|  | بھائی صیح کیا تم نے جو اس چوتیا کے چہرے سے نقاب اتار دیا | 1 |  |  |  |  |  |  |  |  |  |  |
|  | بھائی صیح گندی کی ہے تم نے ان کتوں کی | 1 |  |  |  |  |  |  |  |  |  |  |
|  | بھائی عظیم ہو آپ حقیقت میں | 0 |  |  |  |  |  |  |  |  |  |  |
|  | بھائی غلط کرے گا یہ بہن چود | 1 |  |  |  |  |  |  |  |  |  |  |
|  | بھائی غور تو مار اس عورت پر | 0 |  |  |  |  |  |  |  |  |  |  |
|  | بھائی کا ویڈیو آگیا | 0 |  |  |  |  |  |  |  |  |  |  |
|  | بھائی کب سے انتظار تھا تیری ویڈیو کا | 0 |  |  |  |  |  |  |  |  |  |  |
|  | بھائی کچھ مزاحیہ نہیں ہے یار کیا بنا | 0 |  |  |  |  |  |  |  |  |  |  |
|  | بھائی کچھڑی بھائی کا خیال بہت مست ہے مگر ہو سکے تو ساتھ میں فیس کیم بھی کر نا مست لگتا ہے | 0 |  |  |  |  |  |  |  |  |  |  |
|  | بھائی کر دے اب لائیک اس کو | 0 |  |  |  |  |  |  |  |  |  |  |
|  | بھائی کڑک ویڈیو ہے | 0 |  |  |  |  |  |  |  |  |  |  |
|  | بھائی کس چوتیا کے بارے میں ویڈیو بنا رہے ہو | 1 |  |  |  |  |  |  |  |  |  |  |
|  | بھائی کسی اور زبان میں کمنٹس کیا کرو ان گانڈوؤں کو پتا چل جاتا ہے | 1 |  |  |  |  |  |  |  |  |  |  |
|  | بھائی کسی مشہور گانے پر ایک اچھی سی ویڈیو بناؤ | 0 |  |  |  |  |  |  |  |  |  |  |
|  | بھائی کمال کی ویڈیو تھی ان جیسے چوتیوں کےساتھ ایسا ہی ہونا چاہیے تھا مادر چود کے بچے نہ ہوں کہیں کے | 1 |  |  |  |  |  |  |  |  |  |  |
|  | بھائی کہانی مزہ کی تھی اور سینما سے لے کر فوٹوگرافی تک کی چیزیں | 0 |  |  |  |  |  |  |  |  |  |  |
|  | بھائی کو سونی کی ایمبیسی ڈر بننے پر مبارک ہو | 0 |  |  |  |  |  |  |  |  |  |  |
|  | بھائی کون سا گیم چل رہا ہے | 0 |  |  |  |  |  |  |  |  |  |  |
|  | بھائی کی مزاحیہ ویڈیوز بہت اچھی ہیں | 0 |  |  |  |  |  |  |  |  |  |  |
|  | بھائی کیا تم میرے سبسکرائبر بڑھانے میں میری مدد کر سکتے ہو مجھے آپ کی شہرت کی ضرورت ہے | 0 |  |  |  |  |  |  |  |  |  |  |
|  | بھائی کیا چیز ہے تو میرے بھائی بہت مست ویڈیو یار پروڈکشن ہاؤس کھول لے قسم سے تجھے کہ رہا ہوں | 0 |  |  |  |  |  |  |  |  |  |  |
|  | بھائی کیا ہم بنگلہ دیش میں آپ کی چیزیں خرید سکتے ہیں | 0 |  |  |  |  |  |  |  |  |  |  |
|  | بھائی کیری آپ بہت کام کر رہے ہو بس اسی کو آگے جاری رکھو | 0 |  |  |  |  |  |  |  |  |  |  |
|  | بھائی کیری کا ہو گیا تجھے تو ویڈیو ہی نہیں آرہی تیری ڈینگی تو نہیں لے کر بیٹھ گیا بہتر ہوجا جلدی | 0 |  |  |  |  |  |  |  |  |  |  |
|  | بھائی کیری کیا ہم گیتا کھیل سکتے ہیں | 0 |  |  |  |  |  |  |  |  |  |  |
|  | بھائی کیری گانڈ پھاڑ دی واقعی مزہ آگیا | 1 |  |  |  |  |  |  |  |  |  |  |
|  | بھائی کیری نومان خان کے جواب پر ردعمل دو | 0 |  |  |  |  |  |  |  |  |  |  |
|  | بھائی کیش آف کلین کھیلیں | 0 |  |  |  |  |  |  |  |  |  |  |
|  | بھائی کیوں اس کی اتنی ٹھوکتا رہتا ہے ویسے مجھے اس کا شادی کرلے والا ڈائیلاگ انتہائی چوتیا لگتا ہے | 1 |  |  |  |  |  |  |  |  |  |  |
|  | بھائی کیوں آپ اس چوتیا کے لیے اپنی ویڈیو کی کوالٹی خراب کر رہے ہو | 1 |  |  |  |  |  |  |  |  |  |  |
|  | بھائی کیوں بغیر کسی بات کے بہن چودی والی بات کرتے ہو اچھی اچھی باتیں کرو پلیز | 1 |  |  |  |  |  |  |  |  |  |  |
|  | بھائی گانڈ مار دی سچ میں تونے اس کی | 1 |  |  |  |  |  |  |  |  |  |  |
|  | بھائی گانڈ میں ڈنڈا دے والا پورا گانا اپ لوڈ کرو | 1 |  |  |  |  |  |  |  |  |  |  |
|  | بھائی گانڈ ہی مار دی آپ نے تو بہن چود کی ہاہاہا وہ اس چیز کا مستحق تھا | 1 |  |  |  |  |  |  |  |  |  |  |
|  | بھائی گھس گیا تو اس کی گانڈ میں پورا پورا | 1 |  |  |  |  |  |  |  |  |  |  |
|  | بھائی گیتا والا حصہ | 0 |  |  |  |  |  |  |  |  |  |  |
|  | بھائی گیتا والا سین دیکھ کر آنکھوں میں آنسو آگئے کیا کام دیکھایا بھائی مزیدار ویڈیو | 0 |  |  |  |  |  |  |  |  |  |  |
|  | بھائی گیم چل رہی ہے اور بہت بہترین ہے | 0 |  |  |  |  |  |  |  |  |  |  |
|  | بھائی گیم کا نام کیا ہےبہت ہی اچھا ہے بولو کیا نام ہے | 0 |  |  |  |  |  |  |  |  |  |  |
|  | بھائی لاکھ ہو گیا | 0 |  |  |  |  |  |  |  |  |  |  |
|  | بھائی لنک کی ویڈیو دیکھیں اور اس پر بھی ویڈیو بناؤ | 0 |  |  |  |  |  |  |  |  |  |  |
|  | بھائی لو یو سٹرابری والا پارٹ عمدہ تھا | 0 |  |  |  |  |  |  |  |  |  |  |
|  | بھائی مارکیٹ میں آگیا کیا | 0 |  |  |  |  |  |  |  |  |  |  |
|  | بھائی مبارک ہو پہلا چکن ڈنر کے لیے | 0 |  |  |  |  |  |  |  |  |  |  |
|  | بھائی مت کر بہت بےکار تھا یہ سب | 0 |  |  |  |  |  |  |  |  |  |  |
|  | بھائی مجھے آپ کا ردعمل اچھا لگا تبدیلیوں کا جواب نہیں اسی وجہ سے بول رہا بھائی میں نے تو اپنا سر پھوڑ دیا | 0 |  |  |  |  |  |  |  |  |  |  |
|  | بھائی مناتی اپنا فیس کیم انتا مت ڈال ویلیو کم ہو جائے گی تیرے فیس کی تو گیم پلے ہی روسٹ کیا کر | 0 |  |  |  |  |  |  |  |  |  |  |
|  | بھائی مہربانی کر کے اس کمزور بیٹسمین کی خراب ویڈیو کی مشق دیکھو | 0 |  |  |  |  |  |  |  |  |  |  |
|  | بھائی مہربانی کر کے اس کو بھی دیکھیں | 0 |  |  |  |  |  |  |  |  |  |  |
|  | بھائی مہربانی کر کے پلو آنٹی پر بھی ایک ویڈیو بناؤ | 0 |  |  |  |  |  |  |  |  |  |  |
|  | بھائی مہربانی کر کے ملائکہ پہ بھی ویڈیو بناؤ سنجیدگی سے آپ کی توجہ اس موضوع پہ چائہیے | 0 |  |  |  |  |  |  |  |  |  |  |
|  | بھائی میں امتحان میں فیل ہو جاؤں گا کیوں دونوں ابھی ویڈیوز اپ لوڈ کر رہے ہیں | 0 |  |  |  |  |  |  |  |  |  |  |
|  | بھائی میں انڈیا سے ہوں اور مجھے اس سے کوئی فرق نہں پڑتا کہ آپ دونوں کا آپس میں مسئلہ ہے پر چوتیا چوتیا ہی رہے گا | 1 |  |  |  |  |  |  |  |  |  |  |
|  | بھائی میں انڈیا سے ہوں میں آپ کی ساری ویڈیوز پسند کرتا ہوں آپ اس کو چھوڑ کر اور ویڈیو بناؤ سب جانتے ہیں کہ وہ گانڈو چوتیا ہے | 1 |  |  |  |  |  |  |  |  |  |  |
|  | بھائی میں آپ کا سب سے بڑا فائن ہوں تو کیسے ہو آپ لوگ مہربانی کر کے ہٹی اور چٹی لوگوں پر بھی ایک ویڈیو بنائیں | 0 |  |  |  |  |  |  |  |  |  |  |
|  | بھائی میں بنگلہ دیش سے ہوں اور میں آپ کا بہت بڑا فائن ہوں | 0 |  |  |  |  |  |  |  |  |  |  |
|  | بھائی میں بھی سال کا ہوں لیکن بات یہ ہے کہ آپ اور آپ کی ویڈیوز بہت اچھے ہیں | 0 |  |  |  |  |  |  |  |  |  |  |
|  | بھائی میں تو بھی کر سکتا ہوں لیکن صرف ہی کر سکتا ہوں | 0 |  |  |  |  |  |  |  |  |  |  |
|  | بھائی میں تو کہتا ہوں کی ان کی داڑھی میں نہں ان کی گانڈ میں تنکا ہے | 1 |  |  |  |  |  |  |  |  |  |  |
|  | بھائی میں تو ہندی میں | 0 |  |  |  |  |  |  |  |  |  |  |
|  | بھائی میں تیری بہن یا لڑکی کو اگر چوم لوں گا تو کیسا ہو گا | 1 |  |  |  |  |  |  |  |  |  |  |
|  | بھائی میں خوش ہوں کہ وہ خاص قسم کا ڈریس تم نے مجھے گفٹ کیا | 0 |  |  |  |  |  |  |  |  |  |  |
|  | بھائی میں کلکتہ سے ہوں اور میں اسے جانتا ہوں ایک دوست جو گیا ہے اس کا دوست ہے | 0 |  |  |  |  |  |  |  |  |  |  |
|  | بھائی میں نے آپ کی اس سے بڑیا ویڈیو آج تک نہں دیکھی گئی پلیز اگلی ویڈیو کو جلدی ریلیز کردیں پلیز | 0 |  |  |  |  |  |  |  |  |  |  |
|  | بھائی میں نے صرف مشورہ دیا ہے تم کیوں مجھے گالی نکال رہے ہو | 0 |  |  |  |  |  |  |  |  |  |  |
|  | بھائی نو ایڈیٹنگ کی ہے باقی بندہ تو بہن چود ہے | 1 |  |  |  |  |  |  |  |  |  |  |
|  | بھائی نے پیچھے سے بیڈ ہٹا لیا | 0 |  |  |  |  |  |  |  |  |  |  |
|  | بھائی نے تو شام کی تیل لگا کر گانڈ لے لی | 1 |  |  |  |  |  |  |  |  |  |  |
|  | بھائی ہم آپ سے پیار کرتے ہیں آپ چھوڑو نا اس لوڑو کوبےکار میں سالا پاگل کا بچہ ہے | 1 |  |  |  |  |  |  |  |  |  |  |
|  | بھائی ہم جا رہے ہیں مالے سے کڑنے | 0 |  |  |  |  |  |  |  |  |  |  |
|  | بھائی ہم سب تیرے ساتھ ہیں یہ سب سے بڑا چوتیا ہے برگر کا | 1 |  |  |  |  |  |  |  |  |  |  |
|  | بھائی ہمارا سنہری پلےبٹن کہاں ہے | 0 |  |  |  |  |  |  |  |  |  |  |
|  | بھائی ہماری بھی فنی ویڈیوز دیکھو مہربانی کے بار بار دیکھو پر جانے کے لئیے پروفائل تصویر پر کلک کریں | 0 |  |  |  |  |  |  |  |  |  |  |
|  | بھائی ہمیں کچھ نہیں ہوا تو ہٹا بس اور سن جائے مٹا دی | 0 |  |  |  |  |  |  |  |  |  |  |
|  | بھائی ہنس ہنس کے برا حال ہو گیا | 0 |  |  |  |  |  |  |  |  |  |  |
|  | بھائی ہے تو سیکسی بہت ہے رنڈی | 1 |  |  |  |  |  |  |  |  |  |  |
|  | بھائی واقعی ننگی ویڈیوز دیکھا رہا تھا نوٹیفیکشن آتے ہی آگیا | 0 |  |  |  |  |  |  |  |  |  |  |
|  | بھائی وقار ذکاء خود بہت ذہین ہے | 0 |  |  |  |  |  |  |  |  |  |  |
|  | بھائی وہ سلمان والا گانا تھوڑا اچھا تھا | 0 |  |  |  |  |  |  |  |  |  |  |
|  | بھائی ویڈیو بہت وقت کے بعد نکالا کوئی کام ہو گیا تھا کیا | 0 |  |  |  |  |  |  |  |  |  |  |
|  | بھائی ویڈیو جلدی اپ لوڈ کیا کر ہمیں آپ کی ویڈیو کا ہی انتظار ہوتا ہے ویسے وہ گانڈو ہے ہی چوتیا | 1 |  |  |  |  |  |  |  |  |  |  |
|  | بھائی ویڈیوز دےدے پیسے بےشک نہ دے | 0 |  |  |  |  |  |  |  |  |  |  |
|  | بھائی یار کیا کر رہے ہو آج کل تو بالکل ہی ہنسی نہیں آرہی | 0 |  |  |  |  |  |  |  |  |  |  |
|  | بھائی یہ اچھا نہیں ہے وہ بہت بڑی مسائل سے گزر کر آیا ہے آپ اس پر اس کو منفی روشنی ڈالیں | 0 |  |  |  |  |  |  |  |  |  |  |
|  | بھائی یہ اوم پرکاش سے لیا تھا صیح ہو تو کمنٹ کرو | 0 |  |  |  |  |  |  |  |  |  |  |
|  | بھائی یہ اوم والی آواز ابھی بھی دماغ میں گھوم رہی ہے | 0 |  |  |  |  |  |  |  |  |  |  |
|  | بھائی یہ آپ کی سب سے اچھی ویڈیو تھی آپ اور آپ کے فائن شاندار ہیں | 0 |  |  |  |  |  |  |  |  |  |  |
|  | بھائی یہ آج تک بنایا ہوا کا سب سے اچھا ویڈیو ہے | 0 |  |  |  |  |  |  |  |  |  |  |
|  | بھائی یہ بہت زیادہ ہوگیا | 0 |  |  |  |  |  |  |  |  |  |  |
|  | بھائی یہ مادر چود ہے شام ادریس | 1 |  |  |  |  |  |  |  |  |  |  |
|  | بھائی یہ میری بات سن اور پوپی کی مار ذرا | 1 |  |  |  |  |  |  |  |  |  |  |
|  | بھائیوں اس کی ہر ویڈیو کے نیچے کمنٹ میں جا کر لکھو کہ باہر جا کر اپنی اوقات ہی بھول گیا ہے لوڑا | 1 |  |  |  |  |  |  |  |  |  |  |
|  | بھڑوا رنڈی کی نسل تیری گانڈ میں کیوں آگ لگی ہوئی ہے | 1 |  |  |  |  |  |  |  |  |  |  |
|  | بھڑوے چوتیے جا دفعہ ہو | 1 |  |  |  |  |  |  |  |  |  |  |
|  | بھڑوے کے بچےٹھنڈی ویلاگ نہ بنایا کر | 1 |  |  |  |  |  |  |  |  |  |  |
|  | بھلا بہن چود تیری اتنی کیوں کھجلتی کیوں ہے گانڈ | 1 |  |  |  |  |  |  |  |  |  |  |
|  | بھوسڑی کا سالا یہ ہے ہی ہیرا منڈی کا پیدائیشی | 1 |  |  |  |  |  |  |  |  |  |  |
|  | بھوسڑی کا ہے یہ شام اسے قدر نہیں ہے کی یہ کمنٹس کہاں سے آتی ہیں | 1 |  |  |  |  |  |  |  |  |  |  |
|  | بھوسڑی کے اب تو بتا میرے کمنٹس کدھر گئے گانڈو تیرے پاس ایک کیڑا ہے اور کچھ نہں ہے بہن چود | 1 |  |  |  |  |  |  |  |  |  |  |
|  | بھوسڑی کے اس طرح کی چیزیں انٹرنیٹ پر لکھے گا تو لوگ تجھے بولیں گے نا تو کیا تیری سالے کی تعریف کریں گے کیا | 1 |  |  |  |  |  |  |  |  |  |  |
|  | بھوسڑی کے انہوں نے جو بھی کیا صیح کیا تیری ماں کو چود کے رکھ دوں | 1 |  |  |  |  |  |  |  |  |  |  |
|  | بھوسڑی کے چپ کر نئی ویڈیو بھی آئیں گے | 1 |  |  |  |  |  |  |  |  |  |  |
|  | بھوسڑی کے سارے ویورز پاکستانی ہیں جن کو یہ غریب اور گھٹیا کہ رہا ہے | 1 |  |  |  |  |  |  |  |  |  |  |
|  | بھوسڑی کے کیوں کسی کے پیچھے پڑا ہوا ہے چول | 1 |  |  |  |  |  |  |  |  |  |  |
|  | بھوسڑی کے مادر چود بہن کے لوڑے کتے کے لنڈ ٹٹو کے سوداگر | 1 |  |  |  |  |  |  |  |  |  |  |
|  | بھوسڑی کی اولاد میرا ویڈیو صرف میرے چینل میں دیکھ | 1 |  |  |  |  |  |  |  |  |  |  |
|  | بولو جے جےجے کیری بابا کی | 0 |  |  |  |  |  |  |  |  |  |  |
|  | بے غیرت بے حیا صیح جواب دیا الماس کی بہن چودی کا | 1 |  |  |  |  |  |  |  |  |  |  |
|  | بی بی والے کا باپ ہے تو کیری وہ تیرے سامنے کچھ بھی نہیں تم اس سے بہت بہتر ہو | 0 |  |  |  |  |  |  |  |  |  |  |
|  | بیٹے والے تو ہم بھی ہیں لیکن اپنے تو انت اٹھائی ہوئی ہے | 0 |  |  |  |  |  |  |  |  |  |  |
|  | پاکستان کا پورن سٹار سب ایسے ہوتے جا رہے ہیں | 0 |  |  |  |  |  |  |  |  |  |  |
|  | پاکستان کی امی کی چوت پاکستان بھوسڑیستان ایک بار اپنی اس والی آئی ڈی سے لکھ دے کمال ہو جائے گا | 1 |  |  |  |  |  |  |  |  |  |  |
|  | پاکستان کی امی کی چوت پاکستان بھوسڑیستان کیا ٹوائلٹ تو انڈیا میں نہں رہتا کیا | 1 |  |  |  |  |  |  |  |  |  |  |
|  | پاکستانی چوتیا | 1 |  |  |  |  |  |  |  |  |  |  |
|  | پاکستانی چوتیا سالا سب سے بڑا | 1 |  |  |  |  |  |  |  |  |  |  |
|  | پاگل چپ کر جھوٹا چوتیا | 1 |  |  |  |  |  |  |  |  |  |  |
|  | پاگل زندگی بھائی میں نے | 0 |  |  |  |  |  |  |  |  |  |  |
|  | پاگل کر دیا بھائی تو نے عروج تو بہت شاندار تھا | 0 |  |  |  |  |  |  |  |  |  |  |
|  | پاگل لڑکی ہے گانا تو آتا نہیں چلی گانے | 0 |  |  |  |  |  |  |  |  |  |  |
|  | پاگل ہے یہ پاگل گانڈو سالا | 1 |  |  |  |  |  |  |  |  |  |  |
|  | پپو تم کو کس وقت آنا چاہیے تھا | 0 |  |  |  |  |  |  |  |  |  |  |
|  | پتا نہں پاکستانی عوام اس چوتیا پن پر خاموش کیوں ہے | 1 |  |  |  |  |  |  |  |  |  |  |
|  | پرانی طرز کی نہیں بہت جدید طرح کی ہونی چاہئے | 0 |  |  |  |  |  |  |  |  |  |  |
|  | پرانی ویڈیو میں کیلے لگتے ہیں لیکن لگے ہوئے سنترے ہیں | 0 |  |  |  |  |  |  |  |  |  |  |
|  | پراوین سنگھ سچ بولو تو تم ڈیپی کی بات مت کیا کرو | 0 |  |  |  |  |  |  |  |  |  |  |
|  | پریتم بھائی ٹھیک ہے کہ اچھی ویڈیو نہیں ہے لیکن کچھ اچھا دیکھنے کو تو ملا نا | 0 |  |  |  |  |  |  |  |  |  |  |
|  | پریہا تھاپا پلیز لڑکی کو امپریس کرنے کی میری مزاحیہ ویڈیو دیکھو اور اگر پسند آئے تو میرا چینل سبسکرائب کرو آپ کی مستی میرا چینل ہے | 0 |  |  |  |  |  |  |  |  |  |  |
|  | پشاور گھمائیں گے بہن چود تچھے کیسے کنفرم ہوا کہ وہ دلا ہے | 1 |  |  |  |  |  |  |  |  |  |  |
|  | پلیز اس کی بینڈ بجاؤ | 0 |  |  |  |  |  |  |  |  |  |  |
|  | پلیز آپ گوواتی آئیں ہم سب آپ سے ملنے کا انتظار کر رہے ہیں | 0 |  |  |  |  |  |  |  |  |  |  |
|  | پلیز بھائی اس کو روسٹ مت کرو کیوں کو وہ ہے کی کمینہ اگر تم اس کو اور روسٹ کرو گے تو وہ اور چوڑا ہو گا | 1 |  |  |  |  |  |  |  |  |  |  |
|  | پلیز بھائی ٹی سیریز کی مدد کرنے کا کہو | 0 |  |  |  |  |  |  |  |  |  |  |
|  | پلیز بھائی دنچک پوجا کی نئی ویڈیو پر ایک کلپ بنتا ہے پلیز پلیز | 0 |  |  |  |  |  |  |  |  |  |  |
|  | پلیز پیوڈپائی اور ٹی سیریز کی ویڈیو بناؤ | 0 |  |  |  |  |  |  |  |  |  |  |
|  | پلیز شام ادریس کو روسٹ کر نا یار | 0 |  |  |  |  |  |  |  |  |  |  |
|  | پلیز کچھ منظر کے پیچھے کیا ہو رہا ہے | 0 |  |  |  |  |  |  |  |  |  |  |
|  | پلیز میڈلے شٹ ویڈیو دیکھیں تم اس کو پسند کریں گے | 0 |  |  |  |  |  |  |  |  |  |  |
|  | پلیز میری نئے اکاؤنٹ سے نئی ویڈیو دیکھیں | 0 |  |  |  |  |  |  |  |  |  |  |
|  | پنوراما کا اشتہار کس کس کو آیا ٹھوکو لائیک | 0 |  |  |  |  |  |  |  |  |  |  |
|  | پہلے اس کی گانڈ میں ڈںڈا دے اور پھر اسکی بہن کو چود | 1 |  |  |  |  |  |  |  |  |  |  |
|  | پہلے اس کی گانڈ میں ڈنڈادو اور پھر اس پر جھنڈا لگاؤ تا کہ اس کو پتا چلے کہ اس پرچم میں کتنا دم ہے | 1 |  |  |  |  |  |  |  |  |  |  |
|  | پہلے مجھے کھسرا لگتا تھا پر اب یقین ہو گیا کہ وہ چوتیا ہے | 1 |  |  |  |  |  |  |  |  |  |  |
|  | پہلے میں سکون سے دیکھ سکتا ہوں | 0 |  |  |  |  |  |  |  |  |  |  |
|  | پہلی بار میں نے ویڈیو کو ناپسند کیا دن بہ دن ویڈیوز خراب ہوتی جا رہی ہیں | 0 |  |  |  |  |  |  |  |  |  |  |
|  | پہلی بارکیری طرح سے | 0 |  |  |  |  |  |  |  |  |  |  |
|  | پہلی والی بات نہیں رہی اب بھائی | 0 |  |  |  |  |  |  |  |  |  |  |
|  | پھر سے آپ کا فائن ہوگیا ہوں | 0 |  |  |  |  |  |  |  |  |  |  |
|  | پوجا کا کچھ کرو بھائی | 0 |  |  |  |  |  |  |  |  |  |  |
|  | پوڈکاسٹ پر ویڈیو بناؤ اور ٹی سیریز پر بھی | 0 |  |  |  |  |  |  |  |  |  |  |
|  | پورا کریمیناتی واپس آگیا | 0 |  |  |  |  |  |  |  |  |  |  |
|  | پوری دنیا ختم ہو جائے گی | 0 |  |  |  |  |  |  |  |  |  |  |
|  | پوری دنیا گھما کر مزاحیہ ویڈیو اپ لوڈ کرتا ہوں کیا آپ میرے چینل کو پسند کریں گے | 0 |  |  |  |  |  |  |  |  |  |  |
|  | پوری دنیا میں تیری ماں کی چوت | 1 |  |  |  |  |  |  |  |  |  |  |
|  | پوری ویڈیو میں سب سے اچھا حصہ | 0 |  |  |  |  |  |  |  |  |  |  |
|  | پولیس کی پٹائی اور | 0 |  |  |  |  |  |  |  |  |  |  |
|  | پولیس کی پٹائی اور رات کی چوہی کوئی نہیں روک سکتا | 0 |  |  |  |  |  |  |  |  |  |  |
|  | پونیت گجر پلیز میرا چینل سبسکرائب کرو میں نیا یوٹیوبر ہوں | 0 |  |  |  |  |  |  |  |  |  |  |
|  | پیار ہو گیا تم سے منچلے لویو ڈوڈ شاندار | 0 |  |  |  |  |  |  |  |  |  |  |
|  | پیار ہے تم سے میں تمہارا بہت بڑا فائن ہوں | 0 |  |  |  |  |  |  |  |  |  |  |
|  | پیارے دکی بھائی کو ایک چومی اور بھوسڑی کے شام سالے کے لیے ایک چوپا ادھر ایک چوپا ادھر | 1 |  |  |  |  |  |  |  |  |  |  |
|  | پیارے کیری ایک ویڈیو روسٹ کرنے کے لیے تمہں ہر طرح کے ویڈیوز دیکھنے پڑتےہیں | 0 |  |  |  |  |  |  |  |  |  |  |
|  | پیپ والی کاویڈیو بھی بنادیا ھوتا چائے پی لو دوستوں ویڈیو بہت زیادہ مست تھی | 0 |  |  |  |  |  |  |  |  |  |  |
|  | پیچھے چپل پڑی ہے | 0 |  |  |  |  |  |  |  |  |  |  |
|  | پیچھےمیوزک کون سا ہے | 0 |  |  |  |  |  |  |  |  |  |  |
|  | پینٹ پھٹی پڑی ہے | 0 |  |  |  |  |  |  |  |  |  |  |
|  | پینڈی پئے اور ہا ہا کو بھی گھر کیری بھائی | 0 |  |  |  |  |  |  |  |  |  |  |
|  | تجھ کو ان سبسکرائب کر دیا | 0 |  |  |  |  |  |  |  |  |  |  |
|  | تجھے مشہور ہونے کےلیے گانڈ بھی مارنی پڑے تو تو وہ بھی مارےگا | 1 |  |  |  |  |  |  |  |  |  |  |
|  | تچھے مسئلہ ہے تجھے اس نے کیا کہا ہے چوتیے چلو اگر مان لیتے ہیں یہ جھوٹی ویڈیو ہے تو پھرکیا | 1 |  |  |  |  |  |  |  |  |  |  |
|  | تعاف نے سب بہن چودوں کی گانڈ جلا دی | 1 |  |  |  |  |  |  |  |  |  |  |
|  | تلاش کرو شیام کلب چینل مزہ آجائے گا ایک بار تلاش کر کے تو دیکھو سبسکرائب کیے بناء نہیں آؤگے دیکھنا | 0 |  |  |  |  |  |  |  |  |  |  |
|  | تم ایک چوتیا ہو | 1 |  |  |  |  |  |  |  |  |  |  |
|  | تم بالکل ٹیھک درست ہو | 0 |  |  |  |  |  |  |  |  |  |  |
|  | تم بہت اچھے اور حیران ہو | 0 |  |  |  |  |  |  |  |  |  |  |
|  | تم بہت حیران کن ہو بھائی تم سب سے اعلی ہو | 0 |  |  |  |  |  |  |  |  |  |  |
|  | تم بھی ادھر ایسا کر سکتے ہو | 0 |  |  |  |  |  |  |  |  |  |  |
|  | تم بھی رہو بھائی | 0 |  |  |  |  |  |  |  |  |  |  |
|  | تم پریشان مت ہو میری جان وہ تو ہے ہی کتے کا بچہ دلے کا بچہ | 1 |  |  |  |  |  |  |  |  |  |  |
|  | تم ٹھیک ہو پر پھر بھی ہنسا لیتا ہے اپنی حرکتوں کی وجہ سے لیکن یہ جوتیا شام چوتیا ہی رہنا ہے | 1 |  |  |  |  |  |  |  |  |  |  |
|  | تم جیسے چوتیوں کی وجہ سے سنگھ جیسے لوگوں کی چوتیاں کاٹتے ہیں | 1 |  |  |  |  |  |  |  |  |  |  |
|  | تم چھوٹے یوٹیوبر ہو اس لیئے بڑے یو ٹیوبر سے جلتے ہو گالی مت دو چوتیو | 1 |  |  |  |  |  |  |  |  |  |  |
|  | تم چوتیا ہو ایک | 1 |  |  |  |  |  |  |  |  |  |  |
|  | تم دوبارہ سب پر نمبر لے گئے کیری | 0 |  |  |  |  |  |  |  |  |  |  |
|  | تم سب رنڈٰ کے بچے چوتیا ہو مادر چود ہو کتنے اچھے سے میں نے تمہاری امی کے یار کو بے نقاب کیا | 1 |  |  |  |  |  |  |  |  |  |  |
|  | تم سب سے بڑے چوتیے ہو اپنا کام کررہا ہے تو اپنا کام کر پیسے بنا اور اپنی گانڈ نہ مروا | 1 |  |  |  |  |  |  |  |  |  |  |
|  | تم سچھی بہن چودی میں فراڈ کر لیتا ہے | 1 |  |  |  |  |  |  |  |  |  |  |
|  | تم شام کے پیچھے کیوں پڑ گئے ہو چتو آدمی | 1 |  |  |  |  |  |  |  |  |  |  |
|  | تم صیح کھیل رہےہو | 0 |  |  |  |  |  |  |  |  |  |  |
|  | تم فیس کیم کیوں نہیں کرتے | 0 |  |  |  |  |  |  |  |  |  |  |
|  | تم کتے ہو گشتی ماں کے بچے بہن چود انسان تیری ماں کا پھدا | 1 |  |  |  |  |  |  |  |  |  |  |
|  | تم کہ رہے تھے ہر ہفتہ ڈالوں گا | 0 |  |  |  |  |  |  |  |  |  |  |
|  | تم کو ہمیشہ دیکھنا اچھا لگتا ہے کیری مناتی بھائی | 0 |  |  |  |  |  |  |  |  |  |  |
|  | تم مجھ کو جواب کیوں نہیں دے رہے | 0 |  |  |  |  |  |  |  |  |  |  |
|  | تم میں سے کتنے اپنی ہنسی نہیں روک سکتے | 0 |  |  |  |  |  |  |  |  |  |  |
|  | تم نے اپنا وعدہ پورا کیا نئی چیزیں بنانے کا بجائے کہ وہی پرانی چیزیں کو بار بار دکھانے کا | 0 |  |  |  |  |  |  |  |  |  |  |
|  | تم نے اس کو ختم کردیا مبارک ہو | 0 |  |  |  |  |  |  |  |  |  |  |
|  | تم نے اس کو صیح زخم دیا ہمیں تم جیسے ہو لوگ چاہیں نہ کا شام گانڈو جیسے | 1 |  |  |  |  |  |  |  |  |  |  |
|  | تم نے تو آگ لگا دی کمال کر دیےآپ نے | 0 |  |  |  |  |  |  |  |  |  |  |
|  | تم نے تو یار حد کردی چتیاپا سارا کھول کر رکھ دیا اس رنڈی کا | 1 |  |  |  |  |  |  |  |  |  |  |
|  | تم نے مجھے پوری ویڈیو ک بارے میں کیوں نہیں بتایا | 0 |  |  |  |  |  |  |  |  |  |  |
|  | تم نے مجھے جواب میں گدھا کہا ہے میں نے انٹرنیٹ پہلی بار استعمال کیا پر گالیوں میں میں تیری ماں ہوں | 1 |  |  |  |  |  |  |  |  |  |  |
|  | تم نے یہ کمنٹ سب ویڈیوز میں پوسٹ کر کے بہت برا کیا | 0 |  |  |  |  |  |  |  |  |  |  |
|  | تم نئے فائن ہو تو ادھر رجسٹر ہو جائیں | 0 |  |  |  |  |  |  |  |  |  |  |
|  | تم ہو تم روشنی ہو | 0 |  |  |  |  |  |  |  |  |  |  |
|  | تم یوٹیوب کے سب سے بڑے یوٹیوبر ہو | 0 |  |  |  |  |  |  |  |  |  |  |
|  | تمہارا روسٹ شاندار تھا بھائی | 0 |  |  |  |  |  |  |  |  |  |  |
|  | تمہارا نام کیا ہے کیری مناتی | 0 |  |  |  |  |  |  |  |  |  |  |
|  | تمہارے اندر وہ پہلی والی کیری کی بات ہی نہیں ہے بس ٹھیک ہے یہ ویڈیو بہت بری ہے | 0 |  |  |  |  |  |  |  |  |  |  |
|  | تمہارے پپو کے بارے میں بہت افسوس ہوا ہے | 0 |  |  |  |  |  |  |  |  |  |  |
|  | تمہارے مزاق نے تمہاری عزت ختم کردی ہے | 0 |  |  |  |  |  |  |  |  |  |  |
|  | تمہارے والدین آسانی سے تمہارے چوتڑ توڑ دیں گے | 1 |  |  |  |  |  |  |  |  |  |  |
|  | تمہاری ایڈیٹنگ اور شام کی بے عزتی چوتیا جھوٹا | 1 |  |  |  |  |  |  |  |  |  |  |
|  | تمہاری ایکٹنگ اچھی ہے تم فلموں میں اداکاری کیوں نہں کرتے | 0 |  |  |  |  |  |  |  |  |  |  |
|  | تمہاری غلط سوچ اور زبان آپ کو یہاں تک لے کر آئی ہے | 0 |  |  |  |  |  |  |  |  |  |  |
|  | تمہاری گانڈ میں کیڑا کاٹتا ہے کیا جو کل رات کو تم نےپھر ویڈیو بنائی | 1 |  |  |  |  |  |  |  |  |  |  |
|  | تمہاری موٹر سائیکل بہت شاندار ہے | 0 |  |  |  |  |  |  |  |  |  |  |
|  | تمہاری ویڈیو ہمیشہ سے ہی بہت مست ہوتی ہے | 0 |  |  |  |  |  |  |  |  |  |  |
|  | تمہاری ویڈیوز پسند آئیں ادھار کارڈ کی چینل کو سبسکرائب کرو | 0 |  |  |  |  |  |  |  |  |  |  |
|  | تہ آپ کی سب سے اچھی ویڈیو ہے میری خواہش ہے کہ ہم سب ادھر ہوتے | 0 |  |  |  |  |  |  |  |  |  |  |
|  | تھورا اور سوچےذرا | 0 |  |  |  |  |  |  |  |  |  |  |
|  | تھوڑا کم بولا کر کیری | 0 |  |  |  |  |  |  |  |  |  |  |
|  | تھوڑے لوگوں نے دیکھا ہے لیکن پسند بہت لوگوں نے کیا ہے یہ کیسے ممکن ہے | 0 |  |  |  |  |  |  |  |  |  |  |
|  | تو بہت بڑا چوتیا ہے شکر ہے اس چول کی ایک ویڈیو بھی نہیں دیکھی | 1 |  |  |  |  |  |  |  |  |  |  |
|  | تو بھڑوا ہے جھوٹا ہے گانڈو ہے | 1 |  |  |  |  |  |  |  |  |  |  |
|  | تو بھی چوتیا ہے اللہ تیرےکو گناہ دے | 1 |  |  |  |  |  |  |  |  |  |  |
|  | تو بول یا نہ بول میں بولا نا تو گانڈ میں گھسا لے یہ بات بہن چود | 1 |  |  |  |  |  |  |  |  |  |  |
|  | تو تھوڑا دکی کی گانڈ چاٹ لے | 1 |  |  |  |  |  |  |  |  |  |  |
|  | تو تو چوتیا ہے پکا پکا | 1 |  |  |  |  |  |  |  |  |  |  |
|  | تو جناب آپ کا حضرات مادرچود دلا کے علاوہ کس نے کروایا ہے | 1 |  |  |  |  |  |  |  |  |  |  |
|  | تو جو بار بار کمنٹ کر رہا ہے تجھے مرچیں کیوں لگ رہی ہیں شام کو باجی تو نہیں دی ہوئی جو اتنا اچھل رہا ہے | 1 |  |  |  |  |  |  |  |  |  |  |
|  | تو خود کیا ہے گالیاں تو سڑک کے کتے بھی بول لیتے ہیں ان کی ویڈیو بنا ٹرینڈ ملے گا | 0 |  |  |  |  |  |  |  |  |  |  |
|  | تو کیسے ہیں آپ سب | 0 |  |  |  |  |  |  |  |  |  |  |
|  | تو کیسے ہیں آپ کیری مناتی | 0 |  |  |  |  |  |  |  |  |  |  |
|  | تو کیوں ٹینشن لے رہا ہے چوتیا لوگ ہیں آپ مغز کیوں کھپا رہے ہو | 1 |  |  |  |  |  |  |  |  |  |  |
|  | تو گیم بہت بہن چود جیسا کھیلتا ہے | 1 |  |  |  |  |  |  |  |  |  |  |
|  | تو لن لے لے چوتیا | 1 |  |  |  |  |  |  |  |  |  |  |
|  | تو نے تو اس کی صیح پھاڑ دی کمینے لونڈے کی | 1 |  |  |  |  |  |  |  |  |  |  |
|  | تو نے تو گانڈ ہی مار دی | 1 |  |  |  |  |  |  |  |  |  |  |
|  | تو نےصیح گانڈ ماری اس بہن چود کی اچھے فراڈٰ کی اولاد ہے | 1 |  |  |  |  |  |  |  |  |  |  |
|  | تو ہم کیا کریں اس کی گانڈ میں للی چڑھائیں تاکہ اس کی یہ گھٹیا بیماری ختم ہو | 1 |  |  |  |  |  |  |  |  |  |  |
|  | تو یہ بولتا کیوں ہے | 0 |  |  |  |  |  |  |  |  |  |  |
|  | تو یہ ہے وہ گانڈو | 1 |  |  |  |  |  |  |  |  |  |  |
|  | توکر لےمیرے کوتو یہ ویڈیو اچھا لگا | 0 |  |  |  |  |  |  |  |  |  |  |
|  | تیرا کوئی قصور نہں تو گانڈو ہے بچے | 1 |  |  |  |  |  |  |  |  |  |  |
|  | تیرا مسئلہ کیا ہے بھائی | 0 |  |  |  |  |  |  |  |  |  |  |
|  | تیرے جیسے گانڈؤں کی وجہ سے وہ ہم لوگوں کو پاگل بنا رہا ہے | 1 |  |  |  |  |  |  |  |  |  |  |
|  | تیرے کو گارڈ نے چود دیا | 1 |  |  |  |  |  |  |  |  |  |  |
|  | تیرے لوڑے سالے دیمک نہں ہے کیارفتار ہے | 1 |  |  |  |  |  |  |  |  |  |  |
|  | تیری اوقات ہے کیا جو تو سر کا مزاق اڑا رہا ہے سب سے گھٹیا چینل ہے تیرا | 0 |  |  |  |  |  |  |  |  |  |  |
|  | تیری بات مجھے بہت پسند آئی | 0 |  |  |  |  |  |  |  |  |  |  |
|  | تیری بہن کی چوت ماروں لکھنا تو بین نہں ہے | 1 |  |  |  |  |  |  |  |  |  |  |
|  | تیری بہن کی لوکی بہت زبر دست تھی | 0 |  |  |  |  |  |  |  |  |  |  |
|  | تیری شکل مجھ کو پسند ہے نہ ہی تیری حیابندر لگ رہا ہے تو گوریلا | 0 |  |  |  |  |  |  |  |  |  |  |
|  | تیری گانڈ کیوں جلتی ہے ہمیشہ ہر چیز سے گانڈو | 1 |  |  |  |  |  |  |  |  |  |  |
|  | تیری گانڈ میں کیا کیڑا ہے بھوسڑی کے | 1 |  |  |  |  |  |  |  |  |  |  |
|  | تیری ماں چود دوں گا | 1 |  |  |  |  |  |  |  |  |  |  |
|  | تیری ماں کا پھدا ماروں ماں کے لوڑے کنجری کے بچے تیری پھدی میں مرچیں ڈال دوں | 1 |  |  |  |  |  |  |  |  |  |  |
|  | تیری ماں کا یار ہے وہ مادرچود کیوں مرچیں لگ رہی ہیں تجھ کو | 1 |  |  |  |  |  |  |  |  |  |  |
|  | تیری ماں کو چودنے والا ہے نا وہ اس لیے تیری اس کی بے عزتی سن کر گانڈ پھٹ رہی ہے | 1 |  |  |  |  |  |  |  |  |  |  |
|  | تیری ماں کی چت اور گشتی کے بچے | 1 |  |  |  |  |  |  |  |  |  |  |
|  | تیری ماں کی چوت | 1 |  |  |  |  |  |  |  |  |  |  |
|  | تیری ماں کی چوت اور ساتھ میں تیری بھی | 1 |  |  |  |  |  |  |  |  |  |  |
|  | تیری ماں کی چوت بھوسڑی کے تو شام کا ٹٹہ ہے بہن کے لوڑے چل دفعہ ہو یہاں سے | 1 |  |  |  |  |  |  |  |  |  |  |
|  | تیری ماں کی چوت تیری بہن کی چوت بہن چود چوتیا سالا | 1 |  |  |  |  |  |  |  |  |  |  |
|  | تیری ماں کی چوت کچھ بھی جھٹو سی چیز بنا رہے ہو | 1 |  |  |  |  |  |  |  |  |  |  |
|  | تیری ماں کی چوت مارا میرانام روہن ہے | 1 |  |  |  |  |  |  |  |  |  |  |
|  | تیری ماں کی چوت ماں کے لوڑے بہن چود | 1 |  |  |  |  |  |  |  |  |  |  |
|  | تیری ماں کی چوت میں کیری بھائی کا لنڈ ماں چود دی ہے نا ابلیس کی | 1 |  |  |  |  |  |  |  |  |  |  |
|  | تیواری مہربانی کر کے میرا چینل دیکھیں اگر آپ کے پاس وقت ہے تو میں عام طور پر ایسا کچھ نہیں لکھتا | 0 |  |  |  |  |  |  |  |  |  |  |
|  | ٹٹوتو تیری ساھی دیکھ رہی ہے | 0 |  |  |  |  |  |  |  |  |  |  |
|  | ٹٹے گدگدا دئیے تم نے کیری | 1 |  |  |  |  |  |  |  |  |  |  |
|  | ٹٹی چاہیے نا | 0 |  |  |  |  |  |  |  |  |  |  |
|  | ٹھیک ماں چودی ہے اس گانڈو کی | 1 |  |  |  |  |  |  |  |  |  |  |
|  | ٹوم سے باتیں کرتے ہوئے دیکھ کر میرے پیٹ میں تتلیاں اڑنے لگیں اور چہرے پہ مسکراہٹ | 0 |  |  |  |  |  |  |  |  |  |  |
|  | ٹی سیریز کو سبسکرائب کریں اور مشہور کریں | 0 |  |  |  |  |  |  |  |  |  |  |
|  | ٹی سیریز کے بارے میں بھی ایک ویڈیو بناؤ | 0 |  |  |  |  |  |  |  |  |  |  |
|  | ٹیک سپورٹ ہماری ڈاکٹروں پر مزاحیہ ویڈیو دیکھں اور اس کو لائیک کریں | 0 |  |  |  |  |  |  |  |  |  |  |
|  | ثمینہ کو بیماری نہیں ہوتی ہے بیماری کو ثمینہ ہوتی ہے | 0 |  |  |  |  |  |  |  |  |  |  |
|  | جا کر اپنے ملک میں دیکھ بھوسڑی کے | 1 |  |  |  |  |  |  |  |  |  |  |
|  | جان دکی بھائی ان چوتیوں کو اسی طرح روسٹ کیا کر | 1 |  |  |  |  |  |  |  |  |  |  |
|  | جانےمن آئی لو یو بات آپ کی بالکل صیح ہے چوتیا ہے بہن چود کا بچہ | 1 |  |  |  |  |  |  |  |  |  |  |
|  | جانی ارے یار اس چوتیاپا کو بلاک کرو اس شام اور فراگی نے بہت چولیں ماریں ہیں | 1 |  |  |  |  |  |  |  |  |  |  |
|  | جانی ٹینشن نہیں لینی ساتھ ہیں تیرے ہم میں چود دوں گا اس کی | 1 |  |  |  |  |  |  |  |  |  |  |
|  | جانی صیح اتار دی تو نے اس کی مار سالے کی بھوسڑی کے تو نے تو آنکھیں ہی کھول دیں | 1 |  |  |  |  |  |  |  |  |  |  |
|  | جانی صیح میں چودی ہے بھڑوے کی | 1 |  |  |  |  |  |  |  |  |  |  |
|  | جائے راؤتیری ماں کو چودتا ہوں بھوسڑی کے رنڈے کی بچے | 1 |  |  |  |  |  |  |  |  |  |  |
|  | جب تک یوٹیوب ہے تب تک یہ لوگوں کی چوت مارتے رہیں گے جھوٹے گانڈو | 1 |  |  |  |  |  |  |  |  |  |  |
|  | جب تم بہت جلدی میں ہوتے اور تم کو نہں پتہ ہوتا کہ کیا کرنا ہے | 0 |  |  |  |  |  |  |  |  |  |  |
|  | جب کبھی میں آپ کی ویڈیوز دیکھتا ہوں تو میں اپنی ہنسی نہیں روک سکتا | 0 |  |  |  |  |  |  |  |  |  |  |
|  | جب کیری بھائی نے کہا کہ گانڈ میں ٹیکے لگوا لگوا کے تو میں نے ویڈیو لائیک کردی | 1 |  |  |  |  |  |  |  |  |  |  |
|  | جب لڑکی نے کہا کہ جمشید تو میں نے سمجھا کہ کچھ خراب ہو گیا ہے | 0 |  |  |  |  |  |  |  |  |  |  |
|  | جس کو بولا اس کی نہیں جلتی اس کی چاٹنے والے آگئے گانڈو گری کرنے | 1 |  |  |  |  |  |  |  |  |  |  |
|  | جس کے عمل اس کے ساتھ آپ کیوں اپنی زبان گندی کرتے ہو گالی دے کر میں اس کا فائن نہں لیکن ایسا کرنا بھی ٹھیک نہیں | 0 |  |  |  |  |  |  |  |  |  |  |
|  | جس کی بگی اس کا گھوڑا | 0 |  |  |  |  |  |  |  |  |  |  |
|  | جس کی بگی اس گھوڑا آج کٹے گابہن کا | 0 |  |  |  |  |  |  |  |  |  |  |
|  | جس نے انور بھائی کو سبسکرائب نہیں کیا تو بہت بڑا مادرچود ہوگا ہو | 1 |  |  |  |  |  |  |  |  |  |  |
|  | جس نے بھی لکھا نا کہ ویڈیو بری تھی پلیز پوری ویڈیو دیکھ کر کمنٹ کرنا اس بار کچھ اچھا کیا | 0 |  |  |  |  |  |  |  |  |  |  |
|  | جگ موہن سنگھ میری ڈاکٹروں پر مزاحیہ ویڈیو دیکھو اور لائیک کریں | 0 |  |  |  |  |  |  |  |  |  |  |
|  | جل پری نہیں یہ جل پرا ہے | 0 |  |  |  |  |  |  |  |  |  |  |
|  | جلد ہی یہ بہترین ویڈیو آگئی ہے | 0 |  |  |  |  |  |  |  |  |  |  |
|  | جلدی اپ لوڈ کر ویڈیو | 0 |  |  |  |  |  |  |  |  |  |  |
|  | جن لوگوں نے ناپسند کیا ان کی ماں کی چوت | 1 |  |  |  |  |  |  |  |  |  |  |
|  | جناب آپ میرا چینل کی مشہوری کریں گے میں آپ کی ہر ویڈیو دیکھتا ہوں | 0 |  |  |  |  |  |  |  |  |  |  |
|  | جناب صیح کام کے لیے آپ کو سلام ہے آپ نے غلط کام کو ظاہر کیا | 0 |  |  |  |  |  |  |  |  |  |  |
|  | جہاں بھی جاؤ گے ادھر جا کر گانڈ ماروں گا تیری اگر شام ادریس کو کچھ کہا تو گانڈ میں گھس کر گانڈ پھاڑ دوں گا | 1 |  |  |  |  |  |  |  |  |  |  |
|  | جہاں تک مجھے پتا ہے دکی نے اس سے ویڈیو بنانے کی اجازت مانگی تھی پر پھر بھی اس نے چوتیاپا کرنا شروع کر دیا | 1 |  |  |  |  |  |  |  |  |  |  |
|  | جہاں سے بھی سے گزرو گے خوش رہو گے | 0 |  |  |  |  |  |  |  |  |  |  |
|  | جہنم میں جاؤ آپ کو ئی حق نہیں کہ آپ اس کے بارے میں بات کریں | 0 |  |  |  |  |  |  |  |  |  |  |
|  | جھک شیخ سالے تو تیری ماں کے ساتھ کتے نے کیا تھا تو توں آیا تھا بہن چود ہا ہا ہا | 1 |  |  |  |  |  |  |  |  |  |  |
|  | جھوٹ پر جھوٹ جھوٹ پر جھوٹ یہ چوتیا پاگل ہے قسم سے | 1 |  |  |  |  |  |  |  |  |  |  |
|  | جھوٹا ابلیس بھوسڑی کا | 1 |  |  |  |  |  |  |  |  |  |  |
|  | جھوٹا ڈرامہ بکواس چوتیاپا سارا ٹائم ضائع کردیا | 1 |  |  |  |  |  |  |  |  |  |  |
|  | جھوٹا شام چوتیا شام فضل چوتیا شام گانڈو | 1 |  |  |  |  |  |  |  |  |  |  |
|  | جھوٹا ہے بھوسڑی کا شام | 1 |  |  |  |  |  |  |  |  |  |  |
|  | جھوٹے ادریس کی گانڈ ماردی آپ نے شاباش | 1 |  |  |  |  |  |  |  |  |  |  |
|  | جھوٹے تو ہے ہی چوتیا لیکن تو نے کیوں رنڈی رونا لگایا ہوا ہے موضوع تبدیل کرو | 1 |  |  |  |  |  |  |  |  |  |  |
|  | جھوٹے کی ماں چود دی آج تو | 1 |  |  |  |  |  |  |  |  |  |  |
|  | جھوٹی آواز میں بات مت کرو اور اصلی آواز میں کہو کہ وہ چوتیا ہے | 1 |  |  |  |  |  |  |  |  |  |  |
|  | جو بھی ہو بھائی تم میرے پسندیدہ انڈین یوٹیوبر ہو | 0 |  |  |  |  |  |  |  |  |  |  |
|  | جو لوگ دکی بھائی کی بجائے شام ادریس کی حمایت کر رہے ہیں میں ان سے صرف یہ کہوں گا کہ وہ ابلیس کے کھبے ٹٹے ہیں | 1 |  |  |  |  |  |  |  |  |  |  |
|  | جوتیے جیسی ہے ویڈیو | 1 |  |  |  |  |  |  |  |  |  |  |
|  | جونگ چپ کر بھوسڑی کے چودو انسان | 1 |  |  |  |  |  |  |  |  |  |  |
|  | جی بہت مزہ آگیا کیری بھائی | 0 |  |  |  |  |  |  |  |  |  |  |
|  | جی وائرل ہاں ہاں ہاں | 0 |  |  |  |  |  |  |  |  |  |  |
|  | جیت ٹھاکرے ناچ | 0 |  |  |  |  |  |  |  |  |  |  |
|  | جیتے رہو جناب یہ پاکستان آرہا ہے تم پکڑ کر گانڈ مارو اسکی | 1 |  |  |  |  |  |  |  |  |  |  |
|  | جیتے رہو لڑکے بہت گندی کر دی شام کی | 1 |  |  |  |  |  |  |  |  |  |  |
|  | جیسے کہ آپ سب لوگوں نے دیکھا کیری اپنی ویڈیو کے آخر میں بھولا آدمی بن جاتا ہے پر ویڈیومیں گالی نکالتا ہے | 0 |  |  |  |  |  |  |  |  |  |  |
|  | چپ رہو گانڈو آدمی | 1 |  |  |  |  |  |  |  |  |  |  |
|  | چپ مادر چود گانڈ پھٹے گی لوڑے اور تیرے دماغ میں تو نادیہ علی پھنسی ہوئی ہے | 1 |  |  |  |  |  |  |  |  |  |  |
|  | چت پھاڑ ویڈیو دوبارہ | 1 |  |  |  |  |  |  |  |  |  |  |
|  | چت چھنڈ یار تم نا یار بریک اپ پر ایک ویڈیو بناؤ پلیز | 1 |  |  |  |  |  |  |  |  |  |  |
|  | چتو لوڑا لے لے میرا | 1 |  |  |  |  |  |  |  |  |  |  |
|  | چتیاپا کا حد ہو گیا | 1 |  |  |  |  |  |  |  |  |  |  |
|  | چس آگئی میں بھی اپنے بلاگ میں اس بھڑوے کی گندی کرتا رہتا ہوں | 1 |  |  |  |  |  |  |  |  |  |  |
|  | چس بن ہے چوتیا تو بنا نہیں ہے | 1 |  |  |  |  |  |  |  |  |  |  |
|  | چکن ڈنر کے لیے ترکیب | 0 |  |  |  |  |  |  |  |  |  |  |
|  | چل تیری بہن کی چوت | 1 |  |  |  |  |  |  |  |  |  |  |
|  | چلو اتنی ساری کیری لائیو آیا اور ایک نئی ویڈیو دیکھنے کو ملی | 0 |  |  |  |  |  |  |  |  |  |  |
|  | چلو ٹھیک ہے تم صیح بول رہے ہو پر بھوسڑی کے سالے سب کو بتاتے پھر رہے ہیں لوڑے | 1 |  |  |  |  |  |  |  |  |  |  |
|  | چہرہ سی صرف بنایا کر | 0 |  |  |  |  |  |  |  |  |  |  |
|  | چھا گیا بھائی تو اس کی گانڈ پر کوئی دوائی لگاؤ تاکہ جو کیڑا اس کو تنگ کرتا ہے وہ مر جائے | 1 |  |  |  |  |  |  |  |  |  |  |
|  | چھا گیا لڑکا کینسر مٹاؤ پاکستان بچاؤ ابلیس بھڑوا | 1 |  |  |  |  |  |  |  |  |  |  |
|  | چھا گئے دکی بھائی وہ ہم سے کما رہا تھا اب اس بہن چود کا چینل بند ہوگا تو مزہ آ جائے گا | 1 |  |  |  |  |  |  |  |  |  |  |
|  | چھا گئے ہو بھائی اس بھوسڑٖی کے نے عوام کو پھدو لگا رکھا ہے | 1 |  |  |  |  |  |  |  |  |  |  |
|  | چھا گئے ہو دکی بھائی با لکل صیح فرمایا آپ نے اس چوتیے کے بارے میں | 1 |  |  |  |  |  |  |  |  |  |  |
|  | چھٹی ختم جاؤ سب واپس ھاسٹل | 0 |  |  |  |  |  |  |  |  |  |  |
|  | چھٹی کے وقت بچے | 0 |  |  |  |  |  |  |  |  |  |  |
|  | چھوٹا ڈرامہ کاپی کیا ہے بہن چود | 1 |  |  |  |  |  |  |  |  |  |  |
|  | چھوٹے یوٹیوبر اس بھوسڑی والے کو بتاؤ کہ اس کے ویور میں صرف یوٹیوبر ہی شامل ہیں اور ہمارے ویور پوری دینا سے ہیں | 1 |  |  |  |  |  |  |  |  |  |  |
|  | چھوٹی پروڈکشن ہے بھائی ساھی ھا بہھت چوتیا ہے سالا | 1 |  |  |  |  |  |  |  |  |  |  |
|  | چھوٹی ویڈیو صرف چیک کرو دکی بھائی فارغ وقت میں چوتیا یہ چوسرڑ کنجر | 1 |  |  |  |  |  |  |  |  |  |  |
|  | چھوڑ ان چوتیوں کو ان کو صرف پیسا چاہئے یہ بات ان کی سمجھ میں نہں آئے گی | 1 |  |  |  |  |  |  |  |  |  |  |
|  | چھوڑ ان ماں کے لوڑوں کو ان کے بارے میں جتنی ویڈیوز بناؤ گے اتنا انہوں نے مشہور ہونا ہے | 1 |  |  |  |  |  |  |  |  |  |  |
|  | چھوڑ بھائی اس چوتیا پر لعنت بھیج | 1 |  |  |  |  |  |  |  |  |  |  |
|  | چھوڑ دے بھائی اس مادر چود کو مر جائے گا یہ | 1 |  |  |  |  |  |  |  |  |  |  |
|  | چھوڑ دے بھوسڑی والے کو چوتیے تو باز نہیں آئے گا | 1 |  |  |  |  |  |  |  |  |  |  |
|  | چھوڑ نا یار یہ کالی گانڈ والے انگریز کے لوڑے | 1 |  |  |  |  |  |  |  |  |  |  |
|  | چھوڑ یار بہن کا لوڑ ا ہے وہ تو بنڈ بار اس کی | 1 |  |  |  |  |  |  |  |  |  |  |
|  | چھوڑو اس ذلیل انسان کو یہ بندہ اتنا بڑا چوتیا ہے کہ کیا کہوں | 1 |  |  |  |  |  |  |  |  |  |  |
|  | چھی چھی یہ کیا دیکھ لیا | 0 |  |  |  |  |  |  |  |  |  |  |
|  | چوت ماری کے بگ باس میں جانے کی تیری اوقات بھی نہیں ہے | 1 |  |  |  |  |  |  |  |  |  |  |
|  | چوت ماری کے ڈھکن مست ہے | 1 |  |  |  |  |  |  |  |  |  |  |
|  | چوت مرانی دوسری ویڈیو بناتا ہے تو اس کو مزاق کیوں اڑاتا ہے | 1 |  |  |  |  |  |  |  |  |  |  |
|  | چوت مرائی کے تو ہے کوئی شق تو نہیں ہے اس میں | 1 |  |  |  |  |  |  |  |  |  |  |
|  | چوت مرائی کے کوئی کام دھندا نہیں ہے دوسری کی زندگی میں انگلی کرنے کے علاوہ | 1 |  |  |  |  |  |  |  |  |  |  |
|  | چوت مرائی کے کئی ہفتے ہو گئے ابھی تک ویڈیو نہیں آئی | 1 |  |  |  |  |  |  |  |  |  |  |
|  | چوتڑ اتنے دنوں بعد ویڈیو اپ لوڈ کی تیری منت | 1 |  |  |  |  |  |  |  |  |  |  |
|  | چوتڑ بھاگ یہاں سے لوڑے | 1 |  |  |  |  |  |  |  |  |  |  |
|  | چوتڑ پھیلانے کو جگہ نہیں ملا بس | 1 |  |  |  |  |  |  |  |  |  |  |
|  | چوتڑ دل بائیں طرف ہوتا ہے | 1 |  |  |  |  |  |  |  |  |  |  |
|  | چوتڑ کچھ بڑھیا موضوع پر ویڈیو بناؤ یہ کیا بکواس ہے | 1 |  |  |  |  |  |  |  |  |  |  |
|  | چوتڑ میں چل مچ گئی میں تیری بات مان کے مٹھ مار لوں گا | 1 |  |  |  |  |  |  |  |  |  |  |
|  | چوتڑ ہے یہ سالا | 1 |  |  |  |  |  |  |  |  |  |  |
|  | چوتڑ والا منظر آئے ہائے | 1 |  |  |  |  |  |  |  |  |  |  |
|  | چوتسپا بھی بین ہے | 1 |  |  |  |  |  |  |  |  |  |  |
|  | چوتمگان چرسی چوراسیا ہے دونوں یار منفی شہرت کے لیے اتنی بک چودی دھت تیری ماں | 1 |  |  |  |  |  |  |  |  |  |  |
|  | چوتنے بھی لے لے اچھی رہے گی | 1 |  |  |  |  |  |  |  |  |  |  |
|  | چوتنے بھی لے لے اچھی لگے گی | 1 |  |  |  |  |  |  |  |  |  |  |
|  | چوتیا ابلیس کتی کا بچہ | 1 |  |  |  |  |  |  |  |  |  |  |
|  | چوتیا اور گانڈو بھی بننا ہے کیا بھائی | 1 |  |  |  |  |  |  |  |  |  |  |
|  | چوتیا اور گانڈو بھی بننا ہے کیا دکی بھائی | 1 |  |  |  |  |  |  |  |  |  |  |
|  | چوتیا ایک نمبر کا بھڑوا سالا | 1 |  |  |  |  |  |  |  |  |  |  |
|  | چوتیا بس اس کے لیے چوتیا بننا پڑتا ہے صیح کہا | 1 |  |  |  |  |  |  |  |  |  |  |
|  | چوتیا بناؤ اور بناؤ | 1 |  |  |  |  |  |  |  |  |  |  |
|  | چوتیا بندہ ہے بس وہ فیس بک پیچ پر وہ سب سے بڑا بہن چود ہے | 1 |  |  |  |  |  |  |  |  |  |  |
|  | چوتیا بندہ ہے تو چوتیا | 1 |  |  |  |  |  |  |  |  |  |  |
|  | چوتیا بنے نہیں ہیں | 1 |  |  |  |  |  |  |  |  |  |  |
|  | چوتیا بھاگ یہاں سے بڑا آیا تیرے بھائ کوبینگ کرے گا گس | 1 |  |  |  |  |  |  |  |  |  |  |
|  | چوتیا بھوجی پوری آٹو ٹیون کا بھی ماں چود دیا بھوسڑی کے | 1 |  |  |  |  |  |  |  |  |  |  |
|  | چوتیا جا بیٹا دکی بھائی سے کچھ سیکھ | 1 |  |  |  |  |  |  |  |  |  |  |
|  | چوتیا چلا تھا تیرے کو روسٹ کرنے | 1 |  |  |  |  |  |  |  |  |  |  |
|  | چوتیا حرام زادہ لو یو بھائی | 1 |  |  |  |  |  |  |  |  |  |  |
|  | چوتیا دماغ خراب کردیا تم نے آج میرا | 1 |  |  |  |  |  |  |  |  |  |  |
|  | چوتیا دیپک گانڈو سالا | 1 |  |  |  |  |  |  |  |  |  |  |
|  | چوتیا رام راحیم | 1 |  |  |  |  |  |  |  |  |  |  |
|  | چوتیا ساسوماں پاگل | 1 |  |  |  |  |  |  |  |  |  |  |
|  | چوتیا شام بھوسڑی کا تم بہت اچھے ہو بھائی | 1 |  |  |  |  |  |  |  |  |  |  |
|  | چوتیا شرابی کتا سالا | 1 |  |  |  |  |  |  |  |  |  |  |
|  | چوتیا فالتو ویڈیو | 1 |  |  |  |  |  |  |  |  |  |  |
|  | چوتیا کہیں کی | 1 |  |  |  |  |  |  |  |  |  |  |
|  | چوتیا کھیل ہے بگ باس | 1 |  |  |  |  |  |  |  |  |  |  |
|  | چوتیا گانا ہے | 1 |  |  |  |  |  |  |  |  |  |  |
|  | چوتیا لفظ تو منع نہں ہے | 1 |  |  |  |  |  |  |  |  |  |  |
|  | چوتیا لگ رہا لیکن پھر بھی تیری ویڈیو مزاحیہ ہے | 1 |  |  |  |  |  |  |  |  |  |  |
|  | چوتیا لگ رہا ہے | 1 |  |  |  |  |  |  |  |  |  |  |
|  | چوتیا لگتا ہے تو بھوسڑی کے | 1 |  |  |  |  |  |  |  |  |  |  |
|  | چوتیا لنڈ کا بال | 1 |  |  |  |  |  |  |  |  |  |  |
|  | چوتیا لوگ لائیک دیں گے | 1 |  |  |  |  |  |  |  |  |  |  |
|  | چوتیا لوگ ہیں یہ | 1 |  |  |  |  |  |  |  |  |  |  |
|  | چوتیا مواد بکواس چینل بند کر لو اگر ایسا بکواس مواد اپ لوڈ کرنا ہے تو | 1 |  |  |  |  |  |  |  |  |  |  |
|  | چوتیا ہے ابے تو | 1 |  |  |  |  |  |  |  |  |  |  |
|  | چوتیا ہے بھائی تو | 1 |  |  |  |  |  |  |  |  |  |  |
|  | چوتیا ہے تو کوئی ٹیلنٹ نہیں ہے تیرے پاس | 1 |  |  |  |  |  |  |  |  |  |  |
|  | چوتیا ہے تو لوڑے | 1 |  |  |  |  |  |  |  |  |  |  |
|  | چوتیا ہے دکی بھائی | 1 |  |  |  |  |  |  |  |  |  |  |
|  | چوتیا ہے سالا | 1 |  |  |  |  |  |  |  |  |  |  |
|  | چوتیا ہے سالا | 1 |  |  |  |  |  |  |  |  |  |  |
|  | چوتیا ہے شام ادریس | 1 |  |  |  |  |  |  |  |  |  |  |
|  | چوتیا ہے شام اس کے چینل سے دور رہو | 1 |  |  |  |  |  |  |  |  |  |  |
|  | چوتیا ہے کہ بھوتنی کے جب ویڈیو نہیں بنتی تو لنڈ کے لیے بنا رہا لوڑے جیسا ری ایکشن دے رہا بس | 1 |  |  |  |  |  |  |  |  |  |  |
|  | چوتیا ہے وقار سالا | 1 |  |  |  |  |  |  |  |  |  |  |
|  | چوتیا ہے یہ کچھ بھی بناتا ہے | 1 |  |  |  |  |  |  |  |  |  |  |
|  | چوتیا ہے یہ لوگ | 1 |  |  |  |  |  |  |  |  |  |  |
|  | چوتیا وقار ذکاٰء | 1 |  |  |  |  |  |  |  |  |  |  |
|  | چوتیا ویڈیو مزہ نہیں آیا | 1 |  |  |  |  |  |  |  |  |  |  |
|  | چوتیا یوٹیوبر ایسا بولتا ہے جیسے لاکھوں سبسکرائبرز اپنی گانڈ میں لے کر آیا تھا ماں کی پیٹ میں سے | 1 |  |  |  |  |  |  |  |  |  |  |
|  | چوتیاپا ابھی بند نہیں ہوا میں نے ابھی ابھی چیک کیا ہے | 1 |  |  |  |  |  |  |  |  |  |  |
|  | چوتیاپا اکیلے خود چوتیا بننا پڑتا ہے | 1 |  |  |  |  |  |  |  |  |  |  |
|  | چوتیاپا بند کر بھای | 1 |  |  |  |  |  |  |  |  |  |  |
|  | چوتیاپا بند کرو | 1 |  |  |  |  |  |  |  |  |  |  |
|  | چوتیاپا چیز ہے یہ بڑا صاحب | 1 |  |  |  |  |  |  |  |  |  |  |
|  | چوتیاپا کا شو بس | 1 |  |  |  |  |  |  |  |  |  |  |
|  | چوتیاپا کر رہا ہے | 1 |  |  |  |  |  |  |  |  |  |  |
|  | چوتیاپا کرنے سے بہتر ہےپہلے جیسا کام کر تو کمنٹری سہی کرتا ہے اداکاری نہیں | 1 |  |  |  |  |  |  |  |  |  |  |
|  | چوتیاپا گانڈ مار لین لارڈ | 1 |  |  |  |  |  |  |  |  |  |  |
|  | چوتیاپا لیکن ریپ مست تھا | 1 |  |  |  |  |  |  |  |  |  |  |
|  | چوتیاپا مت کرو | 1 |  |  |  |  |  |  |  |  |  |  |
|  | چوتیاپا ہے دن چک پوجا | 1 |  |  |  |  |  |  |  |  |  |  |
|  | چوتیاپا ہےتو مادر چود | 1 |  |  |  |  |  |  |  |  |  |  |
|  | چوتیاپا ہی رہ جانا ہے اگر تمہں پتا نہ ہو تو تھوڑا گوگل کر لو تمہں سب کچھ آتا تو ہے | 1 |  |  |  |  |  |  |  |  |  |  |
|  | چوتیاپا ویڈیو تھا بالکل بکواس | 1 |  |  |  |  |  |  |  |  |  |  |
|  | چوتیاپوں کی برینڈ ہے پوری زندگی ٹٹی کر کے رکھ دی ہے | 1 |  |  |  |  |  |  |  |  |  |  |
|  | چوتیاپے کی حد ہو گئی یار | 1 |  |  |  |  |  |  |  |  |  |  |
|  | چوتیاپے کی ویڈیو بنائی اس مرتبہ اب لیول وہ نہیں رہا پہلے والا | 1 |  |  |  |  |  |  |  |  |  |  |
|  | چوتیارے انڈین سالے | 1 |  |  |  |  |  |  |  |  |  |  |
|  | چوتیاگری جس کی روٹی اس کی بوٹی ذکاہ کی کاٹ دو گوٹی | 1 |  |  |  |  |  |  |  |  |  |  |
|  | چوتیاں کلیاں وے | 1 |  |  |  |  |  |  |  |  |  |  |
|  | چوتیائے چمن | 1 |  |  |  |  |  |  |  |  |  |  |
|  | چوتیوں انڈر ورلڈ ڈان کھٹی لسی اور دوسرا نعمان خان مس چھکا | 1 |  |  |  |  |  |  |  |  |  |  |
|  | چوتیوں تم لوگوں کا کام دھندہ نہیں ہے کیا | 1 |  |  |  |  |  |  |  |  |  |  |
|  | چوتیوں جیسی ویڈیوز بناتا ہے اور کام نہیں ہے تم سے | 1 |  |  |  |  |  |  |  |  |  |  |
|  | چوتیوں ک لئے اچھا موقع ہوتا ہے بگ باس چوتیا کا بنایا ہوا ہے | 1 |  |  |  |  |  |  |  |  |  |  |
|  | چوتیوں کا بازار ہے پورکستان میں | 1 |  |  |  |  |  |  |  |  |  |  |
|  | چوتیوں کا دیش ہے پورکستان ایسے ایسے لوگ وہاں شو کرتے ہیں | 1 |  |  |  |  |  |  |  |  |  |  |
|  | چوتیوں کتنا دیکھو گے | 1 |  |  |  |  |  |  |  |  |  |  |
|  | چوتیوں کچھ تو نیا کمنٹ کرو | 1 |  |  |  |  |  |  |  |  |  |  |
|  | چوتیوں کو روسٹ کرتے کرتے خود چوتیا بن گیا | 1 |  |  |  |  |  |  |  |  |  |  |
|  | چوتیوں کو نہیں سمجھ آئے گی | 1 |  |  |  |  |  |  |  |  |  |  |
|  | چوتیوں کو یہ ویڈیو پسند نہیں آئے گی | 1 |  |  |  |  |  |  |  |  |  |  |
|  | چوتیوں کوبڑی ہنسی آئی نا بھائی تو نہیں پہنچ پایا اس لیول پر اس لیئے اتنی بک چودی کر رہا ہے | 1 |  |  |  |  |  |  |  |  |  |  |
|  | چوتیوں کوئی ہے | 1 |  |  |  |  |  |  |  |  |  |  |
|  | چوتیوں کی ٹولی رنگ ٹونز کے لنک شئیر کرنا | 1 |  |  |  |  |  |  |  |  |  |  |
|  | چوتیوں کی شادی | 1 |  |  |  |  |  |  |  |  |  |  |
|  | چوتیوں کی کمی دینا میں بھائی تیرا کاروبار چلتا رہے گا سلاتا رہ دوسروں کو | 1 |  |  |  |  |  |  |  |  |  |  |
|  | چوتیوں کی کمی نہیں | 1 |  |  |  |  |  |  |  |  |  |  |
|  | چوتیوں کی کمی نہیں ہاں اس دنیا میں | 1 |  |  |  |  |  |  |  |  |  |  |
|  | چوتیوں کی کمی نہیں ہوگی | 1 |  |  |  |  |  |  |  |  |  |  |
|  | چوتیوں کی کمی ہےکہ کچھ دن تو گزارو گجرات میں بس | 1 |  |  |  |  |  |  |  |  |  |  |
|  | چوتیوں کی نگرانی میں | 1 |  |  |  |  |  |  |  |  |  |  |
|  | چوتیوں کی نگرانی میں ہا ہا | 1 |  |  |  |  |  |  |  |  |  |  |
|  | چوتیوں نے کمنٹس کو ڈس ایبل کر دیا | 1 |  |  |  |  |  |  |  |  |  |  |
|  | چوتیوں نے ناپسند کردیاہے | 1 |  |  |  |  |  |  |  |  |  |  |
|  | چوتیوں والی ویڈیو بس دوست بھی اپنی طرح کی چوتیے چیز دیکھتے ہیں | 1 |  |  |  |  |  |  |  |  |  |  |
|  | چوتیوں یہ کیا اس کا سدا منہ دیکھ رہے ہو جاؤ ڈیڈپول ہندی کا نیا ویڈیو ریلیز ہوا ہے جاؤدیکھو | 1 |  |  |  |  |  |  |  |  |  |  |
|  | چوتیے اچھے سے چلا | 1 |  |  |  |  |  |  |  |  |  |  |
|  | چوتیے اور کوئی موضوع نہیں ملا تو بگ باس کو پکڑ لیا | 1 |  |  |  |  |  |  |  |  |  |  |
|  | چوتیے بی بی کا فیک ہے | 1 |  |  |  |  |  |  |  |  |  |  |
|  | چوتیے تجھ کو ساری ویڈیو دیکھنے کے بعد بھی شرم نہیں آیا | 1 |  |  |  |  |  |  |  |  |  |  |
|  | چوتیے تو تو ویڈیو کیوں بناتا ہے یہ بتا صرف | 1 |  |  |  |  |  |  |  |  |  |  |
|  | چوتیے جیسا گانا ہے | 1 |  |  |  |  |  |  |  |  |  |  |
|  | چوتیے جیسی شکل بناتا ہے چوتیے | 1 |  |  |  |  |  |  |  |  |  |  |
|  | چوتیے چینل بند کر دے ورنہ تیری گانڈ مار لوں گا | 1 |  |  |  |  |  |  |  |  |  |  |
|  | چوتیے کپڑے صرف پہلی بار نہیں ہوتے اس کے بعد مل جاتے ہیں | 1 |  |  |  |  |  |  |  |  |  |  |
|  | چوتیے کتے کمینے کیوں اتنا جلتا ہے فیس بک پیج کو لوڑے لگایا تجھے بھی نہ لوڑے لگوا دے سیدھا ہو جا | 1 |  |  |  |  |  |  |  |  |  |  |
|  | چوتیے کتے کمینے کیوں جلتا ہے اس سے | 1 |  |  |  |  |  |  |  |  |  |  |
|  | چوتیے کچھ اچھا بنا تم اچھی شراب بناتے ہو اور یہ ویڈیو لیٹ کیوں آرہی ہے | 1 |  |  |  |  |  |  |  |  |  |  |
|  | چوتیے کے ڈھکن یار تو گانڈ دے دے | 1 |  |  |  |  |  |  |  |  |  |  |
|  | چوتیے کی نسل روپے یوٹیوب پر کما اور نکل سائیکل لینے کی اوقات ہے نہیں انڈیا والوں کی | 1 |  |  |  |  |  |  |  |  |  |  |
|  | چوتیے لوگ استعمال کرتع ہے موسیقی ڈش کا نام خراب کر رہے ہیں | 1 |  |  |  |  |  |  |  |  |  |  |
|  | چوتیے لوگ ہیں پاکستانی سیدھا سیدھا پیج کو ناپسند کردیں اور کھیل ختم | 1 |  |  |  |  |  |  |  |  |  |  |
|  | چوتیے لوگوں کے اوپر ویڈیو ہے بند کر بھائی | 1 |  |  |  |  |  |  |  |  |  |  |
|  | چوتیے میری طرف سے دنیا کی سب سے گندی گالی رکھ لو | 1 |  |  |  |  |  |  |  |  |  |  |
|  | چوتیے نے انگلش بھی بین کی ہوئی ہے پر ہم تو دیسی اردو میں بھی اس کو گالیاں نکال دیتے ہیں ماں بہن کی | 1 |  |  |  |  |  |  |  |  |  |  |
|  | چوتیے ہیں سارے لوگ | 1 |  |  |  |  |  |  |  |  |  |  |
|  | چوتیے ہیں سالے پاکستان کو اپنی مشہوری کے لیے استعمال کر رہے ہیں | 1 |  |  |  |  |  |  |  |  |  |  |
|  | چوتیے ہیں مادرچود بھوسڑی کے ایک گورا ہونے کا انجیکشن لگاتا دوسرا بیس لاگا کر آیا ہوتا | 1 |  |  |  |  |  |  |  |  |  |  |
|  | چوتیے ہیں یہ سب تیرا بھائی سب سے مست ہے | 1 |  |  |  |  |  |  |  |  |  |  |
|  | چوتیےکے لنڈ ہیں دونوں | 1 |  |  |  |  |  |  |  |  |  |  |
|  | چوتییز جیسا ہے ویڈسچ میں نہیں مزہ آیا | 1 |  |  |  |  |  |  |  |  |  |  |
|  | چود دے شام کو حرام ذادہ فراڈ | 1 |  |  |  |  |  |  |  |  |  |  |
|  | چود دیا بخیر تیل کے | 1 |  |  |  |  |  |  |  |  |  |  |
|  | چودو یار اس شام کو صیح طرح سے پوری زندگی پاکستان اور پاکستانی کی بے عزتی کرو گے | 1 |  |  |  |  |  |  |  |  |  |  |
|  | چودے چودی ہو گئی سب دیکھ رہے ہیں آج کل | 1 |  |  |  |  |  |  |  |  |  |  |
|  | چیتا بہت دلا بندہ ہے بھوسڑی ہے | 1 |  |  |  |  |  |  |  |  |  |  |
|  | حجاب آپ لے گئے بازی ان کنجروں کی گانڈ مار کر | 1 |  |  |  |  |  |  |  |  |  |  |
|  | حجاب بہن چود حجاب | 1 |  |  |  |  |  |  |  |  |  |  |
|  | حجاب بھائی اب ہم آپ کی عزت کرتے ہیں یہ ادریس بہت بڑا چوتیا بندہ ہے آپ اجھے ہو پاکستان سے | 1 |  |  |  |  |  |  |  |  |  |  |
|  | حجاب تم کو سلام ہے دکی گانڈ پھاڑدی تم نے ان دونوں کی | 1 |  |  |  |  |  |  |  |  |  |  |
|  | حجاب جھوٹا چوتیا | 1 |  |  |  |  |  |  |  |  |  |  |
|  | حجاب چھا گئے ہو تم دکی او دلے او کمینے | 1 |  |  |  |  |  |  |  |  |  |  |
|  | حجاب شام چوتیا ہے اصل میں | 1 |  |  |  |  |  |  |  |  |  |  |
|  | حجاب کی بےعزتی کنے والا ہے تو بہت اچھا ہے بس باقی سب چوتیے ہیں ابھی بھڑوے کیوں تو گالیاں دیتا ہے | 1 |  |  |  |  |  |  |  |  |  |  |
|  | حچاب شکریہ یار آخر کوئی تو ہے جو اس چوتیے کے بارے میں بول رہا ہے | 1 |  |  |  |  |  |  |  |  |  |  |
|  | حد سے زیادہ پسند آیا یار | 0 |  |  |  |  |  |  |  |  |  |  |
|  | حق بنتا ہے کی اس کی ویڈٰو کو دوبارہ روسٹ کریں | 0 |  |  |  |  |  |  |  |  |  |  |
|  | حقیقت میں اس کا کیا مطلب ک میں ہوں | 0 |  |  |  |  |  |  |  |  |  |  |
|  | حقیقت میں بگ باس ٹٹی ہے مانو یا نہ مانو | 0 |  |  |  |  |  |  |  |  |  |  |
|  | حقیقت میں بہت اچھی ویڈیو ہے | 0 |  |  |  |  |  |  |  |  |  |  |
|  | حمزہ کیسا ہے | 0 |  |  |  |  |  |  |  |  |  |  |
|  | حیران کر دیا کیری بھائی نے نئی ویڈیوز کے لیے نیک خواہشات ہیں | 0 |  |  |  |  |  |  |  |  |  |  |
|  | حیران کن کیا میں اس قسم کی اور ویڈیوز پر لائیکس لے سکتا ہوں | 0 |  |  |  |  |  |  |  |  |  |  |
|  | حیران ہو جاؤ گے اگر میری ویڈیوز دیکھو گے تو | 0 |  |  |  |  |  |  |  |  |  |  |
|  | خبروں والوں کو اور کوئی کام نہیں ہے | 0 |  |  |  |  |  |  |  |  |  |  |
|  | خدا قسم اس نے تو نعمان خان کو بھی پیچھے چھوڑ دیا | 0 |  |  |  |  |  |  |  |  |  |  |
|  | خدا قسم حمزہ ایک اچھا اداکار ہے میں مزاق نہیں کر رہا | 0 |  |  |  |  |  |  |  |  |  |  |
|  | خط اس کے ہاتھ میں لگا کیا | 0 |  |  |  |  |  |  |  |  |  |  |
|  | خطرناک ہے بھائی تو ایک دم بار بار ویڈیو بناتا ہے تو جو بولتا ہے وہ بالکل صیح ہوتا ہے | 0 |  |  |  |  |  |  |  |  |  |  |
|  | خطرناک والا کیا | 0 |  |  |  |  |  |  |  |  |  |  |
|  | خوش بہتر آجائیں بھائی | 0 |  |  |  |  |  |  |  |  |  |  |
|  | خوش رہو بڑے کیری اچھی ویڈیو تھی | 0 |  |  |  |  |  |  |  |  |  |  |
|  | خوش ہو جا بھائی اب تو اشتہار بھی آنے لگا ہے شام تیری بہن کو لن | 1 |  |  |  |  |  |  |  |  |  |  |
|  | خون پسینہ موت بہا وے | 0 |  |  |  |  |  |  |  |  |  |  |
|  | دارو دارو دارو لنڈ سے تیرے منہ میں موت ڈالوں | 1 |  |  |  |  |  |  |  |  |  |  |
|  | داڑھی میں بچہ لگ رہا ہے نعمان خان کی طرح | 0 |  |  |  |  |  |  |  |  |  |  |
|  | دکی انہوں نے عوام کو چوتیا بنایا ہوا ہے اور عوام کتوں کی طرح پاگل ہے | 1 |  |  |  |  |  |  |  |  |  |  |
|  | دکی بھائی اپنا بھائی ہے چوتیے شام تیری ویڈیوز دیکھ کر بہت خوشی ہوئی | 1 |  |  |  |  |  |  |  |  |  |  |
|  | دکی بھائی اگر اس بھوسڑی ابلیس میں تھوڑی سی بھی شرم باقی رہ گئی ہو گی جو کہ امید ہے کہ یہ اب یوٹیوب پر اب دوبارہ بکواس نہیں کرے گا | 1 |  |  |  |  |  |  |  |  |  |  |
|  | دکی بھائی ایسے جھوٹے لوگوں کی وجہ سے پریشان مت ہوں آپ گانڈ ماریں تے ان سالوں کی ہم | 1 |  |  |  |  |  |  |  |  |  |  |
|  | دکی بھائی آپ بہت عظیم ہو اور یہ فراگی سالی کب مرے گی یار | 1 |  |  |  |  |  |  |  |  |  |  |
|  | دکی بھائی آپ ٹینشن نہ لیں ان گانڈو کے چینل کو بلا ک کر دو صرف | 1 |  |  |  |  |  |  |  |  |  |  |
|  | دکی بھائی آپ کا کچھ زور نہیں کسی یہ ترقی کرتا دیکھ نہں سکتا گانڈو | 1 |  |  |  |  |  |  |  |  |  |  |
|  | دکی بھائی تم نے اس ہائی کلاس چوتیا کی گانڈ مار دی صیح کر کے | 1 |  |  |  |  |  |  |  |  |  |  |
|  | دکی بھائی تمہا را کام بہت اچھا ہے یہ شام ہے ہی بہت گانڈو ناصر خان جان کی طرح | 1 |  |  |  |  |  |  |  |  |  |  |
|  | دکی بھائی چھا گئے دوبارہ مزہ آگیا دل سے سپورٹ ہے آپ کی فل چوتیا ہے وہ چبل ہے ہم سب یہ جانتے ہیں | 1 |  |  |  |  |  |  |  |  |  |  |
|  | دکی بھائی چھوڑو بچارے کو روتے ہوئے اپنی امی کو نہ شکایت کر دے بھوسڑی کا | 1 |  |  |  |  |  |  |  |  |  |  |
|  | دکی بھائی زندہ باد شام چوتیا | 1 |  |  |  |  |  |  |  |  |  |  |
|  | دکی بھائی زندہ باد شام گانڈو مردہ باد | 1 |  |  |  |  |  |  |  |  |  |  |
|  | دکی بھائی سائنسی چوتیا ہے شام سالا | 1 |  |  |  |  |  |  |  |  |  |  |
|  | دکی بھائی شو دے دے اس کو بھوسڑی کا چوتیا ہے سالا | 1 |  |  |  |  |  |  |  |  |  |  |
|  | دکی بھائی کون ہے فضل دکی بھائی کی ماں کی چوت چوتیا دکی بھائی تیری ماں کی چوت سالا اپنی ویڈیو دیکھتانہیں اور دوسروں کی ویڈیو پر ردعمل | 1 |  |  |  |  |  |  |  |  |  |  |
|  | دکی بھائی کے سپورٹرز ہم ہیں نا ہم سب مل کر اس کے پیچ کو رپورٹ کر دیتے ہیں یا ایسا کچھ کر دیتے ہیں کہ وہ بہن کا لوڑا کچھ بھی نہ کر سکے | 1 |  |  |  |  |  |  |  |  |  |  |
|  | دکی بھائی گانڈ پھاڑ دی شام کی | 1 |  |  |  |  |  |  |  |  |  |  |
|  | دکی بھائی لو یو بہن چود کو بہت ذلیل کیا ہے | 1 |  |  |  |  |  |  |  |  |  |  |
|  | دکی بھائی نا امیدی کی حد ہوتی ہے چھٹی لو اور مزے اٹھاؤ ان گدھوں کو گدھے ہی رہنے دو | 1 |  |  |  |  |  |  |  |  |  |  |
|  | دکی بھائی نے تو دلے کی ماں چود دی | 1 |  |  |  |  |  |  |  |  |  |  |
|  | دکی بھائی نے تو گانڈ مار لی اس کی شادی کر لے تیرے نشانے پر ہے حجاب | 1 |  |  |  |  |  |  |  |  |  |  |
|  | دکی بھائی نے شام کی گندی والی لے لی یہ تو | 1 |  |  |  |  |  |  |  |  |  |  |
|  | دکی بھائی یہ نوید ہے ہی چوتیا | 1 |  |  |  |  |  |  |  |  |  |  |
|  | دکی بھائی یہ ہے ہی چوتیا | 1 |  |  |  |  |  |  |  |  |  |  |
|  | دکی تم نے بہت اچھا کام کیا شام شروع میں بہت اچھا تھا پر گانڈو کو اب پتہ نہں کیا ہوگیا ہے | 1 |  |  |  |  |  |  |  |  |  |  |
|  | دکی چھوڑو اس چوتیا کو یہ لوڑا آدمی ہے کسی نہ کام کا | 1 |  |  |  |  |  |  |  |  |  |  |
|  | دل چوری ساڈا ہو گیا کیا کریے کیا کریے | 0 |  |  |  |  |  |  |  |  |  |  |
|  | دل دو پیار میں کھیل رہا ہے | 0 |  |  |  |  |  |  |  |  |  |  |
|  | دل کر رہا ہے کہ اپنا پیج بنا کر اس ابلیس جھوٹے بکواس بہن کے لوڑے کی کچھ ایسی ویڈیوز بناؤں کہ مزہ آجائے | 1 |  |  |  |  |  |  |  |  |  |  |
|  | دل کھول لے لی شاباش بھائی یہ پاکستانی اچکزئی کے ٹٹے جیسے منہ والا شام پاکستانی کی بےعزتی کرتا ہے بھوسڑی کا پلیز پاکستانی اس کی ویڈیوز مت دیکھیں | 1 |  |  |  |  |  |  |  |  |  |  |
|  | دلے جیسے الفاظ کیوں نہیں استعمال کرتا تو بے غیرت انسان | 1 |  |  |  |  |  |  |  |  |  |  |
|  | دلے شلے کی فکر نہں کرنی | 1 |  |  |  |  |  |  |  |  |  |  |
|  | دماغ تو تمہارے پاس نہیں ویسے ہی اس چوتیے ابلیس کو لائیک کرنے کی بجائے مر جاؤ | 1 |  |  |  |  |  |  |  |  |  |  |
|  | دنچک پوجا کی نئی گانے پر ویڈیو بنا نا یار | 0 |  |  |  |  |  |  |  |  |  |  |
|  | دنچک پوجا نے نئی ویڈیو ڈالی ہے سیلفی لینے میں نے اج بھائی پلیز روسٹ کردو | 0 |  |  |  |  |  |  |  |  |  |  |
|  | دنچک پوجا واپس آگئی ہے | 0 |  |  |  |  |  |  |  |  |  |  |
|  | دنچک کا نیا گانا پلیز اس کو روسٹ کریں | 0 |  |  |  |  |  |  |  |  |  |  |
|  | دنیا ک سب سے بڑا دھندا لوگ مذہب کے نام پر چوتیا بنا رہے ہیں | 1 |  |  |  |  |  |  |  |  |  |  |
|  | دنیا کے چوتیے ترین بندے کا ایوارڈ آپ کو جاتا ہے جناب | 1 |  |  |  |  |  |  |  |  |  |  |
|  | دنیا کی سب سے اچھی ویڈیو ہے | 0 |  |  |  |  |  |  |  |  |  |  |
|  | دھوتا کون ہاتھ سے ہیں پھر | 0 |  |  |  |  |  |  |  |  |  |  |
|  | دو بھی بہت اچھی ہے میں آپ کو پسند کرتا ہوں | 0 |  |  |  |  |  |  |  |  |  |  |
|  | دوستو ایک بار ضرور دیکھو | 0 |  |  |  |  |  |  |  |  |  |  |
|  | دوستوں میری اس ویڈیو کو اک بار لازمی دیکھیں اور مجھے بتائیں کہ میں ایسی ویڈیوز بناؤں کہ نہ بناؤں | 0 |  |  |  |  |  |  |  |  |  |  |
|  | دوستوں میری ویڈیو کو دیکھیں اچھی ہے نا | 0 |  |  |  |  |  |  |  |  |  |  |
|  | دوسرا گانا اور ڈانس بہت اچھا تھا | 0 |  |  |  |  |  |  |  |  |  |  |
|  | دوسری ویڈیوز سے اچھی ویڈیو نہیں ہے | 0 |  |  |  |  |  |  |  |  |  |  |
|  | دونوں چوتیے پاگل ہیں میں نے ان دونوں کو ان سبسکرائب کردیا ہے | 1 |  |  |  |  |  |  |  |  |  |  |
|  | دیپک مہتا میرا خیال ہے کہ کیری کی گانڈ کو روسٹ کرنا چاہیے | 1 |  |  |  |  |  |  |  |  |  |  |
|  | دیکھ بھائی تو لوگوں پر تنقید نہ کر وہ کچھ بھی کریں پر تو اپنا کام جاری رکھ وہ خبیس انسان ہے ہی ایسا | 1 |  |  |  |  |  |  |  |  |  |  |
|  | دیکھ کچھ اور رہا ہوتا ہے اور سن کچھ اور رہا ہوتا ہے ویڈیو کا تو کوئی ایک پوائنٹ ہے ہی نہں ان سب نے مل کر ہم سب کو پھدو بنایا ہوا ہے | 1 |  |  |  |  |  |  |  |  |  |  |
|  | دیکھ کر میں نے یہ سوچا کہ کون ہیں یہ لوگ اور کہاں سے آ جاتے ہیں | 0 |  |  |  |  |  |  |  |  |  |  |
|  | دیکھ لے گا یہ چٹنی | 0 |  |  |  |  |  |  |  |  |  |  |
|  | دیکھ یار اس ویڈیو کی رینکنگ دیکھ کتنے لوگوں نے اس کو پسند کیا گا لگتا ہے لوگوں کو سمجھ آگئی ہے اس سالے گانڈو کی | 1 |  |  |  |  |  |  |  |  |  |  |
|  | دیکھو یہ آدمی گے ہے | 0 |  |  |  |  |  |  |  |  |  |  |
|  | دین جارنا بھائی عائیشہ ایک وائنر ہے اور کیری ایک روسٹر ہے کہاں سے کہاں موازنہ کر رہا ہے | 0 |  |  |  |  |  |  |  |  |  |  |
|  | دیوازگرنگ کیا تم جاگ گئے ہو یا نہیں | 0 |  |  |  |  |  |  |  |  |  |  |
|  | دیور جی ستا لیول ہوگیا | 0 |  |  |  |  |  |  |  |  |  |  |
|  | ڈارک پولر ماں چودا | 1 |  |  |  |  |  |  |  |  |  |  |
|  | ڈاکٹر ترکی گانڈو | 1 |  |  |  |  |  |  |  |  |  |  |
|  | ڈاکٹر ترکی گانڈو چل دفعہ ہو گانڈو | 1 |  |  |  |  |  |  |  |  |  |  |
|  | ڈالرز چوتیا سالا | 1 |  |  |  |  |  |  |  |  |  |  |
|  | ڈرامے کا سکرپٹ تو اس نے بین کر دیا کہیں سالا یہ ہم کو چوتیا تو نہں بنا رہا | 1 |  |  |  |  |  |  |  |  |  |  |
|  | ڈیٹا اپ ڈیٹ ہونے میں کچھ ٹائم لگتا ہے | 0 |  |  |  |  |  |  |  |  |  |  |
|  | ذرا بھائی ان چوتیوں کے ساتھ ایسے ہی ہونا چاہیے لو یو کراچی سے | 1 |  |  |  |  |  |  |  |  |  |  |
|  | ذہین بنا دیا سیکورٹی گارڈ نے کیری کو | 0 |  |  |  |  |  |  |  |  |  |  |
|  | رات کو دارو پہ ناچتے مر جاتے ہیں | 0 |  |  |  |  |  |  |  |  |  |  |
|  | راج تیواری ہے بھائی صیح میں | 0 |  |  |  |  |  |  |  |  |  |  |
|  | رام رحیم بہت پیارا ہے | 0 |  |  |  |  |  |  |  |  |  |  |
|  | رضوی گانڈو کی گانڈ پر لات مار کے نکالنا چاہیے پاکستان سے | 1 |  |  |  |  |  |  |  |  |  |  |
|  | رقم اور ویورز کے چکر میں چتیاپے کی ویڈیو بھی اس نے شروع کردی تو ابھی تو میری گانڈ جل گئی | 1 |  |  |  |  |  |  |  |  |  |  |
|  | رنڈی مادرچود ہے یہ فراگی اور شام مادر چود | 1 |  |  |  |  |  |  |  |  |  |  |
|  | رولا دیا بھائی ویڈیو سے | 0 |  |  |  |  |  |  |  |  |  |  |
|  | رومو نو بھگا دیا | 0 |  |  |  |  |  |  |  |  |  |  |
|  | روہت بھائی کبھی بوڑھا نہیں ہوتا تو کون بوڑھا ہوتا ہے | 0 |  |  |  |  |  |  |  |  |  |  |
|  | روہت بھائی میرا خیال ہے کہ آپ کو اور ویڈیوز بنانی چاہیں | 0 |  |  |  |  |  |  |  |  |  |  |
|  | روہت کمار میں نے اس کو پہلے سے ہی ان سبسکرائب کردیا ہے مجھے لگا کہ یہ بہن چود چوتیا جھوٹی ویڈیوز بناتا ہے | 1 |  |  |  |  |  |  |  |  |  |  |
|  | ریا کوسٹا پلیز سبسکرائب میرا چینل | 0 |  |  |  |  |  |  |  |  |  |  |
|  | زبردست بھائی اس کی ایسے ہی بجاؤ بھڑوا چوتیا ہے سالا یہ | 1 |  |  |  |  |  |  |  |  |  |  |
|  | زبردست بھائی شام کی گانڈ پھاڑ دی آپ نے واہ بھائی واہ | 1 |  |  |  |  |  |  |  |  |  |  |
|  | زبردست یار اسکی بہن کو اگر بیچ میں لا ہی رہا ہے تو اسکی ماں اور بہن کو بھی چود دو پورے خاندان کو | 1 |  |  |  |  |  |  |  |  |  |  |
|  | زرداری لوگ بھٹو دشمن ہیں اس کھسرے زرداری پہ لعنت | 1 |  |  |  |  |  |  |  |  |  |  |
|  | زلیل انسان کیا بکواس ہے یہ | 1 |  |  |  |  |  |  |  |  |  |  |
|  | زمینی سیارہ اپنے باپ کا نام مت بتا | 0 |  |  |  |  |  |  |  |  |  |  |
|  | زندگی ہے چھوٹی تھوڑا پیار کرلے اور فضل اور شام گانڈو ہیں | 1 |  |  |  |  |  |  |  |  |  |  |
|  | زہر لگتا ہے بہن چود پتہ نہیں کون سے لوگ ہیں جو فائن ہیں اس چوتیے کے | 1 |  |  |  |  |  |  |  |  |  |  |
|  | زہر لونڈا ارے بھیا | 0 |  |  |  |  |  |  |  |  |  |  |
|  | زہر ہے پیار ہے تیرا چما یہ وہ گانا ہے میرے دوست | 0 |  |  |  |  |  |  |  |  |  |  |
|  | زیادہ بے عزتی کر دی سب کی | 0 |  |  |  |  |  |  |  |  |  |  |
|  | زیی ٹیوب تیرنے والا پول | 0 |  |  |  |  |  |  |  |  |  |  |
|  | سارا ٹٹی ڈرامہ گندی چالو جھوٹی مکار | 1 |  |  |  |  |  |  |  |  |  |  |
|  | سارے چوتیے ہیں سب ادھر | 1 |  |  |  |  |  |  |  |  |  |  |
|  | سارے ہی چوتیا ہیں | 1 |  |  |  |  |  |  |  |  |  |  |
|  | ساسی تم نے بالکل صیح کہا | 0 |  |  |  |  |  |  |  |  |  |  |
|  | سال کا ہے بھڑوے بڑوں سے کیوں پنگے لے رہا ہے اپنی کریلے جیسی شکل دیکھ لے میں دفعہ ہو جاؤں گا پر اپنے ابا شام کے ٹٹے اٹھانے بند کر | 1 |  |  |  |  |  |  |  |  |  |  |
|  | سالا بہت گندہ آدمی ہےیہ شام | 1 |  |  |  |  |  |  |  |  |  |  |
|  | سالا بہت مزیدار ویڈیو ہے | 1 |  |  |  |  |  |  |  |  |  |  |
|  | سالا بہن چود کتے کا بچہ یہ بھوسڑی کا حجاب | 1 |  |  |  |  |  |  |  |  |  |  |
|  | سالا چوتیا ہے شام گانڈو | 1 |  |  |  |  |  |  |  |  |  |  |
|  | سالا چوتیا ہے کیری | 1 |  |  |  |  |  |  |  |  |  |  |
|  | سالا سچ تو یہ ہے کہ تو نے ہی اس کو مشہور کر دیا | 0 |  |  |  |  |  |  |  |  |  |  |
|  | سالا نومان خان بکواس ہے | 1 |  |  |  |  |  |  |  |  |  |  |
|  | سالا ہے ہی بھوسڑی کا دلا ہے بہن چود کیری | 1 |  |  |  |  |  |  |  |  |  |  |
|  | سالگرہ مبارک ہو جناب پلیز نوٹنکی کی دکان پر بھی ویڈیو بناؤ | 0 |  |  |  |  |  |  |  |  |  |  |
|  | سالگرہ مبارک ہو کیری بھائی | 0 |  |  |  |  |  |  |  |  |  |  |
|  | سالگرہ مبارک ہو کیری بھائی پارٹی کب دے رہے ہو | 0 |  |  |  |  |  |  |  |  |  |  |
|  | سالے بہن چود اسے کہتا ہے کہ تو نے سکرپٹڈ الفاظ بین کیے ہوئے ہیں لعنت ہے تیری شکل پر | 1 |  |  |  |  |  |  |  |  |  |  |
|  | سالے حرامی مسلمان کی ترقی دیکھی نہیں جاتی | 1 |  |  |  |  |  |  |  |  |  |  |
|  | سالے کتے کیری تو لاتا کہاں سے ہے ایسے لوگوں کی ویڈیوز | 1 |  |  |  |  |  |  |  |  |  |  |
|  | سالی بےسری میں گالی نہیں نکالنا چاہتا لیکن اگر نکال دی تو پڑھتے پڑھتے تھک جاتا | 1 |  |  |  |  |  |  |  |  |  |  |
|  | سب سے اچھا حصہ پھاڑو کردا تھا آپ کا | 0 |  |  |  |  |  |  |  |  |  |  |
|  | سب سے اچھا حصہ وہ تھا جب کیری ڈانس کر رہا تھا | 0 |  |  |  |  |  |  |  |  |  |  |
|  | سب سے اچھی ویڈیو کیری بھائی | 0 |  |  |  |  |  |  |  |  |  |  |
|  | سب سے بڑا بھوسڑی کا شام ادریس ہے | 1 |  |  |  |  |  |  |  |  |  |  |
|  | سب سے بڑے چوتیے جھوٹے سالے | 1 |  |  |  |  |  |  |  |  |  |  |
|  | سب سے پہلے تم لوگوں کی ماں بہن کروں گا دوسرا اگر تم شام کو سپورٹ کرتے ہو تو گانڈ مروا کر مر جاؤ | 1 |  |  |  |  |  |  |  |  |  |  |
|  | سب سے پہلے تمہاری چودیں گے پھیر اس کی گانڈ ماریں گے | 1 |  |  |  |  |  |  |  |  |  |  |
|  | سب کے لیے شام ایک چوتیا بندہ ہے اور سب سے بڑا جھوٹا ہے | 1 |  |  |  |  |  |  |  |  |  |  |
|  | سب لوگ دیکھ رہے چودے ہو چودے ہو جاؤ چودے ہو ہاہاہاہا | 1 |  |  |  |  |  |  |  |  |  |  |
|  | سبزی منڈی مال روڈ شام ابلیس مادرچود | 1 |  |  |  |  |  |  |  |  |  |  |
|  | سبسکرائب کر لیا شکریہ | 0 |  |  |  |  |  |  |  |  |  |  |
|  | سبسکرائب کرنے میں تھوڑی مدت کم کردو سبسکرائب کردو پلیز | 0 |  |  |  |  |  |  |  |  |  |  |
|  | سبسکرائب کریں میرا چینل کو اور دوسروں کو بھی کہیں | 0 |  |  |  |  |  |  |  |  |  |  |
|  | سچ کہا بھائی یہ بڑا بہن چود ہے کتا | 1 |  |  |  |  |  |  |  |  |  |  |
|  | سچی بھائی کتنے دن سے انتظار کررہا تھا تمہارا کیوں تو اس بہن کے لوڑے کی وجہ سے پریشان ہے | 1 |  |  |  |  |  |  |  |  |  |  |
|  | سچی وہ کسی اور سیارے سے تعلق رکھتی ہے | 0 |  |  |  |  |  |  |  |  |  |  |
|  | سخت چوتیا ہے یہ اکثر ویڈیو کاپی ہوتی ہیں گوروں کی | 1 |  |  |  |  |  |  |  |  |  |  |
|  | سڑک والا بہت اچھا تھا | 0 |  |  |  |  |  |  |  |  |  |  |
|  | سکائم ابلیس کو اٹھانے والے آگئے ہیں چوتیا حجاب | 1 |  |  |  |  |  |  |  |  |  |  |
|  | سکس پیک کا بادشاہ اوہ میرے خدا سلمان خان بھی شرمائے | 0 |  |  |  |  |  |  |  |  |  |  |
|  | سکون آگیا کوئی تو بولا ان چتو پر جانی میں تمہارا فائن ہو گیا | 1 |  |  |  |  |  |  |  |  |  |  |
|  | سلم ویڈیوز میرے اسی خوشی میں لاکھوں بار دکی کی گانڈ مار لے | 1 |  |  |  |  |  |  |  |  |  |  |
|  | سلمان کی ماں کا | 0 |  |  |  |  |  |  |  |  |  |  |
|  | سلینا بولنے والی آواز مست ہے تیری | 0 |  |  |  |  |  |  |  |  |  |  |
|  | سمجھ نہیں آتی سالا ہر گھنٹے کے بعد ایک نئی ویڈیو ڈالتا ہے اور کہتا ہے میری آواز جانے والی ہے چوتیا سالا | 1 |  |  |  |  |  |  |  |  |  |  |
|  | سمن کس کو بھائی میں نے فیس بک کو روسٹ کیا ہے دیکھ لینا ایک بار مزہ آئیگا | 0 |  |  |  |  |  |  |  |  |  |  |
|  | سندر زبردست | 0 |  |  |  |  |  |  |  |  |  |  |
|  | سندیپ ہے ہی چوتیا | 1 |  |  |  |  |  |  |  |  |  |  |
|  | سنگھ وہ پاگل نہیں ہے کہ گالیاں دے | 1 |  |  |  |  |  |  |  |  |  |  |
|  | سنی تو گانڈؤں کی ملکہ اور اجے چوتیوں کا بادشاہ ہے | 1 |  |  |  |  |  |  |  |  |  |  |
|  | سنی کی گانڈ میں کھوتے کا لن | 1 |  |  |  |  |  |  |  |  |  |  |
|  | سورج بھائی میرا چینل کو سپورٹ کرو | 0 |  |  |  |  |  |  |  |  |  |  |
|  | سوروبا سوتر نہیں ہوں اس لیئے تو خوش ہوں | 0 |  |  |  |  |  |  |  |  |  |  |
|  | سوشل میڈیا پر اس کی گانڈ مار دوں گا بہن چود | 1 |  |  |  |  |  |  |  |  |  |  |
|  | سیٹھی تو اپنا پتہ کروا تیری گانڈ میں کیڑا ہے | 1 |  |  |  |  |  |  |  |  |  |  |
|  | شاباش بھائی صیح ثابت کیا ہے کہ یہ بہت بڑا دلا ہے | 1 |  |  |  |  |  |  |  |  |  |  |
|  | شاباش بھائی صیح کر کے مار دی اس گانڈو کی بجا کر رکھ دی اور اوپر سے اتنی اچھی ویڈیو | 1 |  |  |  |  |  |  |  |  |  |  |
|  | شاباش بیٹا تم ٹھیک ہو بھائی | 0 |  |  |  |  |  |  |  |  |  |  |
|  | شاباش دکی بھائی تم نے ادریس کی گانڈ مار لی اور حجاب کی بھی | 1 |  |  |  |  |  |  |  |  |  |  |
|  | شادی کر لے ویسے شام بہت بڑا دلا ہے سالا دلا کتا | 1 |  |  |  |  |  |  |  |  |  |  |
|  | شادی کرلے کرلے بھوسڑی کے فراگی تو ملے گی نہں ایسے تو اس کے ساتھ ناجائز بچے نکالتا رہے گا | 1 |  |  |  |  |  |  |  |  |  |  |
|  | شادی کرلے کون ہنسا کون ہنسا جلدی بتا بھڑوے میں اس کی گانڈ میں اپنے پاؤں کا انگوٹھا گھسا دوں | 1 |  |  |  |  |  |  |  |  |  |  |
|  | شام میرا لنڈ سے معافی مانگو دکی بھائی مہربانی | 1 |  |  |  |  |  |  |  |  |  |  |
|  | شام ابلیس بھوسڑی کے | 1 |  |  |  |  |  |  |  |  |  |  |
|  | شام ادریس چوتیا کی پھاڑ دی تم بہت اچھے ہو بھائی | 1 |  |  |  |  |  |  |  |  |  |  |
|  | شام ادریس چوتیا ہے سالا | 1 |  |  |  |  |  |  |  |  |  |  |
|  | شام ادریس دلے تو یوٹیوب چھوڑ کر چلا جاتا ہے بھڑوے | 1 |  |  |  |  |  |  |  |  |  |  |
|  | شام ادریس رنڈی کا بچہ ہے | 1 |  |  |  |  |  |  |  |  |  |  |
|  | شام ادریس کو بس سارے چوتیا دیکھتے ہیں | 1 |  |  |  |  |  |  |  |  |  |  |
|  | شام ادریس کی ماں کی چوت | 1 |  |  |  |  |  |  |  |  |  |  |
|  | شام ادریس ہے ہی چوتیا | 1 |  |  |  |  |  |  |  |  |  |  |
|  | شام ادیس چوتیا بہن کا لوڑا | 1 |  |  |  |  |  |  |  |  |  |  |
|  | شام ادیس کی گانڈ میں سریا | 1 |  |  |  |  |  |  |  |  |  |  |
|  | شام اور فرگی دونوں چوتیے ہیں بہن چود | 1 |  |  |  |  |  |  |  |  |  |  |
|  | شام ایک دلا گانڈو ہے | 1 |  |  |  |  |  |  |  |  |  |  |
|  | شام آؤ کبھی خوشبو لگا کر پاکستان بتاتے ہیں تم کو کہ کون سب سے بڑا چوتیا ہے | 1 |  |  |  |  |  |  |  |  |  |  |
|  | شام بہن چود مادر چود کبھی آ پاکستان تو تیری گانڈ میں سپورٹ ڈالتا ہوں | 1 |  |  |  |  |  |  |  |  |  |  |
|  | شام بہن کے لوڑے یاد کر تیرے سب سے زیادہ سبسکرائبر پاکستان سے ہی ہیں | 1 |  |  |  |  |  |  |  |  |  |  |
|  | شام بھائی بہت صیح کیا آپ نے | 0 |  |  |  |  |  |  |  |  |  |  |
|  | شام بھوسڑی کا | 1 |  |  |  |  |  |  |  |  |  |  |
|  | شام ٹٹہ بہن چود | 1 |  |  |  |  |  |  |  |  |  |  |
|  | شام جیسا اور کوئی چوتیا نہں آیا | 1 |  |  |  |  |  |  |  |  |  |  |
|  | شام چوتیا ہے اور تم اس کے لیے ایک کتیا ہو | 1 |  |  |  |  |  |  |  |  |  |  |
|  | شام چوتیا ہے ایک بہت بڑا چوتیا | 1 |  |  |  |  |  |  |  |  |  |  |
|  | شام چوتیا ہے تم پھر بھی اس سے بہتر ہو | 1 |  |  |  |  |  |  |  |  |  |  |
|  | شام دنیا کا سب سے بڑا چوتیا بندہ ہے | 1 |  |  |  |  |  |  |  |  |  |  |
|  | شام سے بڑا چوتیا آج تک کبھی نہیں دیکھا اس کی ویڈیوز دیکھنے والے بھڑوے کا نہ کوئی پیچ لائیک کرتا ہے اور نہ اس کو | 1 |  |  |  |  |  |  |  |  |  |  |
|  | شام کا فائن تھا پر وہ چوتیا ہے | 1 |  |  |  |  |  |  |  |  |  |  |
|  | شام کے چوتیے فائن نے اگر یہاں کمنٹس کیے تو ان کی ماں چود دوں گا | 1 |  |  |  |  |  |  |  |  |  |  |
|  | شام کی شام کے وقت میں گانڈ مار دی ہے | 1 |  |  |  |  |  |  |  |  |  |  |
|  | شام کی گانڈ میں ڈنڈا | 1 |  |  |  |  |  |  |  |  |  |  |
|  | شام کی گانڈ ہی پھاڑ دی صرف یہ چینل ہے جس پر میں روز آتا ہوں ویڈیوز دیکھنے | 1 |  |  |  |  |  |  |  |  |  |  |
|  | شام کی ماں کا بھوسڑا چوتیا سالا | 1 |  |  |  |  |  |  |  |  |  |  |
|  | شام کی ماں کی بھوسڑی | 1 |  |  |  |  |  |  |  |  |  |  |
|  | شام کی ماں کی چوت | 1 |  |  |  |  |  |  |  |  |  |  |
|  | شام کی ماں کی چوت اور فراگی کی ماں کی بھی | 1 |  |  |  |  |  |  |  |  |  |  |
|  | شام کی ماں کی چوت دلا ہے شام رنڈی کی نسل بھی پلیز پن کردوں جیو جیو | 1 |  |  |  |  |  |  |  |  |  |  |
|  | شام گانڈو سالا چوتیا مادرچود اور فراگی آجا تجھے فراگ طریقے سے کروں | 1 |  |  |  |  |  |  |  |  |  |  |
|  | شام گانڈو ہے | 1 |  |  |  |  |  |  |  |  |  |  |
|  | شام گانڈوسے تو اچھا اپنا نادر ہے اس سے زیادہ سبسکرائبر ہیں | 1 |  |  |  |  |  |  |  |  |  |  |
|  | شام مادرچود کا بچہ | 1 |  |  |  |  |  |  |  |  |  |  |
|  | شام مکمل طور پر پاگل کے بھوسڑی کا | 1 |  |  |  |  |  |  |  |  |  |  |
|  | شام نے پھچھلی ویڈیو بھی ڈنڈا سمجھ کر گانڈ میں لے لی تھی | 1 |  |  |  |  |  |  |  |  |  |  |
|  | شام ہمیشہ سے ہی چوتیا ہے | 1 |  |  |  |  |  |  |  |  |  |  |
|  | شام ہے بہن چود مادر چود بھوسڑی کا لوڑا کتی کا | 1 |  |  |  |  |  |  |  |  |  |  |
|  | شام ہے ہی گانڈو جو جدھر کھاتے ہیں ادھر ہی پیشاب کرتے ہیں | 1 |  |  |  |  |  |  |  |  |  |  |
|  | شام ویسے کی چوتیا ہے | 1 |  |  |  |  |  |  |  |  |  |  |
|  | شانایا خان یہ | 0 |  |  |  |  |  |  |  |  |  |  |
|  | شاندار اترو میوزک ایسا دل کو چھو لینے والا میوزک اور ہمیشہ کی طرح ویڈیو بھی بہت اچھی | 0 |  |  |  |  |  |  |  |  |  |  |
|  | شاندار بہت مزہ آیا | 0 |  |  |  |  |  |  |  |  |  |  |
|  | شاندار بھائی صیح کتوں والی کی آپ نے اس کی بھوسڑی کا کھبہ ٹٹہ ہے بہت اعلی | 1 |  |  |  |  |  |  |  |  |  |  |
|  | شاندار بھائی کچھ ایسے ہی جھٹکے کی امید تھی مست بھائی | 0 |  |  |  |  |  |  |  |  |  |  |
|  | شاندار دکی بھائی کیری کو لوڑے کا ٹیکہ چاہیے سب لوگ مل کر دو اس کو | 1 |  |  |  |  |  |  |  |  |  |  |
|  | شاندار شاندار شاندار میرے پاس آپ کے لیے کوئی الفاظ نہیں ہیں | 0 |  |  |  |  |  |  |  |  |  |  |
|  | شاندار کام بہت عمدہ کیری بھائی تم میرے سب سے پسندیدہ یوٹیوبرہو | 0 |  |  |  |  |  |  |  |  |  |  |
|  | شاندار کمال ہوگیا اچھا کیا اس رنڈی اور بھڑوی کی حقیقت بتا دی | 1 |  |  |  |  |  |  |  |  |  |  |
|  | شاندار کیری بھائی آپ کی ویڈیو بہت اعلی ہے | 0 |  |  |  |  |  |  |  |  |  |  |
|  | شاندار ویڈیو تھا بھائی | 0 |  |  |  |  |  |  |  |  |  |  |
|  | شاندار ویڈیو کیری واہ | 0 |  |  |  |  |  |  |  |  |  |  |
|  | شبی پھڑوے جا اپنا کام کر | 1 |  |  |  |  |  |  |  |  |  |  |
|  | شرم کر ادریس بھڑوے | 1 |  |  |  |  |  |  |  |  |  |  |
|  | شروع میں اگر ایسا ہوتا تو بہت مزہ آتا ہلیو دوستوں اور فائن یہ اس دلے کی ویڈیو ہے | 1 |  |  |  |  |  |  |  |  |  |  |
|  | شروع میں پاجامہ بھی فالتو میں زبردستی کٹا ہے | 0 |  |  |  |  |  |  |  |  |  |  |
|  | شروع میں تو آگ لگا دی گورے گورے گال تری چھاتی پر موسمی مزہ آگیا | 0 |  |  |  |  |  |  |  |  |  |  |
|  | شکر ہے ان چوتیوں کے خلاف کوئی ویڈیو آئی | 1 |  |  |  |  |  |  |  |  |  |  |
|  | شکر ہے بھائی تیری ویڈیو آئی | 0 |  |  |  |  |  |  |  |  |  |  |
|  | شکریہ بھائی تو نے سب کو آگاہ کر دیا ورنہ یہ لوگ تو صرف ٹٹے چوسنے کے لیے ہیں | 1 |  |  |  |  |  |  |  |  |  |  |
|  | شکریہ دکی بھائی تم نے سچائی بتائی سب کو شام اور فراگی گانڈ میں دے دینا | 1 |  |  |  |  |  |  |  |  |  |  |
|  | شکریہ دوبارہ آنا | 0 |  |  |  |  |  |  |  |  |  |  |
|  | شکل سے ہی چوتیا لگتا ہے یہ نفرت ہو گئی ہےمجھے اس سے | 1 |  |  |  |  |  |  |  |  |  |  |
|  | شھزدہ ہے بھائی تم نے تو تیل لگائے بناء ہی اس کی مار لی | 1 |  |  |  |  |  |  |  |  |  |  |
|  | صابن کھانا بہت بہتر ہے | 0 |  |  |  |  |  |  |  |  |  |  |
|  | صابن والا بہت ہارنی تھا | 0 |  |  |  |  |  |  |  |  |  |  |
|  | صبح بھوسڑی کا مادر چود | 1 |  |  |  |  |  |  |  |  |  |  |
|  | صرف انتظار کریں ایسا ہی ہوگا | 0 |  |  |  |  |  |  |  |  |  |  |
|  | صرف مادر چود لکھ دیا کرو بین نہں ہوگا | 1 |  |  |  |  |  |  |  |  |  |  |
|  | صیح بات ہے بھائی کسی اور کا مزاق نہں اڑانا چاہیے | 0 |  |  |  |  |  |  |  |  |  |  |
|  | صیح بات ہے مجھے تو ویسے بھی یہ پسند نہں ہے کتی نسل کا کیڑا ہے اس کی گانڈ میں | 1 |  |  |  |  |  |  |  |  |  |  |
|  | صیح بتا اس کی باتوں میں پوائنٹ ہوتا ہے یا نہیں میں نے اس کی ساری ویڈیوز دیکھی ہیں | 0 |  |  |  |  |  |  |  |  |  |  |
|  | صیح بہن چودی آپ نے اس کی اس لیے ہے یہ باہر کا ٹٹہ | 1 |  |  |  |  |  |  |  |  |  |  |
|  | صیح بول رہے ہو بھائی ان کی سب ویڈیوز چوتیاپوں سے بھری ہوئی ہیں | 1 |  |  |  |  |  |  |  |  |  |  |
|  | صیح بول رہے ہو بھائی میں نے بھی اس بہن چود کو سبسکرائب کر دیا ہے | 1 |  |  |  |  |  |  |  |  |  |  |
|  | صیح بولا بھائی بھڑوے ہیں سب بہن چود | 1 |  |  |  |  |  |  |  |  |  |  |
|  | صیح بولا بھائی ماں کی چوت شام ادریس کی | 1 |  |  |  |  |  |  |  |  |  |  |
|  | صیح بولا تو وہ سالا عوام کو چوتیا بنا رہا ہے اور عوام تو ہے ہی چوتیا اور ویسے بھی لوگ جھوٹی ایکٹنگ کرتے ہیں | 1 |  |  |  |  |  |  |  |  |  |  |
|  | صیح سچ میں ایک نمبر کا چوتیا ہے اس کا بائیکاٹ کرو | 1 |  |  |  |  |  |  |  |  |  |  |
|  | صیح سے چودا ہے اسے بہت اچھا کیا | 1 |  |  |  |  |  |  |  |  |  |  |
|  | صیح کہ رہا ہے آپ نے کیوں اس کی ماں اور بہن پر نہں بنایا شام بھوسڑی کا | 1 |  |  |  |  |  |  |  |  |  |  |
|  | صیح کہا آپ نے یہ جو کر رہے ہیں نہ تو اسلام میں ان کی اجازت ہے اورنہ ہی انسانیت میں | 0 |  |  |  |  |  |  |  |  |  |  |
|  | صیح کہا سالے بھکاری ہوتے ہیں | 1 |  |  |  |  |  |  |  |  |  |  |
|  | صیح کھیل گیا ایسے ہی ویڈیو بناؤ بھائی مزہ آگیا آج | 0 |  |  |  |  |  |  |  |  |  |  |
|  | صیح گانڈ پھاڑی آپ نے اس کی | 1 |  |  |  |  |  |  |  |  |  |  |
|  | صیح گانڈ پھاڑی ہے بھوسڑی والے کی | 1 |  |  |  |  |  |  |  |  |  |  |
|  | صیح ہے باس اچھی سوچ تھی ہماری قوم میں | 0 |  |  |  |  |  |  |  |  |  |  |
|  | صیح ہے بھائی چرس گانجا مرے کو ایک نمبر بھائی | 0 |  |  |  |  |  |  |  |  |  |  |
|  | صیح ویڈیو ہے کتنے ہفتوں کے بعد | 0 |  |  |  |  |  |  |  |  |  |  |
|  | ضرور ضرور یہ ویڈیو دیکھیں | 0 |  |  |  |  |  |  |  |  |  |  |
|  | عاصم میرے باپ نے یہی سیکھایا ہے | 0 |  |  |  |  |  |  |  |  |  |  |
|  | عاصمہ بختیاری تجھے پتہ ھے ماتا چڑہنا کیا ہوتا ہے | 0 |  |  |  |  |  |  |  |  |  |  |
|  | عالیا میں پورا پانہراما استعمال کر دوبارہ ایک بار فوٹوشاپ کا موقع دو | 0 |  |  |  |  |  |  |  |  |  |  |
|  | عالیہ بھٹ کی اشتہارمیں نئی انٹری | 0 |  |  |  |  |  |  |  |  |  |  |
|  | عجیب رنڈی کے بچے گانڈو کی نسل کوئی اور بات بول بھڑوے | 1 |  |  |  |  |  |  |  |  |  |  |
|  | عظیم کام کیا دکی بھائی تو نے شام ہے ہی بچپن سے کمینہ | 1 |  |  |  |  |  |  |  |  |  |  |
|  | عمدہ بھائی اور بھی ویڈیوز بناؤ اسی طرح کی | 0 |  |  |  |  |  |  |  |  |  |  |
|  | عمدہ کیری بھائی آپ ایسی ویڈیوز کو اپ لوڈ کرو | 0 |  |  |  |  |  |  |  |  |  |  |
|  | عمدہ مجھے یہ پسند آیا اور بناؤ ایسی ویڈیوز | 0 |  |  |  |  |  |  |  |  |  |  |
|  | عمدہ یار گانڈ مار دے اس کی | 1 |  |  |  |  |  |  |  |  |  |  |
|  | عمیر عامر وہ ساری ویڈیوز جھوٹی ہیں چوتیاپا ہے تاکہ ہم سب اس کے ساتھ آجائیں | 1 |  |  |  |  |  |  |  |  |  |  |
|  | عمیر وہ جھوٹا سال چوتیا | 1 |  |  |  |  |  |  |  |  |  |  |
|  | عیش علی تم نے بہن چود دی ہے تونے | 1 |  |  |  |  |  |  |  |  |  |  |
|  | غضب غضب غضب بھائی | 0 |  |  |  |  |  |  |  |  |  |  |
|  | غضب ک گانے گاتی ہے یار | 0 |  |  |  |  |  |  |  |  |  |  |
|  | غضب کی ذہانت ہے بہن چود | 1 |  |  |  |  |  |  |  |  |  |  |
|  | غلط بول دیا بھائی تو کیسے ہوں گے آپ سب یہ ہوگا | 0 |  |  |  |  |  |  |  |  |  |  |
|  | فالتو بکواس مت کر نہں تو پھاڑ کر تمہاری ہاتھ میں دے دوں گا | 1 |  |  |  |  |  |  |  |  |  |  |
|  | فائدہ نہں ہے بھائی یہ بہت ہی چوتیا اور کمینہ بندہ ہے | 1 |  |  |  |  |  |  |  |  |  |  |
|  | فٹا فٹی کیری بھائی | 0 |  |  |  |  |  |  |  |  |  |  |
|  | فخر محسوس ہو رہا ہے آج جو کچھ ہوا وہ کچھ چوتیا لوگوں کی وجہ سے ہوا | 1 |  |  |  |  |  |  |  |  |  |  |
|  | فراگی تیری ماں کی چوت | 1 |  |  |  |  |  |  |  |  |  |  |
|  | فراگی خود بہت بڑی چوتیا ہے کیوں کی شام کی بہن ہے بہن چود دو اس سالے کی | 1 |  |  |  |  |  |  |  |  |  |  |
|  | فراگی کی گانڈ ملے گی مجھے جلدی | 1 |  |  |  |  |  |  |  |  |  |  |
|  | فراگی ہیرا منڈی کی گشتی ہے اور شام سب سے بڑا گانڈو ہے | 1 |  |  |  |  |  |  |  |  |  |  |
|  | فضل الدین لوڑا میرا رہنے دو تم اتنی اچھی بھی نہیں ہے وہ | 1 |  |  |  |  |  |  |  |  |  |  |
|  | فل بہن چودائی ہو رہی ہے ادھر تو | 1 |  |  |  |  |  |  |  |  |  |  |
|  | فل بہن چودی مزہ آگیا بھائی جان | 1 |  |  |  |  |  |  |  |  |  |  |
|  | فل بہن چودی ہو رہی ہے ادھر | 1 |  |  |  |  |  |  |  |  |  |  |
|  | فلم کا آخر بہت تکلیف دہ تھا یار | 0 |  |  |  |  |  |  |  |  |  |  |
|  | فیس کیم پر آؤ بھڑوے جھوٹے چتو | 1 |  |  |  |  |  |  |  |  |  |  |
|  | قسم سے بھائی کمال کردیا اس بھوسڑی کے بندے کی اوقات اس کو دکھا دی تونے بہت اچھا | 1 |  |  |  |  |  |  |  |  |  |  |
|  | قسم سے دماغ خراب ہو گیا جو اپنی ملک کی عزت نہیں کرتا وہ مادرچود ہے | 1 |  |  |  |  |  |  |  |  |  |  |
|  | قسم یار کیری ہنسا ہنسا کر پاگل کردیتا ہے اور مزہ آگیا | 0 |  |  |  |  |  |  |  |  |  |  |
|  | کا سپرمین نے ایسے کہا تھا کہ چائے پی لو دوستوں | 0 |  |  |  |  |  |  |  |  |  |  |
|  | کاش یوٹیوب میں ایک ویڈیو کو ایک بار سے زیادہ بار بار لائیک کرنے کا آپشن ہوتا تو اس ویڈیو کو لائیکس دیتا | 0 |  |  |  |  |  |  |  |  |  |  |
|  | کافر بھی نہیں کرتا کس کافر کو اپنی بہن دی ہے تو نے کیا رنڈی کی اولاد بھڑوے گالیاں دے کر اپنے آپ کو اچھا سمجھتا تھا | 1 |  |  |  |  |  |  |  |  |  |  |
|  | کافی صیح ویڈیو ہے یہ | 0 |  |  |  |  |  |  |  |  |  |  |
|  | کافی وقت کے بعد غضب ویڈیو بنائی شادی کون کرے گا | 0 |  |  |  |  |  |  |  |  |  |  |
|  | کالی گھٹا نہں ڈالنی تھی تجھ کو بھائی | 0 |  |  |  |  |  |  |  |  |  |  |
|  | کبھی بھی نہیں وہ پہلے بھی بہت مشہور ہو گئی ہے | 0 |  |  |  |  |  |  |  |  |  |  |
|  | کبھی تم کو موت کو قریب سے دیکھنے کا چانس ملا ہے | 0 |  |  |  |  |  |  |  |  |  |  |
|  | کبھی مارے ہو توے سے | 0 |  |  |  |  |  |  |  |  |  |  |
|  | کتا تو خود ہے ادریس بہن چود تری ماں نوں لن واراں | 1 |  |  |  |  |  |  |  |  |  |  |
|  | کتا رنڈی کا بچہ مادر چود | 1 |  |  |  |  |  |  |  |  |  |  |
|  | کتنے چوتیے لوگ ہیں اس دنیا میں جو ابلیس کا چینل دیکھتے ہیں | 1 |  |  |  |  |  |  |  |  |  |  |
|  | کتنی بار دیکھی ہے ویڈیو بتاؤ بہن چود | 1 |  |  |  |  |  |  |  |  |  |  |
|  | کتے کہیں کے تمہارا اور کام ہی کیا ہے دوسروں کی ویڈیوز بنا کر یا تبدیل کر کے ان کو بدنام کرنا | 1 |  |  |  |  |  |  |  |  |  |  |
|  | کچھ بننے کے لیے بمبئی میں آنا پڑتا ہے پر میں تو رہتا ہی بمبئی میں ہوں ہا ہا ہا | 0 |  |  |  |  |  |  |  |  |  |  |
|  | کچھ بھی بول لیکن چوتیاپا ہی ہے | 1 |  |  |  |  |  |  |  |  |  |  |
|  | کچھ چوتیے ہیں ابھی بھی ناپسند کر رہے ہیں ہمارے چینل کو | 1 |  |  |  |  |  |  |  |  |  |  |
|  | کچھ روشنی ڈالو اس پر بھی | 0 |  |  |  |  |  |  |  |  |  |  |
|  | کچھ لوگوں کی آنکھیں نہیں کھلی ابھی تک اور تو جا کر گانڈ مروا | 1 |  |  |  |  |  |  |  |  |  |  |
|  | کر کر آخر کر دیا نا | 0 |  |  |  |  |  |  |  |  |  |  |
|  | کر کر کھا چپ رنڈی کے بچے | 1 |  |  |  |  |  |  |  |  |  |  |
|  | کرکٹ لورز کلب پھیر بھی امتحان دینا بہت ضروری ہے | 0 |  |  |  |  |  |  |  |  |  |  |
|  | کروڑ لائیکس کا ٹارگٹ کب پورا ہوگا | 0 |  |  |  |  |  |  |  |  |  |  |
|  | کڑک میرا بھائی پچھلے والا بھاری تھا الیکشن والا بھاری تھا | 0 |  |  |  |  |  |  |  |  |  |  |
|  | کس چوتیے پر ویڈیو بنا دی یار اس کے ابو کا کنڈم لیک ہو گیا تھا اور یہ اس سے پیدا ہوا تھا | 1 |  |  |  |  |  |  |  |  |  |  |
|  | کس کو چوتیا بناتا ہے تو | 1 |  |  |  |  |  |  |  |  |  |  |
|  | کس نے کیری کا پچلا چینل دیکھا ہے | 0 |  |  |  |  |  |  |  |  |  |  |
|  | کس نے میرے ہیڈفون چرائے ہیں | 0 |  |  |  |  |  |  |  |  |  |  |
|  | کسی جگہ کی سیر کی ویڈیو بنا | 0 |  |  |  |  |  |  |  |  |  |  |
|  | کسی کا کوئی رجحان نہیں واہ | 0 |  |  |  |  |  |  |  |  |  |  |
|  | کسی کام بھی ویڈیو نہیں ہے | 0 |  |  |  |  |  |  |  |  |  |  |
|  | کسی کی توجہ لینے کے لیے اور سستی شہرت کے لیے یہ بہن کا لوڑا گانڈ بھی مروا لے گا | 1 |  |  |  |  |  |  |  |  |  |  |
|  | کسی نے نوٹس کیا کہ ٹیڈی ٹھگ ہے | 0 |  |  |  |  |  |  |  |  |  |  |
|  | کمار لازمی اس ویڈیو کو دیکھویہ ویڈیو مزاحیہ ڈاکٹروں پر ہے | 0 |  |  |  |  |  |  |  |  |  |  |
|  | کمال کر دیا پھاڑ کر رکھ دی گانڈ ابلیس کی | 1 |  |  |  |  |  |  |  |  |  |  |
|  | کمال کردیا بھائی اس سالے کی گانڈ مار کے | 1 |  |  |  |  |  |  |  |  |  |  |
|  | کمال کی ایڈیٹنگ بھائی جیسے اصلی میں ہو | 0 |  |  |  |  |  |  |  |  |  |  |
|  | کمال ہے بھائی اس چوتیا کی تھوڑی اور مار اور اسکی ماں بہن ایک کر دے | 1 |  |  |  |  |  |  |  |  |  |  |
|  | کمال ہے بھائی کمال | 0 |  |  |  |  |  |  |  |  |  |  |
|  | کمیسٹری کا ردعمل بھی اتنے مشکل نہیں ہوتے | 0 |  |  |  |  |  |  |  |  |  |  |
|  | کہاں سے آتے ہیں یہ لوگ | 0 |  |  |  |  |  |  |  |  |  |  |
|  | کھسرا ہے شام ادریس بہن چود | 1 |  |  |  |  |  |  |  |  |  |  |
|  | کھوتی کے بچے عمران خان ک خلاف بکواس نہ کر گانڈو | 1 |  |  |  |  |  |  |  |  |  |  |
|  | کھیت اور جنگل میں بہت کم لوگ اپنا پریشر چھوڑنے جاتے ہیں | 0 |  |  |  |  |  |  |  |  |  |  |
|  | کوچی کامے سب سے بہتر ہے | 0 |  |  |  |  |  |  |  |  |  |  |
|  | کون پسند کرتا ہا ایس ٹی کو دیکھنا کیوں کی ویڈیوز ہمیشہ کی طرح بہت اچھی ہوتی ہیں | 0 |  |  |  |  |  |  |  |  |  |  |
|  | کون چوتیا قسم کے لوگ اس شام کی ویڈیوز کو دیکھتے ہیں | 1 |  |  |  |  |  |  |  |  |  |  |
|  | کون چوتیا ہے جو ان لائیک کر رہا ہے | 1 |  |  |  |  |  |  |  |  |  |  |
|  | کون سا گانا وہ چھوٹا لڑکا گانے لگا ہے | 0 |  |  |  |  |  |  |  |  |  |  |
|  | کون ہے یہ نعمان خان کی گانڈ کھجانے والا بھوسڑی والا | 1 |  |  |  |  |  |  |  |  |  |  |
|  | کوئی بھی کیری کو ہرا نہیں سکتا | 0 |  |  |  |  |  |  |  |  |  |  |
|  | کوئی بھی میرا کمنٹ سیکشن گندا نہ کرے | 0 |  |  |  |  |  |  |  |  |  |  |
|  | کوئی شک نہں کہ بہن چود کی گانڈ مار دی سب نے | 1 |  |  |  |  |  |  |  |  |  |  |
|  | کوئی مجھے مار ڈالو | 0 |  |  |  |  |  |  |  |  |  |  |
|  | کوئی مسئلہ نہیں اشیش ہمہشہ سے اچھا ہے | 0 |  |  |  |  |  |  |  |  |  |  |
|  | کوئی نیا ویڈیو بناؤ | 0 |  |  |  |  |  |  |  |  |  |  |
|  | کیا ایڈیٹینگ ہے مان گئے بھائی | 0 |  |  |  |  |  |  |  |  |  |  |
|  | کیا آپ اس کے ساتھ کچھ کر سکتے ہو | 0 |  |  |  |  |  |  |  |  |  |  |
|  | کیا آپ بگ باس اور سوامی پر ویڈیو بنا سکتے ہو | 0 |  |  |  |  |  |  |  |  |  |  |
|  | کیا بکواس آدمی ہے یہ | 1 |  |  |  |  |  |  |  |  |  |  |
|  | کیا بکواس ہے کیوں ان کو الزام دیتے ہو سارے باہر جا کر گانڈ مرواتے ہیں کس کی گانڈ محفوظ ہے ادھر | 1 |  |  |  |  |  |  |  |  |  |  |
|  | کیا بہن چودی ہے | 1 |  |  |  |  |  |  |  |  |  |  |
|  | کیا بہن چودی ہے یار | 1 |  |  |  |  |  |  |  |  |  |  |
|  | کیا بھنگی وہ اصلی میں ہے | 0 |  |  |  |  |  |  |  |  |  |  |
|  | کیا بولوں بھائی مست ویڈیو ہے پبجی والے نے دیکھ لیا تو کیا بولوں | 0 |  |  |  |  |  |  |  |  |  |  |
|  | کیا بے کار ویڈیو ہے بھائی میں ان سبکرائب | 0 |  |  |  |  |  |  |  |  |  |  |
|  | کیا پاگل پن ہے کسی کے مذہب پر گالی نکالنےکا | 0 |  |  |  |  |  |  |  |  |  |  |
|  | کیا تم پچھلا گانا بنا سکتے ہو | 0 |  |  |  |  |  |  |  |  |  |  |
|  | کیا تم مارول کو ہرا سکتے ہو | 0 |  |  |  |  |  |  |  |  |  |  |
|  | کیا تم نے کبھی ناروتو کو دیکھا ہے | 0 |  |  |  |  |  |  |  |  |  |  |
|  | کیا ٹیلنٹ ہے میرے بھائی | 0 |  |  |  |  |  |  |  |  |  |  |
|  | کیا ریپ کیا ہے بھائی نے ایک نمب یار | 0 |  |  |  |  |  |  |  |  |  |  |
|  | کیا کبھی تم نے ہاتھ سے کام کرنے کی کوشش کی ہے | 0 |  |  |  |  |  |  |  |  |  |  |
|  | کیا کر رہا ہے ابھی تک میرے بھائی ابھی تک رجحان میں آیا ہے | 0 |  |  |  |  |  |  |  |  |  |  |
|  | کیا گانڈ ماری ہے یار | 1 |  |  |  |  |  |  |  |  |  |  |
|  | کیا لوگ ہو تم ایک جھوٹ پر کھڑے ہو گئے ہو لگتا ہے کہ اس گانڈو کو بین کرواؤ گے تم سب مل کر | 1 |  |  |  |  |  |  |  |  |  |  |
|  | کیا میں اکیلا ہوں جو یہ سمجھتا ہوں کہ وہ دونوں کیری کے ویڈیو کلپ سے غائب ہو گئے ہیں | 0 |  |  |  |  |  |  |  |  |  |  |
|  | کیا میوزک ہے پلیز | 0 |  |  |  |  |  |  |  |  |  |  |
|  | کیا ہو گیا ہے اس قوم کو بہن چودو | 1 |  |  |  |  |  |  |  |  |  |  |
|  | کیا ہوا تھا ابے تم کو کیوں اتنا دیر سے ویڈیو ڈال رہے ہو | 0 |  |  |  |  |  |  |  |  |  |  |
|  | کیا ہوگا اگر کیری کا ریپ ہو جائے | 1 |  |  |  |  |  |  |  |  |  |  |
|  | کیا ہوگیا ہے بھائی تیرا ہومر کہاں گیا | 0 |  |  |  |  |  |  |  |  |  |  |
|  | کیا یار شام ادریس کی گانڈ کے پیچھے لگے ہو آپ تخلیقی بنو اور نئی ویڈیوز لے کر آؤ | 1 |  |  |  |  |  |  |  |  |  |  |
|  | کیا یہ حقیقی کہانی ہے | 0 |  |  |  |  |  |  |  |  |  |  |
|  | کیا یہ دونوں پاگل خانہ سے بھاگ کے تو نہیں آئے | 0 |  |  |  |  |  |  |  |  |  |  |
|  | کیر جتنی دیر سے ویڈیو بنائی ہے اتنی اچھی ہے | 0 |  |  |  |  |  |  |  |  |  |  |
|  | کیری اس بار تم نے عالیہ کو پریشان والی بات کر کے مجھے ناپسند کرنے پر مجبور کردیا | 0 |  |  |  |  |  |  |  |  |  |  |
|  | کیری اس کے لیے کتنا انتظار کر رہے تھے | 0 |  |  |  |  |  |  |  |  |  |  |
|  | کیری اس ویڈیو کا کون سا موضوع آپ نے اٹھایا ہے | 0 |  |  |  |  |  |  |  |  |  |  |
|  | کیری اس ویڈیو کو دیکھو اگر دیکھو گے تو کامیاب رہو گے | 0 |  |  |  |  |  |  |  |  |  |  |
|  | کیری اس ویڈیو کے آخر میں کون سا پنجابی گانا تھا | 0 |  |  |  |  |  |  |  |  |  |  |
|  | کیری اسامہ بن لادن کی طرح لگ رہا تھا | 0 |  |  |  |  |  |  |  |  |  |  |
|  | کیری اور اجے بھائی بہت عمدہ ویڈیو تھی فالتو کے کمنٹس پر کمانڈ مت دو اور کام جاری رکھو | 0 |  |  |  |  |  |  |  |  |  |  |
|  | کیری اور اجے کی ماں کا بھوسڑی کا چوتیا سالا | 1 |  |  |  |  |  |  |  |  |  |  |
|  | کیری اور پوجا دونوں پروموشن کو چوری کر رہے ہیں | 0 |  |  |  |  |  |  |  |  |  |  |
|  | کیری ایک چوتیا اور گانڈو انسان ہے کتے کی شکل والاشام چوتیا کی گانڈ اتنی مارو کہ اس کی گانڈ سےخون نکل جائے | 1 |  |  |  |  |  |  |  |  |  |  |
|  | کیری ایک فیک اکاؤنٹ ہے اس کی رپورٹ کر | 0 |  |  |  |  |  |  |  |  |  |  |
|  | کیری آپ اپنے آپ کو بہتر کرو | 0 |  |  |  |  |  |  |  |  |  |  |
|  | کیری آگیا نئی ویڈٰیو | 0 |  |  |  |  |  |  |  |  |  |  |
|  | کیری بریک اپ کے لیے کوئی مزاحیہ ویڈیو بناؤ | 0 |  |  |  |  |  |  |  |  |  |  |
|  | کیری بھائی اب روسٹ بند کر اور کچھ تخلیقی ویڈیو بنا ویسے یہ ویڈیو بھی مست ہے | 0 |  |  |  |  |  |  |  |  |  |  |
|  | کیری بھائی اب کچھ تگڑا لانا ہے تو کچھ وقت لے لے میں چاہتا ہوں کہ اگے پہ لاکھوں دیکھنے والے ہوں | 0 |  |  |  |  |  |  |  |  |  |  |
|  | کیری بھائی اتنے دنوں کے بعد آپ واپس آگئے میں بہت خوشی ہوں | 0 |  |  |  |  |  |  |  |  |  |  |
|  | کیری بھائی اس بار تو سٹرائیک ہٹ گئی اگلی بار سوچ سمجھ کر روسٹ کرنا ویڈیو یہ نہ ہو کہ تو خود ہی روسٹ ہوجائے اڑنا اچھی بات ہے لیکن پر سیدھا زمین پر ہی ہونے چاہیں | 0 |  |  |  |  |  |  |  |  |  |  |
|  | کیری بھائی اس کےچیینل کا نام کیا ہے میں اس کی ماں چود ڈالوں گا | 1 |  |  |  |  |  |  |  |  |  |  |
|  | کیری بھائی انڈین کے پرانے ہیروزپر بھی ویڈیو بناؤ | 0 |  |  |  |  |  |  |  |  |  |  |
|  | کیری بھائی آپ بہت اچھے ہو | 0 |  |  |  |  |  |  |  |  |  |  |
|  | کیری بھائی آپ کون سی کلاس میں پڑھتے ہو | 0 |  |  |  |  |  |  |  |  |  |  |
|  | کیری بھائی آج تیرے پیج پر اس چوتیا کے چوتیا فائن آئیں گے دھیان کرنا | 1 |  |  |  |  |  |  |  |  |  |  |
|  | کیری بھائی بالی ووڈ فلم کا ایک ویڈیو بناؤ میں آپ کا بہت بڑا فائن ہوں | 0 |  |  |  |  |  |  |  |  |  |  |
|  | کیری بھائی پلیز دنچک پوجا کو بھی روسٹ کرو نا | 0 |  |  |  |  |  |  |  |  |  |  |
|  | کیری بھائی تم اچھے ہو مہربانی کر کے اترانگی یاری چینل | 0 |  |  |  |  |  |  |  |  |  |  |
|  | کیری بھائی تم بہت اچھے ہو شام ہم سب کو چوتیا بنا رہا ہے آپ نے بہت اچھا کیا اس کے ساتھ لو یو | 1 |  |  |  |  |  |  |  |  |  |  |
|  | کیری بھائی تم بہت عظیم ہو تم نے تو دنیا کی گانڈ پھاڑ دی واقعی میں | 0 |  |  |  |  |  |  |  |  |  |  |
|  | کیری بھائی تم کچھ اچھا کرنا | 0 |  |  |  |  |  |  |  |  |  |  |
|  | کیری بھائی تم ہمیشہ سب سے بہتر ہو تمہارا نیا کام بہت پسند آیا اس کو جاری رکھو | 0 |  |  |  |  |  |  |  |  |  |  |
|  | کیری بھائی تھوڑا موٹا ہو گیا ہے یا نیہں | 0 |  |  |  |  |  |  |  |  |  |  |
|  | کیری بھائی تو ہمیشہ لائیک کی بھیک کوں مانگتا رہتا ہے گھر والے کھانا نہیں دیتے کیا ہا ہا | 0 |  |  |  |  |  |  |  |  |  |  |
|  | کیری بھائی جی اگر موقع ملا تھا تم سے بات کرنے کا مگر ٹائم ضائع ہو گیا | 0 |  |  |  |  |  |  |  |  |  |  |
|  | کیری بھائی زبردست ہو | 0 |  |  |  |  |  |  |  |  |  |  |
|  | کیری بھائی شام کسی رنڈی کا بچہ ہے مادرچود سالا یہ پانے باپ کا نہں بلکہ کسی پڑوسی کی لن کی پیداوار ہے | 1 |  |  |  |  |  |  |  |  |  |  |
|  | کیری بھائی کتنے دن ہو گئے کب نئی ویڈیو ڈالو گے | 0 |  |  |  |  |  |  |  |  |  |  |
|  | کیری بھائی لے لے تو اس گانڈو کی | 1 |  |  |  |  |  |  |  |  |  |  |
|  | کیری بھائی ملائیکہ نے بی بی کی ویڈیوز کو سٹرائیک لگوا دی ہے اور اس کا چینل بند ہو گیا ہے | 0 |  |  |  |  |  |  |  |  |  |  |
|  | کیری بھائی مہربانی کر کے تاؤ کو لا نا | 0 |  |  |  |  |  |  |  |  |  |  |
|  | کیری بھائی میرا نام بھی اپنے فائن میں شامل کر لو | 0 |  |  |  |  |  |  |  |  |  |  |
|  | کیری بھائی میرے ہاتھ کھڑے ہیں یہ کام تمہاری ذہنییت اور پیار کوظاہر کرتا ہے | 0 |  |  |  |  |  |  |  |  |  |  |
|  | کیری بھائی ہم آپ کے ساتھ ہیں اس جیسی غلط ویڈیوز نہ پوسٹ کریں | 0 |  |  |  |  |  |  |  |  |  |  |
|  | کیری بھائی ہمارا نیتا آصف ہے | 0 |  |  |  |  |  |  |  |  |  |  |
|  | کیری بھائی ویڈیو بہت دیر کے بعد آرہی ہے | 0 |  |  |  |  |  |  |  |  |  |  |
|  | کیری پب جی کے اوپر ایک بناؤ | 0 |  |  |  |  |  |  |  |  |  |  |
|  | کیری پلیز اس کو دیکھو | 0 |  |  |  |  |  |  |  |  |  |  |
|  | کیری تم اتنی زیادہ رقم کہاں سے کما لیتے ہو | 0 |  |  |  |  |  |  |  |  |  |  |
|  | کیری تم بہت اچھے ہو اگلی ویڈیو کب اپ لوڈ کر رہے ہو | 0 |  |  |  |  |  |  |  |  |  |  |
|  | کیری تم کو ڈانس کرنے کی کوشش کرنی چاہیے | 1 |  |  |  |  |  |  |  |  |  |  |
|  | کیری تمہاری اس ویڈیو میں تمہارا پاجامہ پھٹا ہوا ہے | 0 |  |  |  |  |  |  |  |  |  |  |
|  | کیری تو سب سے اچھا ہے اور اس میں کوئی شک نہں | 0 |  |  |  |  |  |  |  |  |  |  |
|  | کیری تیرے پاس کم دھوئیں والی گاڑی ہے | 0 |  |  |  |  |  |  |  |  |  |  |
|  | کیری تیرے ہاتھ چلتے ہیں جب ممی بلاتی ہیں | 0 |  |  |  |  |  |  |  |  |  |  |
|  | کیری تیری ویڈیوز بہت کمال کی ہیں لیکن تیرے بات کرنے کا طریقہ انتا اچھا کیسے ہے پلیز جواب لازمی دیں | 0 |  |  |  |  |  |  |  |  |  |  |
|  | کیری دنچک پوجا | 0 |  |  |  |  |  |  |  |  |  |  |
|  | کیری دنچک پوجا کا نئی ویڈیو آیا ہے سیلفی مہربانی کر کے پوجا کے سیلفی گانے پر ویڈیو بناؤ | 0 |  |  |  |  |  |  |  |  |  |  |
|  | کیری سال بہت تم بہت کم ویڈیو بنائے گا میری پیشین گوئی ہے یہ | 0 |  |  |  |  |  |  |  |  |  |  |
|  | کیری سے بڑا چوتیا تو پیدا ہی نہں ہوا کوئی جو کتوں کی طرح سبسکرائبرز مانگ رہا ہے | 1 |  |  |  |  |  |  |  |  |  |  |
|  | کیری شریک پوپ کے اوپر بھی | 0 |  |  |  |  |  |  |  |  |  |  |
|  | کیری فاطمہ کنچ والا ویڈیو بناؤ پلیز | 0 |  |  |  |  |  |  |  |  |  |  |
|  | کیری کا سب سے اچھا شو | 0 |  |  |  |  |  |  |  |  |  |  |
|  | کیری کپل شرما کے شو پر کب آئے گا تو | 0 |  |  |  |  |  |  |  |  |  |  |
|  | کیری کوئی الفاظ نہیں آپ کے لیے پیار ہے بہت بہت گجرات سے | 0 |  |  |  |  |  |  |  |  |  |  |
|  | کیری کی کاپی ہے تو | 0 |  |  |  |  |  |  |  |  |  |  |
|  | کیری لویومیں پاکستان سے ہوں اور وقار ذکاء کو نہ دیکھا دیں | 0 |  |  |  |  |  |  |  |  |  |  |
|  | کیری مجھے تجھ سے پیار ہے بچے | 0 |  |  |  |  |  |  |  |  |  |  |
|  | کیری مناتی بہت اچھی ویڈیو اور پلیز بتا دو کہ تم اپنی ویڈیوز کو کس سافٹ وئیر سے ایڈٹ کرتی ہو | 0 |  |  |  |  |  |  |  |  |  |  |
|  | کیری مناتی بھائی میں سب سے تمہارا بڑا فائن ہوں پر ایک بات ہے کہ تو پہلے والی لوک میں ہی خوبصورت لگتا ہے | 0 |  |  |  |  |  |  |  |  |  |  |
|  | کیری مناتی بی بی سے بہتر ہے | 0 |  |  |  |  |  |  |  |  |  |  |
|  | کیری مناتی پلیز ناڈی خان کو بھی روسٹ کریں | 0 |  |  |  |  |  |  |  |  |  |  |
|  | کیری مناتی تمہاری آواز بہت سندر ہے | 0 |  |  |  |  |  |  |  |  |  |  |
|  | کیری مناتی کی اچھی ویڈیو ہے | 0 |  |  |  |  |  |  |  |  |  |  |
|  | کیری مناتی کی مناتی محفوظ کیسے ہو گیا پیچھے کا کیا ہوا | 0 |  |  |  |  |  |  |  |  |  |  |
|  | کیری مہربانی کرکے سالیان بھائیوں پر بھی ویڈیو بناؤ بہت مزہ آئی مہربانی کر کے ویڈیو بناؤ | 0 |  |  |  |  |  |  |  |  |  |  |
|  | کیری میری ویڈیو کی بھی بے عزتی کر دو | 0 |  |  |  |  |  |  |  |  |  |  |
|  | کیری یار اتنا عرصہ ہو گیا کوئی ویڈیو اپ لوڈ نہیں کیں | 0 |  |  |  |  |  |  |  |  |  |  |
|  | کیری یار بہت رامی | 0 |  |  |  |  |  |  |  |  |  |  |
|  | کیری یار تمہارا ڈانس بہت اچھا تھا اب ہر ویڈیو میں موہت سے ڈانس کروانا ضروری ہوتا ہے | 0 |  |  |  |  |  |  |  |  |  |  |
|  | کیری یہ تو پکا عمر کا پابند ہو گا بہن چود | 0 |  |  |  |  |  |  |  |  |  |  |
|  | کیسے ہو کیری بھائی ایک بار بات کرلو بھائی | 0 |  |  |  |  |  |  |  |  |  |  |
|  | کیوں اس کی خوشی میں اس کی گانڈ چاٹے گا | 1 |  |  |  |  |  |  |  |  |  |  |
|  | کیوں بھائی | 0 |  |  |  |  |  |  |  |  |  |  |
|  | کیوں بھائی پڑی ہوئی لکڑی گانڈ میں ڈال رہے ہو | 1 |  |  |  |  |  |  |  |  |  |  |
|  | کیوں بھائی رنڈی کیسی ہے وہ | 1 |  |  |  |  |  |  |  |  |  |  |
|  | کیوں سلمان اس کو کچھ آفر کروں گا | 0 |  |  |  |  |  |  |  |  |  |  |
|  | گاڈ آف وار تو کمپلیٹ کر بھائی | 0 |  |  |  |  |  |  |  |  |  |  |
|  | گار صیح گندی کی اس کی آپ سب نے مل کر بھوسڑی کا کنجر | 1 |  |  |  |  |  |  |  |  |  |  |
|  | گالی گالی میں ہلا ہے ادریس دلا ہے چار چوانی گوڈے پے فراگی دکی کے لوڑے پہ | 1 |  |  |  |  |  |  |  |  |  |  |
|  | گالیاں دینا انڈیا میں ایک عام سی بات ہے | 0 |  |  |  |  |  |  |  |  |  |  |
|  | گانڈ پھاڑ بہن چود ایک نمبر | 1 |  |  |  |  |  |  |  |  |  |  |
|  | گانڈ پھاڑ دی دکی بھائی | 1 |  |  |  |  |  |  |  |  |  |  |
|  | گانڈ پھاڑ دی میں آپ کے ساتھ ہوں بھائی انڈیا سے | 1 |  |  |  |  |  |  |  |  |  |  |
|  | گانڈ پھٹٰی کا گانڈو یہ ہرتیک روشن کے کھبے ٹٹے کی پیدائش ہے یہ | 1 |  |  |  |  |  |  |  |  |  |  |
|  | گانڈ پھٹ گئی شام کی اور نیا ویڈیو آگیا پلیز معاف کردو یار | 1 |  |  |  |  |  |  |  |  |  |  |
|  | گانڈ کا علاج ہے گانڈو کا نہں | 1 |  |  |  |  |  |  |  |  |  |  |
|  | گانڈ کے بال جلا دیے شام نے | 1 |  |  |  |  |  |  |  |  |  |  |
|  | گانڈ مار دی بھائی ایک نمبر | 1 |  |  |  |  |  |  |  |  |  |  |
|  | گانڈ مار دی تم نے کیری کی | 1 |  |  |  |  |  |  |  |  |  |  |
|  | گانڈ مارو اس کی اپنا لن اسکی گانڈ میں ڈال کتی کے بچے کی گانڈ پھاڑ دے | 1 |  |  |  |  |  |  |  |  |  |  |
|  | گانڈ مارو سالے کی چوتیا | 1 |  |  |  |  |  |  |  |  |  |  |
|  | گانڈ میں آگ لگ گئی ہو گی ان دونوں کی | 1 |  |  |  |  |  |  |  |  |  |  |
|  | گانڈ میں ڈالنا ہے منہ سے نکالنا ہے سب سے مزاحیہ جملہ | 1 |  |  |  |  |  |  |  |  |  |  |
|  | گانڈ ہی مار دی دونوں کی | 1 |  |  |  |  |  |  |  |  |  |  |
|  | گانڈ ہی مار لو بہن چود کی | 1 |  |  |  |  |  |  |  |  |  |  |
|  | گانڈو تو اپنے گریبان میں جھانک پہلے | 1 |  |  |  |  |  |  |  |  |  |  |
|  | گانڈو چبل لوگ ہیں یہ دونوں عجیب بہن چھولی چلائی ہے یوٹیوب پر | 1 |  |  |  |  |  |  |  |  |  |  |
|  | گانڈو شام ادریس | 1 |  |  |  |  |  |  |  |  |  |  |
|  | گانڈو کا بچہ شام | 1 |  |  |  |  |  |  |  |  |  |  |
|  | گانڈو کیوں مجھے نکال دیا | 1 |  |  |  |  |  |  |  |  |  |  |
|  | گانڈو نے اپنی فیس بک پیج پر کمنٹس بند کیے ہوئے ہیں | 1 |  |  |  |  |  |  |  |  |  |  |
|  | گانڈو ہے شام ادریس | 1 |  |  |  |  |  |  |  |  |  |  |
|  | گدی پر تھپڑ واہ بھائی واہ | 0 |  |  |  |  |  |  |  |  |  |  |
|  | گراگی تیری پھدی میں لن ماروں جب سے تمہں دیکھا ہے ہر روز مٹھ لگ جاتی ہے | 1 |  |  |  |  |  |  |  |  |  |  |
|  | گراگی کی میں گانڈ میں لن ماروں بہت بہت مزہ آئے گا | 1 |  |  |  |  |  |  |  |  |  |  |
|  | گروہ چوہدری تم گھر میں بھی ایسے ہی بات کرتے ہو | 0 |  |  |  |  |  |  |  |  |  |  |
|  | گروہ کیری مہاراج کی جے | 0 |  |  |  |  |  |  |  |  |  |  |
|  | گلشن بھیا کو بھی ویڈیو میں لے آیا کر | 0 |  |  |  |  |  |  |  |  |  |  |
|  | گلی گلی میں شور ہے سیٹھی دلا چور ہے | 1 |  |  |  |  |  |  |  |  |  |  |
|  | گندے لڑکے یہ کوئی پیدائیشی نہں تھا | 0 |  |  |  |  |  |  |  |  |  |  |
|  | گھر پر تمہارا عاشق کھڑا ہے | 0 |  |  |  |  |  |  |  |  |  |  |
|  | گورے گورے گال تیری چھاتی پر | 0 |  |  |  |  |  |  |  |  |  |  |
|  | گیا وہ گانڈو اب وہ نہں آئے گا | 1 |  |  |  |  |  |  |  |  |  |  |
|  | گیتا کا حققی ورژن بہت اچھا تھا | 0 |  |  |  |  |  |  |  |  |  |  |
|  | گیتا والے گانے میں میں حیران کو کون سا گانا چلایا ہے جو بالکل کردار کے ساتھ میچ ہو رہا تھا | 0 |  |  |  |  |  |  |  |  |  |  |
|  | گیم پلے دیکھانا شروع کریں کیوںکہ یہ بہت دلچسپ ہے | 0 |  |  |  |  |  |  |  |  |  |  |
|  | گیم پلے کون سی گیم کا ہے | 0 |  |  |  |  |  |  |  |  |  |  |
|  | گیم کون سی ہے یہ | 0 |  |  |  |  |  |  |  |  |  |  |
|  | گیمنگ والی ویڈیو کر کیری کبھی کبھی | 0 |  |  |  |  |  |  |  |  |  |  |
|  | لاکھوں اور لاکھوں کمنٹس | 0 |  |  |  |  |  |  |  |  |  |  |
|  | لاکھوں لائیکس بھی ہیں بھائی | 0 |  |  |  |  |  |  |  |  |  |  |
|  | لاکھوں لائیکس ٹک ٹاک کےچھکے | 0 |  |  |  |  |  |  |  |  |  |  |
|  | لاکھوں ہونے والے ہیں بھائی کے مبارک ہو ابھی سے آپ کو | 0 |  |  |  |  |  |  |  |  |  |  |
|  | لاکھوں ویورز اور لائیکس آپ کے لیے کیا چوتیاپا ہے | 1 |  |  |  |  |  |  |  |  |  |  |
|  | لائیکس کچھ اوقات سے زیادہ ہیں | 0 |  |  |  |  |  |  |  |  |  |  |
|  | لب اس بار آپ کی ویڈیوز نے تو شام ادریس کی گانڈ پھاڑ دی ہے | 1 |  |  |  |  |  |  |  |  |  |  |
|  | لبیب تو چوتیا ہے یہ لڑکی نہیں ہے لڑکا ہے | 1 |  |  |  |  |  |  |  |  |  |  |
|  | لڑکے سخت ہو گیا یہ تو اس مادر چود کو نیند نہیں آنی پوری زندگی اس سالے کو | 1 |  |  |  |  |  |  |  |  |  |  |
|  | لڑکے لیول کر دیا تو نے اسی گندی کی کہ کسی کی ہوتی نہیں دیکھی تم نے اس گانڈو کو کھول کے رکھ دیا | 1 |  |  |  |  |  |  |  |  |  |  |
|  | لڑکیوں اور لڑکوں میرے چینل کو پسند کریں میں جلد اس پر ویڈیوز ڈالوں گا | 0 |  |  |  |  |  |  |  |  |  |  |
|  | لعنت ایسی رنڈی پر | 1 |  |  |  |  |  |  |  |  |  |  |
|  | لعنت ہے شام تم پر چوتیا | 1 |  |  |  |  |  |  |  |  |  |  |
|  | لگتا ہے اب ان کی گانڈ اتنی پھٹ چکی ہے کہ یہ سالا اب پاکستان ہی نہں آرہا | 1 |  |  |  |  |  |  |  |  |  |  |
|  | لگتا ہے تیرے کو برنال کی ضرورت ہے پہلے تیری گانڈ میں لنڈ گھوساؤں گا اور پھر برنال لگاؤں گا کتے کے بچے | 1 |  |  |  |  |  |  |  |  |  |  |
|  | لن سے بڑا کچھ نہیں ہے | 1 |  |  |  |  |  |  |  |  |  |  |
|  | لو ابھی دکی بھائی باقی ہےفراگی شادی کر لے صیح چوتیاپا ہے | 1 |  |  |  |  |  |  |  |  |  |  |
|  | لو تمہارے لیے ایک ویڈیو ڈیڈیکیٹ کیا ہے | 0 |  |  |  |  |  |  |  |  |  |  |
|  | لو یو بہت اچھا لگا بہن چود زبردست | 1 |  |  |  |  |  |  |  |  |  |  |
|  | لو یو بھائی اس بھڑوے کے ساتھ یہی ہونا چاہیے تھا | 1 |  |  |  |  |  |  |  |  |  |  |
|  | لو یو بھائی بہت مزے کی ویڈیو بنائی آپ نےبہت اچھا چوتڑ کوٹا ہے اس کا | 1 |  |  |  |  |  |  |  |  |  |  |
|  | لو یو بھائی زبردست بہن چود کی صیح گانڈ ماری ہے اس کتے نے | 1 |  |  |  |  |  |  |  |  |  |  |
|  | لو یو بھائی میں تو ان بہن چودوں کے ایک سال سے مخالف ہوں | 1 |  |  |  |  |  |  |  |  |  |  |
|  | لو یو حجاب انڈیا سے اور یہ شام چوتیا بکواس ویڈیوز بناتا ہے | 1 |  |  |  |  |  |  |  |  |  |  |
|  | لو یو کیری بھائی ہمارے لاکھ سبسکرائبرز پورے ہونے والے ہیں | 0 |  |  |  |  |  |  |  |  |  |  |
|  | لو یو میری جان یہ بندہ ہے ہی بھوسڑی کا دلا اور بیوقوف آپ پریشان نہ ہوں | 1 |  |  |  |  |  |  |  |  |  |  |
|  | لوڑا کھا میرا | 1 |  |  |  |  |  |  |  |  |  |  |
|  | لوڑا ہا ہا ہا ہا | 1 |  |  |  |  |  |  |  |  |  |  |
|  | لوڑو لوڑو یدھو لوڑو | 1 |  |  |  |  |  |  |  |  |  |  |
|  | لوڑے تمہیں پاکستان کی فیفا رینکنگ پتہ ہے آگیا سالا فٹ بال کھیلنے | 1 |  |  |  |  |  |  |  |  |  |  |
|  | لوڑے تیری ویڈیو لوڑا دیتا نہیں | 1 |  |  |  |  |  |  |  |  |  |  |
|  | لوڑے لگ گئے سب | 1 |  |  |  |  |  |  |  |  |  |  |
|  | لوڑے لگ گئے شام ادریس کے بھائی بالکل ٹھیک بات اور بہت بڑا دلا ہے سالا | 1 |  |  |  |  |  |  |  |  |  |  |
|  | لوڑے لگا دئیے تم نے بھوسڑی کے کو لو یو | 1 |  |  |  |  |  |  |  |  |  |  |
|  | لوگ اس کی ویڈیو کو بھی ناپسند کر رہے ہیں سچی میں چوتیا ہے یہ | 1 |  |  |  |  |  |  |  |  |  |  |
|  | لوگوں نے اس ویڈیو کو ناپسند کیا ہے یہ وہی ہے جن کی گانڈ پر کوڑے مارے گیئے تھے | 1 |  |  |  |  |  |  |  |  |  |  |
|  | لویو حجاب دکی بھائی گانڈ مارو اس سالے کی | 1 |  |  |  |  |  |  |  |  |  |  |
|  | لے بھائی اس پر ویڈیو بنا اور کھول کر داد وصول کر | 0 |  |  |  |  |  |  |  |  |  |  |
|  | لیکن ابھی بھی تم سے پیار کرتا ہوں | 0 |  |  |  |  |  |  |  |  |  |  |
|  | لیکن اپنی موٹر سائیکل کتنے میں بیچی | 0 |  |  |  |  |  |  |  |  |  |  |
|  | لیکن اچھا کام ہے آپ کا میں آپ کی تعریف کرتا ہوں | 0 |  |  |  |  |  |  |  |  |  |  |
|  | لیکن اس طرف کوئی رجحان نہیں ہے | 0 |  |  |  |  |  |  |  |  |  |  |
|  | لیکن اس کا اصل نام فراڈی ہے | 0 |  |  |  |  |  |  |  |  |  |  |
|  | لیکن اس کا لنڈ بہت بڑا ہے آؤ نا کبھی حویلی پر | 0 |  |  |  |  |  |  |  |  |  |  |
|  | لیکن اسلام تشدد نہیں سکھاتا | 0 |  |  |  |  |  |  |  |  |  |  |
|  | لیکن ان کی بغیرت اور چت قسم کی ویڈیوز نے مجھے پاگل بنا دیا | 1 |  |  |  |  |  |  |  |  |  |  |
|  | لیکن آپ بابوسر تو نہیں گیئے | 0 |  |  |  |  |  |  |  |  |  |  |
|  | لیکن بھائی یہ آخر میں کیا تھا اس کی تو شکل ہی چھڈو ہے سالے کی | 1 |  |  |  |  |  |  |  |  |  |  |
|  | لیکن تم ایک چوتیے ہو | 1 |  |  |  |  |  |  |  |  |  |  |
|  | لیکن چوتیا ہے کون | 1 |  |  |  |  |  |  |  |  |  |  |
|  | لیکن حادثہ والا چکر نہیں بتایا کتے | 1 |  |  |  |  |  |  |  |  |  |  |
|  | لیکن دکان والے نے کہا کہ سالے منہ مع لوڑا پھینک کر ماروں گا نکل میری دکان سے | 0 |  |  |  |  |  |  |  |  |  |  |
|  | لیکن دوسروں سے بری باتیں کر رہا ہے | 0 |  |  |  |  |  |  |  |  |  |  |
|  | لیکن کیوں اتنی گندی زبان کوئی ڈر نہیں | 0 |  |  |  |  |  |  |  |  |  |  |
|  | لیکن مسلم عورتوں کو ہی ضروری نہیں کہ وہ نقاب کریں | 0 |  |  |  |  |  |  |  |  |  |  |
|  | لیکن میں اب بہت خوش ہوں | 0 |  |  |  |  |  |  |  |  |  |  |
|  | لیکن میں ایک بہت اچھی بہوں ہوں | 0 |  |  |  |  |  |  |  |  |  |  |
|  | لیکن میں پسند نہیں کرتا | 0 |  |  |  |  |  |  |  |  |  |  |
|  | لیکن میں کہوں گا کہ دونوں گانڈ مرواؤ بہن چودی کی بھی کوئی حد ھوتی ہے | 1 |  |  |  |  |  |  |  |  |  |  |
|  | لیکن میں نے بی بی کا میوزک سنا ہے لیکن اس کو پہلے ایڈ کرو | 0 |  |  |  |  |  |  |  |  |  |  |
|  | لیکن ہم پیار کرتے ہیں عرفان بھائی | 0 |  |  |  |  |  |  |  |  |  |  |
|  | لیکن یہ دیپک کا لڑکا زیادہ بے عزتی نہیں کر رہا ہے | 1 |  |  |  |  |  |  |  |  |  |  |
|  | لینے کا کیا پلان ہے | 0 |  |  |  |  |  |  |  |  |  |  |
|  | مادر چود تیری گانڈ میں بمبو ڈالے گا بہن چود | 1 |  |  |  |  |  |  |  |  |  |  |
|  | مادر چود سالا پاکستان | 1 |  |  |  |  |  |  |  |  |  |  |
|  | مادر چود شام لونڈا فراگی تیری گانڈ میں گھوڑے کا لوڑا | 1 |  |  |  |  |  |  |  |  |  |  |
|  | مادر چود کیسے ڈسلائیک مار رہے ہو دکی بھائی کو نبڑو ویڈیو ٹھیک سے دیکھو | 1 |  |  |  |  |  |  |  |  |  |  |
|  | مادر چود ہے شام سالا لو یو دکی بھائی | 1 |  |  |  |  |  |  |  |  |  |  |
|  | مادرچود ادریس | 1 |  |  |  |  |  |  |  |  |  |  |
|  | مادرچود خود ہے زیادہ نہں ہو سکتا تو شام کو بدنام ہی کر کے دیکھ لیں | 1 |  |  |  |  |  |  |  |  |  |  |
|  | مادرچود مار اس شام کی | 1 |  |  |  |  |  |  |  |  |  |  |
|  | مادرچود وہ آئرن مین نہں ہے وہ بیٹ میں ہے | 1 |  |  |  |  |  |  |  |  |  |  |
|  | مار جاتے ہیں بہن کے لوڑے | 1 |  |  |  |  |  |  |  |  |  |  |
|  | ماریہ باجی آپ کو کچھ نہیں پتہ آپ بس گانا سنا کرو دکی کے بارے میں کوئی غلط بات نہں | 1 |  |  |  |  |  |  |  |  |  |  |
|  | ماں چود اس بھوسڑی کے والے کی | 1 |  |  |  |  |  |  |  |  |  |  |
|  | ماں چود اندھے کی آنکھ کول نہ کہ دھندے کی | 1 |  |  |  |  |  |  |  |  |  |  |
|  | ماں چود دو اس کی | 1 |  |  |  |  |  |  |  |  |  |  |
|  | ماں چود دی اس کی | 1 |  |  |  |  |  |  |  |  |  |  |
|  | ماں چود دی بھائی اس بھوسڑی کے کی تم نے | 1 |  |  |  |  |  |  |  |  |  |  |
|  | ماں چود دی تو نے تو اس کی | 1 |  |  |  |  |  |  |  |  |  |  |
|  | ماں چود دی دکی بھائی لو یو انڈیا سے | 1 |  |  |  |  |  |  |  |  |  |  |
|  | ماں چود دیں گے اس کی اگر غلطی سے بھی برے کمنٹس کرنے کی کوشش کی تو | 1 |  |  |  |  |  |  |  |  |  |  |
|  | ماں چود کر رکھ دی آج تو ان سالوں کی | 1 |  |  |  |  |  |  |  |  |  |  |
|  | ماں چودو اس کی ہم آپ کے ساتھ ہیں | 1 |  |  |  |  |  |  |  |  |  |  |
|  | ماں کی چوت کو بند کرو کمینے تم ادریس کے چینل کو بند کرواؤ گے | 1 |  |  |  |  |  |  |  |  |  |  |
|  | ماں کی گانڈ لنگڑے کی | 1 |  |  |  |  |  |  |  |  |  |  |
|  | مبارک کو کیری آئی لو یو | 0 |  |  |  |  |  |  |  |  |  |  |
|  | مبارک ہو بھائی ایسی والی ہی چاہیے | 0 |  |  |  |  |  |  |  |  |  |  |
|  | مبارک ہو بھائی لاکھوں ویورز کے لیئے | 0 |  |  |  |  |  |  |  |  |  |  |
|  | مبارک ہو مجھے سبسکرائب کرو | 0 |  |  |  |  |  |  |  |  |  |  |
|  | مجھے ابھی تک سمجھ نہں آئی کہ کیری کو لاکھوں سبسکرائبرز کیسے مل گئے کون چوتیا لوگ ہیں جو اس کو سبسکرائب کرتے ہیں | 1 |  |  |  |  |  |  |  |  |  |  |
|  | مجھے اس کا موضوع بہت پسند ہے | 0 |  |  |  |  |  |  |  |  |  |  |
|  | مجھے ایسا ہی تو لگتا ہے | 0 |  |  |  |  |  |  |  |  |  |  |
|  | مجھے بتائیں کہ کس نے کیری کو لائیک کیا ہے | 0 |  |  |  |  |  |  |  |  |  |  |
|  | مجھے بمبئی میں جانا تھا پر میں بممبئی میں ہی رہ رہا تھا | 0 |  |  |  |  |  |  |  |  |  |  |
|  | مجھے بھی اپنا لوڑا گورا کرنا ہے مجھے بھی اپنی کریم دےدے اس کی شکل سے تو اچھا میرا لوڑا ہے | 1 |  |  |  |  |  |  |  |  |  |  |
|  | مجھے بھی ہمیشہ زندہ رہنے والی بوٹی دے دو شکریہ | 0 |  |  |  |  |  |  |  |  |  |  |
|  | مجھے پہلے سے ہی اس پر چوتیا پر غصہ آتا تھا آج دکی بھائی نے اس کو اور کھول کر رکھ دیا | 1 |  |  |  |  |  |  |  |  |  |  |
|  | مجھے تم پر فخر ہے بھائی | 0 |  |  |  |  |  |  |  |  |  |  |
|  | مجھے تو شروع سے ہی شام رنڈی نہں پسند | 1 |  |  |  |  |  |  |  |  |  |  |
|  | مجھے سٹار بوائے کا گانا بہت پسند آیا بہت اچھا کام تھا کیری | 0 |  |  |  |  |  |  |  |  |  |  |
|  | مجھے سمجھ نہیں آتا کہ کون بڑا چوتیا نا پسند کی بٹن کو کلک کرتا ہے | 1 |  |  |  |  |  |  |  |  |  |  |
|  | مجھے کالج جانا پسند نہیں ہے | 0 |  |  |  |  |  |  |  |  |  |  |
|  | مجھے کسی بھی وجہ سے سبسکرائبر چاہیئں | 0 |  |  |  |  |  |  |  |  |  |  |
|  | مجھے لاکھوں سبسکرائبر چاہیں مہربانی فرمائیں | 0 |  |  |  |  |  |  |  |  |  |  |
|  | مجھے لگا تو نے کہا ہے پھنس گیا تھا پھر دوبارہ دیکھا | 0 |  |  |  |  |  |  |  |  |  |  |
|  | مجھے نہں پتا پر چوتیا وہ نہں چوتیا وہ لوگ ہیں جو اس کی ویڈیوز دیکھتے ہیں | 1 |  |  |  |  |  |  |  |  |  |  |
|  | مجھے یہ ٹون اور میوزک بہت پسند ہے | 0 |  |  |  |  |  |  |  |  |  |  |
|  | مجھے یہ کہتے ہوئے شرم آرھی ہے کہ میں آسام سے ہوں | 0 |  |  |  |  |  |  |  |  |  |  |
|  | محترم حجاب بھائی صیح بجائی تم نے اس چوتیا کی میں تم کو اس کی داد دیتا ہوں | 1 |  |  |  |  |  |  |  |  |  |  |
|  | مذہب کے نام پر جب یہ چوتیاپا رہنا تب تک انڈیا پاکستان اور برصغیر کی حالت نہں بدل سکتی | 1 |  |  |  |  |  |  |  |  |  |  |
|  | مرے کمنٹ کو سب سے زیادہ لائیکس کرنے کا شکریہ | 0 |  |  |  |  |  |  |  |  |  |  |
|  | مری مدد کرنا پلیز میں ایک سافٹ وئیر چلا رہا ہوں لیکن اس کے لیے بٹ چاہیے اور میرے پاس بٹ نہں ہے کیا میں کیسے چل سکتا ہوں | 0 |  |  |  |  |  |  |  |  |  |  |
|  | مزہ آگیا بھائی اس میں جو گیم ہے اس کا نام کیا ہے | 0 |  |  |  |  |  |  |  |  |  |  |
|  | مزہ آگیا بھائی مگر یہ شکل اور عقل سے بھی چوتیا ہے | 1 |  |  |  |  |  |  |  |  |  |  |
|  | مزہ آگیا بھائی میری دعا ہے کی تم ہمیں اسی طرح محظوظ کرنے میں کامیاب رہو | 0 |  |  |  |  |  |  |  |  |  |  |
|  | مزہ آگیا تم ویڈیو بنانے میں مست ہو وہ سیکس کو ملانے ولا سین بہت اچھا تھا | 0 |  |  |  |  |  |  |  |  |  |  |
|  | مزہ آگیا کیری بھائی | 0 |  |  |  |  |  |  |  |  |  |  |
|  | مس وہ گیم پلے کی ویڈیو فیس کیم سے زیادہ پسند آیا | 0 |  |  |  |  |  |  |  |  |  |  |
|  | مست بھائی چھا گئے | 0 |  |  |  |  |  |  |  |  |  |  |
|  | مست ہے دکی بھائی شام کی گانڈ جلتےہوئے دیکھنے میں مزہ آیا بہت | 1 |  |  |  |  |  |  |  |  |  |  |
|  | مست ویڈیو ہے بھائی | 0 |  |  |  |  |  |  |  |  |  |  |
|  | مست ویڈیو ہے بھائی پہلے ولا بغیر شکل کے بنایا اس بار | 0 |  |  |  |  |  |  |  |  |  |  |
|  | مسٹر لوتھا ٹھیک ہے بھائی تم کرو | 0 |  |  |  |  |  |  |  |  |  |  |
|  | مسکان کالرہ پر کوئی ویڈیو بناؤیار | 0 |  |  |  |  |  |  |  |  |  |  |
|  | مسلمان کے شر سے مسلمان بچے رہیں اور غیر مسلم کی گانڈ مار دو | 1 |  |  |  |  |  |  |  |  |  |  |
|  | مشرہ جی تو توپ نکلے | 0 |  |  |  |  |  |  |  |  |  |  |
|  | مشرہ جی سب سے اچھا بہاری کریکٹر کر رہا ہے مجھے بہت پسند آیا | 0 |  |  |  |  |  |  |  |  |  |  |
|  | مشرہ جی کے لیے لائیک کرو یارو | 0 |  |  |  |  |  |  |  |  |  |  |
|  | مشرہ جی نے سب سے زیادہ کھیل کھیلا ہے | 0 |  |  |  |  |  |  |  |  |  |  |
|  | مشہور لوگوں کا ڈانس اور بیک گراؤنڈ میں میوزک | 0 |  |  |  |  |  |  |  |  |  |  |
|  | مطلب کچھ بھی فالتو میں چھیڑ سے چھیڑا لوگوں نے | 0 |  |  |  |  |  |  |  |  |  |  |
|  | معزز بھائی یہ چوتیا کبھی بھی کسی کو نہیں چھوڑے گا | 1 |  |  |  |  |  |  |  |  |  |  |
|  | معصومی ہا ہا ہا ہنس ہنس کے برا ہال ہو گیا بھائی کمال ہو گیا | 0 |  |  |  |  |  |  |  |  |  |  |
|  | ملائکہ پر ویڈیوز بنائیں | 0 |  |  |  |  |  |  |  |  |  |  |
|  | ممسئلوں کو حل کرنےوالا | 0 |  |  |  |  |  |  |  |  |  |  |
|  | ممی میرے ہیڈفون دیکھے ہیں کیا کیری مناتی | 0 |  |  |  |  |  |  |  |  |  |  |
|  | مناتی بھائی اترو میں ٹیکنیکل گروجی کو روسٹ کرنے لگا تھا کیا | 0 |  |  |  |  |  |  |  |  |  |  |
|  | مناتی بھائی گیم پر کب ویڈیو بنا رہا ہے تو | 0 |  |  |  |  |  |  |  |  |  |  |
|  | مناتی تم بہترین ہو سب سے اور وہ سموسہ مزے کا تھا | 0 |  |  |  |  |  |  |  |  |  |  |
|  | منٹ ہوا نہیں کہ کمنٹ آگئے | 0 |  |  |  |  |  |  |  |  |  |  |
|  | منٹوں میں لاکھوں دیکھنے والے واہ | 0 |  |  |  |  |  |  |  |  |  |  |
|  | منچلوں مہربانی کر کے آم چھی والے گانے پر بھی کچھ کہو | 0 |  |  |  |  |  |  |  |  |  |  |
|  | منچلے بالوں کی کٹنگ بہت بری ہے | 0 |  |  |  |  |  |  |  |  |  |  |
|  | منہ بند کروا دیا آپ نے اس سالے کا بہت بکواس کر رہا تھا آج کل وہ بہن چود | 1 |  |  |  |  |  |  |  |  |  |  |
|  | منوج سنگھ تیری ماں اور بہن کو رکھیل بنانے والے | 0 |  |  |  |  |  |  |  |  |  |  |
|  | مہربانی فرما کر ایک ویڈیو اس پوپی پر بھی بنائیں | 0 |  |  |  |  |  |  |  |  |  |  |
|  | مہربانی کر کے اپ میرے چینل کو سبسکرائب کریں پروان ٹیک | 0 |  |  |  |  |  |  |  |  |  |  |
|  | مہربانی کر کے اس بکواس پر بھی ایک ویڈیو بناؤ | 0 |  |  |  |  |  |  |  |  |  |  |
|  | مہربانی کر کے اکثر اپ لوڈ کیا کریں | 0 |  |  |  |  |  |  |  |  |  |  |
|  | مہربانی کر کے جھوٹی عورتوں پر ویڈیو بناؤ | 0 |  |  |  |  |  |  |  |  |  |  |
|  | مہربانی کر کے دنچک پوجا کا نیا گانا روسٹ کرو سیلفی لینے آجا | 0 |  |  |  |  |  |  |  |  |  |  |
|  | مہربانی کر کے فیس کیم بند کر کے گیم پلے پر دوبارہ آؤ | 0 |  |  |  |  |  |  |  |  |  |  |
|  | مہربانی کر کے مجھے سبسکرائب کریں اور میں بھی آپ کو سبسکرائب کروں گا | 0 |  |  |  |  |  |  |  |  |  |  |
|  | مہربانی کر کے ہماری مزاحیہ ویڈیو دیکھیں اور اگر اچھی لگے تو مہربانی کر کے رجسٹرہوں | 0 |  |  |  |  |  |  |  |  |  |  |
|  | مہربانی کرکے میرا چینل سبکرائب کریں میں مزاحیہ ویڈیوز کو اپ لوڈ کرتا ہوں | 0 |  |  |  |  |  |  |  |  |  |  |
|  | مہربانی کرکے میرا چینل کی ویڈیوز دیکھیں نہیں تو میں اس کو چھوڑ رہا ہوں | 0 |  |  |  |  |  |  |  |  |  |  |
|  | مہربانی کرو مجھے چاند پر نہں جانا ہے | 0 |  |  |  |  |  |  |  |  |  |  |
|  | مہربانی ہوگی مجھےآخری والا پورا گانا چاہیے | 0 |  |  |  |  |  |  |  |  |  |  |
|  | موہت مناتی بھی ہے | 0 |  |  |  |  |  |  |  |  |  |  |
|  | موہت نام لے رہا تھا بندہ تو کچھ تو منطق ہوگی کوئی دوست ہوگا موہت نام کا وغیرہ وغیرہ | 0 |  |  |  |  |  |  |  |  |  |  |
|  | میاں خلیفہ کی طرح دیکھا رہا ہے تولیکن جو بھی ہے میرا بہت پسندیدہ ہے تو | 0 |  |  |  |  |  |  |  |  |  |  |
|  | میر عامر بھائی یہ بہت سخت ہے | 0 |  |  |  |  |  |  |  |  |  |  |
|  | میرا اطلاعات کا وزیر کدہر ہے | 0 |  |  |  |  |  |  |  |  |  |  |
|  | میرا بہت پسندیدہ کیری | 0 |  |  |  |  |  |  |  |  |  |  |
|  | میرا تو بورڈ کا امتحان ہے اور تیاری تو بالکل بھی نہیں ہے | 0 |  |  |  |  |  |  |  |  |  |  |
|  | میرا خیال ہے کہ مجھے ویڈیوز دیکھنی چاہیے لیکن میں نے اب حادثاتی طور پر یہ ویڈیو دیکھی ہے | 0 |  |  |  |  |  |  |  |  |  |  |
|  | میرا خیال ہے کہ میں اپنی ساس کے ساتھ موم کی طرح سلوک کروں گی | 0 |  |  |  |  |  |  |  |  |  |  |
|  | میرا خیال ہے وہ جانتا ہے کہ وہ کتنے منٹ کی ویڈیو تھی | 0 |  |  |  |  |  |  |  |  |  |  |
|  | میرا دل چاہتا ہے شام کو ایک لوڑا ماروں اس کا خاندان پورا چود گیا | 1 |  |  |  |  |  |  |  |  |  |  |
|  | میرا دل کرتا ہے جب تک یہ یوٹیوب ہے میری تیری ساری ویڈٰیوز دیکھتا رہوں بہن چود آواز سن کر ہی ہنسی آجاتی ہے | 1 |  |  |  |  |  |  |  |  |  |  |
|  | میرہ خیال ہے یہ بندہ ٹھیک نہیں ہے | 0 |  |  |  |  |  |  |  |  |  |  |
|  | میرے بھائی تجھے تو خوش ہونا چاہئیے انتنی خوبصورت لڑکی تیرے ٹکڑوں پر زندہ ہے سالی تیرا نام لے کر بیرون ملک | 1 |  |  |  |  |  |  |  |  |  |  |
|  | میرے پسندیدہ حصہ کو شروع کریں | 0 |  |  |  |  |  |  |  |  |  |  |
|  | میرے چینل کا نام بھی اٹھا دوں یارنام ہے انڈین ٹھرکی چور | 0 |  |  |  |  |  |  |  |  |  |  |
|  | میرے ساتھ گھر میں مسلمان دوست رہتےہیں جو کہ غلط نہں ہے باقی دینا کے لیے وہ کمینے ہیں اگر میرا دوست کمینہ نہیں ہے تو باقی مسلمان کیوں کمینے ہو گئے | 1 |  |  |  |  |  |  |  |  |  |  |
|  | میرے شائقین لائیک بٹن کو دبائیں | 0 |  |  |  |  |  |  |  |  |  |  |
|  | میرے علاوہ کون کیری مناتی کی ویڈیوز کا انتظار کر رہا ہے | 0 |  |  |  |  |  |  |  |  |  |  |
|  | میرے لیول کا ویڈیو ہے | 0 |  |  |  |  |  |  |  |  |  |  |
|  | میری آرایس کی جوتی آپ کا انتظار کررہی ہے | 0 |  |  |  |  |  |  |  |  |  |  |
|  | میری خواہش ہے کہ میں اکیلا اس کو لاکھوں بار لائیکس کر سکوں | 0 |  |  |  |  |  |  |  |  |  |  |
|  | میری خواہش ہے کہ میں ایک سے زیادہ بار لائیک کر سکتا | 0 |  |  |  |  |  |  |  |  |  |  |
|  | میری سب سے آخری پسندیدہ انوپ جلوتا ہے | 0 |  |  |  |  |  |  |  |  |  |  |
|  | میری طرف سے ان کو بھی مبارک دے دینا کیری بھائی | 0 |  |  |  |  |  |  |  |  |  |  |
|  | میری ویڈیوز آپ سے بہتر ہیں میں اپنے آپ کو مارنے لگا ہوں | 0 |  |  |  |  |  |  |  |  |  |  |
|  | میں اب جب بھی کمپیوٹر پر گانے چلاتا ہوں تو مجھے یہ ویڈیو یاد آجاتی ہے | 0 |  |  |  |  |  |  |  |  |  |  |
|  | میں ابھی تک اسی کو دیکھ رہا ہوں | 0 |  |  |  |  |  |  |  |  |  |  |
|  | میں اس چوتیا کا انتظار کر رہا ہوں ویسے ویڈیو بہت اچھی تھی دکی بھائی | 1 |  |  |  |  |  |  |  |  |  |  |
|  | میں اس سے متفق ہوں کہ میں یہ لے لیتا ہوں یہ کہ کر کہ اس کی کیا ضرورت ہے | 0 |  |  |  |  |  |  |  |  |  |  |
|  | میں اس کی گندی والی بے عزتی چاہتی ہوں | 1 |  |  |  |  |  |  |  |  |  |  |
|  | میں اس کی ہر ویڈیو پر کمنٹ کرتا ہوں یہ ہے ہی چوتیا سالا بکواس کرتا ہے | 1 |  |  |  |  |  |  |  |  |  |  |
|  | میں اس ویڈیو کو کئی باردیکھ سکتا ہوں بہت دلچسپ اور مشرہ جی تو پکے بہاری لگ رہے ہیں | 0 |  |  |  |  |  |  |  |  |  |  |
|  | میں انڈیا سے ہوں اور سیٹھی چوتیا ہے سالا | 1 |  |  |  |  |  |  |  |  |  |  |
|  | میں ایسا سافٹ وئیر بنا رہا ہوں جو ان جیسے چوتیوں کے چینل بند کرے | 1 |  |  |  |  |  |  |  |  |  |  |
|  | میں آپ سے منتی پیار کرتا ہوں | 0 |  |  |  |  |  |  |  |  |  |  |
|  | میں آپ سےبھائی بہت متاثر ہوا ہوں اصل محنت تو آپ لوگ کرتے ہیں وہ تو صرف لوگوں کو چوتیا بنا رہا ہے | 1 |  |  |  |  |  |  |  |  |  |  |
|  | میں آپ کا بہت بڑا شائق ہوں مہربانی کر کے آپ میری دوستی قبول کریں | 0 |  |  |  |  |  |  |  |  |  |  |
|  | میں آپ کا بہت بڑا فین ہوں | 0 |  |  |  |  |  |  |  |  |  |  |
|  | میں آپ کا سب سے بڑا فائن ہوں اور اگر کوئی تمہارے بارے میں برا بولے گا تو میں اس کی گانڈ پھاڑ دوں گا | 1 |  |  |  |  |  |  |  |  |  |  |
|  | میں آپ کا فائن ہوں پلیز ویڈیو بناؤ میرا یار موڑ دو سچی دوستی | 0 |  |  |  |  |  |  |  |  |  |  |
|  | میں آپ کی ویڈیوز سے بہت پیار کرتا ہوں | 0 |  |  |  |  |  |  |  |  |  |  |
|  | میں بتا دوں کہ راج کمار ایک رنڈوہ اور بیٹی چود ہے | 1 |  |  |  |  |  |  |  |  |  |  |
|  | میں بس اس کے لیےانتظار کر رہا تھا | 0 |  |  |  |  |  |  |  |  |  |  |
|  | میں بمبئی سے ہوں میں شام کا بہت بڑا فین تھا پر اب وہ مجھ کو بھوسڑی کا چوتیا لگتا ہے | 1 |  |  |  |  |  |  |  |  |  |  |
|  | میں بنگلہ دیش سے ہو اردو بول نہں سکتا پر سمجھ آتی ہے یہ چوتیا ادریس کیسا نام ہے | 1 |  |  |  |  |  |  |  |  |  |  |
|  | میں بہت بے صبری سے شام کی میسج کا انتظار کر رہا ہوں میرا مطلب ہے اس چوتیا کا | 1 |  |  |  |  |  |  |  |  |  |  |
|  | میں بھی اس کو پسند کرتا ہوں | 0 |  |  |  |  |  |  |  |  |  |  |
|  | میں بھی ان دونوں کی ویڈیوز پسند کرتا ہوں | 0 |  |  |  |  |  |  |  |  |  |  |
|  | میں بیڈ سے نیچے گر گیا میرے پیچھے بندہ ڈال کر چلا گیا | 0 |  |  |  |  |  |  |  |  |  |  |
|  | میں پہلے اس کی ویڈیوز دیکھتا تھا پر ایک تو اتنا چوتیاپا کرکے دوسرا اتنی ہٹ دھرمی یہ بہت بڑا کمینہ ہے | 1 |  |  |  |  |  |  |  |  |  |  |
|  | میں پیار کرتا ہوں جس انداز سے وہ تم سب کی کھول کے مارتا ہے | 0 |  |  |  |  |  |  |  |  |  |  |
|  | میں تم سے بہت پیار کرتا ہوں۔ اچھا کام تھا | 0 |  |  |  |  |  |  |  |  |  |  |
|  | میں تم سے متفق ہو اوئے دلے | 1 |  |  |  |  |  |  |  |  |  |  |
|  | میں تم کو اس سے بہتر آدمی کامشورہ دیتا ہوں اس سے بھی خطرناک قسم کے لوگ ادھر رہتے ہیں | 0 |  |  |  |  |  |  |  |  |  |  |
|  | میں تمہارے چینل میں ہوں بہت اچھی ویڈیو ہے | 0 |  |  |  |  |  |  |  |  |  |  |
|  | میں تو پبجی نہیں کھیلتا لیکن لڑکیاں بھی کھیلتی ہیں کیا یہ گیم | 0 |  |  |  |  |  |  |  |  |  |  |
|  | میں تو تیرا بہت بڑا فائن ہو گیا عمل ردعمل مزاحیہ سب کچھ کمال کرتا ہے تو | 0 |  |  |  |  |  |  |  |  |  |  |
|  | میں چوتیا اس آدمی کو کہ رہا تھا آپ کو نہں معاف کرنا بھائی | 1 |  |  |  |  |  |  |  |  |  |  |
|  | میں حقیقت میں یہ چاہتا ہوں کہ تم یہ ویڈیو بناؤ | 0 |  |  |  |  |  |  |  |  |  |  |
|  | میں حیران ہوں کہ وہ کیوں کمنٹ نہیں کرتے نیچے او دلے او سورا | 1 |  |  |  |  |  |  |  |  |  |  |
|  | میں حیران ہوں کیری بھائی تم بہت عظیم ہو | 0 |  |  |  |  |  |  |  |  |  |  |
|  | میں دسمبر میں دوبارہ دیکھ رہا ہوں | 0 |  |  |  |  |  |  |  |  |  |  |
|  | میں دوبارہ لسی نہیں پی سکتا | 0 |  |  |  |  |  |  |  |  |  |  |
|  | میں صرف ابلیس کے بارے میں یہ کہتا ہوں کہ میں اس کو لنڈ مارتا ہوں | 1 |  |  |  |  |  |  |  |  |  |  |
|  | میں عمران خان کا فائن نہں ہو پر اس چوتیے نے اپنی اوقات دیکھا دی | 1 |  |  |  |  |  |  |  |  |  |  |
|  | میں کہتا ہوں کہ آپ نے کمال کر دیا اس کنجری کے بچے کو سبق سیکھا کر ایسے گانڈو لوگوں کو ایسا ہی جواب ملنا چاہیے | 1 |  |  |  |  |  |  |  |  |  |  |
|  | میں کیری مناتی کو بہت پسند کرتا ہوں اس کی ویڈیوز بہت زیادہ حیران کن ہیں | 0 |  |  |  |  |  |  |  |  |  |  |
|  | میں مسلمانوں کو سپورٹ کرتا ہوں | 0 |  |  |  |  |  |  |  |  |  |  |
|  | میں نہیں آیا بھائی اور وقت لگے گا ابھی لیکن کچھ اچھا بنایا کر | 0 |  |  |  |  |  |  |  |  |  |  |
|  | میں نے اس کا چینل ایک سال پہلے ہی ان سبسکرائب کر دیا چوتیا شام ہے یا گراگی | 1 |  |  |  |  |  |  |  |  |  |  |
|  | میں نے اس کو تلاش کیا اور ویڈیو کلک کیا | 0 |  |  |  |  |  |  |  |  |  |  |
|  | میں نے اس کے پیج پر اس کی گانڈ مار ہی دی ہے | 1 |  |  |  |  |  |  |  |  |  |  |
|  | میں نے اس کے کمنٹ باکس میں کمنٹ لکھا تھا اس چوتیا نے تھوڑی دیر بعد ڈیلیٹ کردیا | 1 |  |  |  |  |  |  |  |  |  |  |
|  | میں نے الکوحل پینا ختم کر دی ہے | 0 |  |  |  |  |  |  |  |  |  |  |
|  | میں نے ان کا بکواس چینل سبسکرائب کیا تھا لیکن بعد میں مجھے پتہ چل گیا کیا چوتیاپا سستا ترین شہرت پانے کے لیے ہے | 1 |  |  |  |  |  |  |  |  |  |  |
|  | میں نے ایک ماہ کے لیے رجسٹریشن کروائی ہے | 0 |  |  |  |  |  |  |  |  |  |  |
|  | میں نے بھی اس کی ویڈیو دیکھی ہے کیا تم ایسا کر سکتے ہوجو میں نے آپ کو ٹویٹر پر ویڈیو بھیجی ہے | 0 |  |  |  |  |  |  |  |  |  |  |
|  | میں نے تو اس بھوسڑی کے کی ویڈیوز دیکھنا ہی بند کر دی ہیں | 1 |  |  |  |  |  |  |  |  |  |  |
|  | میں نے تو اس چوتیا کو سبسکرائب بھی نہں کیا ہوا چھا کیا ہوا ہے آپ نے کام چک کر رکھو تھوڑا اور زیادہ | 1 |  |  |  |  |  |  |  |  |  |  |
|  | میں نے تو اس گانڈو کو بلاک کر دیا ہے | 1 |  |  |  |  |  |  |  |  |  |  |
|  | میں نے شروع سے ہی اس چھپکلی کی گانڈ والے کی کوئی ویڈیو ہی دیکھی میں یوٹیوبر ہوتا تو اس کی گانڈ پھاڑ دیتا | 1 |  |  |  |  |  |  |  |  |  |  |
|  | میں نے یوٹیوب کی گانڈ مار دے نہیں ہے اگریہ ویڈیو اس گانڈو کی وجہ سےیہ ویڈیو ہٹی ہے | 1 |  |  |  |  |  |  |  |  |  |  |
|  | میں نیا ہوں اور آپ کا کام بہت اچھا ہے | 0 |  |  |  |  |  |  |  |  |  |  |
|  | میں ہر آدمی کو جانتا ہوں لہذا کیری کو ان پر ویڈیو بنانی چاہیے | 0 |  |  |  |  |  |  |  |  |  |  |
|  | میں ہمیشہ ایسی ویڈیو کا انتظار کرتا ہوں جو اس نسلی چوتیا سالے پر بنائی گئی ہو | 1 |  |  |  |  |  |  |  |  |  |  |
|  | میں ہندو ہوں لیکن مسلمانوں اور سکھوں کے بارے بہت کچھ جانتا ہوں اگر یہ ویڈیو انہوں نے سکھوں کے بارے میں بنائی ہوتی تو انہوں نے کب کی اس کی گانڈ مار لی ہوتی | 1 |  |  |  |  |  |  |  |  |  |  |
|  | میں ہنسنا بند نہیں کرسکتا آج پہلی بار دیکھا کہ سموسے کو بھی حفاظت کے لیے استعمال کیا جا سکتا ہے | 0 |  |  |  |  |  |  |  |  |  |  |
|  | میں ہنسنا روک نہیں سکتا | 0 |  |  |  |  |  |  |  |  |  |  |
|  | میں وہ ویڈیو روک کر اس کے چینل پر اس کو گالی دینے گیا پر سب کچھ گانڈو نے بند کردیا | 1 |  |  |  |  |  |  |  |  |  |  |
|  | میں یہ دیکھ کر بتا سکتا ہوں کہ پاکستان انڈیا سے بہت اچھا ہے | 0 |  |  |  |  |  |  |  |  |  |  |
|  | میں یہ کہتا ہوں کی ایک رنڈی اور ایک عورت میں فرق ہوتا ہے وہ ایک رنڈی ہے ماں کی لوڑی جو بھی کرلو رنڈٰی رنڈی ہی رہتی ہے | 1 |  |  |  |  |  |  |  |  |  |  |
|  | میں یہ کہنا چاہتا ہوں کی شام ہے ہی دلا چوتیا اس کو جتنی گالیاں دو اتنی کم ہیں | 1 |  |  |  |  |  |  |  |  |  |  |
|  | میں یوٹیوب پر کبوتروں کے کھیل کی مشہوری کرتا ہوں | 0 |  |  |  |  |  |  |  |  |  |  |
|  | میں یوٹیوب پر نیا ہوں اور مجھے آپ کی مدد چاہیے پلیز میری مدد کریں | 0 |  |  |  |  |  |  |  |  |  |  |
|  | میں یوٹیوب والوں کو گزارش کرتا ہوں کہ وہ دیپک کلال کو بین کرے | 0 |  |  |  |  |  |  |  |  |  |  |
|  | میوزک انڈسٹری کا تو پتہ نہں لیکن یوٹیوب انڈسٹری ٹاپ پر ہے اور وہ بھی کیری کی وجہ سے | 0 |  |  |  |  |  |  |  |  |  |  |
|  | میوزک کی دنیا کیوں کہ پاکستان میں شام ادریس چوتیا بھی ہوتا ہے | 1 |  |  |  |  |  |  |  |  |  |  |
|  | میوزک ورلڈ تم چوتیا ہو | 1 |  |  |  |  |  |  |  |  |  |  |
|  | ناپسند کرو شام ادریس کے پیج کو چول بندہ ہے قسم سے بھوسڑی کا | 1 |  |  |  |  |  |  |  |  |  |  |
|  | ناراض بھائی اچھی ویڈیو ہے تمہاری بھی | 0 |  |  |  |  |  |  |  |  |  |  |
|  | ناصر جان اور نعمان سے جان چھوٹی اور اب یہ کتے کے بچے آگئے | 1 |  |  |  |  |  |  |  |  |  |  |
|  | نام کا پتہ کروا شام فراگی کا بھڑواہ ہے | 1 |  |  |  |  |  |  |  |  |  |  |
|  | نکھیل ویڈیو کا لنک بھیجنا آخری والی گانے کا | 0 |  |  |  |  |  |  |  |  |  |  |
|  | نمبر موٹا بھائی | 0 |  |  |  |  |  |  |  |  |  |  |
|  | نہ تو وکیل ہے نہ جج ہے نہ تو اپنی بہن کا یار ہے تیری ماں کو لنڈ | 1 |  |  |  |  |  |  |  |  |  |  |
|  | نہ سونے کا سریا اس کی گانڈمیں ڈنڈا دوں | 1 |  |  |  |  |  |  |  |  |  |  |
|  | نہں تھا پتہ مجھے اس بات کا ساقی کہ بلاگ اس لیول لے بہن چودی پر بھی کئے جاتے ہیں | 1 |  |  |  |  |  |  |  |  |  |  |
|  | نہیں بھائی تم سب سے جلتے ہو بس وہ ایک خالی یوٹیوبر ہے چھوڑو اس کمینے کو | 1 |  |  |  |  |  |  |  |  |  |  |
|  | نہیں بیٹا تیری ماں کو لنڈ دینا ہے اس کا | 1 |  |  |  |  |  |  |  |  |  |  |
|  | نہیں نہیں نہیں آخر ابھی بھی سمجھ میں آرہا ہے کوئلہ رگڑو اس پر | 0 |  |  |  |  |  |  |  |  |  |  |
|  | نوکیا کے کیمرہ کا سٹائل جاری رکھو یار اس کو | 0 |  |  |  |  |  |  |  |  |  |  |
|  | نیپالی بھائی سے آپ کے لیے بہت بہت پیار ہے | 0 |  |  |  |  |  |  |  |  |  |  |
|  | نیکھیل تیری بہن کی چت بھانگی | 1 |  |  |  |  |  |  |  |  |  |  |
|  | نیل شاہ نقاب کرنے والی بھی اپنی چوتیاپا کی حرکت بند کرے | 1 |  |  |  |  |  |  |  |  |  |  |
|  | نیو مونسٹر تو اپنی گانڈ مروا | 1 |  |  |  |  |  |  |  |  |  |  |
|  | نئے آنے والوں کی لیے جگنو آٹوز کی طرف سے فری سیر کرنے کا پروگرام ہے | 0 |  |  |  |  |  |  |  |  |  |  |
|  | ہا ہا اور ویڈیوز چاہیے | 0 |  |  |  |  |  |  |  |  |  |  |
|  | ہا ہا آجا مار مار | 0 |  |  |  |  |  |  |  |  |  |  |
|  | ہا ہا بھائی ٹھیک بولا | 0 |  |  |  |  |  |  |  |  |  |  |
|  | ہا ہا بھائی سپر ہے اور تم بھی | 0 |  |  |  |  |  |  |  |  |  |  |
|  | ہا ہا ٹٹے چاہیں بہت اچھا فک کرو | 1 |  |  |  |  |  |  |  |  |  |  |
|  | ہا ہا کیری کی اصلیت | 0 |  |  |  |  |  |  |  |  |  |  |
|  | ہا ہا محفل لوٹ لی کمال کردیا دیکھنا پھر کہیں سالا یہ چینل ہی بین نہ کروا دے | 1 |  |  |  |  |  |  |  |  |  |  |
|  | ہا ہا مزہ آگیا کیری بھائی | 0 |  |  |  |  |  |  |  |  |  |  |
|  | ہا ہا ہا بہت اچھا بھائی آخر کار تم نہ کر دیا ابلیس جھوٹے چینل والے بہن چود | 1 |  |  |  |  |  |  |  |  |  |  |
|  | ہا ہا ہا کم دھوئیں والی بیڑی ہے کیا بہت اعلی بھائی | 0 |  |  |  |  |  |  |  |  |  |  |
|  | ہا ہا ہا ارے واہ آپ تو کتنے بڑے چوتیا ہو | 1 |  |  |  |  |  |  |  |  |  |  |
|  | ہا ہا ہا اعلی یار شام ادریس کی گانڈ مار دے ویڈیو بنا بنا کر | 1 |  |  |  |  |  |  |  |  |  |  |
|  | ہا ہا ہا بہت اچھا شاباش | 0 |  |  |  |  |  |  |  |  |  |  |
|  | ہا ہا ہا بھائی آپ بہت مزاقیہ ہو | 0 |  |  |  |  |  |  |  |  |  |  |
|  | ہا ہا ہا بھائی تو اس کی گانڈ مار لے تھوک لگا کر ہا ہا ہا ہا | 1 |  |  |  |  |  |  |  |  |  |  |
|  | ہا ہا ہا کھجلانے سے جن آجاتا ہے میں بہت عرصہ سے کوشش کر رہا ہوں ہو نہں رہا | 0 |  |  |  |  |  |  |  |  |  |  |
|  | ہا ہا ہا کئی بار یہ ویڈیو دیکھی | 0 |  |  |  |  |  |  |  |  |  |  |
|  | ہا ہا ہا نہیں روک سکتا | 0 |  |  |  |  |  |  |  |  |  |  |
|  | ہا ہا یار دکی کی بجا دی تو نے بھوسڑی کا گانڈو | 1 |  |  |  |  |  |  |  |  |  |  |
|  | ہارش سنگھ ایک جیسا نام | 0 |  |  |  |  |  |  |  |  |  |  |
|  | ہازق اقبال ذاتی جنگ یہ لوگ خود بنا رہے ہیں اور کیوں روسٹ بنا رہا تھا دکی | 0 |  |  |  |  |  |  |  |  |  |  |
|  | ہاں اگر پھنسی ہوئی ہے تو مادر چود میرا کیا اکھاڑ لے گا جا اپنی ماں کی گانڈ میں گھس | 1 |  |  |  |  |  |  |  |  |  |  |
|  | ہاں تجھے گھنٹے کی سمجھ آئے گا تیرے دماغ میں سنی لیون جو پھنسی ہوئی ہے چل ہٹ بہن چود | 1 |  |  |  |  |  |  |  |  |  |  |
|  | ہاں تم ٹھیک ہو لیکن تم ہر کسی کا مشاہدہ نہں کر سکتا کیوںکہ ایسے گانا گانے والے لوگ صیح نہیں ہو سکتے | 0 |  |  |  |  |  |  |  |  |  |  |
|  | ہاں کیری بہت فین ہے | 0 |  |  |  |  |  |  |  |  |  |  |
|  | ہاں میں دیس ہوں | 0 |  |  |  |  |  |  |  |  |  |  |
|  | ہاں ہم چوتیے ہیں تو کیا بغل پھاڑ کر آیا تھا | 1 |  |  |  |  |  |  |  |  |  |  |
|  | ہاہا جانی تونے تو واٹ لگا دی اس دلے کی امید ہے آئندہ ویڈیوز کام کی ہی اپ لوڈ کیا کرے گا | 1 |  |  |  |  |  |  |  |  |  |  |
|  | ہاہا مزہ آگیا | 0 |  |  |  |  |  |  |  |  |  |  |
|  | ہاہا ہاآخیر کرگیا بہن چود آخر میں شادی کرنے کی جگہ | 1 |  |  |  |  |  |  |  |  |  |  |
|  | ہاہا یہ دونوں ہیں ہی مادر چود ان کی حادثہ والی ویڈیو دیکھ کر میں نے ان مادر چودوں کا چینل چھوڑ دیا تھا | 1 |  |  |  |  |  |  |  |  |  |  |
|  | ہاہاکیا بات ہے حیران کن | 0 |  |  |  |  |  |  |  |  |  |  |
|  | ہاہاہ بہت اچھا گیا دکی گانڈ مار دی سالے کی | 1 |  |  |  |  |  |  |  |  |  |  |
|  | ہاہاہا اچھا کیا کیری بھائی تم نے وہ پیشہ ور لوگ ہیں انتہا ئی چوتیا قسم کے | 1 |  |  |  |  |  |  |  |  |  |  |
|  | ہاہاہا الگ ہی بہن چودی ہے بہت بہترین ہے مائی ڈیئر | 1 |  |  |  |  |  |  |  |  |  |  |
|  | ہاہاہا بہن چود میٹھا ہے یہ نعمان خان | 1 |  |  |  |  |  |  |  |  |  |  |
|  | ہاہاہا پسند کرو اگر آپ کو پتا چل گیا ہے تو | 0 |  |  |  |  |  |  |  |  |  |  |
|  | ہاہاہا کیا بہن چود آدمی ہو تم | 1 |  |  |  |  |  |  |  |  |  |  |
|  | ہاہاہا کیری بھائی مباکر ہو پاکستان سے | 0 |  |  |  |  |  |  |  |  |  |  |
|  | ہاہاہا لو یو بھائی صیح کتوں والی کی آپ نے ان بھوسڑی کے کی | 1 |  |  |  |  |  |  |  |  |  |  |
|  | ہاہاہا لونڈے کی سخگی تو دیکھو دکی آج نہیں پگھلے گا کیا | 1 |  |  |  |  |  |  |  |  |  |  |
|  | ہاہاہا مار مار اس کی گانڈ مار | 1 |  |  |  |  |  |  |  |  |  |  |
|  | ہاہاہاہ ماں چود دی شام کی | 1 |  |  |  |  |  |  |  |  |  |  |
|  | ہاہاہاہا کیا بات ہے | 0 |  |  |  |  |  |  |  |  |  |  |
|  | ہاہاہاہاہا میں آج ان دونوں کی وجہ سے ہنس سکتا ہوں پر کچھ بول نہں سکتا چوتیا بنا رہے ہیں یہ سب کو | 1 |  |  |  |  |  |  |  |  |  |  |
|  | ہاہاہاہاہاہاہ بھائی آپ نے تو اس شامو کی گانڈ پھاڑ دی | 1 |  |  |  |  |  |  |  |  |  |  |
|  | ہائے آپ سب کو پریشان کیا پہلے آپ کو غلط لنک دے دیا تھا یہ اصل لنک ہے ویڈیو اور چینل کا آپ سبسکرائب بھی کر سکتے ہو شکرہ بہت بہت | 0 |  |  |  |  |  |  |  |  |  |  |
|  | ہائے پیارے کیسے ہو تم مجھے آپ کی مدد کی ضرورت ہے اپنا یوٹیوب کا چینل بہتر کرنے کے لیے | 0 |  |  |  |  |  |  |  |  |  |  |
|  | ہائے دوستو ویڈیوز دیکھو اور اپنے دوستوں کو ڈراؤ | 0 |  |  |  |  |  |  |  |  |  |  |
|  | ہائے سالگرہ مبارک ہو خوبصورت پائی | 0 |  |  |  |  |  |  |  |  |  |  |
|  | ہائے کیری جو بھی ردعمل ہے اس پر آپ پریشان نہ ہوں یہ بہت بہترین ویوڈیو تھی اور اس میں کو غلط بات نہیں ہے | 0 |  |  |  |  |  |  |  |  |  |  |
|  | ہائے کیری مجھے آپ کی ساری ویڈیوز بہت پسند ہیں مہربانی فرما کر ان کو بند مت کرنا | 0 |  |  |  |  |  |  |  |  |  |  |
|  | ہائے کیری میرا بھی میتھ کے ساتھ چھتیس کا اکڑا ہے | 0 |  |  |  |  |  |  |  |  |  |  |
|  | ہائے کیری میرا خیال ہے کہ میں آپ کے لیے سکرپٹ لکھ سکتا ہوں پر میں آپ کا بہت بڑا فائن ہوں | 0 |  |  |  |  |  |  |  |  |  |  |
|  | ہائے کیری میرا خیال ہے کہ والدین اور اساتذہو کو بھی یہ ویڈیوز دیکھنی چاہیں | 0 |  |  |  |  |  |  |  |  |  |  |
|  | ہائے معاف کرنا تمہارا منہ | 0 |  |  |  |  |  |  |  |  |  |  |
|  | ہائے میری ویڈیو دیکھو | 0 |  |  |  |  |  |  |  |  |  |  |
|  | ہپس ہپس ہرے ہپس ہپس ہرے | 0 |  |  |  |  |  |  |  |  |  |  |
|  | ہر کسی کی زندگی میں کچھ لوگ ایسے آتے ہیں جنہں دیکھ کر آپ پہلی بار ہی کہ دیتے ہیں کہ یہ اکھنڈ چوتیا ہے | 1 |  |  |  |  |  |  |  |  |  |  |
|  | ہرپل سنگ کاش آج تیرے باپ نے تجھے پیدا نہ کیا ہوتا کاش ایسا ہوتا | 0 |  |  |  |  |  |  |  |  |  |  |
|  | ہلاؤ جلاؤ یا دودھ پلاؤ | 0 |  |  |  |  |  |  |  |  |  |  |
|  | ہم تمہارے ساتھ ہیں بھوسڑی کا ہے جھوٹا ہے | 1 |  |  |  |  |  |  |  |  |  |  |
|  | ہم دکی بھائی کی سپورٹ کرتے ہیں چار چوانی گھوڑے پر سندھو میرے لوڑے پر | 1 |  |  |  |  |  |  |  |  |  |  |
|  | ہم سڑکوں پر پیشاب کرتے ہیں اور تم لوگ کھانے آجاتو ہو | 1 |  |  |  |  |  |  |  |  |  |  |
|  | ہم کیری کو دیکھنا پسند کرتے ہیں اگلے سیزن میں پلیز اس کو لائیک کریں | 0 |  |  |  |  |  |  |  |  |  |  |
|  | ہماری مزاحیہ ویڈیوز دیکھو آپ کو فوری پسند آجائیں گی اور سبسکرائب کرنا مت بھولنا | 0 |  |  |  |  |  |  |  |  |  |  |
|  | ہماری ویڈیو دیکھیں اور لنک پر کلک کریں اور اچھا لگے تو مہربانی کر کے سبسکرائب کریں سب کی مستی آپ کا سبسکرائب ہمارے لئیے انمول ہے | 0 |  |  |  |  |  |  |  |  |  |  |
|  | ہمیشہ کے لیے چوتیا | 1 |  |  |  |  |  |  |  |  |  |  |
|  | ہمیشہ کے لیئے اجے تم بہت بہتر اور بہت اچھے ہو | 0 |  |  |  |  |  |  |  |  |  |  |
|  | ہمیشہ کی طرح بہترین کیری | 0 |  |  |  |  |  |  |  |  |  |  |
|  | ہمیں اس کو بلاک کر دینا چاہیےپاکستان میں اس جھوٹے موٹے گانڈو کو | 1 |  |  |  |  |  |  |  |  |  |  |
|  | ہمیں پرامن احتجاج کرنا چاہیے | 0 |  |  |  |  |  |  |  |  |  |  |
|  | ہمیں کاپی کیے ہوئیں چیزوں پر کمنٹ نہیں کرنے چاہیں وہ تو بیوقوف ہے کچھ بھی سیدھا سیدہا کہ سکتا ہے | 0 |  |  |  |  |  |  |  |  |  |  |
|  | ہمیں کیا چاہئیں بس کچھ دن کے بعد امتحان ہے | 0 |  |  |  |  |  |  |  |  |  |  |
|  | ہمیں یہ تسلیم کرنا ہوگا کہ عظمیٰ فلاح حقیقت میں بہت پیاری ہے | 0 |  |  |  |  |  |  |  |  |  |  |
|  | ہوس کے پجاری میں نے یہ تو کہا ہی نہیں کہ یہ گالی ہےمطلب اس طرح کا کچھ بھی نہیں اس میں | 0 |  |  |  |  |  |  |  |  |  |  |
|  | ہیلو بھائی چائے پلاؤ نا | 0 |  |  |  |  |  |  |  |  |  |  |
|  | ہیلو بھائی سیدھی بات ہے بے غیرت اور چوتیا لکھ دو اس کی ویڈیو کے کمنٹس میں | 1 |  |  |  |  |  |  |  |  |  |  |
|  | ھمیں بھی نہیں پتا | 0 |  |  |  |  |  |  |  |  |  |  |
|  | واقعی میں شام چوتیا ہے اور فراگی باہر کے ملکوں میں ہماری مسلم عورتوں کی نمائندگی کرتی ہے | 1 |  |  |  |  |  |  |  |  |  |  |
|  | واہ اچھی ہے ویڈیو ایسے ہی لگے رہو | 0 |  |  |  |  |  |  |  |  |  |  |
|  | واہ بھائی کمال کر دیا اس بہن چود کی اور بونڈ مارو کتے کا بچہ گشتا ہے یہ | 1 |  |  |  |  |  |  |  |  |  |  |
|  | واہ بھائی واہ مزہ آگیا آپ کو بھی بھائی نیا سال مبارک ہو | 0 |  |  |  |  |  |  |  |  |  |  |
|  | واہ دکی بھائی چوت بند کردی ہے دونوں کی تم نے | 1 |  |  |  |  |  |  |  |  |  |  |
|  | واہ دیکھتا ہے کیری بھائی آگیا | 0 |  |  |  |  |  |  |  |  |  |  |
|  | واہ کیا بات ہے بھائی ایک اور بار مار دی اس لوڑے کی | 1 |  |  |  |  |  |  |  |  |  |  |
|  | واہ کیا بکواس ہے اپنے منہ میاں مٹھو بننا | 1 |  |  |  |  |  |  |  |  |  |  |
|  | واہ کیا ویڈیو ہے عمدہ عمدہ | 0 |  |  |  |  |  |  |  |  |  |  |
|  | واؤ بھائی کیا بات ہے | 0 |  |  |  |  |  |  |  |  |  |  |
|  | وشال وجال صیح ہے | 0 |  |  |  |  |  |  |  |  |  |  |
|  | وکاش تاؤ کو دیکھا ہے اگر پسند آیا ہے تو اک نظر میرے چینل پر بھی ڈال دو | 0 |  |  |  |  |  |  |  |  |  |  |
|  | ونتی اگروال وہ بہت پیارا ہے دیوالی مبارک ہو | 0 |  |  |  |  |  |  |  |  |  |  |
|  | وہ اترو گانا ایک دم کول تھا | 0 |  |  |  |  |  |  |  |  |  |  |
|  | وہ امریکہ میں رہتا ہے اپنے آپ کو پروفیسر ڈاکٹر کہلاتا ہے پر ہے سب سے بڑا چوتیا مادر چود | 1 |  |  |  |  |  |  |  |  |  |  |
|  | وہ بلکل چوتیا بھوسڑی کا ہے | 1 |  |  |  |  |  |  |  |  |  |  |
|  | وہ تو چوتیا ہے پتہ نہں اپنے آپ کو کیا سمجھتا رہتا ہے | 1 |  |  |  |  |  |  |  |  |  |  |
|  | وہ جون سینا اور میاں خلیفہ بول رہا ہے جون سینا نہیں آرہا | 0 |  |  |  |  |  |  |  |  |  |  |
|  | وہ حصہ سب سے بہتر ہے | 0 |  |  |  |  |  |  |  |  |  |  |
|  | وہ دن گئے جو اچھے تھے | 0 |  |  |  |  |  |  |  |  |  |  |
|  | وہ دونوں بہت مادرچود ہیں ان کی ماں اور بہن کو دیکھ کر کچھ کچھ ہوتا ہے تم بھی پہلے دیکھ لو پھر ان پر ویڈیو بناؤ | 1 |  |  |  |  |  |  |  |  |  |  |
|  | وہ سب تو ٹھیک ہے پر لائیکس | 0 |  |  |  |  |  |  |  |  |  |  |
|  | وہ سب کیری سے جلتے ہیں لیکن تمہاری برابری نہیں کر سکتے | 0 |  |  |  |  |  |  |  |  |  |  |
|  | وہ سرپرائز واؤ اور ویڈیو بھی ایک دم ہٹ کر اور ہاں آخری والا پارٹ بھی چاہیے پر جلدی | 0 |  |  |  |  |  |  |  |  |  |  |
|  | وہ گانا بہت عمدہ تھا مجھے بہت پسند آیا | 0 |  |  |  |  |  |  |  |  |  |  |
|  | وہ گانا ہا ہا بہت اچھا گزارش ہے کہ اس طرح کے اور بھی گانے بنائیں | 0 |  |  |  |  |  |  |  |  |  |  |
|  | وہ گانڈو بناتا ہے سب کو آپ نے تو اس کو کہ کر لے لی | 1 |  |  |  |  |  |  |  |  |  |  |
|  | وہ مشہور نعمان خان کا باپ ہے | 0 |  |  |  |  |  |  |  |  |  |  |
|  | وہ ہمیشہ چوسیاں ہی مارتا ہے شام ادیس چوتیا بندہ ہے | 1 |  |  |  |  |  |  |  |  |  |  |
|  | وہ والا ریپ بھائی جان سے کہ کر لے لے | 0 |  |  |  |  |  |  |  |  |  |  |
|  | وہ والا لائیک میرا ہے | 0 |  |  |  |  |  |  |  |  |  |  |
|  | وہ وہ کر کے دیکھانا | 0 |  |  |  |  |  |  |  |  |  |  |
|  | وے مجھے تیری آخری والی ویڈیو چاہیے تو کیسے ہیں آپ لوگ ری مکس | 0 |  |  |  |  |  |  |  |  |  |  |
|  | ویڈوی ختم ہو گئی بھائی تھک گئے ہم لیکن ان بہن چودوں کی بہن چودیاں ختم نہں ہوئیں ابھی تک | 1 |  |  |  |  |  |  |  |  |  |  |
|  | ویڈیو اور وہ بھی انکل مجبور پر | 0 |  |  |  |  |  |  |  |  |  |  |
|  | ویڈیو بہت لمبی ہو گئی ہے بہت مزہ آئے گا | 0 |  |  |  |  |  |  |  |  |  |  |
|  | ویڈیو تبدیل کرنے کا کون سا سافٹ وئیر آپ استعمال کرتے ہو | 0 |  |  |  |  |  |  |  |  |  |  |
|  | ویڈیو ڈالیں شوٹر بھائی کب سے آپ کا انتظار کر رہا ہوں اگر نہیں بنا سکتے تو پلیز بتا دو | 0 |  |  |  |  |  |  |  |  |  |  |
|  | ویڈیو کے شروع میں تیری داڑھی ہے لیکن آخر میں نہیں ہے | 0 |  |  |  |  |  |  |  |  |  |  |
|  | ویڈیو کے لنک کا ایرر آرہا ہے | 0 |  |  |  |  |  |  |  |  |  |  |
|  | ویڈیو لمبی ہے کافی پر تم نے چوتیا کی لے لی | 1 |  |  |  |  |  |  |  |  |  |  |
|  | ویڈیوبہت اعلیٰ تھی لیکن آخری والا منظر جگ جگ جیو بھائی | 0 |  |  |  |  |  |  |  |  |  |  |
|  | ویڈیوکچھ دیکھنے والوں کو بہت اچھی ہے اچھی کام ہوا ہے | 0 |  |  |  |  |  |  |  |  |  |  |
|  | ویرا ان کو صیح چوتیا کہتا ہے | 1 |  |  |  |  |  |  |  |  |  |  |
|  | ویرے مجھے اپ کی ویڈیوز سے پیار ہے | 0 |  |  |  |  |  |  |  |  |  |  |
|  | ویسے اس بڈھے گانڈل کا نام کیا ہے چیینل تلاش کرنا تھا | 1 |  |  |  |  |  |  |  |  |  |  |
|  | ویسے اس کا حادثہ اصلی تھا | 0 |  |  |  |  |  |  |  |  |  |  |
|  | ویسے بھائی آپ کی ویڈیوز بہت اچھی ہیں اس کو جاری کھیں اور ہمیں خوش کرتے رہیں | 0 |  |  |  |  |  |  |  |  |  |  |
|  | ویسے تم چھا گئے ہو بھائی اس کو جاری رکھو | 0 |  |  |  |  |  |  |  |  |  |  |
|  | ویسے تم چھا گئے ہو بھائی آگے بڑھو اور اس کو جاری رکھو | 0 |  |  |  |  |  |  |  |  |  |  |
|  | ویسے تمہارے پاس اور کوئی کام نہیں ہے کرنے کو | 0 |  |  |  |  |  |  |  |  |  |  |
|  | ویسے رجسٹریشن کیلئے شکریہ | 0 |  |  |  |  |  |  |  |  |  |  |
|  | ویسے شلوار قمیض میں تم بہت پیارے لگ رہے ہو | 0 |  |  |  |  |  |  |  |  |  |  |
|  | ویسے لڑکی جو گانا گاتی ہے وہ بھی بہت بڑی چوتیا ہے | 1 |  |  |  |  |  |  |  |  |  |  |
|  | ویسے میں آسام سے ہوں لیکن آپ درست ہو | 0 |  |  |  |  |  |  |  |  |  |  |
|  | ویسے یہ گانا بہت اچھا تھا | 0 |  |  |  |  |  |  |  |  |  |  |
|  | ویوز لاکھوں اور لاکھوں کمنٹس | 0 |  |  |  |  |  |  |  |  |  |  |
|  | ویوز لائیکس اور کمنٹس واؤ | 0 |  |  |  |  |  |  |  |  |  |  |
|  | یاد کرلو آگے جا کر کام آئےگا | 0 |  |  |  |  |  |  |  |  |  |  |
|  | یار اتنی دیر ویڈیو مت ڈالا کر کب سے انتظار کر رہے ہیں | 0 |  |  |  |  |  |  |  |  |  |  |
|  | یار اس کنجری کے بچے نے سب کو پاگل بنایا ہوا تھا ان دونوں بہن چودوں نے کمال کر دیا | 1 |  |  |  |  |  |  |  |  |  |  |
|  | یار اگر ان نامونوں کی اگر کوئی کھجلی مٹا سکتا ہے تو وہ ھے کیری | 0 |  |  |  |  |  |  |  |  |  |  |
|  | یار بھائی ویڈیو اپ لوڈکرو مہینہ ہو گیا | 0 |  |  |  |  |  |  |  |  |  |  |
|  | یار تم سب میری اگلی مزاحیہ ویڈیو دیکھ لو مزہ آجائے گا | 0 |  |  |  |  |  |  |  |  |  |  |
|  | یار تم کو واقعی میں لاکھوں سبسکرائبرز ملنے چائیں پھیر دیکھیں گے کہ چھوٹا یوٹیوبر ہے کون سالا | 1 |  |  |  |  |  |  |  |  |  |  |
|  | یار تمہاری چیزیں پاکستان میں ہوں گی کیوں کہ میں بھی پاکستان سے ہوں اور میں آپ کا سب بہت بڑا فائن ہوں | 0 |  |  |  |  |  |  |  |  |  |  |
|  | یار دکی بھائی اس مادر چود کے بچے نے حجاب بھی بند کردیا ہے اپنی ویڈیوز پر | 1 |  |  |  |  |  |  |  |  |  |  |
|  | یار دکی بھائی ویڈیو کا موضوع تبدیل کرلیا کرو شام کو چود دے | 1 |  |  |  |  |  |  |  |  |  |  |
|  | یار سنجیدگی سے کہ رہا ہوں کام کروا کروا کر بہن چود دی | 1 |  |  |  |  |  |  |  |  |  |  |
|  | یار کیوں لنڈ فراگی کا چینل دیکھتے ہو اور شام چوتیا کا چینل بھی فراگی کی وجہ سے چلتا ہے | 1 |  |  |  |  |  |  |  |  |  |  |
|  | یار گانڈو تو نے تو ہمارا ٹائم ہی ضائع کر دیا کیا فضول انسان ہے شام | 1 |  |  |  |  |  |  |  |  |  |  |
|  | یار گانڈو میں تمہں فالو کرتا ہوں | 1 |  |  |  |  |  |  |  |  |  |  |
|  | یار مادرچود کیا اپنے نہانے کی ویڈیو بھی ڈال دو گے اہ دلے | 1 |  |  |  |  |  |  |  |  |  |  |
|  | یار مزہ آگیا | 0 |  |  |  |  |  |  |  |  |  |  |
|  | یار مطلب گانڈ ہی پھاڑ دے بندے کی | 1 |  |  |  |  |  |  |  |  |  |  |
|  | یار منڈل مست ہے | 0 |  |  |  |  |  |  |  |  |  |  |
|  | یار میرا خیال ہے حجاب والا ایکٹ بھی کام نہیں کرے گا وہ بھوسڑی کے بہن چود یہ بھی بند کرے گا | 1 |  |  |  |  |  |  |  |  |  |  |
|  | یار میں اس کو نہں چھوڑوں گا اور نہ ہی اس کے چینل کو کیوں کہ میں دیکھنا چاہتا ہوں کہ اس سے بڑا چوتیا کوئی اور ہے یا نہیں | 1 |  |  |  |  |  |  |  |  |  |  |
|  | یار نظر انداز کرہ بس گانڈو کو | 1 |  |  |  |  |  |  |  |  |  |  |
|  | یار وہ انٹرویو دیکھ کر تاؤ کی یاد آگئی یار لیکن آپ کی یوٹیوب سے کام کی لگن کی بہت تعریف کرتا ہوں | 0 |  |  |  |  |  |  |  |  |  |  |
|  | یار وہ پھر سٹرائیک لگا دے حرامی آپ کی ویڈیو پر بھائی چھوڑ نا اس چوتیا کو | 1 |  |  |  |  |  |  |  |  |  |  |
|  | یار یہ چوتیا کس کی گانڈ سے پیدا ہوا ہے | 1 |  |  |  |  |  |  |  |  |  |  |
|  | یار یہ عوام چوتیے لوگوں کو فالو کرتی ہے | 1 |  |  |  |  |  |  |  |  |  |  |
|  | یار یہ کون سے درامے کا گانا ہےسننے کو بہت دل کر رہا ہے | 0 |  |  |  |  |  |  |  |  |  |  |
|  | یار یہ ویڈیوبڑی غضب تھی | 0 |  |  |  |  |  |  |  |  |  |  |
|  | یارا کمال کرتے ہو اس پھدو کی صیح طرح کرکے گانڈ مارو | 1 |  |  |  |  |  |  |  |  |  |  |
|  | یاش پٹیل نے اپنی سفر کی ویڈیوز بنانی شروع کردی ہیں ایک بار ضرور دیکھیں اور اگر پسند آئیں تو لائیک ضرور کریں | 0 |  |  |  |  |  |  |  |  |  |  |
|  | یاشپال سنگھ تو مرنہیں صرف لاکھوں | 0 |  |  |  |  |  |  |  |  |  |  |
|  | یعنی شام پہلے بھی کتا تھا بہن چود عوام کو پاگل بنایا ہوا عوام بھی اسی چوتیا کہ رہی ہوتی | 1 |  |  |  |  |  |  |  |  |  |  |
|  | یہ ابھی بھی بہت دلچسپ ہے | 0 |  |  |  |  |  |  |  |  |  |  |
|  | یہ ایسے ہی ہے جیسے کچھ سال پہلے میں نے زیدی کا چینل لائیک کیا تھا وہ بھی جھوٹی ویڈیوز بناتاتھا پر یہ شام تو بہت بڑا چوتیا نکلا یار | 1 |  |  |  |  |  |  |  |  |  |  |
|  | یہ ایک گھنٹہ کی ویڈیو ہے لیکن اس بھڑوے کو ذلیل کرنے کے لیے ایک منٹ ہی کافی ہے | 1 |  |  |  |  |  |  |  |  |  |  |
|  | یہ آپ کا سب سے اچھا کام ہے | 0 |  |  |  |  |  |  |  |  |  |  |
|  | یہ آپ کی سب سے بہترین ویڈیو ہے | 0 |  |  |  |  |  |  |  |  |  |  |
|  | یہ آخر میں جو گانا تھا وہ کس کا ہے کوئی بتائے گا | 0 |  |  |  |  |  |  |  |  |  |  |
|  | یہ بندہ بہت گالیاں نکالتا ہے بات بات پر اک اک جملہ میں گالی دیتا ہے | 1 |  |  |  |  |  |  |  |  |  |  |
|  | یہ بندہ شکل سے ہی بغیرت لگتا ہے تربیت کی کمی حرام کما رہا | 1 |  |  |  |  |  |  |  |  |  |  |
|  | یہ بندہ گانڈو بے غیرت مادرچود دلا چوتیاہے | 1 |  |  |  |  |  |  |  |  |  |  |
|  | یہ بندے مجھے شروع سے اچھے نہں لگتے چوتیے ہیں یہ دونوں | 1 |  |  |  |  |  |  |  |  |  |  |
|  | یہ بہت شاندار چھوٹی سی کہانی تھی | 0 |  |  |  |  |  |  |  |  |  |  |
|  | یہ بہن چود ادھر آئے گا اور کبھی ملے گا تو گانڈ ماروں گا میں اس کی | 1 |  |  |  |  |  |  |  |  |  |  |
|  | یہ بہن چود ادھر بھی آگیا | 0 |  |  |  |  |  |  |  |  |  |  |
|  | یہ بہن چود چوتیا ابلیس بدنام کر رہا ہے اسلام کو حجاب کو اور پاکستان کو | 1 |  |  |  |  |  |  |  |  |  |  |
|  | یہ بہن چود لوگ ہیں ہی ایسے ان لوگوں کی ایسے ہی میں نے ویڈیو دیکھی مادر چود | 1 |  |  |  |  |  |  |  |  |  |  |
|  | یہ بھائی آپ کی سب سے شاندارکام ہے | 0 |  |  |  |  |  |  |  |  |  |  |
|  | یہ بھائی بہن چود بھڑوا ہے | 1 |  |  |  |  |  |  |  |  |  |  |
|  | یہ بھی بہت زیادہ اداکاری کرتا ہے چوتیا انگریز کا بچا اپنی اوقات کتنی جلدی بھول جاتا ہے شا باش دکی بھائی | 1 |  |  |  |  |  |  |  |  |  |  |
|  | یہ بھی چوتیا ہے اس جیسا | 1 |  |  |  |  |  |  |  |  |  |  |
|  | یہ بھی کتے سے گیدڑ بن گیا تو اب باتیں کر رہا ہے | 1 |  |  |  |  |  |  |  |  |  |  |
|  | یہ بیک گراؤنڈ پر کون سا گیم چل رہا ہے | 0 |  |  |  |  |  |  |  |  |  |  |
|  | یہ پاکستانی اس لیے کہ رہا ہے کہ پاکستانیوں کی کوئی عزت نہں کرتا مادر چوس | 1 |  |  |  |  |  |  |  |  |  |  |
|  | یہ تھوڑا بہت اچھا ہے لیکن یہ آپ کی اچھی آواز کے معیار کی وجہ سے ہے | 0 |  |  |  |  |  |  |  |  |  |  |
|  | یہ تو ٹائیگر شروف کا بھی باپ ہے | 0 |  |  |  |  |  |  |  |  |  |  |
|  | یہ ٹھنڈی فلم ہے بھوسڑی کا بچہ | 1 |  |  |  |  |  |  |  |  |  |  |
|  | یہ چھوٹے یوٹیوبر کو کیوں تنگ کرتے ہیں اگر ہم چھوٹے ہیں تو ہم کیوں ان کی گانڈ میں ڈنڈا بن کر چبھ رہے ہیں | 1 |  |  |  |  |  |  |  |  |  |  |
|  | یہ چوتیا کاپی رائٹ لگائے گا س پر | 1 |  |  |  |  |  |  |  |  |  |  |
|  | یہ حقیقی چینل نہیں ہے میں نے اصل سمجھ کر اس کو ریپ کیا تھا | 0 |  |  |  |  |  |  |  |  |  |  |
|  | یہ دونو چوتیا ہیں | 1 |  |  |  |  |  |  |  |  |  |  |
|  | یہ دونو سالے بھوسڑی کے ویوز کے لیے کچھ بھی کر سکتے ہیں | 1 |  |  |  |  |  |  |  |  |  |  |
|  | یہ دونوں دنیا کے سب سے بڑے چوتیا ہیں | 1 |  |  |  |  |  |  |  |  |  |  |
|  | یہ دونوں لوگوں کو چوتیا بنانے میں لگے ہیں | 1 |  |  |  |  |  |  |  |  |  |  |
|  | یہ دونوں مل کر چوتیا کر رہے ہیں جھوٹے لوگ پلیز مجھے بتائیں کہ میں ان ویڈیوز کو کیسے روک سکتا ہوں | 1 |  |  |  |  |  |  |  |  |  |  |
|  | یہ دیکھ کر انداہ لگایا جا سکتا ہے کہ سندھ ایک دلچسپ جگہ ہے | 0 |  |  |  |  |  |  |  |  |  |  |
|  | یہ روپالی یہ روپالی پکڑ میری ڈالی | 0 |  |  |  |  |  |  |  |  |  |  |
|  | یہ سارے مل کر ہم کو پاگل بنا رہے مادر چود کے بچے شادی کر لے | 1 |  |  |  |  |  |  |  |  |  |  |
|  | یہ سارے یوٹیوبر مل کر ہم سب کو پاگل بنا رہے ہیں مادر چود کے بچے | 1 |  |  |  |  |  |  |  |  |  |  |
|  | یہ ساس کی گانڈکا سائز کیا ہے | 1 |  |  |  |  |  |  |  |  |  |  |
|  | یہ سالا ہے ہی بہن چود | 1 |  |  |  |  |  |  |  |  |  |  |
|  | یہ سب برے ہیں کیا | 0 |  |  |  |  |  |  |  |  |  |  |
|  | یہ سب بہن کے ٹکے ہیں دونوں چوتیا | 1 |  |  |  |  |  |  |  |  |  |  |
|  | یہ سب تم ویوز کے لیے کر رہے ہو بہن چود | 1 |  |  |  |  |  |  |  |  |  |  |
|  | یہ سب قسمت کی باتیں ہیں ہم جو کچھ کرتے ہیں وہ ٹھیک نہں ہے۔ | 0 |  |  |  |  |  |  |  |  |  |  |
|  | یہ سب مل کر ہم کو چوتیا بنا رہے رہیں وہ بک گیا ہے اس کے پاس پیچھے کچھ بھی نہیں رہ گیا | 1 |  |  |  |  |  |  |  |  |  |  |
|  | یہ سب ہم کو مل چوتیا بنا رہے ہیں ابلیس لوگ | 1 |  |  |  |  |  |  |  |  |  |  |
|  | یہ سچ ہے بھائی کہ یہ کام بالکل اچھا نیہں ہے | 0 |  |  |  |  |  |  |  |  |  |  |
|  | یہ شام کم عقل چوتیا ہے اسکی توصیح کر کے لے رہے ہو | 1 |  |  |  |  |  |  |  |  |  |  |
|  | یہ شرٹ دن سے پہن رہا ہوں ہینڈ کرکٹ سکول کا سب سے بڑا سپورٹس کی تقریب | 0 |  |  |  |  |  |  |  |  |  |  |
|  | یہ صیح نہیں ہو رہا لیکن آپ کسی کی پراپرٹی کو نقصان نہ پہنچائیں | 0 |  |  |  |  |  |  |  |  |  |  |
|  | یہ فراگی رنڈی کی وجہ سے چلتا ہے | 1 |  |  |  |  |  |  |  |  |  |  |
|  | یہ کیا بہن چودی ہے | 1 |  |  |  |  |  |  |  |  |  |  |
|  | یہ گشتی کے بچے باس نہیں آنے والے بہن چود ایک ویڈیو دیکھی تھی | 1 |  |  |  |  |  |  |  |  |  |  |
|  | یہ گلوریا آیا ہوا تھا اس بےشرم اور بےغیرت کو کسی نے پوچھا تک نہں سالا پوری دنیا کا | 1 |  |  |  |  |  |  |  |  |  |  |
|  | یہ گندی گندی گالیاں بھی دینی تھیں نہ | 1 |  |  |  |  |  |  |  |  |  |  |
|  | یہ لڑکے لیکن یہ ویڈیو بہت اچھی ہے | 0 |  |  |  |  |  |  |  |  |  |  |
|  | یہ لوگ پاگل ہیں جوتم کو روسٹ کرتے ہیں جلتے ہیں تم سے اصل میں | 0 |  |  |  |  |  |  |  |  |  |  |
|  | یہ موہت کون ہے رے | 0 |  |  |  |  |  |  |  |  |  |  |
|  | یہ ہمارا مسئلہ ہے کہ ہم ان فراگ چوتیے کی ویڈیوز دیکھتے ہیں | 1 |  |  |  |  |  |  |  |  |  |  |
|  | یہ ہے نا گانڈو کی اولاد کتے کا بچہ رنڈی کا بھائی شام اس نے بہت ذلیل کیا ہوا تھا صیح سبق سیکھایا تم نے اس کو | 1 |  |  |  |  |  |  |  |  |  |  |
|  | یہ وہ انڈین مادرچود ہے جو پیسے کما کر اپنی گانڈ میں ڈالتا ہے | 1 |  |  |  |  |  |  |  |  |  |  |
|  | یہ وہ بات ہے جو کیری کو لائیو لاتی ہے حقیقت میں ایسی ویڈیوز بنانا مشکل ہوتا ہے | 0 |  |  |  |  |  |  |  |  |  |  |
|  | یہ وہی لوگ ہیں جو مروا مروا کر ایجنٹوں سے لندن فرانس میں پھر پھٹی ہوئی گانڈ لے کر واپس آجاتے ہیں | 1 |  |  |  |  |  |  |  |  |  |  |
|  | یہ ویڈیو اصلی ہے کیا اس کا کوئی لنک دو | 0 |  |  |  |  |  |  |  |  |  |  |
|  | یہ ویڈیو بہت اچھی ہے | 0 |  |  |  |  |  |  |  |  |  |  |
|  | یہ ویڈیو بہت اچھی ہے | 0 |  |  |  |  |  |  |  |  |  |  |
|  | یہ ویڈیو دیکھ کر سندیپ کی پھٹ جائے گی اور وہ ایک اور چبل مارےگا اور مزہ آجائے گا جب اس کی گانڈ پھٹ جائے گی تو | 1 |  |  |  |  |  |  |  |  |  |  |
|  | یہ ویڈیو کون سی ہے | 0 |  |  |  |  |  |  |  |  |  |  |
|  | یہاں آنے کے لئیےشکریہ | 0 |  |  |  |  |  |  |  |  |  |  |
|  | یہی آخری ویڈیو ہے | 0 |  |  |  |  |  |  |  |  |  |  |
|  | یوٹیوب ایکس میں جا کر ماں چدوا | 1 |  |  |  |  |  |  |  |  |  |  |
|  | یوٹیوب کے دوسرے ویڈیوز کو بھی آپ دیکھو زیادہ مزہ آئیگا | 0 |  |  |  |  |  |  |  |  |  |  |
|  | یوٹیوبر ہا ہا ہا | 0 |  |  |  |  |  |  |  |  |  |  |

 Go

## Footer

© 2022 GitHub, Inc.

### Footer navigation

- Terms
- Privacy
- Security
- Status
- Docs
- Contact GitHub
- Pricing
- API
- Training
- Blog
- About

You can’t perform that action at this time.

You signed in with another tab or window. Reload to refresh your session.
You signed out in another tab or window. Reload to refresh your session.
