# Supplementary material for: Detection of offensive terms in resource-poor language using machine learning algorithms
Source: PeerJ Comput Sci. 2023 Aug 29;9:e1524. doi: 10.7717/peerj-cs.1524 (PMC10496005; doi:10.7717/peerj-cs.1524)
Supplement: Supplemental Information 3 [file peerj-cs-09-1524-s003.htm]

Resource-Poor-Language-Urdu-Dataset/Stopword.csv at main · owais4321/Resource-Poor-Language-Urdu-Dataset · GitHub


Skip to content


Toggle navigation

Sign up

- Product

  - Actions

    Automate any workflow
  - Packages

    Host and manage packages
  - Security

    Find and fix vulnerabilities
  - Codespaces

    Instant dev environments
  - Copilot

    Write better code with AI
  - Code review

    Manage code changes
  - Issues

    Plan and track work
  - Discussions

    Collaborate outside of code
  - Explore
  - All features
  - Documentation
  - GitHub Skills
  - Blog
- Solutions

  - For
  - Enterprise
  - Teams
  - Startups
  - Education
  - By Solution
  - CI/CD & Automation
  - DevOps
  - DevSecOps
  - Case Studies
  - Customer Stories
  - Resources
- Open Source

  - GitHub Sponsors

    Fund open source developers
  - The ReadME Project

    GitHub community articles
  - Repositories
  - Topics
  - Trending
  - Collections
- Pricing

- In this repository

  All GitHub
  ↵

  Jump to
  ↵

- No suggested jump to results

- In this repository

  All GitHub
  ↵

  Jump to
  ↵
- In this user

  All GitHub
  ↵

  Jump to
  ↵
- In this repository

  All GitHub
  ↵

  Jump to
  ↵

Sign in

Sign up

{{ message }}

owais4321
/
**Resource-Poor-Language-Urdu-Dataset**
Public

- Notifications
- Fork
  0
- Star
   0

- Code
- Issues
  0
- Pull requests
  0
- Actions
- Projects
  0
- Security
- Insights

More


- Code
- Issues
- Pull requests
- Actions
- Projects
- Security
- Insights

Permalink

main

Switch branches/tags


Branches
Tags

Could not load branches


Nothing to show

{{ refName }}
default
View all branches

Could not load tags


Nothing to show


{{ refName }}
default
View all tags

# Name already in use

A tag already exists with the provided branch name. Many Git commands accept both tag and branch names, so creating this branch may cause unexpected behavior. Are you sure you want to create this branch?

 Cancel
 Create

## Resource-Poor-Language-Urdu-Dataset/**Stopword.csv**

 Go to file

 

- Go to file
  T
- Go to line
  L
- Copy path
- Copy permalink

This commit does not belong to any branch on this repository, and may belong to a fork outside of the repository.

Cannot retrieve contributors at this time

266 lines (266 sloc)
2.06 KB

Raw
  Blame

Edit this file

E


Open in GitHub Desktop

- Open with Desktop
- View raw
- Copy raw contents
   Copy raw contents

   Copy raw contents

   Copy raw contents
- View blame

We can make this file beautiful and searchable if this error is corrected: No commas found in this CSV file in line 0.

This file contains bidirectional Unicode text that may be interpreted or compiled differently than what appears below. To review, open the file in an editor that reveals hidden Unicode characters.
Learn more about bidirectional Unicode characters

Show hidden characters


|  |  |
| --- | --- |
|  | Stopwords |
|  | اب |
|  | ابھی |
|  | اپنا |
|  | اپنے |
|  | اپنی |
|  | اٹھا |
|  | اس |
|  | اسے |
|  | اسی |
|  | اگر |
|  | ان |
|  | انہوں |
|  | انہی |
|  | انہیں |
|  | انھیں |
|  | او |
|  | اور |
|  | اے |
|  | ایسا |
|  | ایسے |
|  | ایسی |
|  | ایک |
|  | آ |
|  | آپ |
|  | آتا |
|  | آتے |
|  | آتی |
|  | آگے |
|  | آنا |
|  | آنے |
|  | آنی |
|  | آئے |
|  | آئی |
|  | آئیں |
|  | آیا |
|  | با |
|  | بڑا |
|  | بڑے |
|  | بڑی |
|  | بعد |
|  | بعض |
|  | بلکہ |
|  | بہت |
|  | بھی |
|  | بے |
|  | پاس |
|  | پر |
|  | پہلے |
|  | پھر |
|  | تا |
|  | تاکہ |
|  | تب |
|  | تجھ |
|  | تجھے |
|  | تک |
|  | تم |
|  | تمام |
|  | تمہارا |
|  | تمہارے |
|  | تمھارے |
|  | تمہاری |
|  | تمہیں |
|  | تمھیں |
|  | تھا |
|  | تھے |
|  | تھی |
|  | تھیں |
|  | تو |
|  | تیری |
|  | تیرے |
|  | جا |
|  | جاتا |
|  | جاتی |
|  | جاتے |
|  | جاتی |
|  | جانے |
|  | جانی |
|  | جاؤ |
|  | جائے |
|  | جائیں |
|  | جب |
|  | جس |
|  | جن |
|  | جنہوں |
|  | جنہیں |
|  | جو |
|  | جیسا |
|  | جیسے |
|  | جیسی |
|  | جیسوں |
|  | چاہیئے |
|  | چلا |
|  | چاہے |
|  | چونکہ |
|  | حالاں |
|  | حالانکہ |
|  | دو |
|  | دونوں |
|  | دوں |
|  | دے |
|  | دی |
|  | دیا |
|  | دیں |
|  | دیے |
|  | دیتا |
|  | دیتے |
|  | دیتی |
|  | دینا |
|  | دینے |
|  | دینی |
|  | دیئے |
|  | ڈالا |
|  | ڈالنا |
|  | ڈالنے |
|  | ڈالنی |
|  | ڈالے |
|  | ڈالی |
|  | ذرا |
|  | رکھا |
|  | رکھتا |
|  | رکھتے |
|  | رکھتی |
|  | رکھنا |
|  | رکھنے |
|  | رکھنی |
|  | رکھے |
|  | رکھی |
|  | رہ |
|  | رہا |
|  | رہتا |
|  | رہتے |
|  | رہتی |
|  | رہنا |
|  | رہنے |
|  | رہنی |
|  | رہو |
|  | رہے |
|  | رہی |
|  | رہیں |
|  | زیادہ |
|  | سا |
|  | سامنے |
|  | سب |
|  | سکتا |
|  | سو |
|  | سے |
|  | سی |
|  | شاید |
|  | صرف |
|  | طرح |
|  | طرف |
|  | عین |
|  | کا |
|  | کبھی |
|  | کچھ |
|  | کہہ |
|  | کر |
|  | کرتا |
|  | کرتے |
|  | کرتی |
|  | کرنا |
|  | کرنے |
|  | کرو |
|  | کروں |
|  | کرے |
|  | کریں |
|  | کس |
|  | کسے |
|  | کسی |
|  | کہ |
|  | کہا |
|  | کہے |
|  | کو |
|  | کون |
|  | کوئی |
|  | کے |
|  | کی |
|  | کیا |
|  | کیسے |
|  | کیوں |
|  | کیونکہ |
|  | کیے |
|  | کئے |
|  | گا |
|  | گویا |
|  | گے |
|  | گی |
|  | گیا |
|  | گئے |
|  | گئی |
|  | لا |
|  | لاتا |
|  | لاتے |
|  | لاتی |
|  | لانا |
|  | لانے |
|  | لانی |
|  | لایا |
|  | لائے |
|  | لائی |
|  | لگا |
|  | لگے |
|  | لگی |
|  | لگیں |
|  | لو |
|  | لے |
|  | لی |
|  | لیا |
|  | لیتا |
|  | لیتے |
|  | لیتی |
|  | لیکن |
|  | لیں |
|  | لیے |
|  | لئے |
|  | مجھ |
|  | مجھے |
|  | مگر |
|  | میرا |
|  | میرے |
|  | میری |
|  | میں |
|  | نا |
|  | نہ |
|  | نہایت |
|  | نہیں |
|  | نے |
|  | ہاں |
|  | ہر |
|  | ہم |
|  | ہمارا |
|  | ہمارے |
|  | ہماری |
|  | ہو |
|  | ہوا |
|  | ہوتا |
|  | ہوتے |
|  | ہوتی |
|  | ہوتیں |
|  | ہوں |
|  | ہونا |
|  | ہونگے |
|  | ہونے |
|  | ہونی |
|  | ہوئے |
|  | ہوئی |
|  | ہوئیں |
|  | ہے |
|  | ہی |
|  | ہیں |
|  | و |
|  | والا |
|  | والوں |
|  | والے |
|  | والی |
|  | وہ |
|  | وہاں |
|  | وہی |
|  | وہیں |
|  | یا |
|  | یعنی |
|  | یہ |
|  | یہاں |
|  | یہی |
|  | یہیں |

- Copy lines
- Copy permalink
- View git blame
- Reference in new issue

 Go

## Footer

© 2022 GitHub, Inc.

### Footer navigation

- Terms
- Privacy
- Security
- Status
- Docs
- Contact GitHub
- Pricing
- API
- Training
- Blog
- About

You can’t perform that action at this time.

You signed in with another tab or window. Reload to refresh your session.
You signed out in another tab or window. Reload to refresh your session.
